# Probing the Activation Mechanisms of Agonist DPI-287 to Delta-Opioid Receptor and Novel Agonists Using Ensemble-Based Virtual Screening with Molecular Dynamics Simulations

**DOI:** 10.1021/acsomega.3c01918

**Published:** 2023-08-31

**Authors:** Emily Dean, AnneMarie Dominique, Americus Palillero, Annie Tran, Nicholas Paradis, Chun Wu

**Affiliations:** Department of Molecular & Cellular Biosciences, College of Science and Mathematics, Rowan University, Glassboro, New Jersey 08028, United States

## Abstract

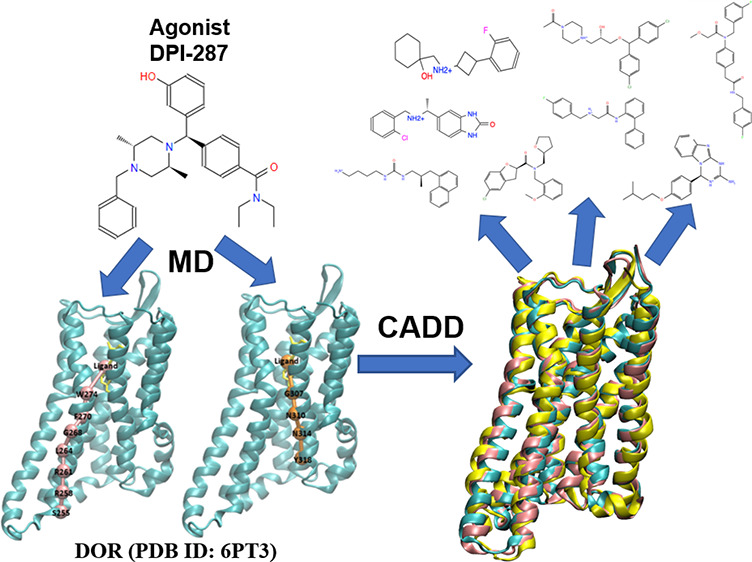

Pain drugs targeting mu-opioid receptors face major addiction problems that have caused an epidemic. The delta-opioid receptor (DOR) has shown to not cause addictive effects when bound to an agonist. While the active conformation of the DOR in complex with agonist DPI-287 has been recently solved, there are still no FDA-approved agonists targeting it, providing the opportunity for structure-based virtual screening. In this study, the conformational plasticity of the DOR was probed using molecular dynamics (MD) simulations, identifying two representative conformations from clustering analysis. The two MD conformations as well as the crystal conformation of DOR were used to screen novel compounds from the ZINC database (17 million compounds), in which 69 drugs were picked as potential compounds based on their docking scores. Notably, 37 out of the 69 compounds were obtained from the simulated conformations. The binding stability of the 69 compounds was further investigated using MD simulations. Based on the MM-GBSA binding energy and the predicted drug properties, eight compounds were chosen as the most favorable, six of which were from the simulated conformations. Using a dynamic network model, the communication between the crystal agonist and the top eight molecules with the receptor was analyzed to confirm if these novel compounds share a similar activation mechanism to the crystal ligand. Encouragingly, docking of these eight compounds to the other two opioid receptors (kappa and mu) suggests their good selectivity toward DOR.

## Introduction

Pain signal processing, or pain perception, occurs when the brain is alerted by electrical signals transmitted by nociceptors due to tissue damage. These electrical signals are transmitted to the central nervous system to alert the brain about potential harm.^1^ The key membrane receptors involving pain signal processing are nociceptors, sensory neurons, glial cells, and postsynaptic neurons. As previously mentioned, nociceptors are responsible for detecting potential damaging stimuli at the skin caused by environmental factors, such as chemicals or heat.^1^ Sensory neurons, such as sodium and potassium channels, are responsible for transmitting pain signals from the wounded area to the central nervous system.^1^ Glial cells, such as toll-like receptors (TLRs), are responsible for releasing the inflammatory mediators. Postsynaptic neurons, which are the opioid receptors, provide analgesic effects when activated.

In pain perception for opioids, the neurotransmitter that is linked to the pain sensation in the central nervous system (CNS) and peripheral nervous system (PNS) is called substance P (SP). SP is a neuropeptide whose role is to act on its receptor, known as the neurokinin-1 (NK1) receptor, to transmit pain signals in the CNS. Nociceptors are responsible for releasing SP following tissue damage.^2^ When SP activates the NK1 receptor, it releases inflammatory mediators, such as histamine and cytokines, from the peripheral nerves.^3^ This process is known as neurogenic inflammation, which enhances pain signals and promotes tissue inflammation.^4^ During pain sensation, the brain signals the release of endorphins to relieve pain and other opioid-associated effects, such as respiratory depression. Endorphins are endogenous opioid peptides that the body produces to bind to opioid receptors for regulating pain perception.^5^ This also leads to the activation of internal opioid receptors caused by external opioids. As a result, it leads to pain relief, euphoria, and respiratory depression. There is no clinical evidence to support that the NK1 receptor antagonist is a good pain-killing drug target in pain management.

Some major pain medicines used to treat pain are opioids, anticonvulsants, and topical analgesics, such as gels. Anticonvulsants are used in neuropathic pain management; they bind to specific subunits of voltage-gated calcium channels to regulate neurotransmitter release and reduce neuronal activity.^6^ In topical analgesics, sodium channels are blocked to reduce pain signals.^7^ Nonetheless, opioid drugs are essential in pain management.

While pharmaceuticals have made great strides over the years in many areas, the field of pain management is still lacking. There are great medications that target pain and are effective; however, there has been a rise in the use of opioids over the years that has led to the opioid epidemic in Northern America and other parts of the world. The use of opioids (legally and illegally) has risen between 10 and 14 times in the last 20 years, with more than 42,000 deaths in 2016 in the USA occurring from opioid overdoses alone.^14,15^ Most prescribed opioids target the mu-opioid receptor (MOR) in Table 1, which are located in the reward areas of the brain.^14,16,17^ When opioid agonists bind to and activate these receptors, it causes euphoria, which can lead to addiction and severe withdrawal symptoms after repeated use. The need for better pain management without addictive properties is thus pressing.

**Table 1:** Comparison between MOR, DOR, and KOR^a^

receptor type	therapeutic function and side effects	model type	reference
mu	pain relief euphoric effect—addiction, physical dependence, respiratory depression	humans	Shipton et al. (2018),^1^ Pergolizzi Jr. et al. (2020),^2^ Volkow et al. (2017),^3^ Centers for Disease et al. (2016)^4^
delta	chronic pain relief	spinal administration rat, gene knockout mice	Holdridge et al. (2007),^6^ Nadal et al. (2006),^7^ Gavériaux-Ruff et al. (2008)^8^
	anti-depression	gene knockout mice, forced swim assay rats	Filliol et al. (2000),^9^ Broom et al. (2002)^10^
	ischemic preconditioning	ischemia reperfusion injury rats, post-ischemic mice	Tian et al. (2013),^11^ Min et al. (2018)^12^
	convulsions	systemic administration mice, rats, electroencephalographic rhesus monkeys	Comer et al. (1993),^8^ Jutkiewicz et al. (2005),^9^ Danielsson et al. (2006)^10^
kappa	analgesia, antidepressant, and anxiolytic effects	humans	Dalefield et al. (2022),^11^ Hang et al. (2015),^12^ Kaski et al. (2021)^13^

a Note: MOR is mu-opioid receptor, DOR is delta-opioid receptor, and KOR is kappa-opioid receptor.

The delta-opioid receptor (DOR) has shown to have potential in not only pain management but also psychiatric and neurological disorders without the potential for dependence or respiratory depression (Table 1).^18−25^ Different DOR agonists were found to not increase tolerance and may be effective in preventing relapse by reducing emotional alterations from withdrawal periods due to the DOR having a role in emotional processing, reduction of pain, and enhancing of moods in animal models.^18−25^ Due to the physiological symptoms that occur when opioid agonists bind to the MOR, this makes the DOR an attractive target to further study to potentially help alleviate the opioid epidemic in the world. Like the DOR, the kappa-opioid receptor (KOR) has also shown to not have addictive properties and does not induce respiratory depression.^11^ When this receptor is activated by its endogenous ligand dynorphin, it produces analgesic, physiological, and behavioral effects (Table 1).^12^ A study has shown that the selective KOR agonists, such as U50, can be considered as a non-addictive alternative without the analgesic effects.^13^

There are multiple studies that have reported convulsions in various animal models with the use of DOR agonists. After systemic administration of a DOR agonist, mice displayed convulsive effects.^8^ Using rats, tolerance rapidly developed to convulsive and locomotor-stimulating effects of a selective DOR agonist but did not display tolerance to the antidepressant-like effects.^9^ When using rhesus monkeys, only one out of the four monkeys had convulsions; however, this same monkey did not display convulsive activity when given a smaller dose weeks later or even the same dose 1 year later.^10^ The difference in convulsions in species could indicate that these are species-dependent effects, and further studies are required to resolve this issue. As mentioned previously, the DOR is distributed in different areas of the spinal cord in rodents versus primates. In rodents, the delta receptor is found dispersed in the spinal cord whereas it is limited to the superficial laminae of the spinal dorsal horn in humans and non-human primates.

Although DOR agonists have displayed convulsive effects in mice, and rhesus monkeys after administration, the DOR is still an attractive target for treating chronic pain, anti-depression, and ischemic preconditioning (Table 1). DPI-287 is an experimental opioid agonist found to induce less convulsions than most drugs in this family and is more selective toward the DOR than MOR or KOR in the rat forced swim test.^58,26^ The DOR has a possible site where morphine tolerance and dependence can be regulated.^61^ Further analysis showed that DPI-287 had weaker docking scores at MOR and KOR and stronger docking scores at DOR, demonstrating a 10-fold selectivity increase for DOR. With DPI-287 being more selective toward the DOR and having reduced risks for convulsions, it makes the DOR an attractive target. Despite this, no FDA-approved drugs currently exist for targeting the DOR; thereby, discovering potential lead compounds for DOR treatment is of utmost importance.

The integration of high-throughput virtual screening (HTVS) in drug discovery is very useful in screening for thousands of molecules that bind favorably to a molecular target and for identifying toxic or unfavorable pharmacodynamic and pharmacokinetic properties of these compounds.^27^ Molecular dynamics (MD) simulations are important in drug discovery due to the significant kinetic and mechanistic information it provides.^64^ Structure-based virtual screening (SBVS) is an HTVS approach that predicts the interactions between ligands and proteins as a complex, ranking them by their affinity to the receptor. The top hit compounds are then selected based on the desired parameters and are then optimized to undergo preclinical and clinical trials. Other computational methods such as molecular modeling are used in the HTVS approach. The process speeds up the drug discovery development by analyzing the interactions of multiple molecules in a shorter period of time, which can look into interactions before the drug is even synthesized. Hence, SBVS is a good technique due to its low cost, faster result time, and good results achieved.

The flexibility of receptors is a challenge researchers face as binding sites usually consist of 10–20 amino acid side chains that have multiple rotatable conformations, which are larger than the rotatable torsions of a ligand.^28^ The movements of the protein backbone can make this even more complex by affecting the conformations of multiple side chains. Using an ensemble-based receptor technique combats this issue in HTVS and MD simulations by sampling the degrees of freedom instead of traditional techniques using one receptor conformation with a flexible ligand. It has been found in cases to also improve docking scores. With previous studies, it has been suggested that ensembles generated from MD simulations have been closely similar in replicating the dynamics of proteins in NMR experiments.^29,30^ It is better to use a few specifically selected conformations as using too many could give false results. In the case of virtual screening, using the top 10% of a compound library subset is more efficient with this approach.^28^ Previous studies using ensemble-based virtual screening have been successful in screening ligands against various drug targets and provide insight for future drug designs.^31,32,65^ The integration of this ensemble-based technique helps to have a better understanding of the structural dynamics of a receptor and have a better understanding of ligand–receptor interactions, which aids in discovering novel ligand binding modes, and helps to develop better therapeutic molecules.^33^

Dynamic network analysis was used to interpret and understand atomic information and structural changes among different regions of the DOR.^63,66^ Unweighted and weighted network models were calculated to decipher the allosteric signal transmission pathway, and this comparison showed their connection to be in good agreement. With MD simulations and network models, this allowed for identification and comparison with interactions of signaling pathways that occur in a system.^62^ Ensemble-based screening was also used in this study, and it is a cost-effective method that has the potential to provide breakthrough discoveries, by providing accurate estimates of free binding energies. To the best of our knowledge, this is the first study to use ensemble-based screening to discover potential agonists to target the DOR.

**Figure 1:**
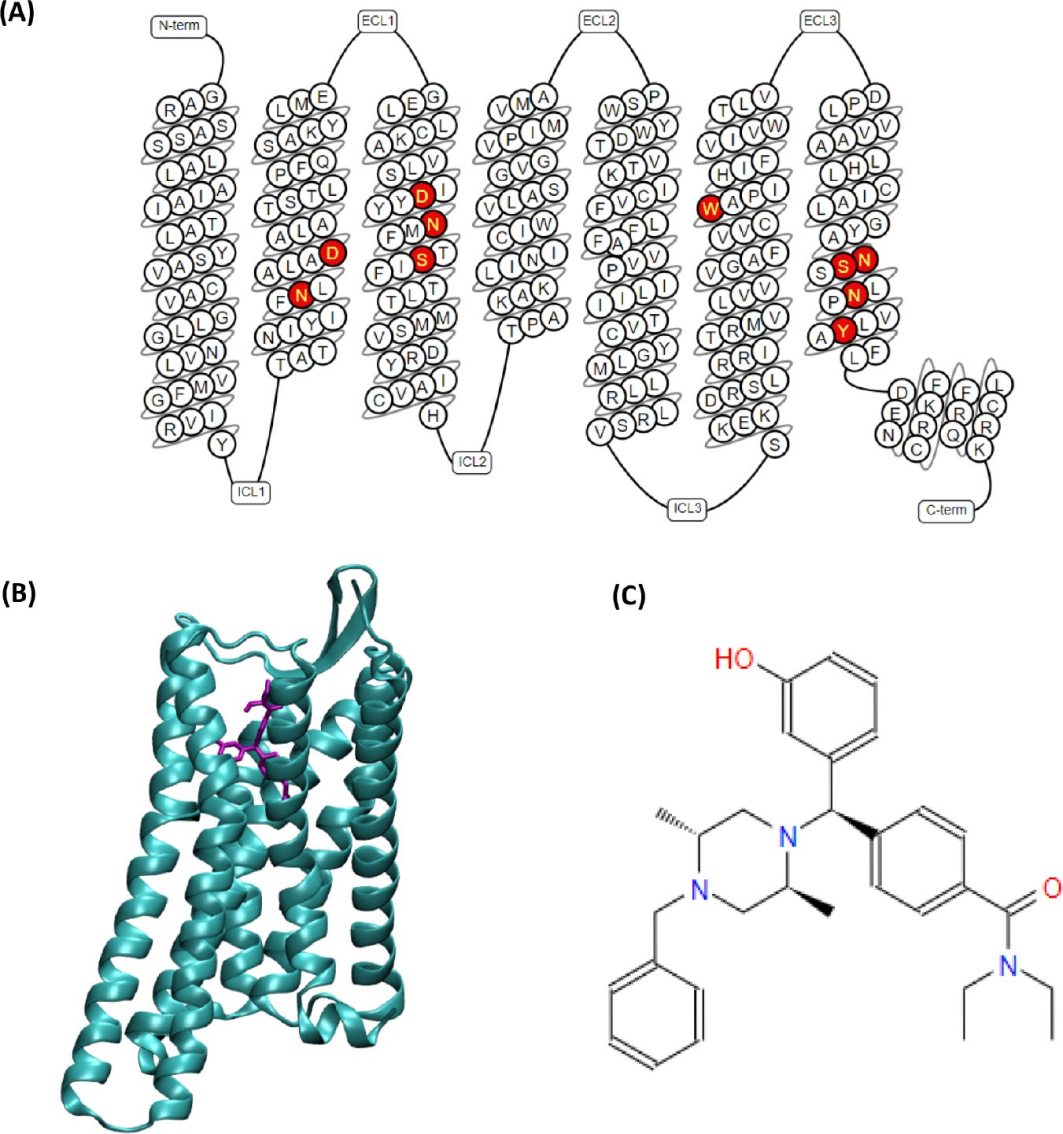
(A) Structural organization of the active conformation of the DOR with the binding pocket residues highlighted in red; *N-terminus (Res: 41–44), TM1 (Res: 45–77), Intracellular loop 1 (Res: 78–82), TM2 (Res: 83–111), Extracellular loop 1 (Res: 112–117), TM3 (Res: 118–153), Intracellular loop 2 (Res: 154–160), TM4 (Res: 161–186), Extracellular loop 2 (beta sheet) (Res: 187–205), TM5 (Res: 206–246), Intracellular loop 3 (Res: 247–249), TM6 (Res: 250–286), Extracellular loop 3 (Res: 287–293), TM7 (Res: 294–321), H8 (Res: 322–327), C-terminus (Res: 328–329)*. (B) DOR in complex with agonist DPI-287 (PDB ID: 6PT3). (C) Chemical structure of DPI-287.

In the present study, MD simulations were used to probe the active conformation of the DOR starting with the active crystal conformation (PDB ID: 6PT3) and the crystal agonist DPI-287 in Figure 1. Using the ensemble-based method, two representative conformations were identified from the clustering and principal component analysis based on the MD simulations. These two conformations and the crystal conformation were used to screen 17 million compounds from the ZINC database. As a result, 69 compounds were identified based on their docking scores. These 69 protein–compound complexes underwent MD simulations to assess their stability. From this, eight compounds were identified that showed significantly improved MM-GBSA binding free energy scores with high blood–brain barrier (BBB) permeability and high gastrointestinal (GI) absorption. This study helps to identify potential compounds to be further tested that will aid in antinociception without addictive or convulsive properties for the DOR.

## Methods

**Figure 2:**
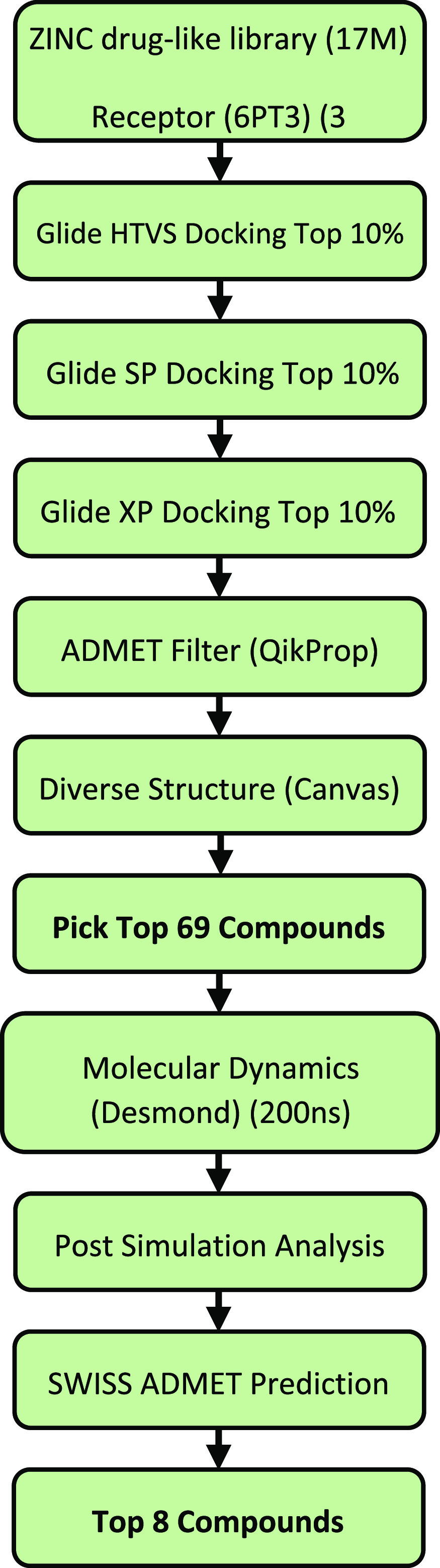
Virtual screening workflow to identify top compounds for DOR from the ZINC15 drug-like library.

Using the ZINC15 “drug-like” library, which contains 17 million entries including FDA-approved drugs (∼1615 entries), a virtual screening workflow (VSW) was developed to identify lead agonists to the DOR (Figure 2) The ZINC library defines “drug-like” using a widely used Lipinski’s Rule of 5: molecular weight (≤500), number of hydrogen bond donors (≤5 H-bonds from the sum of NH and OH), number of hydrogen bond acceptors (≤10 H-bonds from the sum of N and O), and the octanol–water partition coefficient LogP (≤5).^34,35^ The VSW comprises 10 steps including molecular docking, drug property prediction, MD simulations, and post MD-simulation analysis. Inputting the prepared protein structure and ligand library is the first step of the VSW. In steps 2–5, the compounds were then filtered by docking scores and multiple Glide docking score functions that have increasing accuracy (Glide HTVS, SP, and XP). In the next step, a ligand similarity analysis was performed to identify different molecular scaffolds. In step 7, the ligands removed were based on if they had a worse Glide XP score than the reference compound DPI-287 (PDB ID: 6PT3) and/or if they had more than one red flag in drug property (number of stars, from QikProp). QikProp is a Maestro software module that evaluates the drug-like characteristics of a compound by comparing its molecular properties to those of established drugs. Unlike Lipinski’s rule, which uses only four properties, QikProp examines 24 molecular properties. The drug likeness of a specific molecule is forecasted by applying a Gaussian probability distribution function to each of these 24 properties, drawing from data on FDA-approved drugs. For each property, the cutoff value is defined as the value that 95% of FDA-approved drugs adhere to. A compound is awarded a violation star (*) across all of its properties, and the total number of stars is then calculated. As such, fewer stars (0–1 star as per this study) suggest fewer violations and toxicity, indicating a compound is more drug-like. A list of the 24 properties analyzed by QikProp can be found in the supporting document (Table S1).^36^

The top compounds were manually selected from the remaining compounds by maximizing the number of molecular scaffolds (i.e., different ligand cluster IDs). The 200 ns MD simulations were carried out in step 8, followed by the post-MD simulation analyses in step 9, including MM-GBSA binding free energy calculation, simulation interaction diagram analysis, and protein conformation clustering analysis. In the last step, the ADMET (absorption, distribution, metabolism, excretion, and toxicity) prediction was used to examine the human oral bioavailability of potential drug candidates. From this, the compounds with better MM-GBSA binding free energy compared to the reference compound were selected and presented in the main text. The 10 steps are presented in detail in the following six modules.

### 1. Preparation of the Protein and Ligand Library

The crystal structure (PDB ID: 6PT3) of the active conformation of the DOR was prepared and preprocessed using Maestro’s Protein Preparation Wizard.^37^ The preprocessed protein’s charge state was optimized at pH 7. Then, a restrained minimization was performed to relax the protein structure using an OPLS3 force field.^38^ Maestro Elements was used to prepare the 3D structures of DPI-287 and the ZINC compounds and FDA-approved drugs (2466 entries). The 3D structure of DPI-287 was extracted from the crystal structure (PDB ID: 6PT3), and the ZINC compounds and FDA-approved drugs were downloaded from the ZINC15 database (https://zinc15.docking.org/) and the Drug Bank, respectively. In order to generate each ligand’s ionization/tautomeric states at pH 7, Maestro’s Epik tool was used, based on the Hammet and Taft methodologies for increased accuracy.^37^ The lowest ionization/tautomeric state was then chosen. Afterward, the ligand geometry was optimized using quantum mechanics in Maestro’s Jaguar tool.

### 2. Filtering and Docking

The prepared protein and ligand structures were merged into a complex to then be ran through the Schrodinger Virtual Screening Interface using prefilters through Lipinski’s Rule and filtered with ADMET risk parameter assessments through QikProp. The receptor grid files were generated from the prepared receptors, in which the centroid of the crystal ligand, DPI-287, was used to specify the active site. The prepared ligands were docked into their corresponding generated grids using Glide XP scoring with default procedures and parameters.^38^ In detail, the receptor grid required for the docking process was generated using a van der Waals scaling factor of 1 and a partial charge cutoff of 0.25. Docking was performed using a ligand-centered grid and an OPLS3 force field. Glide XP Dock was used perform a comprehensive systematic search for the best receptor conformations and orientations to fit the ligand. The docked poses were compared to the active crystal complex (PDB ID: 6PT3) with the agonist to verify if the docked ligand poses were reasonable. All ligands were bound within the binding pocket with DPI-287 binding similar to the crystal ligand, providing a reasonable starting pose for later molecular dynamic simulations. The binding pose can then be refined given the full conformation flexibility in the simulations. The docking results comprised 69 top ZINC compounds and top 10 FDA-approved compounds. Notably, the 69 top ZINC compounds exhibited much higher docking scores than the reference ligand (PDB ID: 6PT3) and the top 10 FDA-approved compounds, indicating that these ZINC compounds all had high affinity for the receptor. Additional analyses were thus only done for the top 69 ZINC compounds.

### 3. Ligand Similarity Clustering

The Canvas program was used for determining ligand similarity clustering. Canvas uses pharmacophore fingerprinting and hierarchical clustering to further filter out the top 69 ZINC compounds. Pharmacophore fingerprinting identifies similar groups of compounds to match the crystal structure.^39^ Hierarchical clustering forms cluster groups of similar compounds based on their docking score, binding affinity, drug properties, and ligand similarities.^40,41^ Encouragingly, the top 69 ZINC compounds were then used for the MD simulations.

### 4. MD Simulation

#### 4.1. MD Simulation System Setup

The 69 prepared receptor–ligand complexes from Glide XP docking were used as input files to construct MD simulation systems using Desmond System Builder.^38^ Each complex was inserted in a phosphatidylcholine (POPC) lipid membrane,^42^ solvated in an SPC water box of orthorhombic shape with a 6 Å water buffer,^43^ and Na^+^ and Cl^–^ ions neutralized the intrinsic system charge and established a 0.15 M NaCl salt concentration using the OPLS3 force field.

#### 4.2. Relaxation and Production Runs

A default relaxation protocol for membrane proteins in the Desmond module was used to relax the system. The relaxation protocol consisted of eight steps. The first step minimizes the solute heavy atoms with restraints. The second step minimizes the solute heavy atoms without restraints. The third step performs a heat transfer simulation from 0 to 300 K, which led to a H_2_O barrier and gradual restraining. The fourth step performs a simulation under the NPT ensemble (constant pressure (1 bar) and temperature (300 K)) with a H_2_O barrier and heavy atoms becoming restrained. The fifth step performs a simulation under the NPT ensemble to equilibrate the solvent and lipid components. The sixth step performs protein heavy atoms annealing from 10.0 to 2.0 kcal/mol by performing a simulation under the NPT ensemble. The seventh step was to restrain protein Cα atoms at 2 kcal/mol by simulation under the NPT ensemble. The eighth step was to execute simulation for 1.5 ns under the NPT ensemble with no restraints.

After the relaxation protocol, each equilibrated MD system was put through a 200 ns production run under the NPT ensemble using the default protocol. During this process, the Nosé–Hoover chain coupling scheme^44^ was used to control the temperature at a coupling constant of 1.0 ps. The Martyna–Tuckerman–Klein chain coupling scheme^44^ was used to control the pressure at a coupling constant of 2.0 ps. To restrict all bonds connecting hydrogen atoms, M-SHAKE^45^ was applied, enabling a 2.0 fs time step in the simulation. The *k*-space Gaussian split Ewald method^46^ was used to handle long-range electrostatic interactions under periodic boundary conditions. During this step, the charge grid spacing was set to about ∼1.0 Å while the direct sum tolerance was 10^–9^. The short-range non-bonded interactions had a cutoff distance of 9 Å. Meanwhile, the long-range van der Waals interactions were established on a uniform density approximation. An r-RESPA integrator^47^ was used to reduce the computation by calculating the non-bonded forces. The short-range forces were updated at each step, while the long-range forces were updated every three steps. The trajectories at 50.0 ps intervals were saved for further analysis.

### 5. Post-Simulation Analysis

#### 5.1. Simulation Interaction Diagram (SID) Analysis

The Desmond SID tool was used to generate protein/ligand root mean square deviation (RMSD), protein–ligand contacts, secondary structure changes, and protein/ligand root mean square fluctuation (RMSF) measures. The protein Cα and ligand heavy atom RMSD plots were analyzed to check the convergence of MD simulations (i.e., steady-state equilibrium).

#### 5.2. Trajectory Clustering Analysis

For each complex system, the Desmond trajectory clustering tool^48^ was used to combine complex structures from the last 100 ns of each simulation. The backbone RMSD matrix was used as a structural similarity metric, while the hierarchical clustering with average linkage was used as the clustering method. The merging distance cutoff was 2 Å. The centroid structure was represented as the structural family, where the centroid structure represents the largest number of neighbors in the structural family.

#### 5.3. Binding Energy Calculations and Decompositions

The surface-area-based generalized Born model^49,50^ was used to calculate the ligand-binding affinities on the frames, in the last 50 ns of each MD simulation, with an implicit membrane solvation model (VSGB 2.0).^51^ Slab-shaped regions with a low dielectric constant between 1 and 4 were excluded from the implicit membrane and were assigned with the solvent (water) dielectric constant of 80. The MM-GBSA calculation used an OPLS3 force field and the default Prime procedure.^38^ The OPLS3 force field employs a CM1A-BCC-based charge model based on a combination of Cramer–Truhlar CM1A charges^52^ with an extensive parameterization of bond charge correction (BCC) terms. This process begins with minimizing the receptor only, then the ligand only, and then the receptor–ligand complex. Using eq 1, the MM-GBSA binding free energy for each system was calculated from three separate simulations: ligand-only, receptor-only, and the receptor–ligand complex. Equation 2 contains four components: van der Waals interaction energy (VDW), hydrophobic interaction energy (SUR), electrostatic interaction (GBELE), and the change of the conformation energy for the receptor and ligand that were calculated based on eqs 3 and 4.
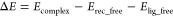
1
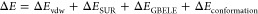
2
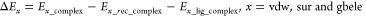
3
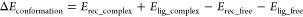
4

The MM-GBSA scoring function lacks the solute conformational entropy contribution, which causes higher negative values when compared to actual values. It is essential to rank a drug’s ability to target a receptor when it is used to rank different drugs targeting receptors with comparable entropy values.^53^ MM-GBSA has shown to be a powerful tool in ranking ligands via their binding affinity to a biological target; multiple benchmarking studies have demonstrated that MM-GBSA binding free energies are good predictors when compared to experimentally determined binding affinities.^54−59^

### 6. ADMET Prediction

ADMET properties were predicted for the best ZINC compounds and were performed on the SwissADME web server (http://www.swissadme.ch/). The SwissADME server was developed by the Swiss Institute of Bioinformatics and is used to provide physiochemical descriptors, ADMET parameters, pharmacokinetic properties, and drug-like small molecules to support drug discovery.^60^ In order to receive each compounds ADMET properties, their respective SMILE codes were inserted into the web server as inputs.

### 7. Normal Mode Analysis

The Normal Mode Wizard plugin in VMD^61^ with default settings was used to generate a main component analysis of the top 10 normal modes for the crystal complex (PDB ID: 6PT3).

### 8. Dynamical Network Model

A dynamic network model, defined as a set of nodes connected by edges,^58,62−65^ was generated using the individual trajectories of each system using the NetworkView plugin in VMD.^66^ We first generated a contact map for each system of the top compounds and the crystal complex, which added an edge between nodes, whose heavy atoms interacted within a cutoff of 4.5 Å for at least 75% of the MD simulation time. The edge distance was derived from pairwise correlations^64^ in the contact map using the program Carma,^67^ which defines the probability of information transfer across a given edge using the following equation:
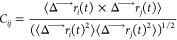
5

The edges in the dynamic network model are weighted (*w_ij_*) between two nodes *i* and *j*, which uses the following calculation: *w*_ij_ = −log(|*C_ij_*|). The weight of the edge is correlated with the probability for information to transfer across the edge between two nodes. Because of this, a thicker edge is characterized as a higher probability of information transfer. The network for each system was further grouped into communities, or subnetworks based on groups of nodes with more frequent and stronger connection to each other, by applying the Girvan–Newman algorithm to the original network.^68^ The critical nodes that connect communities to each other were identified as well. Optimal communication paths were generated between the ligand node and the molecular switch residue number using the data from the molecular switches.

### 9. Selectivity Analysis on Different Opioid Receptors

To determine the relative selectivity of the top eight ZINC compounds to DOR, Glide XP docking of the top eight ZINC compounds was also performed on the KOR and MOR structures. The MOR is taken from our previous studies,^69,70^ which is based on the human MOR homology model built from the crystal structure of mouse MOR in complex with the agonist BU72 (PDB ID: 5C1M). The KOR is taken from the PDB bank (PDB ID: 6B73) and is based on the crystal structure of a nanobody-stabilized active state of KOR. The Glide XP docking scores of the eight ZINC compounds were compared between those of the DOR crystal structure and of the KOR crystal structure and MOR homology model structure. Furthermore, the crystal ligands DPI-287/DOR, MP1104/KOR, and BU27/MOR were also docked to each of the three receptors to serve as reference values.

## Results

### Stability of Crystal Conformation of DOR Maintained during MD Simulation

**Figure 3:**
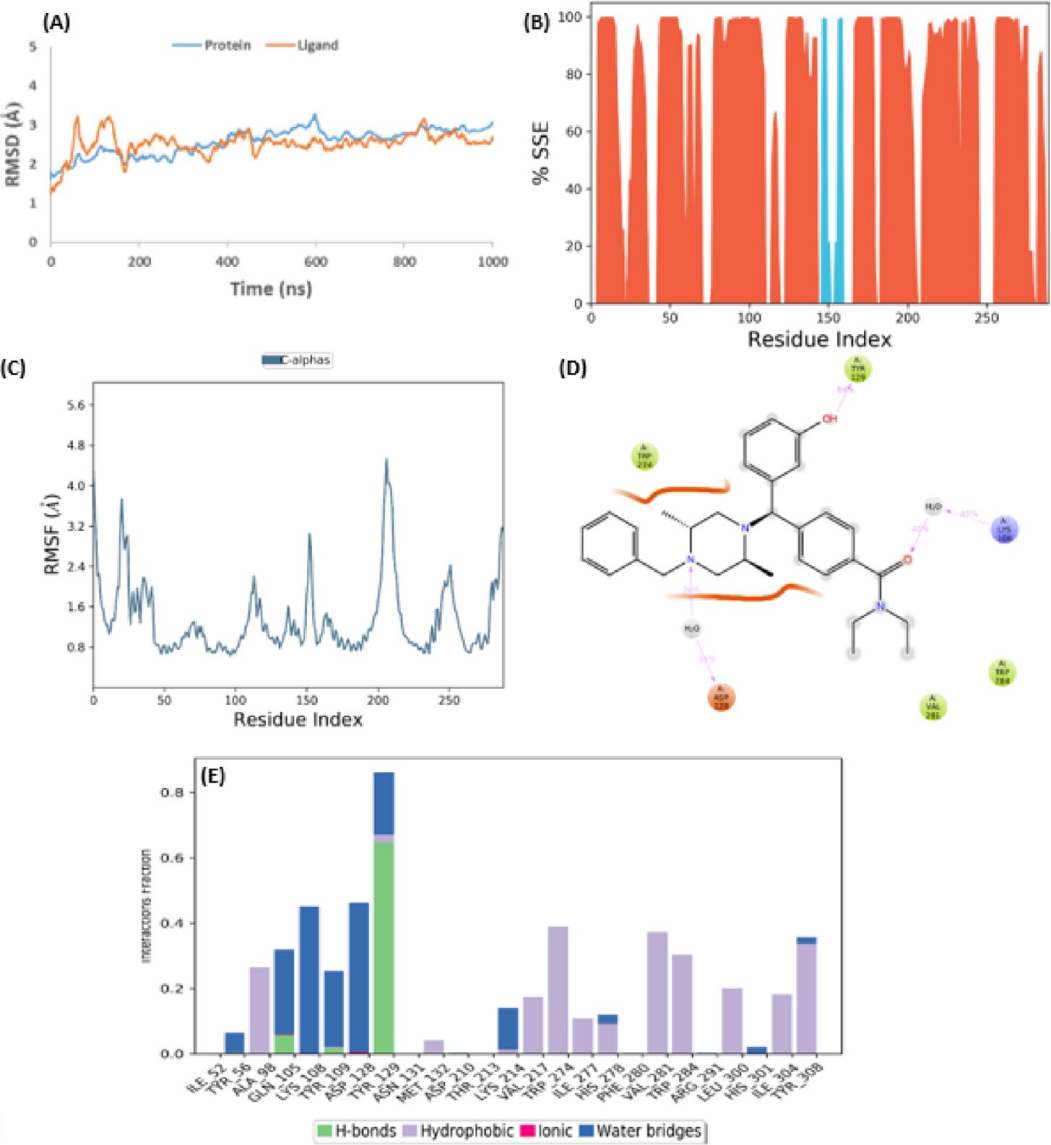
Simulation interaction diagrams after MD simulation of the DOR crystal structure (PDB ID: 6PT3). (A) RMSD plot from MD simulation of 1000 ns. (B) Protein secondary structure elements (SSE). Orange represents alpha helices, and blue represents beta strands. (C) RMSF graph of protein of the crystal complex structure. (D) 2D ligand–protein interaction diagram from the MD trajectory. The residue displayed interacted with ligand for at least 30% of the simulation time. (E) Protein–ligand contacts during MD simulations. Interaction fraction greater than 1 is because of multiple contacts on one residue.

The crystal complex of the active conformation of the human DOR (PDB ID: 6PT3), with crystal agonist DPI-287, was used in the experiment to serve as the control. DPI-287 was first docked back into the crystal conformation and resulted in a similar binding pose with the crystal ligand pose (RMSD = 0.47 Å) and a docking score of −8.6 kcal/mol. The low RMSD value validates the docking protocol (Figure S6, row 1). A 1000 ns MD simulation production run and post-MD simulation analyses were then performed to check the stability of the prepared crystal structure complex. Indeed, the complex was stable throughout the entire trajectory (Figure 3).

To check if the simulation system reached convergence, the protein and ligand RMSD was calculated over the whole 1000 ns trajectory (Figure 3A). The last 200 ns shows that the protein and ligand system components converged with relatively flat plots. The average RMSD values of the protein (2.9 ± 0.1 Å) and ligand RMSD (2.5 ± 0.1 Å) are relatively small, suggesting the crystal complex was stable throughout the trajectory (Table 2).

**Table 2:** Various Properties of the Top Eight ZINC Compounds Identified from our Virtual Screening Workflow^a^

receptor structure	no.	ZINC ID	docking score (kcal/mol)	VDW (kcal/mol)	ELE (kcal/mol)	hydrophobic (kcal/mol)	MM-GBSA (kcal/mol)	receptor RMSD^b^ (Å)	ligand RMSD^b^ (Å)
	ref.	crystal ligand (6PT3): DPI-287	–8.6	–57.0 ± 2.8	18.5 ± 3.7	–51.7 ± 3.5	–90.2 ± 6.3	2.9 ± 0.1	2.5 ± 0.1
CC	(1)	ZINC000020559278	–9.9	–44.7 ± 2.8	–31.3 ± 3.7	–40.8 ± 2.3	–116.9 ± 5.0	2.2 ± 0.1	2.5 ± 0.3
CC	(17)	ZINC000078515864	–8.9	–36.2 ± 3.8	–25.9 ± 5.3	–50.9 ± 2.9	–113.1 ± 6.1	2.6 ± 0.1	2.7 ± 0.2
C1	(1)	ZINC000025329384	–10.2	–57.2 ± 3.6	–19.9 ± 9.4	–57.7 ± 4.1	–134.9 ± 11.3	2.4 ± 0.1	2.9 ± 0.4
C1	(2)	ZINC000037556415	–9.4	–58.5 ± 2.4	–9.6 ± 2.3	–43.8 ± 2.6	–111.9 ± 5.1	3.4 ± 0.2	6.0 ± 0.1
C1	(6)	ZINC000827360794	–9.1	–43.7 ± 3.5	–18.8 ± 8.4	–41.6 ± 2.8	–104.2 ± 11.1	2.4 ± 0.1	2.9 ± 0.1
C1	(9)	ZINC000078648574	–9.0	–50.4 ± 5.2	–4.1 ± 3.5	–54.1 ± 6.5	–108.7 ± 13.1	2.8 ± 0.1	6.3 ± 0.5
C2	(1)	ZINC000057999653	–10.1	–47.1 ± 4.1	–8.6 ± 13.5	–38.9 ± 2.4	–94.6 ± 10.9	2.3 ± 0.1	2.7 ± 0.2
C2	(5)	ZINC000006664413	–9.8	–46.4 ± 2.8	–20.1 ± 7.0	–43.4 ± 3.2	–109.9 ± 8.9	2.6 ± 0.1	3.6 ± 0.3

a CC: the crystal conformation; C1: the first populated conformation from the MD simulation of CC; C2: the second populated conformation from the MD simulation of CC. See Table S2 for the initial compound reference numbers. VDW is van der Waals interaction, and ELE is electrostatic interaction.

b Based on the snapshots from the last 20 ns of simulation.

To determine any changes in the protein secondary structure, secondary structure element (SSE) plots were generated for each protein residue. The SSE plot summarizes the structure element distribution by residue position throughout the protein. The three categories are alpha-helices, beta-strands, and random coils. Alpha-helices are mainly made up of hydrophobic residues that are located in the core of the protein and are depicted by the orange sections. Beta-strands, however, contain both hydrophobic and polar amino acids, which are depicted by blue sections. The random coil is not one specific shape of a polymer conformation but a distribution of statistics of all chains depicted by the white spaces in the plots. SSE indicates that the helices were maintained during the simulation (Figure 3B).

RMSF was then calculated to determine fluctuations in each protein residue, intra- and extracellular loops, and N- and C-termini, which are usually the most flexible regions of the receptor. While some protein regions exhibited some increased RMSF spikes, the average RMSF was relatively small (<2 Å) and indicates low global fluctuations (Figure 3C). Finally, the 2D ligand interaction diagram was generated to determine the specific interactions between the crystal ligand and the protein active site, which revealed mostly hydrophobic contacts and some hydrogen bonding (Figure 3D,E). Overall, the system was shown to be stable and mimic the crystal structure with a stable binding pose.

### Crystal Complex Produces Other Conformations to Use for HTVS

**Figure 4:**
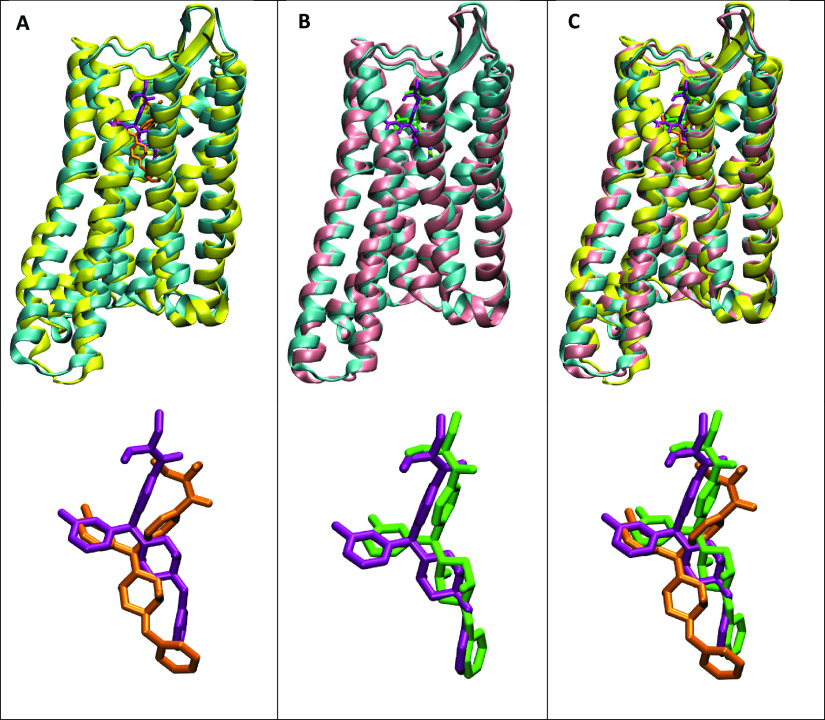
Superimposition of the active crystal DOR structure (PDB ID: 6PT3) (cyan) in complex with crystal agonist DPI-287 (purple) with the most abundant conformations from the MD simulations with agonist DPI-287. (A) DOR crystal conformation superimposed with the first abundant conformation (yellow, 75%) in complex with agonist DPI-287 (orange) including ligand-only view. (B) DOR crystal conformation superimposed with the second abundant conformation (pink, 24%) in complex with agonist DPI-287 (green) including ligand-only view. (C) DOR crystal conformation superimposed with both abundant clusters and all three ligand poses.

**Figure 5:**
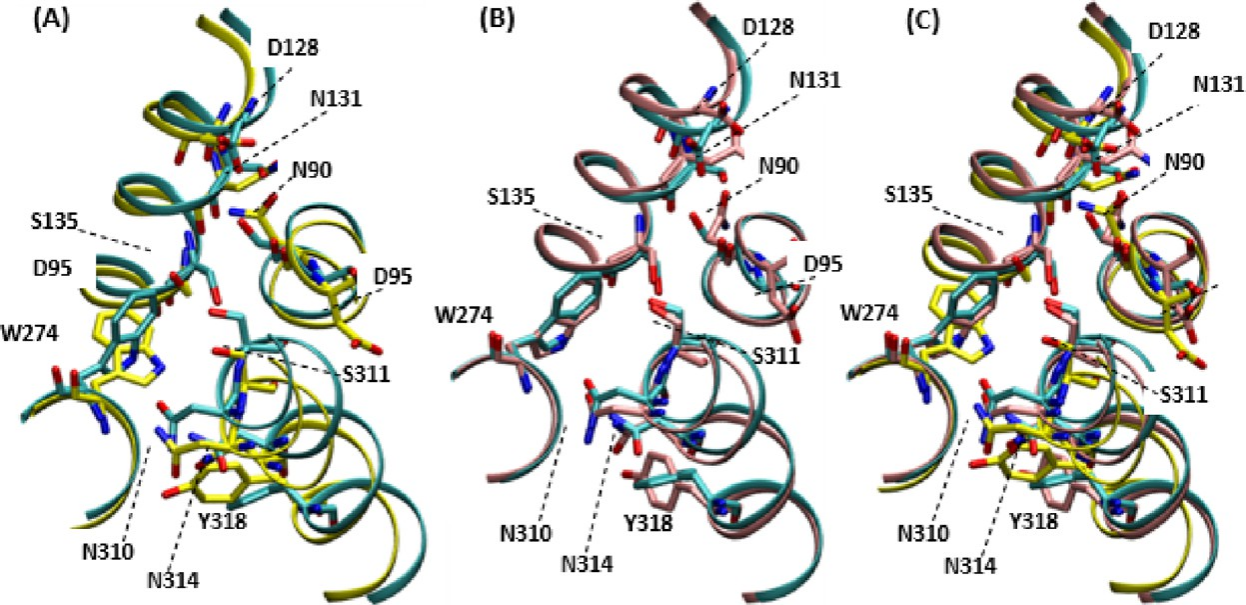
Predicted binding pocket of the different DOR conformations (yellow). Structural alignment of crystal conformation (cyan) with (A) representative conformation 1, (B) representative conformation 2, and (C) both conformations.

Clustering analysis was done after MD simulation to identify the populated conformations for the trajectory. Each cluster conformation contains a percentage of abundance based on the clustering algorithm in which a cutoff of 2% was used. From this, there were two abundant clusters (75 and 24%, respectively) produced from the crystal conformation simulation and were compared to the crystal complex (Figure 4). The two clusters slightly differ in conformation and the ligand binding pose from the crystal conformation. In a more precise view, the binding pocket of each structure was compared to the crystal pose (Figure 5). In this view, differences can be seen in the receptor itself and the side chains that have adopted different rotamer states. Specific residues where the side chains differ the most from the crystal in both clusters are N90, D95, D128, N131, N310, and Y318.

**Figure 6:**
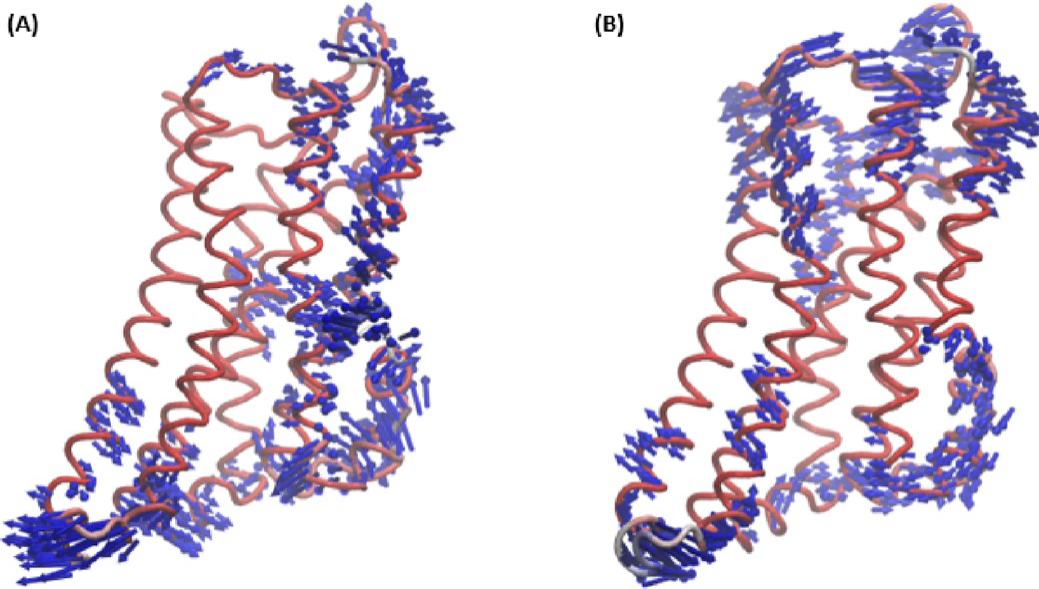
Top two low-frequency vibrational modes from the normal mode analysis based on the MD simulation of the crystal conformation of the active DOR in complex with agonist DPI-287. (A) Mode 1 and (B) mode 2. The top 10 modes can be found in Figure S1 of the supporting document.

Normal mode analysis on the crystal MD simulation trajectory revealed the top two low-frequency vibrational modes (Figure 6). This further validates the result of two cluster conformations that differ from the crystal conformation and therefore can be used as additional conformations to use for HTVS. Unweighted and weighted dynamic network models of the DPI-287/DOR system were calculated as described in the Methods section to decipher the allosteric signal transmission pathway. The unweighted network model shows that their connections are in good agreement with each other. Quantification of the correlation between the nodes in the weighted network model reveals the areas of the receptor that are in higher correlation to each other. The system appears to have higher correlations between edges TM5 and TM6.

**Figure 7:**
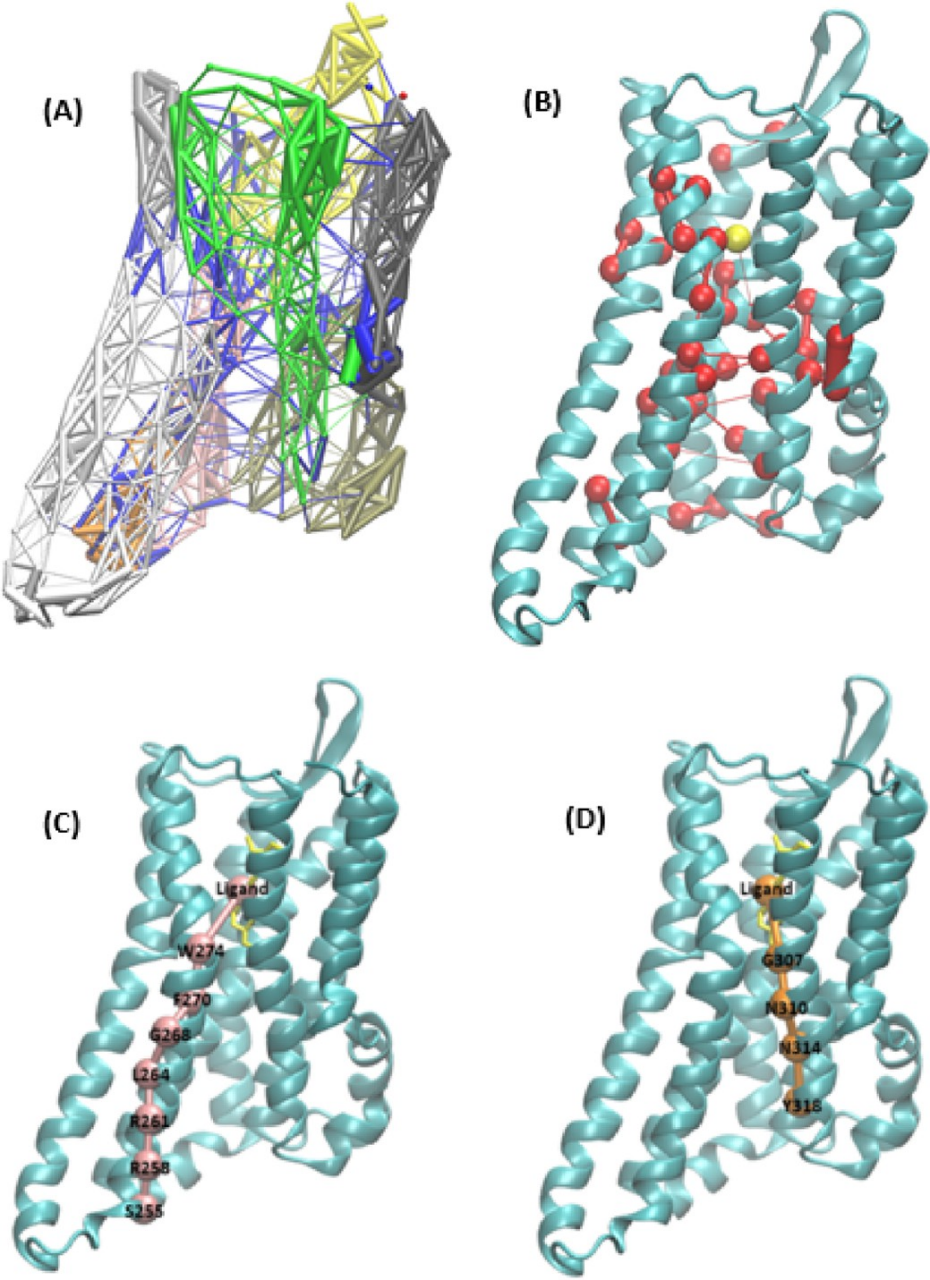
Network analysis. (A) Structural communities generated from the weighted dynamic network model separated by color. (B) The critical nodes and weighted edges (red) shown for the crystal DPI-287 system with the node for the ligand represented in yellow (top). (C, D) Optimal signal transduction pathway of the Transmission switch (pink nodes) and Toggle switch (orange nodes) starting from the ligand (bottom).

A community network model was generated using the weighted network model, which grouped residues together that interact more frequently and stronger than to residues in other communities (Figure 7). There were 38 critical nodes identified for the crystal system from the community analysis. These critical nodes were involved in signal transduction between different parts of the receptor throughout the simulation; therefore, the critical residue information was then cross referenced with experimentally reported mutagenesis data available on the G protein-coupled receptor databank (GPCRdb) to see if the residues were involved in the physical signal transduction. The DPI-287 system had nine critical residues that were also naturally occurring mutations (D95, Y129, M132, M142, R160, M186, A269, I282, N310). It also had three critical residues that were mutations in vitro (D95, W274, S312). Optimal paths generated for the DPI-287/DOR system give insight into the molecular signal transduction pathways involving the ligand. From the weighted network models, the shortest pathways able to pass a signal from the ligand to the site of the molecular switch (Tyrosine Toggle Switch: Y318) and the intracellular end of TM6 (Transmission Switch: S255) were calculated as the optimal paths. DPI-287 has a direct optimal path for the Transmission Switch (CWXP) through TM6 and another direct path for the Tyrosine Toggle Switch (NPXXY) through TM7.

### Top Eight ZINC Compounds Identified from MD Simulations, MM-GBSA, and SwissADME Property Prediction

HTVS was ran on all three conformations of the active DOR (crystal conformation, cluster 1, cluster 2) for FDA-approved 2466 drugs^32^ and compounds from the ZINC15 database, as described in the Methods section. Using the docking score of the crystal ligand DPI-287 as a cutoff for favorable binding (−8.6 kcal/mol), the number of stars (0–1 stars) for drug-likeness, and the cluster ID in picking diverse chemical scaffolds, a total of 69 ZINC compounds were chosen. The top 10 FDA-approved drugs were also identified from their docking scores. However, comparing the docking scores revealed that the top 69 ZINC compounds had significantly better docking scores than the FDA-approved drugs, therefore ruling out the further investigation of the FDA-approved drugs (Figure S2, Table 2, and Table S2). From the 69 ZINC compounds, 32 were docked to the crystal receptor (Figure S3), 11 were targeted to the first abundant cluster receptor (Figure S4), and 26 were targeted to the second abundant cluster receptor (Figure S5). Each docked ZINC compound showed good binding pose agreement with the crystal ligand (Figures S6–S8). Each of the 69 ZINC compound–protein complexes then underwent 200 ns MD simulations and underwent post-MD simulation analyses, including RMSD convergence (Figure S9), comparison between Glide XP dock and MD simulation binding poses (Figures S10–S12), protein–ligand contacts (Figures S13–S16), SSE (Figures S17–S20), RMSF (Figure S21), and SwissADME property prediction (Figure S22).

MM-GBSA was calculated for each of the 69 ZINC compounds to their respective receptor conformation, including the crystal ligand DPI-287, to determine the binding affinity for the receptor, with more negative values indicating better binding (Table S2). DPI-287 had an MM-GBSA of −90.2 kcal/mol, which was used as the cutoff and reference. From this, the top 69 ZINC compounds were filtered down to 22 compounds, based on their favorable MM-GBSA binding energies (−91.5 ± 7.8 to −134.9 ± 11.3 kcal/mol).

To further filter down the top ZINC compounds, SwissADME properties were then calculated for the top 69 ZINC compounds (Table 3 and Table S3). SwissADME properties include but are not limited to GI absorption and BBB permeability, which were the major determinates. Also, similar to the crystal ligand, having no alerts (PAINS, BRENKS) was attractive as well due to having a low chance of false positives from occurring. Considering these desired properties, eight ZINC compounds were chosen as the top compounds (Tables 2 and 3), which also exhibit more favorable MM-GBSA binding free energies (Table 2). Two of the ZINC compounds were targeting the crystal conformation, four were targeting the first abundant cluster, and two were targeting the second abundant cluster. From here on, the top eight ZINC compounds are the focus of additional analysis.

**Table 3:** The Predicted Pharmacokinetics ADME Properties for Top Eight ZINC Compounds by SwissSimilarity Server^a^

compound	GI absorption	BBB permeant	CYP1A2	CYP2C19	CYP2C9	CYP2D6	CYP3A4	Lipinski rule	PAINS	Brenk
crystal structure (PDB ID: 6PT3)	high	yes	no	no	no	yes	yes	yes; 0 violation	0 alert	0 alert
ZINC000020559278	high	yes	yes	no	no	yes	yes	yes; 0 violation	0 alert	0 alert
ZINC000078515864	high	yes	no	no	no	yes	no	yes; 0 violation	0 alert	0 alert
ZINC000025329384	high	yes	no	no	no	yes	yes	yes; 0 violation	0 alert	0 alert
ZINC000037556415	high	yes	no	yes	yes	yes	yes	yes; 0 violation	0 alert	0 alert
ZINC000827360794	high	yes	no	no	no	yes	yes	yes; 0 violation	0 alert	0 alert
ZINC000078648574	high	yes	no	yes	yes	yes	no	yes; 0 violation	0 alert	0 alert
ZINC000057999653	high	yes	yes	yes	yes	yes	yes	yes; 0 violation	0 alert	0 alert
ZINC000006664413	high	yes	yes	yes	yes	yes	yes	yes; 0 violation	0 alert	0 alert

a Note: GI is gastrointestinal, and BBB is the blood–brain barrier.

### Top Eight ZINC Compounds Assume Steady-State Equilibrium

**Figure 8:**
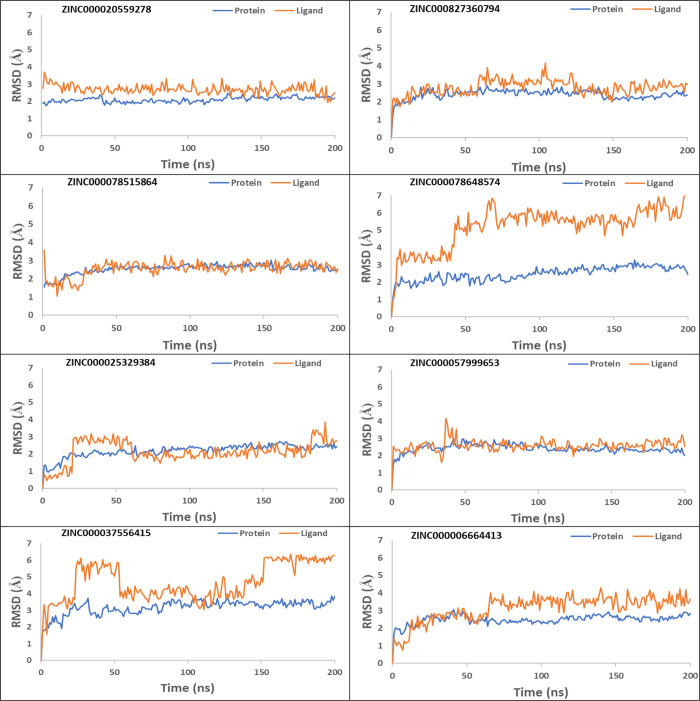
Cα RMSD of the top eight ZINC compounds during 200 ns MD simulation in reference to the crystal active DOR conformation (PDB ID: 6PT3).

Protein and ligand RMSDs were calculated over each trajectory for the top eight ZINC compounds to check for convergence (Figure 8). RMSD plots for the remaining 61 ZINC compounds are in the supporting document (Figure S9). All eight MD simulations achieved steady-state equilibrium during the last 50 ns of simulation time, with the lower protein RMSD values indicating they remained stable throughout the simulation. Ligand RMSD indicated fluctuations in three ZINC compounds (ZINC000025329384, ZINC000037556415, and ZINC000078648574), whereas the remaining five compounds remained more stable throughout the simulation.

### MD Simulations Improved the Binding Pose of the Top Eight ZINC Compounds

**Figure 9:**
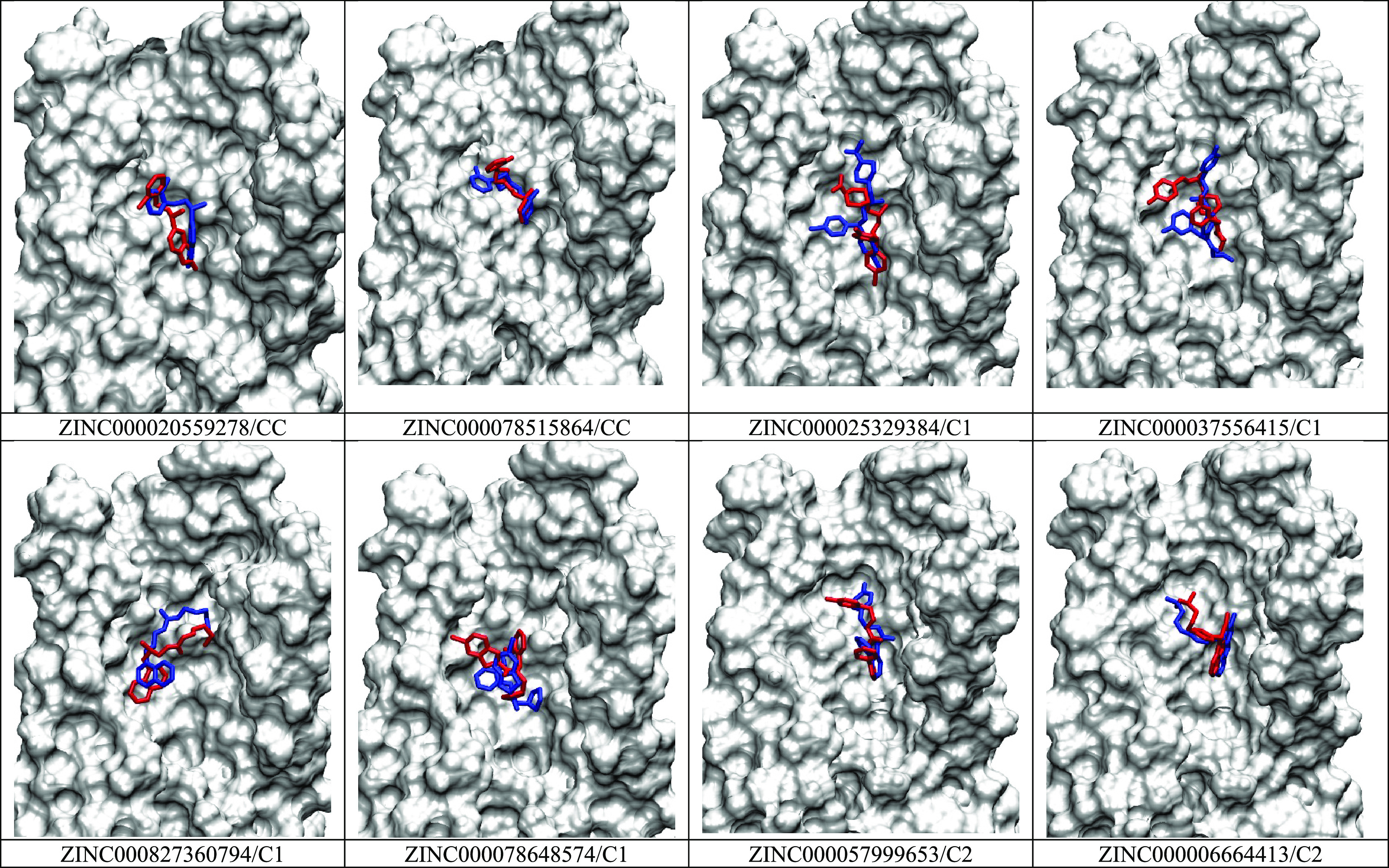
Comparison of Glide XP docking pose (blue) and MD simulation pose (red) for the top eight ZINC compounds. Receptor is in surface representation (gray).

Comparison between the Glide XP docking pose and the MD simulation pose was done for the top eight ZINC compounds (Figure 9). The same comparison was made for the remaining 61 ZINC compounds in the supporting document (Figures S10–S12). The simulation can significantly alter the ligand original bound conformation to optimize their interactions with the receptor. The simulation thus improved the binding pose of each of the top compounds. This corresponds with the MM-GBSA results (Table 1) that were used to estimate the binding free energy of the compounds where the binding interaction between the protein and ligand complexes is specified by the free energy binding. The crystal ligand was used as a control where its score was −90.2 kcal/mol. The top compounds picked had significantly higher binding energy to the DOR, with the lowest of the scores being −134.9 ± 11.3 kcal/mol.

### Protein–Ligand Interactions of the Top Eight ZINC Compounds to the DOR

**Figure 10:**
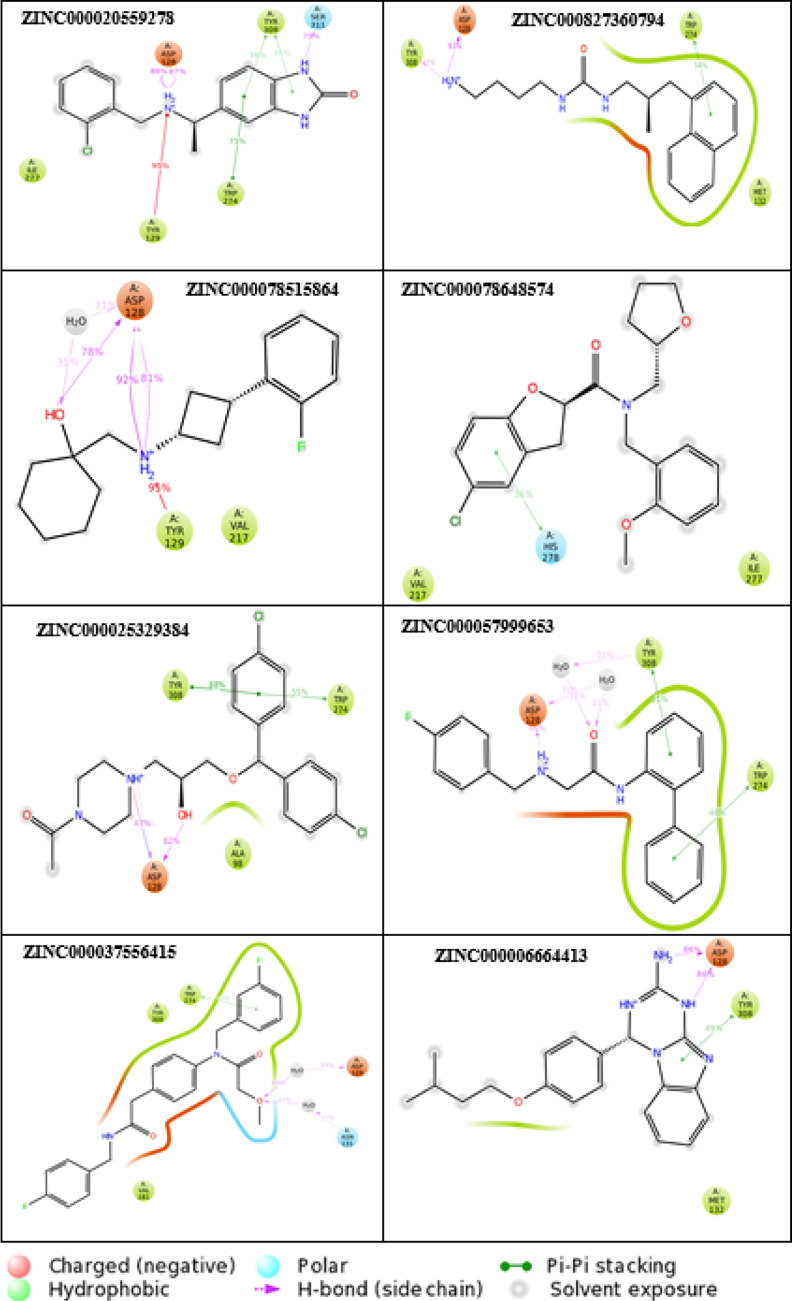
2D ligand interaction diagrams for the top eight ZINC compounds with the crystal DOR receptor binding site.

The residues involved in the top eight ZINC compounds binding to the protein receptor were analyzed as described in the Methods section with the Desmond SID. All interacting residues to the top eight ZINC compounds and crystal ligand were tabulated (Table 4). 2D ligand interaction diagrams were generated for the top eight ZINC compounds (Figure 10). Protein–ligand contact histograms were generated for all the top 69 ZINC compounds (Figures S13–S16). The highest amount of hydrogen bonding was observed in compounds ZINC000078515864, ZINC000006664413, and ZINC000020559278. ASP128 is the main residue involved in hydrogen bonding in seven out of the eight ZINC compounds and is also maintained in the crystal ligand (Figure 10). ZINC000037556415, ZINC000827360794, ZINC000078648574, ZINC000057999653, and ZINC000006664413 exhibited the highest number of hydrophobic contacts, where the interaction of such generally involves hydrophobic amino acids and an aromatic or aliphatic group on the ligand. Ionic interactions were mainly observed in ZINC000025329384 and ZINC000057999653. Water bridges occurred in all eight ZINC compounds except for ZINC000078648574, which is the same compound that did not show hydrogen bonding to residue ASP128. Most of the compounds showed higher hydrophobic interactions and hydrogen bonding, in comparison to the crystal structure, leading to higher GI absorption.

**Table 4:** Protein-Ligand Interactions during MD Simulations for the Top Eight ZINC Compounds from the MD Simulations^a^

Ref Comp	ZINC20559278	ZINC78515864	ZINC25329384	ZINC37556415	ZINC827360794	ZINC78648574	ZINC57999653	ZINC6664413
ILE 52								
TYR 56							TYR 56	TYR 56
				ALA 94				
	ASP 95							
ALA 98	ALA 98		ALA 98	ALA 98			ALA 98	ALA 98
					THR 101			THR 101
					LEU 102			
GLN 105		GLN 105	GLN 105	GLN 105	GLN 105		GLN 105	GLN 105
LYS 108				LYS 108	LYS 108		LYS 108	LYS 108
TYR 109								
					VAL 124		VAL 124	VAL 124
				LEU 125	LEU 125			LEU 125
	ILE 127			ILE 127				
ASP 128	ASP 128	ASP 128	ASP 128	ASP 128	ASP 128		ASP 128	ASP 128
TYR 129	TYR 129	TYR 129	TYR 129	TYR 129	TYR 129	TYR 129	TYR 129	
ASN 131	ASN 131		ASN 131	ASN 131	ASN 131			
MET 132	MET 132	MET 132	MET 132	MET 132	MET 132	MET 132	MET 132	MET 132
	SER 135					SER 135		
					ILE 136	ILE 136		
ASP 210								
THR 213				THR 213				
LYS 214	LYS 214	LYS 214		LYS 214	LYS 214	LYS 214		
VAL 217	VAL 217	VAL 217	VAL 217	VAL 217	VAL 217	VAL 217	VAL 217	VAL 217
	PHE 218							
					ALA 221	ALA 221		
					PHE 270			
TRP 274	TRP 274	TRP 274	TRP 274	TRP 274	TRP 274	TRP 274	TRP 274	TRP 274
ILE 277	ILE 277	ILE 277	ILE 277	ILE 277	ILE 277	ILE 277	ILE 277	ILE 277
HIS 278	HIS 278	HIS 278		HIS 278	HIS 278	HIS 278	HIS 278	
PHE 280		PHE 280						PHE 280
VAL 281	VAL 281	VAL 281		VAL 281			VAL 281	VAL 281
				ILE 282				
TRP 284		TRP 284		TRP 284				TRP 284
ARG 291								
LEU 300		LEU 300		LEU 300		LEU 300		LEU 300
HIS 301				HIS 301			HIS 301	
								CYS 303
ILE 304	ILE 304	ILE 304	ILE 304	ILE 304	ILE 304	ILE 304	ILE 304	ILE 304
	GLY 307			GLY 307				
TYR 308	TYR 308	TYR 308	TYR 308	TYR 308	TYR 308	TYR 308	TYR 308	TYR 308
	SER 311			SER 311	SER 311			

a Reference compound is the crystal ligand from PDB ID: 6PT3.

### Secondary Structure Maintained for the Top Eight ZINC Compound–Protein Complexes

As was done for the crystal DOR system, protein SSE plots were generated for the top 69 ZINC compound–protein complexes (Figures S17–S20). Only minor changes in alpha-helical content were observed in each of these complexes, indicating that the protein secondary structure was maintained.

### RMSF Shows Fluctuation in Regions of the Protein with Respect to the Ligand

Protein RMSF was calculated for the top eight ZINC compounds (Figure S21). Higher values depicted by peaks are areas of the protein that fluctuate the most, such as the N- and C-terminals as well as the intra- and extracellular loops. ZINC000037556415 showed the greatest fluctuation at nearly 5 Å around residue 200, which is located in extracellular loop 2. All compounds showed similar fluctuation at the same residue positions between 1 and 3 Å. A small fluctuation from ZINC000078648574 and ZINC000020559278 was observed around residue 250. Using the crystal structure as a positive control, each complex showed the same or higher residual fluctuation throughout the simulations.

### Network Analysis Revealed Communication among Different Regions of the DOR

**Figure 11:**
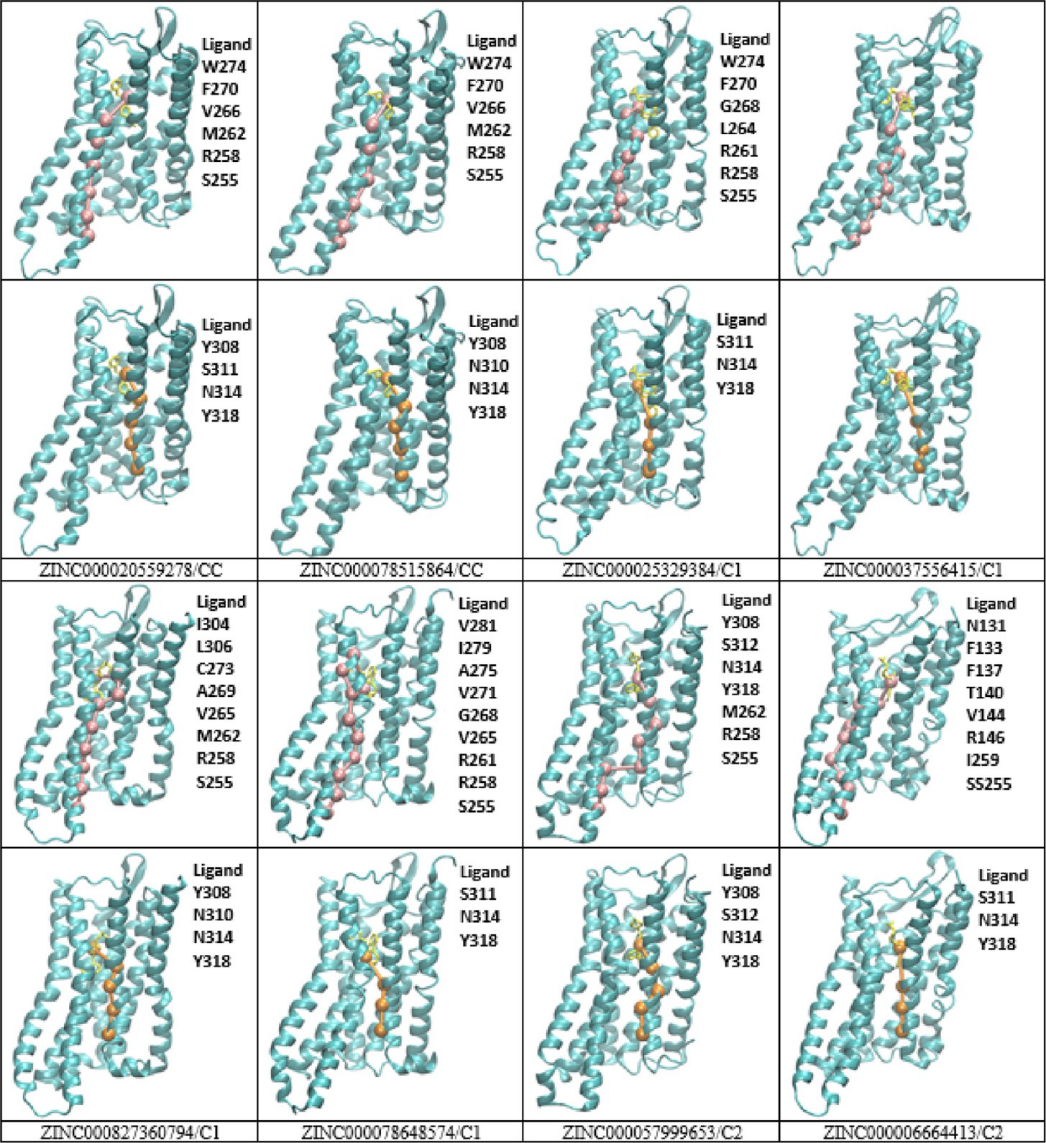
Optimal signal transduction pathway of the Transmission Switch (pink nodes) and the Toggle Switch (orange nodes) starting from the ligand of each of the top eight ZINC compounds.

Unweighted and weighted network models of the DPI-287/DOR system and the top eight ZINC compound systems were calculated, as described in the Methods section, to decipher the allosteric signal transmission pathway. The comparison of the unweighted network models between the systems shows that their connections are in good agreement with each other. Quantifying the correlation between the nodes in the weighted network model reveals similarities observed between the systems. All systems appear to have higher correlations between edges TM5 and TM6. Community network models were generated using the weighted network model, which grouped residues together that interact more frequently and more strongly than to residues in other communities. The systems that seem to communicate more similarly to the crystal complex are ZINC000020559278, ZINC000078515864, ZINC000827360794, ZINC000078648574, ZINC000057999653, and ZINC000006664413. The basis for the similarity is that the intracellular portions of TM5 and TM6 are in the same community represented by one single color. Another observation in most of the similar systems is that the extracellular portions of TM6 and TM7 belong to the same community while the same region in TM5 belongs to a different community. This trend occurred in ZINC000078515864, ZINC000827360794, and ZINC000078648574. The systems that had the most similarities in critical nodes with the crystal system are ZINC000020559278 with 11 of the same critical nodes, ZINC000078515864 with 13, and ZINC000078648574 with 11 (Table S4). When calculating the optimal paths of the Transmission and Toggle Switch for each of the top eight ZINC compounds, multiple systems showed great similarities to the crystal complex (Figure 11 and Table S5). ZINC000025329384 shared every residue as the crystal ligand with the Transmission Switch pathway and two out of the four residues of the Toggle Switch pathway. ZINC000020559278, ZINC000078515864, and ZINC000037556415 also had many similar residues to the crystal complex with the Transmission Switch and Toggle Switch. ZINC000827360794 had the most residues similar to the crystal with the Toggle Switch.

### Selectivity Analysis on DOR, KOR, and MOR

To determine the selectivity of the top eight ZINC compounds for the DOR, the Glide XP docking scores of the top eight ZINC compounds on the crystal conformation (CC) of DOR, KOR, and MOR were obtained using the docking protocol in our original VSW (Table 5, columns 4–6). The original docking scores (Table 5, column 3) on DOR with the specific conformation (CC, C1/the first populated MD conformation, C2/the second populated conformation) were compared these docking scores on DOR, KOR, and MOR with CC. Two notable features are identified: (1) The docking score on DOR with specific conformation (C1 or C2) of the same compounds (3–8) is on average better than the docking on DOR with CC by −1.8 kcal/mol, supporting our virtual screening strategy using multiple conformations rather than just crystal conformation to enrich the top eight ZINC compounds. (2) The docking score on DOR with specific conformation (CC, C1, and C2) is on average better than the docking score on KOR and MOR with CC by −1.3 and −2.4 kcal/mol, respectively. This clearly shows the good selectivity of our top eight ZINC compounds to DOR rather than KOR and MOR, if the binding entropy is comparable for these complexes. Only in two cases (ZINC000078515864 and ZINC000827360794) are the docking scores on DOR slightly worse than those on KOR by 0.7 and 1.7 kcal/mol. To support the validity of our selectivity analysis, we docked the crystal ligands of DOR (DPI-287), KOR (MP1104), and MOR (BU27) to each of the opioid receptors. These ligands should have the highest selectivity (lowest docking score) for their receptor. Indeed, this was the case. DPI-287 showed quite favorable selectivity to the DOR (XP = −8.6 kcal/mol, ΔXP score = 0) compared to KOR (XP = −5.4 kcal/mol, ΔXP score = 3.2) and MOR (XP = −3.9 kcal/mol, ΔXP score = 4.7). MP1104 selectivity was greatest for KOR (XP = −8.8 kcal/mol, ΔXP score = 0) compared to DOR (XP = −6.9 kcal/mol, ΔXP score = 1.9) and MOR (XP = −6.5 kcal/mol, ΔXP score = 2.3). Last, BU27 selectivity was highest for MOR (XP = −6.6 kcal/mol, ΔXP score = 0) compared to DOR (XP = −5.1 kcal/mol, ΔXP score = 1.5) and KOR (XP = −5.9 kcal/mol, ΔXP score = 0.7). Further experiments are required to validate the selectivity of these ligands toward DOR.

**Table 5:** Glide XP Docking Scores of the Crystal Ligands DPI-287, MP1104, and BU27 and the Top Eight ZINC Compounds on the DOR, KOR, and MOR^a^

compound ID	ref no.	original docking score on DOR (kcal/mol)	docking score on DOR (6 pt3) (kcal/mol)	docking score on KOR (6b73) (kcal/mol)	docking score on MOR (5c1m) (kcal/mol)
DPI-287 (DOR crystal ligand)	DPI-287	N/A	–8.6	–5.4	–3.9
comparison of docking scores		N/A	0	3.2	4.7
MP1104 (KOR crystal ligand)	MP1104	N/A	–6.9	–8.8	**–**6.5
comparison of docking scores			1.9	0	2.3
BU27 (MOR crystal ligand)	BU27	N/A	–5.1	–5.9	–6.6
comparison of docking scores			1.5	0.7	0
ZINC000020559278	(1)	–9.9(CC)	–9.9 (CC)	–9.2 (CC)	–7.1 (CC)
comparison of docking scores		0	0	0.7	2.8
ZINC000078515864	(17)	–8.9(CC)	–8.9 (CC)	–9.6 (CC)	–6.7 (CC)
comparison of docking scores		0	0	–0.7	2.2
ZINC000025329384	(1)	–10.2 (C1)	–7.7 (CC)	–7.9 (CC)	–7.7 (CC)
comparison of docking scores		0	2.5	2.3	2.5
ZINC000037556415	(2)	–9.4 (C1)	–7.6 (CC)	–8.4 (CC)	–7.6 (CC)
comparison of docking scores		0	1.8	1.0	1.8
ZINC000827360794	(6)	–9.1 (C1)	–8.2 (CC)	–10.8 (CC)	–8.2 (CC)
comparison of docking scores		0	0.9	–1.7	0.9
ZINC000078648574	(9)	–9.0 (C1)	–6.5 (CC)	–8.7 (CC)	–6.5 (CC)
comparison of docking scores		0	2.5	0.3	2.5
ZINC000057999653	(1)	–10.1 (C2)	–7.7 (CC)	–8.9 (CC)	–7.7 (CC)
comparison of docking scores		0	2.4	1.2	2.4
ZINC000006664413	(5)	–9.8 (C2)	–5.5 (CC)	–2.7 (CC)	–5.5 (CC)
comparison of docking scores		0	4.3	7.1	4.3
average docking scores		–9.6	–7.8	–8.3	–7.2
		0	1.8	1.3	2.4

a The original docking scores of DOR were compared to the receptors based on their crystal conformations, respectively. The total average docking scores of DOR, KOR, and MOR for the top eight ZINC compounds are shown as well. The total average docking scores of each receptor were calculated based on the positive values. CC: the crystal conformation; C1: the first populated conformation from the MD simulation of CC; C2: the second populated conformation from the MD simulation of CC. See Table S2 for the initial compound reference number.

## Discussion

The opioid epidemic has brought to light the need for better opioid alternatives in public health all around the world. The continued rise of opioid addiction and overdose will not stop until there are better therapeutic agents available. Researchers and scientists have discovered that the DOR shows potential in not only pain management but also neurological and psychiatric disorders. Agonists targeting the DOR are strongly believed to not display addictive or dependence properties, such as MOR agonists, having the potential to help combat the addictive opioid crisis today.

Although previous studies have been done on the DOR, none to our best knowledge have ever utilized multiple conformations from MD simulations for HTVS. MD simulations are able to probe deeper into interactions and dynamics that happen in a system that cannot be obtained from a crystal structure alone. Sampling the conformations from the active state DOR and using them for HTVS offers an opportunity to find better potential agonists to be of therapeutic use. Running long MD simulations on each of the top ZINC compounds identified from our VSW allows us to determine if the compounds bound to the DOR will remain stable and their protein–ligand interactions. Here, we present the first study using the ensemble-based HTVS approach to discover potential agonists to target the DOR. Our VSW combining molecular docking, MD simulation, bioinformatics tools, and drug similarity search revealed the top 69 hits from the ZINC15 database (32 from crystal conformation, 11 from cluster conformation 1, and 26 from cluster conformation 2). MM-GBSA and SwissADME prediction analyses reduced the top 69 ZINC compounds to eight compounds. The predicted drug ADME properties helped to specify if the compounds were highly (GI) absorbent as well as BBB permeant. These were the properties most valued due to opioids coming in an oral form and the receptors being located in areas of the brain. Without these specific properties, the compounds could be ruled out.

The protein–ligand interaction further validated the choice of the top eight ZINC compounds as potential DOR agonists. The large dataset and extended HTVS method portray the best interactions between ligands to form a complex with a molecular target. The adverse effects and related articles of the selected compounds were checked through CAS SciFinder and PubChem, where the compounds showed no adverse effects. Out of the top eight ZINC compounds, ZINC000057999653 is patented to be useful for altering the lifespan of eukaryotic organisms. These results further validate the top hits to be potential agonists.

The use of dynamic network models based on MD simulations data has shown to be efficient in extracting correlated motions, allosteric signals, and signal transduction networks within complex systems. The correlated motions are thought to be linked to their activity, which is normally difficult to accurately distinguish through visualization of the MD simulations alone. In addition, the communities that are generated with the dynamic network are highly correlated and provide insight into the overall communication network from ligand binding.^71−73^ In our study, the dynamic network analysis aided in identifying similar communication systems between the crystal DPI-287 and top eight ZINC compound systems. These similarities became more apparent when comparing the weighted networks that base their correlated motion in the simulation trajectories to the connections between nodes in the networks. The community models show that compound systems of ZINC000078515864, ZINC000827360794, and ZINC000078648574 communicate the most similarly to the DPI-287 system. This analysis highlights how the structural differences of the ligands can have similar or different dynamics to the DOR due to the grouping of communities based on residues that interact strongly and frequently with one another.

Based on the results, the potential binding and agonistic effects of the top eight ZINC compounds are indicated. Ensemble-based structure HTVS is a useful approach to find potential molecules that could target the binding pocket of the DOR. After examining 17 million ZINC15 compounds using structure-based HTVS methods, the most potential hits were further examined by MD simulations followed by MMGBSA binding free energy analysis. Further experiments are required to validate these compounds as potent DOR agonists. Nonetheless, this study has the potential to assist in the efforts to aid in the opioid epidemic. Experimental studies can be conducted on these compounds to help in the efforts of opioid addiction.

## Conclusions

The lack of opioid alternatives with non-addictive properties has prompted researchers to look for effective candidates on all opioid receptors (mu, kappa, delta), with delta showing promising effects. Computational studies are a cost-effective method to identify a new target of existing drugs. Since the DOR is an attractive target for therapeutic effects without significant adverse effects, we have exploited the conformational flexibility of the DOR to search novel ZINC15 compounds, which may aid in opioid addiction. 1 μs MD simulation of the active crystal conformation of the DOR was used to generate the structure ensemble. Using the clustering method, two major conformations of the DOR were identified. A total of three conformations (crystal conformation and two MD generated conformations) were used in our VSW of zinc compounds (17 million), leading to 69 compounds with top Glide XP docking scores and diverse structures. To further validate these compounds, 200 ns MD simulations were carried out to check the stability of the docked complexes, and the predicted drug ADME properties were examined. Eight stable systems were identified using a combination of dynamic properties (RMSD, RMSF) and MM-GBSA binding free energy calculation. Each of the eight top compounds exhibited better binding energy than the crystal ligand and contained attractive drug properties. Although this study suggests these top eight compounds may serve as good drug candidates for the DOR, further experimental studies and risk–benefit assessment are needed to evaluate the therapeutic values of the mentioned novel compounds. This study shows flexibility modeling the DOR; using MD simulations is a powerful tool in identifying novel compounds that could potentially show no adverse effects and aid in the opioid epidemic.

## Supporting Information Available

(Table S1) Molecular properties calculated using QikProp; (Table S2) detailed information regarding various properties from the Glide XP docking and MD simulations of the top 32 ZINC compounds in reference to the crystal structure (CC), the top 11 ZINC compounds in reference to the first conformation from the crystal MD simulation (C1), and the top 26 ZINC compounds in reference to the second conformation from the crystal MD simulation (C2); (Table S3) the predicted ADME properties for the top 32 best compounds in reference to the crystal conformation (PDB ID: 6PT3), the top 11 compounds in reference to the first conformation from the crystal MD simulation (C1), and the top 26 compounds in reference to the second conformation from the crystal MD simulation (C2), including the crystal reference compound from the SwissADME server.; (Table S4) critical nodes identified from the Network Analysis for the DPI-287/DOR system and the top eight compound systems; (Table S5) the optimal path of the transmission and tyrosine toggle switch generated from the Network Analysis for the DPI-287/DOR system crystal complex and the top eight ZINC compounds; (Figure S1) top 10 low vibrational modes from the normal mode analysis (PCA) based on the active conformation DOR agonist DPI-287 system; (Figure S2) top 10 FDA-approved drug compounds based on their docking scores for the crystal conformation of the DOR; (Figure S3) top 32 ZINC compounds for the DOR crystal conformation including the crystal reference compound with ZINC ID, structure, SMILE code, docking score, number of STARs (indicator for “drug-likeness”), cluster IDs (ligand similarity clustering based on Canvas), and centroid (measures distances to the arithmetic means of clusters; (Figure S4) top 11 ZINC compounds for the first representative conformation of the DOR MD simulation structure conformation including the crystal reference compound with ZINC ID, structure, SMILE code, docking score, number of STARs (indicator for “drug-likeness”), cluster IDs (ligand similarity clustering based on Canvas), and centroid (measures distances to the arithmetic means of clusters); (Figure S5) top 26 ZINC compounds for the second representative conformation of the DOR MD simulation structure conformation including the crystal reference compound with ZINC ID, structure, SMILE code, docking score, number of STARs (indicator for “drug-likeness”), cluster IDs (ligand similarity clustering based on Canvas), and centroid (measures distances to the arithmetic means of clusters); (Figure S6) comparison between the DOR crystal structure (PDB ID: 6PT3) (cyan) and the docked complex of the top 32 ZINC compounds (gray) in the side view and ligand view with the 2D chemical structure of the ZINC compounds; (Figure S7) comparison between the DOR crystal structure (PDB ID: 6PT3) (cyan) and the docked complex of the top 11 ZINC compounds (gray) of the first representative structure in the side view and ligand view with the 2D chemical structure of the ZINC compounds; (Figure S8) comparison between the DOR crystal structure (PDB ID: 6PT3) (cyan) and the docked complex of the top 26 ZINC compounds (gray) of the second representative structure in the side view and ligand view with the 2D chemical structure of the ZINC compounds; (Figure S9) Cα RMSD of the top ZINC compounds during 200 ns MD simulation in reference to the crystal active DOR conformation (PDB ID: 6PT3); (Figure S10) comparison of the top 32 ZINC compounds for the crystal conformation (PDB ID: 6PT3) in the docked pose (blue) and the MD simulation pose (red) with the DOR in surface representation (gray); (Figure S11) comparison of the top 11 ZINC compounds for the first representative structure from the MD crystal conformation (PDB ID: 6PT3) in the docked pose (blue) and the MD simulation pose (red) with the DOR in surface representation (gray); (Figure S12) comparison of the top 26 ZINC compounds for the second representative structure from the MD crystal conformation (PDB ID: 6PT3) in the docked pose (blue) and the MD simulation pose (red) with the DOR in surface representation (gray); (Figure S13) protein–ligand contacts during MD simulations for the top eight ZINC compounds; (Figure S14) protein–ligand contacts during MD simulations for top 32 ZINC compounds for the crystal conformation (PDB ID: 6PT3); (Figure S15) protein–ligand contacts during MD simulations for top 11 ZINC compounds for the first representative structure from the MD of the crystal conformation (PDB ID: 6PT3); (Figure S16) protein–ligand contacts during MD simulations for top 26 ZINC compounds for the second representative structure from the MD of the crystal conformation (PDB ID: 6PT3); (Figure S17) protein secondary structure elements (SSE) of the receptor in complex with the top eight ZINC compounds; (Figure S18) protein secondary structure elements for the top 32 ZINC compounds for the crystal conformation (PDB ID: 6PT3); (Figure S19) protein secondary structure elements for the top 11 ZINC compounds for the first representative structure from the crystal conformation (PDB ID: 6PT3); (Figure S20) protein secondary structure elements for the top 26 ZINC compounds for the second representative structure from the crystal conformation (PDB ID: 6PT3); (Figure S21) the Cα root mean square fluctuation (RMSF) of the receptor in complex with the top eight ZINC compounds; (Figure S22) the predicted ADME properties for the top 32 ZINC compounds based on the crystal conformation (PDB ID: 6PT3) including the reference compound from the SwissADME server (PDF)
