# Supplementary material for: Probing the Activation Mechanisms of Agonist DPI-287 to Delta-Opioid Receptor and Novel Agonists Using Ensemble-Based Virtual Screening with Molecular Dynamics Simulations
Source: ACS Omega. 2023 Aug 31;8(36):32404–23. doi: 10.1021/acsomega.3c01918 (PMC10500586; doi:10.1021/acsomega.3c01918)
Supplement: Supplementary file 1 — ao3c01918_si_001.pdf [file ao3c01918_si_001.pdf]

To Probe Activation mechanism of agonist DPI-287 to Delta Opioid Receptor and Novel Agonists to using Ensemble-based Virtual Screening with Molecular Dynamics Simulations

Emily Dean, AnneMarie Dominique, Americus Palillero, Annie Tran, Nicholas Paradis, and Chun Wu*

Department of Molecular & Cellular Biosciences, College of Science and Mathematics, Rowan University, Glassboro, New Jersey 08028, United States.

*To whom correspondence should be addressed: wuc@rowan.edu

Table S1. Molecular properties calculated using Qikprop.

No.	Property of Descriptors	Description[1]
1	**Dipole**	Dipole moment
2	**IP**	Ionization potential (negative HOMO energy) using Parametric method 3 (PM3)
3	**EA**	Electron affinity (negative HOMO energy) using Parametric method 3 (PM3)
4	**SASA**	Total solvent accessible surface area (SASA, probe radius of 1.4 Å)
5	**FOSA**	SASA hydrophobic component (attached H, saturated C atoms)
6	**FISA**	SASA hydrophilic component (N, O, H of the heteroatoms, carbonyl C)
7	**PISA**	SASA π component (attached C and H atoms)
8	**WPSA**	Weak polar component of SASA (halogens, P, and S atoms)
9	**donorHB**	Number of hydrogen bonds *donated* by the solute to water molecules in aqueous solution
10	**accptHB**	Number of hydrogen bonds *accepted* by the solute to water molecules in aqueous solution
11	**QPlogPC16**	Predicted hexadecane/gas partition coefficient
12	**QPlogPoct**	Predicted octanol/gas partition coefficient
13	**QPlogPw**	Predicted water/gas partition coefficient
14	**QPlogPo/w**	Predicted octanol/water partition coefficient
15	**QPlogBB**	Predicted brain/blood partition coefficient
16	**PSA**	Predicted van der Waals surface area (polar N, O and carbonyl C atoms)
17	**volume**	Total solvent-accessible volume (in $Å^3$, probe radius of 1.4 Å)
18	**MW**	Molecular weight
19	**#rotor**	Number of rotatable bonds (non-trivial (CX3) and non-hindered (not alkene, amide, and small ring).
20	**glob**	Globularity descriptor using the expression ($4\pi r^{2)/ (SASA)}$). r is the radius of a sphere and v is equivalent to the molecular volume
21	**QPpolrz**	Predicted polarizability ($Å^3$)
22	**logS**	Predicts key solubility properties
23	**QPLogKhsa**	Predicts binding of molecule to human serum albumin
24	**#metabol**	Predicted number of likely metabolic reactions

[1] : Molecular property descriptions are from the Qikprop User Manual 4.4.
Note: Å = Angstroms, N, C, O = Nitrogen, carbon and oxygen atoms, respectively.

Table S2. Detailed information regarding various properties from the Glide XP docking and MD simulations of the top 32 ZINC compounds in reference to the crystal structure (CC), the top 11 ZINC compounds in reference to the first conformation from the crystal MD simulation (C1), and the top 26 ZINC compounds in reference to the second conformation from the crystal MD simulation (C2). The top 22 ZINC compounds are in bold.

Structure	#	ZINC ID	Docking Score (kcal/mol)	VDW (kcal/mol)	ELE (kcal/mol)	Hydrophobic (kcal/mol)	MM-GBSA (kcal/mol)	Receptor RMSD[1] (Å)	Ligand RMSD[1] (Å)
	Ref.	*PDB ID: 6PT3*	-8.6	-57.0±2.8	18.5±3.7	-51.7±3.5	-90.2±6.3	2.9±0.1	2.5±0.1
**CC**	**1**	**ZINC000020559278**	**-9.9**	**-44.7±2.8**	**-31.3±3.7**	**-40.8±2.3**	**-116.9±5.0**	**2.2±0.1**	**2.5±0.3**
CC	2	ZINC000014242201	-9.9	-53.3±3.6	-25.5±7.6	-32.9±3.1	-111.7±9.6	3.9±0.2	6.8±0.5
CC	3	ZINC000562639987	-9.6	-40.3±4.1	-7.9±10.8	-31.9±3.3	-80.2±9.9	3.4 ±0.1	6.9±0.4
CC	4	ZINC000071763967	-9.5	-47.6±3.1	-16.3±5.3	-40.6±3.5	-104.5±7.6	2.5±0.1	2.2±0.2
CC	5	ZINC000067947687	-9.5	-42.0±3.7	-21.9±11.4	-37.1±2.6	-88.4±3.6	2.9±0.2	3.2±0.1
**CC**	**6**	**ZINC000634950507**	**-9.4**	**-42.7±2.9**	**-11.4±7.3**	**-38.1±2.9**	**-92.2±7.6**	**2.2±0.1**	**2.6±0.2**
**CC**	**7**	**ZINC000072227493**	**-9.4**	**-38.6±3.9**	**-19.6±6.8**	**-43.2±4.6**	**-101.4±13.6**	**2.4±0.1**	**1.2±0.4**
CC	8	ZINC000257262766	-9.4	-44.1±9.2	-23.1±21.4	-41.8±5.8	-108.9±29.6	2.4±0.2	12.2±0.7
**CC**	**9**	**ZINC000006645700**	**-9.3**	**-41.4±2.8**	**-19.7±4.5**	**-41.2±1.9**	**-102.3±5.0**	**3.0±0.2**	**3.6±0.2**
CC	10	ZINC000082157638	-9.3	-42.1±2.9	-15.3±6.2	-36.3±2.5	-93.7±7.6	2.4±0.1	3.2±0.2
CC	11	ZINC000091782363	-9.3	-42.4±3.6	-24.5±3.9	-32.0±2.2	-98.9±6.0	2.9±0.1	1.5±0.2
**CC**	**12**	**ZINC000091595762**	**-9.3**	**-49.9±3.7**	**-17.9±3.9**	**-48.0±4.1**	**-115.9±7.3**	**2.5±0.2**	**1.9+0.2**
CC	13	ZINC000067558896	-9.1	-39.7±2.7	-19.9±6.8	-38.6±3.2	-98.3±6.7	3.3±0.1	3.6±0.2
CC	14	ZINC000757213857	-9.0	-22.1±4.6	-60.3±8.7	-29.3±2.6	-111.8±9.2	3.0±0.1	3.7±0.3
**CC**	**15**	**ZINC000218867772**	**-9.0**	**-29.2±2.8**	**-23.6±5.9**	**-38.7±3.3**	**-91.5±7.8**	**2.6±0.1**	**3.7±0.4**
CC	16	ZINC000013637398	-8.9	-47.6±4.7	-25.0±7.4	-24.3±2.2	-97.0±7.9	2.2±0.1	1.7±0.2
**CC**	**17**	**ZINC000078515864**	**-8.9**	**-36.2±3.8**	**-25.9±5.3**	**-50.9±2.9**	**-113.1±6.1**	**2.6±0.1**	**2.7±0.2**
CC	18	ZINC000014538415	-8.9	-65.5±5.0	-25.4±6.3	-62.6±6.3	-153.6±11.6	3.2±0.1	5.4±0.2
CC	19	ZINC000218873420	-8.9	-23.1±3.6	-53.8±6.0	-29.1±2.4	-106.1±7.1	2.5±0.1	2.4±0.2
**CC**	**20**	**ZINC000001743629**	**-8.9**	**-43.7±3.1**	**-17.7±7.1**	**-50.8±3.8**	**-112.3±9.7**	**2.4±0.1**	**3.9±0.8**
**CC**	**21**	**ZINC000028295259**	**-8.9**	**-47.0±5.9**	**-19.3±6.2**	**-45.1±4.8**	**-111.4±8.9**	**2.6±0.1**	**3.3±0.2**
CC	22	ZINC000014536536	-8.9	-51.3±3.9	-23.5±3.3	-52.6±4.7	-127.5±7.5	2.8±0.1	3.0±0.2
**CC**	**23**	**ZINC000147095213**	**-8.8**	**-31.4±3.6**	**-34.4±5.7**	**-39.1±3.8**	**-104.9±8.7**	**3.8±0.1**	**2.9±0.2**
CC	24	ZINC000095698957	-8.8	-40.5±4.4	-22.0±8.8	-38.4±2.9	-100.9±8.4	3.1±0.1	3.2±0.2
**CC**	**25**	**ZINC000014888594**	**-8.8**	**-46.9±4.2**	**-30.8±5.5**	**-44.7±3.1**	**-122.5±7.8**	**3.4±0.2**	**3.0±0.4**
CC	26	ZINC000015080658	-8.8	-50.0±3.7	-17.0±5.3	-36.5±4.7	-103.5±11.3	3.5±0.2	2.4±0.3
CC	27	ZINC000096027748	-8.8	-24.6±3.3	-27.0±15.4	-26.2±2.5	-77.8±16.4	2.5±0.1	2.4±0.3
CC	28	ZINC000067675691	-8.8	-47.5±4.9	-22.0±6.2	-45.2±6.1	-114.7±12.4	3.2±0.1	5.4±0.3
CC	29	ZINC000011691661	-8.7	-40.4±7.1	-37.2±19.1	-42.2±3.3	-119.8±16.1	4.0±0.2	8.1±0.3
**CC**	**30**	**ZINC000096312018**	**-8.7**	**-43.6±3.3**	**-16.5±7.5**	**-36.7±2.7**	**-96.8±8.2**	**2.8±0.1**	**3.6±0.4**
**CC**	**31**	**ZINC000248261142**	**-8.7**	**-42.3±4.5**	**-22.7±7.4**	**-44.2±3.9**	**-109.2±9.3**	**3.3±0.1**	**3.0±0.5**
CC	32	ZINC000036160706	-8.7	-53.2±5.0	-23.1±5.0	-43.7±3.1	-120.0±10.0	2.6±0.1	4.3±0.6
**C1**	**1**	**ZINC000025329384**	**-10.2**	**-57.2±3.6**	**-19.9±9.4**	**-57.7±4.1**	**-134.9±11.3**	**2.4±0.1**	**2.9±0.4**
**C1**	**2**	**ZINC000037556415**	**-9.4**	**-58.5±2.4**	**-9.6±2.3**	**-43.8±2.6**	**-111.9±5.1**	**3.4±0.2**	**6.0±0.1**
C1	3	ZINC000095456365	-9.2	-53.6±3.8	-24.6±5.8	-36.2±1.9	-114.5±6.2	2.1 ±0.1	3.1±0.2
C1	4	ZINC000225318193	-9.2	-33.6±6.7	-12.4±5.3	-26.6±5.5	-72.6±14.8	2.1±0.2	9.7±0.6
C1	5	ZINC000299770618	-9.2	-48.0±4.2	-8.7±5.6	-35.7±3.7	-92.5±7.3	2.9±0.1	2.5±0.3
**C1**	**6**	**ZINC000827360794**	**-9.1**	**-43.7±3.5**	**-18.8±8.4**	**-41.6±2.8**	**-104.2±11.1**	**2.4±0.1**	**2.9±0.1**
C1	7	ZINC000035373220	-9.1	-56.6±4.2	-17.3±9.4	-54.8±3.1	-128.8±9.1	2.3±0.1	1.0±0.1

**C1**	**8**	**ZINC000072430969**	**-9.0**	**-46.3±3.0**	**-5.2±3.3**	**-44.1±3.1**	**-95.7±5.8**	**2.2±0.1**	**1.9±0.2**
**C1**	**9**	**ZINC000078648574**	**-9.0**	**-50.4±5.2**	**-4.1±3.5**	**-54.1±6.5**	**-108.7±13.1**	**2.8±0.1**	**6.3±0.5**
**C1**	**10**	**ZINC000005344596**	**-9.0**	**-53.9±3.8**	**-7.5±5.5**	**-39.5±4.8**	**-100.9±11.6**	**3.5±0.3**	**2.2±0.4**
C1	11	ZINC000069348668	-8.9	-57.2±2.4	-13.9±3.4	-44.5±2.3	-115.7±5.8	2.2±0.1	3.9±0.1
**C2**	**1**	**ZINC000057999653**	**-10.1**	**-47.1±4.1**	**-8.6±13.5**	**-38.9±2.4**	**-94.6±10.9**	**2.3±0.1**	**2.7±0.2**
C2	2	ZINC000002877267	-9.9	-48.2±5.3	-3.8±6.1	-40.2±4.1	-92.3±9.2	2.5±0.1	6.7±0.6
C2	3	ZINC000408693879	-9.8	-61.9±4.6	-31.9±13.1	-54.7±3.1	-148.5±14.0	3.8±0.1	3.2±0.2
C2	4	ZINC000000880008	-9.8	-61.5±3.8	-12.0±4.3	-49.0±3.2	-122.6±6.3	3.1±0.1	2.8±0.3
**C2**	**5**	**ZINC000006664413**	**-9.8**	**-46.4±2.8**	**-20.1±7.0**	**-43.4±3.2**	**-109.9±8.9**	**2.6±0.1**	**3.6±0.3**
C2	6	ZINC000001408226	-9.7	-35.0±4.1	-17.3±7.9	-23.5±3.0	-75.8±11.4	3.3±0.1	3.3±0.3
C2	7	ZINC000001045477	-9.7	-45.9±5.1	-11.7±5.7	-28.3±2.4	-86.0±11.7	2.9±0.1	3.6±0.2
C2	8	ZINC000005493735	-9.7	-46.3±3.0	-5.2±3.3	-44.1±3.1	-95.7±5.8	2.2±0.1	1.9±0.2
C2	9	ZINC000001641702	-9.6	-36.2±3.4	-2.6±5.0	-30.3±2.2	-69.1±6.0	2.8±0.2	5.7±0.3
C2	10	ZINC000000302628	-9.5	-39.3±2.7	-12.9±3.3	-36.7±1.9	-89.1±3.8	2.6±0.1	1.8±0.3
C2	11	ZINC000095418373	-9.3	-48.3±3.1	-9.0±5.4	-36.1±2.9	-93.5±7.6	2.6±0.1	4.0±0.2
**C2**	**12**	**ZINC000006750553**	**-9.3**	**-44.6±3.5**	**-21.4±8.8**	**-42.9±3.1**	**-109.1±10.8**	**2.9±0.1**	**2.8+0.3**
C2	13	ZINC000097002851	-9.3	-34.0±5.7	-29.8±9.4	-27.3±3.7	-91.2±10.2	2.9±0.1	6.6±0.6
C2	14	ZINC000005776998	-9.3	-43.2±3.3	-25.4±6.5	-44.0±3.3	-112.6±9.2	3.3±0.1	2.7±0.8
C2	15	ZINC000004663101	-9.3	-45.0±2.7	-6.9±3.5	-47.9±2.5	-99.9±5.3	2.9±0.1	2.8±0.3
C2	16	ZINC000005604766	-9.2	-35.7±3.3	-18.2±14.1	-32.7±2.8	-86.6±14.7	3.0±0.1	3.5±0.2
C2	17	ZINC000225173433	-9.1	-51.5±3.7	-5.2±2.1	-47.2±2.7	-103.9±5.9	2.0±0.1	3.0±0.1
C2	18	ZINC000034720963	-9.1	-49.2±5.6	-50.2±13.8	-44.9±3.4	-144.3±10.4	2.8±0.1	5.4±0.3
C2	19	ZINC000409066936	-9.1	-40.1±2.6	-4.0±5.9	-34.2±3.3	-78.4±9.5	2.9±0.1	5.2±0.4
C2	20	ZINC000014750115	-9.0	-50.6±3.4	-12.2±2.9	-43.3±3.5	-106.1±6.7	3.0±0.1	3.4±0.2
C2	21	ZINC000059677349	-8.9	-52.4±2.5	-3.6±2.7	-50.7±3.3	-106.7±4.6	3.3±0.2	4.5±0.2
C2	22	ZINC000002690402	-8.9	-67.4±6.3	-14.6±3.9	-47.7±4.7	-129.8±11.1	2.9±0.1	3.7±0.2
C2	23	ZINC000020572602	-8.9	-53.5±3.2	-9.5±3.3	-53.5±3.6	-116.6±6.2	2.5±0.1	2.2±0.3
C2	24	ZINC000408729576	-8.9	-33.7±3.4	-24.7±11.3	-29.9±2.2	-88.4±11.9	2.8±0.1	1.5±0.3
C2	25	ZINC000009660785	-8.9	-58.5±4.6	-21.7±4.5	-49.4±3.0	-129.7±8.5	2.8±0.1	2.0±0.3
C2	26	ZINC000334160398	-8.8	-58.9±3.5	-21.5±4.1	-42.0±1.9	-122.5±5.7	2.7±0.1	2.4±0.3

[1]: Based on the snapshots from the last 20ns of simulation

Note: VDW is Van Der Waals interaction and ELE is electrostatic interaction.

Table S3. The predicted ADME properties for top 32 best compounds in reference to the crystal conformation (PDB ID: 6PT3), the top 11 compounds in reference to the first conformation from the crystal MD simulation (C1), and the top 26 compounds in reference to the second conformation from the crystal MD simulation (C2), including the crystal reference compound from the SwissADME server.

Structure	Compound	GI absorption	BBB permeant	CYP1A2	CYP2C19	CYP2C9	CYP2D6	CYP3A4	Lipinksi rule	PAINS	Brenk
	Crystal Structure (PDB ID:6PT3)	High	Yes	No	No	No	Yes	Yes	Yes; 0 violation	0 alert	0 alert
CC	ZINC000020559278	High	Yes	Yes	No	No	Yes	Yes	Yes; 0 violation	0 alert	0 alert
CC	ZINC000014242201	Low	No	No	No	No	No	Yes	Yes; 0 violation	0 alert	0 alert
CC	ZINC000562639987	High	No	No	No	No	No	No	Yes; 0 violation	0 alert	0 alert
CC	ZINC000071763967	High	Yes	No	No	No	No	No	Yes; 0 violation	1 alert: catechol_A	1 alert: catechol
CC	ZINC000067947687	High	Yes	Yes	No	No	Yes	No	Yes; 0 violation	1 alert: catechol_A	1 alert: catechol
CC	ZINC000634950507	High	Yes	No	No	No	No	No	Yes; 0 violation	0 alert	0 alert
CC	ZINC000072227493	High	Yes	Yes	Yes	Yes	No	No	Yes; 0 violation	0 alert	0 alert
CC	ZINC000257262766	High	No	No	No	No	Yes	Yes	Yes; 0 violation	0 alert	0 alert
CC	ZINC000006645700	High	Yes	Yes	Yes	No	Yes	No	Yes; 0 violation	0 alert	0 alert
CC	ZINC000082157638	High	No	No	No	No	No	No	Yes; 0 violation	0 alert	0 alert
CC	ZINC000091782363	Low	No	No	No	No	No	No	Yes; 0 violation	0 alert	1 alert: hydantoin
CC	ZINC000091595762	High	Yes	No	No	No	No	No	Yes; 0 violation	0 alert	0 alert
CC	ZINC000067558896	High	No	No	No	No	No	No	Yes; 0 violation	0 alert	0 alert
CC	ZINC000757213857	High	No	No	No	No	No	No	Yes; 0 violation	0 alert	0 alert
CC	ZINC000218867772	High	Yes	No	No	No	No	No	Yes; 0 violation	0 alert	0 alert
CC	ZINC000013637398	High	No	Yes	Yes	No	No	Yes	Yes; 0 violation	0 alert	1 alert: thiocarbonyl_group
CC	ZINC000078515864	High	Yes	No	No	No	Yes	No	Yes; 0 violation	0 alert	0 alert
CC	ZINC000014538415	High	No	No	No	No	No	No	Yes; 0 violation	0 alert	0 alert
CC	ZINC000218873420	High	No	Yes	No	No	No	No	Yes; 0 violation	0 alert	0 alert
CC	ZINC000001743629	High	Yes	Yes	Yes	Yes	Yes	No	Yes; 0 violation	0 alert	0 alert
CC	ZINC000028295259	High	Yes	No	No	No	No	No	Yes; 0 violation	0 alert	0 alert
CC	ZINC000014536536	High	No	No	No	No	No	No	Yes; 0 violation	0 alert	0 alert
CC	ZINC000147095213	High	Yes	Yes	No	No	No	No	Yes; 0 violation	0 alert	0 alert
CC	ZINC000095698957	Low	No	No	Yes	No	No	No	Yes; 0 violation	0 alert	0 alert
CC	ZINC000014888594	High	Yes	No	No	No	No	No	Yes; 0 violation	0 alert	0 alert
CC	ZINC000015080658	High	No	No	No	No	No	No	Yes; 0 violation	0 alert	1 alert: phthalimide
CC	ZINC000096027748	High	No	No	No	No	No	No	Yes; 0 violation	0 alert	2 alerts: imine_1, imine_2
CC	ZINC000067675691	High	No	No	No	No	No	No	Yes; 0 violation	0 alert	0 alert
CC	ZINC000011691661	High	No	Yes	Yes	Yes	No	Yes	Yes; 0 violation	0 alert	1 alert: isolated_alkene
CC	ZINC000096312018	High	Yes	Yes	Yes	Yes	Yes	Yes	Yes; 0 violation	0 alert	0 alert
CC	ZINC000248261142	High	Yes	Yes	No	No	Yes	No	Yes; 0 violation	0 alert	0 alert
CC	ZINC000036160706	High	No	No	No	Yes	No	Yes	Yes; 0 violation	0 alert	1 alert: nitro_group
C1	ZINC000025329384	High	Yes	No	No	No	Yes	Yes	Yes; 0 violation	0 alert	0 alert
C1	ZINC000037556415	High	Yes	No	Yes	Yes	Yes	Yes	Yes; 0 violation	0 alert	0 alert
C1	ZINC000095456365	High	No	No	Yes	Yes	Yes	Yes	Yes; 0 violation	0 alert	0 alert
C1	ZINC000225318193	High	Yes	Yes	Yes	No	No	No	Yes; 0 violation	0 alert	0 alert

C1	ZINC000299770618	High	No	Yes	No	No	Yes	No	Yes; 0 violation	0 alert	0 alert
C1	ZINC000827360794	High	Yes	No	No	No	Yes	Yes	Yes; 0 violation	0 alert	0 alert
C1	ZINC000035373220	High	No	No	No	Yes	No	No	Yes; 0 violation	1 alert: catechol_A	1 alert: catechol
C1	ZINC000072430969	High	Yes	Yes	Yes	Yes	Yes	Yes	Yes; 0 violation	0 alert	0 alert
C1	ZINC000078648574	High	Yes	No	Yes	Yes	Yes	No	Yes; 0 violation	0 alert	0 alert
C1	ZINC000005344596	High	Yes	No	No	Yes	No	No	Yes; 0 violation	0 alert	0 alert
C1	ZINC000069348668	High	Yes	Yes	Yes	Yes	Yes	Yes	Yes; 0 violation	0 alert	1 alert: triple_bond
C2	ZINC000057999653	High	Yes	Yes	Yes	Yes	Yes	Yes	Yes; 0 violation	0 alert	0 alert
C2	ZINC000002877267	High	No	Yes	Yes	Yes	Yes	Yes	Yes; 0 violation	0 alert	0 alert
C2	ZINC000408693879	High	No	No	No	Yes	No	Yes	Yes; 0 violation	1 alert: ene_six_het_A	2 alerts: michael_acceptor_1, michael_acceptor_4
C2	ZINC000000880008	High	No	No	Yes	Yes	Yes	Yes	Yes; 0 violation	0 alert	1 alert: thiocarbonyl_group
C2	ZINC000006664413	High	Yes	Yes	Yes	Yes	Yes	Yes	Yes; 0 violation	0 alert	0 alert
C2	ZINC000001408226	High	Yes	Yes	No	No	No	No	Yes; 0 violation	0 alert	1 alert: imine_1
C2	ZINC000001045477	Low	No	Yes	Yes	Yes	Yes	No	Yes; 1 violation: MLOGP>4.15	0 alert	1 alert: thiocarbonyl_group
C2	ZINC000005493735	High	No	No	Yes	Yes	No	Yes	Yes; 0 violation	0 alert	0 alert
C2	ZINC000001641702	High	Yes	Yes	Yes	Yes	Yes	No	Yes; 0 violation	0 alert	0 alert
C2	ZINC000000302628	High	Yes	Yes	Yes	No	No	No	Yes; 0 violation	0 alert	0 alert
C2	ZINC000095418373	High	No	Yes	Yes	Yes	Yes	Yes	Yes; 0 violation	0 alert	0 alert
C2	ZINC000006750553	High	Yes	Yes	Yes	No	Yes	No	Yes; 0 violation	0 alert	0 alert
C2	ZINC000097002851	High	No	Yes	No	No	Yes	No	Yes; 0 violation	0 alert	0 alert
C2	ZINC000005776998	High	No	No	No	No	Yes	No	Yes; 0 violation	1 alert: indol_3yl_alk	0 alert
C2	ZINC000004663101	High	Yes	Yes	Yes	No	Yes	No	Yes; 0 violation	1 alert: anil_alk_ene	1 alert: isolated_alkene
C2	ZINC000005604766	High	No	Yes	Yes	No	No	No	Yes; 0 violation	0 alert	2 alerts: imine_1, imine_2
C2	ZINC000225173433	High	No	Yes	Yes	Yes	No	Yes	Yes; 0 violation	0 alert	1 alert: thiocarbonyl_group
C2	ZINC000034720963	High	No	Yes	Yes	Yes	No	No	Yes; 1 violation: MLOGP>4.15	1 alert: ene_five_het_B	1 alert: michael_acceptor_1
C2	ZINC000409066936	High	Yes	Yes	Yes	Yes	Yes	Yes	Yes; 0 violation	0 alert	1 alert: michael_acceptor_1
C2	ZINC000014750115	High	No	No	No	No	No	No	Yes; 0 violation	0 alert	0 alert
C2	ZINC000059677349	High	Yes	Yes	Yes	No	Yes	Yes	Yes; 0 violation	1 alert: indol_3yl_alk	0 alert
C2	ZINC000002690402	Low	No	No	No	Yes	No	Yes	Yes; 0 violation	0 alert	1 alert: thiocarbonyl_group
C2	ZINC000020572602	High	No	No	No	Yes	No	Yes	Yes; 0 violation	1 alert: hzone_phenol_B	1 alert: imine_1
C2	ZINC000408729576	High	No	No	No	No	No	No	Yes; 0 violation	0 alert	1 alert: imine_1
C2	ZINC000009660785	High	No	No	No	Yes	Yes	Yes	Yes; 0 violation	0 alert	1 alert: hydantoin
C2	ZINC000334160398	High	No	No	No	Yes	Yes	Yes	Yes; 0 violation	0 alert	0 alert

Note: ADME is absorption, distribution, metabolism, and excretion, GI is gastro-intestinal, and BBB is blood brain barrier.

Table S4. Critical nodes identified from the Network Analysis for the DPI-287/DOR system and the top 8 compound systems. Bolded representation indicates the same residues as the crystal complex.

**Critical Nodes**								
**Crystal**	**CC1**	**CC17**	**C1 01**	**C1 02**	**C1 06**	**C1 09**	**C2 01**	**C2 05**
**V59**	L48	**V59**	A43	L48	Y56	L48	62	L55
**G63**	N67	I88	L48	S57	**G63**	A61	88	**V59**
**K81**	L69	**D95**	L55	C60	**L91**	L65	**D95**	N67
**Y87**	I74	**A98**	**V59**	I86	**D95**	I86	T99	V70
**L91**	**L91**	T99	A92	**Y87**	T99	I88	A107	M80
**A94**	**D95**	T101	**D95**	I88	T101	N90	M111	T84
**D95**	**A98**	L102	L97	**L91**	L102	**L91**	T113	I86
**A98**	T101	F104	T113	T113	Q105	L93	F116	**L91**
**Y129**	L102	Q105	A123	**M132**	Y109	**D95**	Y130	L97
**N131**	F104	Y109	I127	F133	T113	L97	F133	**A98**
**M132**	T113	T113	D128	T134	A123	T99	T134	K108
**S135**	V124	L120	**Y129**	**F137**	I127	L102	**I136**	Y109
**I136**	I127	I127	**M132**	T138	Y130	T113	T138	L110
**F137**	D128	D128	**L139**	**T140**	T134	I127	**L139**	M111
**L139**	**M132**	**N131**	S143	L157	**I136**	D128	**T140**	E112
**T140**	T138	F133	A165	T161	**F137**	**M132**	R146	I127
**M142**	**T140**	**S135**	N169	I172	T138	**I136**	G178	Y130
**Y147**	**M142**	**I136**	V196	A176	**T140**	**L139**	P182	**N131**
**A149**	**M186**	**F137**	Q201	G180	**M142**	V150	**M186**	F133
**R160**	V196	T138	P203	**M186**	S143	L157	F202	T134
**P162**	F202	**L139**	**V217**	V196	D145	T161	**K214**	T138
**M186**	**K214**	**T140**	F218	F202	L157	I172	I215	L157
**V188**	F218	M141	V224	F218	T161	A176	F218	**R160**
**K214**	**L219**	S143	L227	**L219**	V196	V196	**L219**	T161
**C216**	A221	**M186**	I229	V224	L200	Q201	F220	**P162**
**V217**	F222	V196	R244	P225	F202	P203	F222	K166
**L219**	V224	F202	D253	R244	V223	**K214**	M236	I172
**A269**	P225	**L219**	V266	D253	L227	**C216**	R239	W173
**F270**	V265	F220	G268	R258	M262	F218	M262	**M186**
**W274**	**A269**	F222	**F270**	M262	V265	**L219**	C273	F202
**I277**	**H278**	V224	**W274**	V267	**A269**	F220	**I277**	W209
**H278**	V281	P225	**H278**	**F270**	A275	F222	V281	V212
	A298	I229	R292	**W274**	I279	R239	C303	P225
	Y308	Y233	P294	**H278**	V297	R261	N310	I228
	N310	M262	A305	V297	A298	V265	P315	V281
	Y318	V266	L306	L306	H301	**A269**	V316	V287
		**F270**	G307	Y308	A305	**F270**	Y318	I289
		V271	Y308	A309	G307	**H278**		
		**W274**	N310	N310	Y308	V297		
		V297	S312	L313	N310	N310		
		A309	Y318		S311	S312		
		S311	L321		L313	N314		
		L313			N314			
		Y318			Y318			
		L321			L321			
		N324						
		K326						
**32 total**	**36 total**	**47 total**	**42 total**	**40 total**	**45 total**	**42 total**	**37 total**	**37 total**
	**11 same**	**13 same**	**9 same**	**10 same**	**8 same**	**11 same**	**8 same**	**7 same**

Table S5. The optimal path of the transmission and tyrosine toggle switch generated from the Network Analysis for the DPI-287/DOR system crystal complex and the top 8 ZINC compounds. Bolded represents the same residue as the crystal complex.

**Molecular Switch Optimal Path**								
**Crystal**	**CC1**	**CC17**	**C1 01**	**C1 02**	**C1 06**	**C1 09**	**C2 01**	**C2 05**
**Transmission Switch**								
Ligand	Ligand	Ligand	Ligand	Ligand	Ligand	Ligand	Ligand	Ligand
**W274**	**W274**	**W274**	**W274**	**W274**	I304	V281	Y308	N131
**F270**	**F270**	**F270**	**F270**	**F270**	L306	I279	S312	F133
**G268**	V266	V266	**G268**	V267	C273	A275	N314	F137
**L264**	M262	M262	**L264**	V265	A269	V271	Y318	T140
**R261**	**R258**	**R258**	**R261**	M262	V265	**G268**	M262	V144
**R258**	**S255**	**S255**	**R258**	**R258**	M262	V265	**R258**	R146
**S255**			**S255**	**S255**	**R258**	**R261**	**S255**	I259
					**S255**	**R258**		**S255**
						**S255**		
**7 total**	**6 total**	**6 total**	**7 total**	**7 total**	**8 total**	**9 total**	**7 total**	**8 total**
	**4 same**	**4 same**	**7 same**	**4 same**	**2 same**	**4 same**	**2 same**	**1 same**
**Crystal**	**CC1**	**CC17**	**C1 01**	**C1 02**	**C1 06**	**C1 09**	**C2 01**	**C2 05**
**Tyrosine Toggle Switch**								
Ligand	Ligand	Ligand	Ligand	Ligand	Ligand	Ligand	Ligand	Ligand
**G307**	Y308	Y308	S311	S311	Y308	S311	Y308	S311
**N310**	S311	**N310**	**N314**	P315	**N310**	**N314**	S312	**N314**
**N314**	**N314**	**N314**	**Y318**	**Y318**	**N314**	**Y318**	**N314**	**Y318**
**Y318**	**Y318**	**Y318**			**Y318**		**Y318**	
**4 total**	**4 total**	**4 total**	**3 total**	**3 total**	**4 total**	**3 total**	**4 total**	**3 total**
	**2 same**	**3 same**	**2 same**	**1 same**	**3 same**	**2 same**	**2 same**	**2 same**

Mode 1	Mode 6
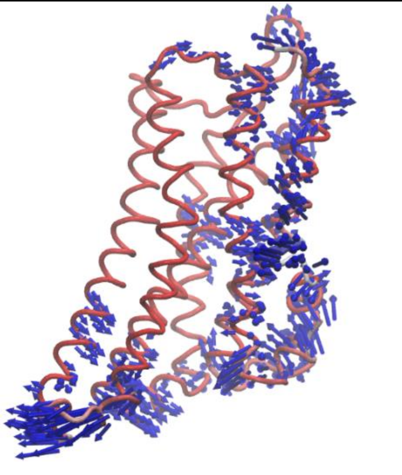	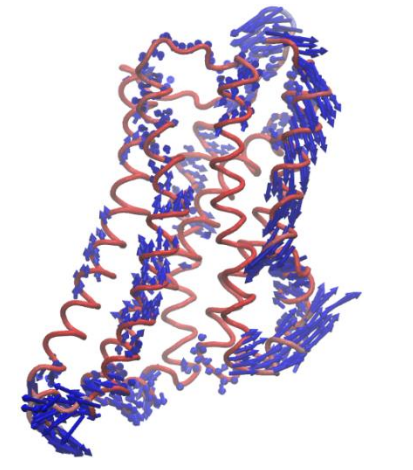
**Mode 2**	**Mode 7**
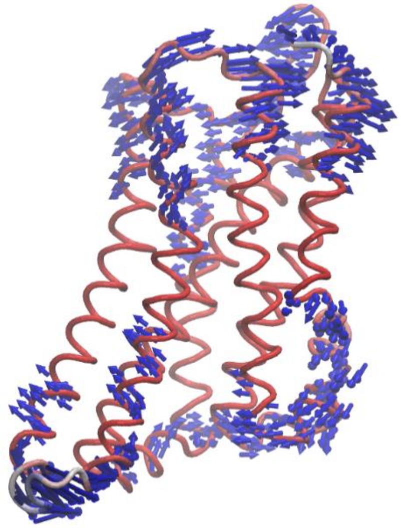	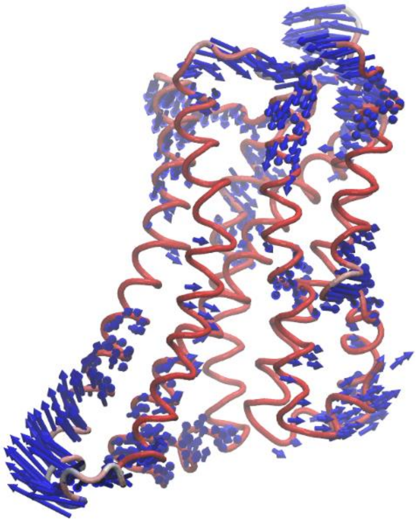
**Mode 3**	**Mode 8**
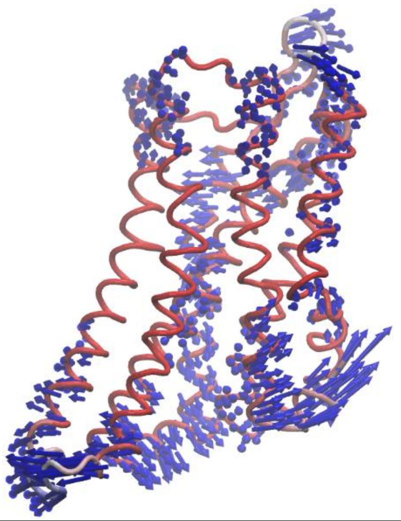	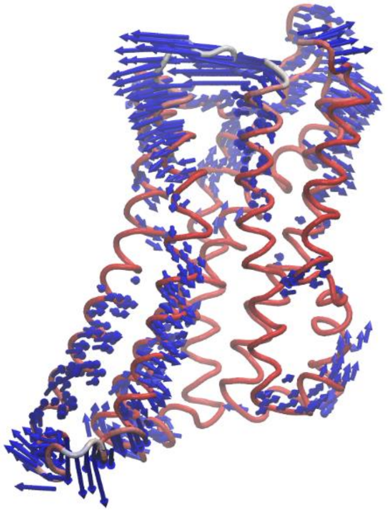
**Mode 4**	**Mode 9**

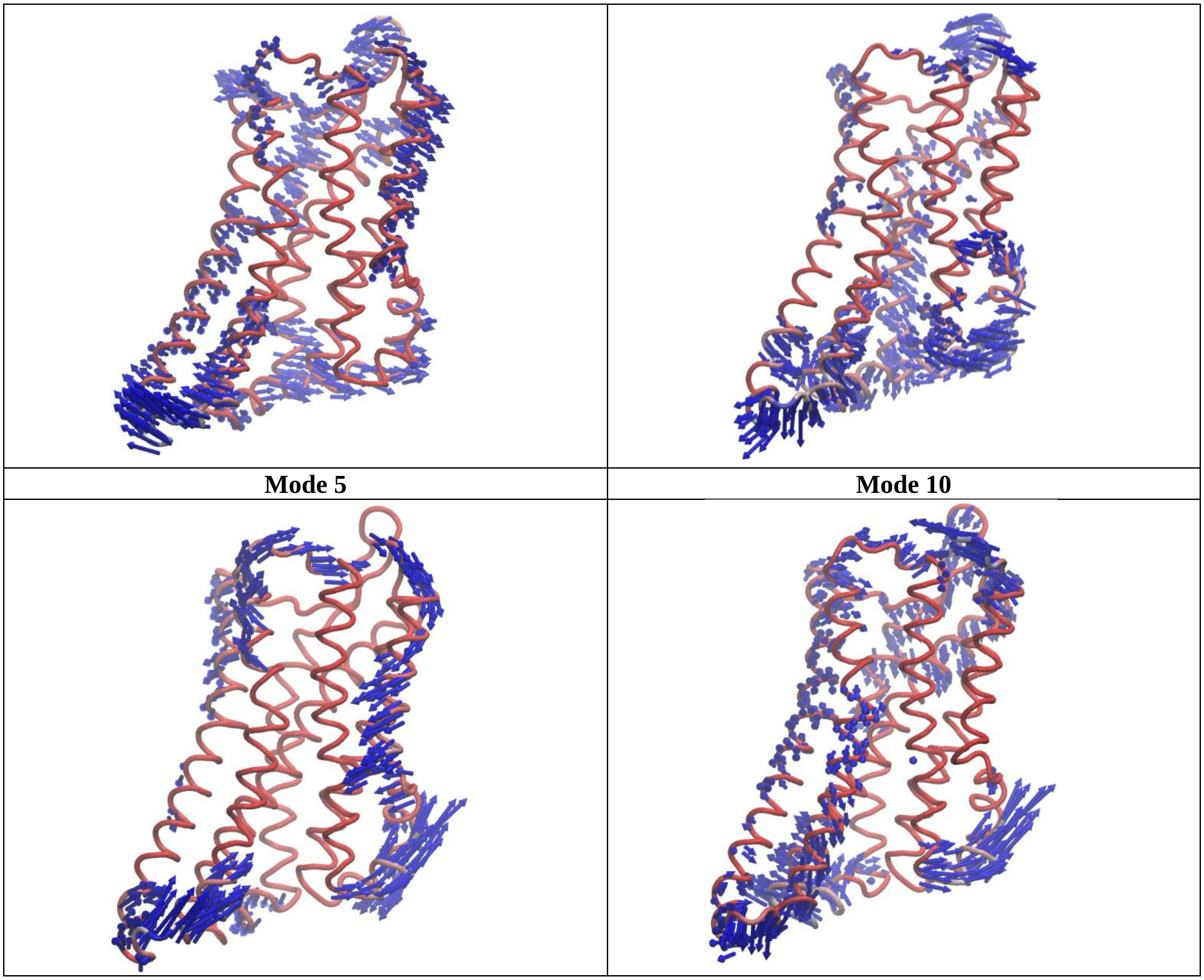

Figure S1. Top 10 low vibrational modes from the normal mode analysis (PCA) based on the active conformation DOR agonist DPI-287 system.

No	FDA approved drugs/ Structure/ Docking score	No	FDA approved drugs/ Structure/ Docking score
1	Lobivon; Nebivolol<br>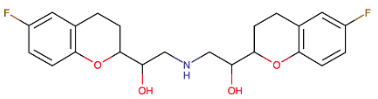<br>Docking Score: -8.467 kcal/mol	6	Eluxadoline<br>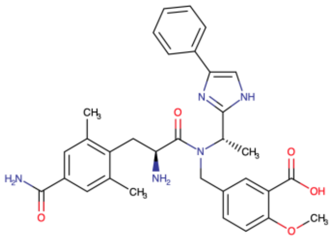<br>Docking Score: -7.297 kcal/mol
2	Lanreotide<br>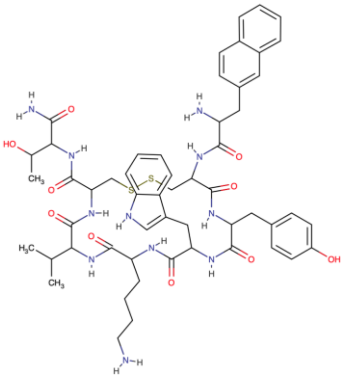<br>Docking Score: -8.037 kcal/mol	7	Oxamniquine; Mansil<br>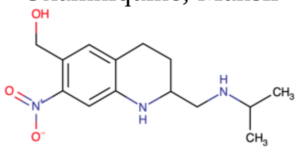<br>Docking Score: -7.202 kcal/mol
3	Dobuject; Dobutamine<br>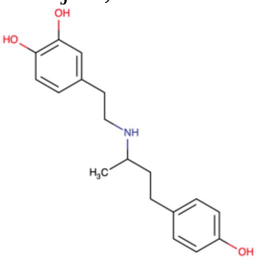<br>Docking Score: -7.638 kcal/mol	8	Hydroxyethyl cellulose<br>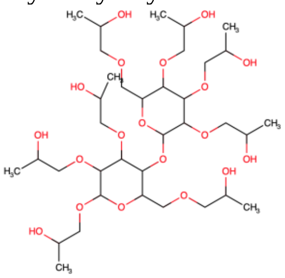<br>Docking Score: -7.132 kcal/mol
4	Lercanidipine<br>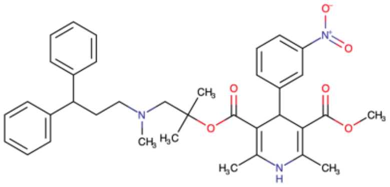<br>Docking Score: -7.504 kcal/mol	9	Terbutaline; Brethaire<br>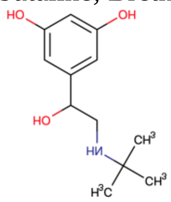<br>Docking Score: -7.054 kcal/mol
5	Nylidrin; Arbid<br>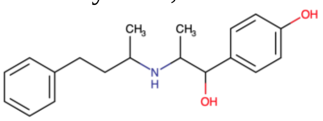<br>Docking Score: -7.482 kcal/mol	10	Fenoterol; Alveofen<br>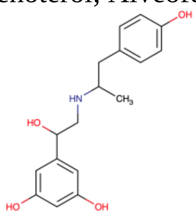<br>Docking Score: -6.965 kcal/mol

Figure S2. Top 10 FDA-approved drug-compounds based on their docking scores for the crystal conformation of the DOR.

No.	Zinc ID/ Structure/ SMILE_code	Docking score	# STAR	# Cluster ID	Centroid
Ref. Comp	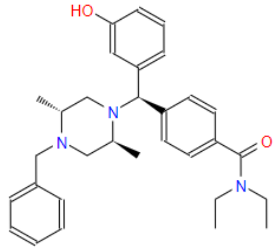	-8.635 kcal/mol	0		Centroid
1	ZINC000020559278 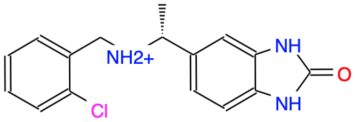 C[C@H](c1ccc2c(c1)[nH]c(=O)[nH]2)[NH2+]Cc3ccccc3Cl	−9.999 kcal/mol	0	39	Centroid
2	ZINC000014242201 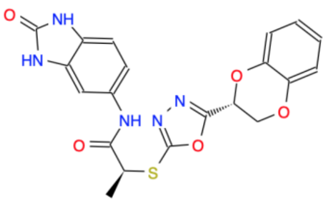 C[C@H](Sc1nnc([C@H]2COc3ccccc3O2)o1)C(=O)Nc1ccc2[nH]c(=O)[nH]c2c1	−9.918 kcal/mol	0	50	Centroid
3	ZINC000562639987 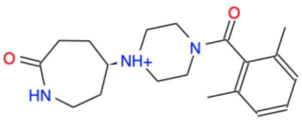 Cc1cccc(C)c1C(=O)N3CC[NH+]([C@H]2CCNC(=O)CC2)CC3	−9.592 kcal/mol	0	82	Centroid
4	ZINC000071763967 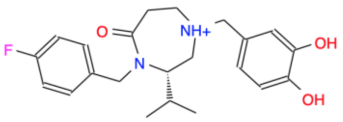 CC(C)[C@H]2C[NH+](Cc1ccc(O)c(O)c1)CCC(=O)N2Cc3ccc(F)cc3	−9.532 kcal/mol	0	38	Centroid
5	ZINC000067947687 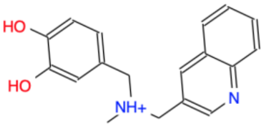 C[NH+](Cc1ccc(O)c(O)c1)Cc3cnc2ccccc2c3	−9.507 kcal/mol	0	40	Centroid
6	ZINC000634950507 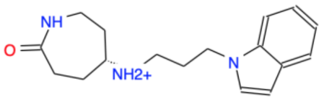 O=C3CC[C@@H]([NH2+]CCCn2ccc1ccccc12)CCN3	−9.445 kcal/mol	0	83	Centroid
7	ZINC000072227493	−9.431 kcal/mol	0	63	Centroid

	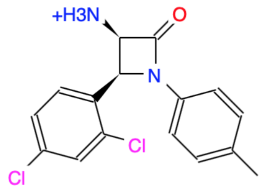<br>Cc3ccc(N1C(=O)[C@H]([NH3+])[C@@H]1c2ccc(Cl)cc2Cl)cc3				
8	ZINC000257262766<br>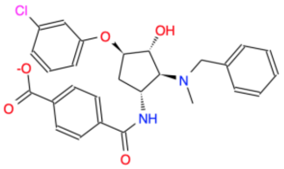<br>CN(Cc1ccccc1)[C@@H]3[C@@H](O)[C@H](Oc2cccc(Cl)c2)C[C@H]3N C(=O)c4ccc(C(=O)[O-])cc4	−9.401 kcal/mol	1	53	Centroid
9	ZINC000006645700<br>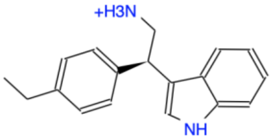<br>CCc3ccc([C@@H](C[NH3+])c1c[nH]c2ccccc12)cc3	−9.344 kcal/mol	1	70	Centroid
10	ZINC000082157638<br>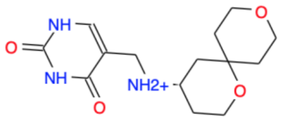<br>O=c3[nH]cc(C[NH2+][C@@H]2CCOC1(CCOCC1)C2)c(=O)[nH]3	−9.297 kcal/mol	0	44	Centroid
11	ZINC000091782363<br>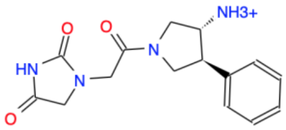<br>[NH3+][C@H]2CN(C(=O)CN1CC(=O)NC1=O)C[C@@H]2c3ccccc3	−9.287 kcal/mol	0	33	Centroid
12	ZINC000091595762<br>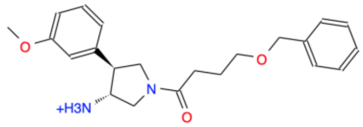<br>COc3cccc([C@H]2CN(C(=O)CCCOCc1ccccc1)C[C@@H]2[NH3+])c3	−9.282 kcal/mol	0	66	Centroid
13	ZINC000067558896<br>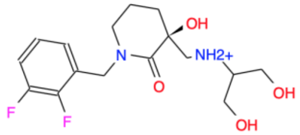<br>O=C2N(Cc1cccc(F)c1F)CCC[C@]2(O)C[NH2+]C(CO)CO	−9.095 kcal/mol	1	43	Centroid
14	ZINC000757213857<br>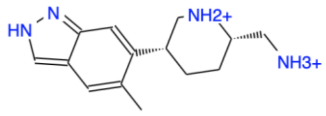<br>Cc2cc1c[nH]nc1cc2[C@H]3CC[C@@H](C[NH3+])[NH2+]C3	−9.056 kcal/mol	0	10	Centroid
15	ZINC000218867772<br>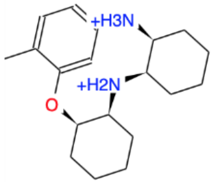<br>Cc1ccccc1O[C@@H]2CCCC[C@@H]2[NH2+][C@@H]3CCCC[C@@H] 3[NH3+]	−9.010 kcal/mol	0	8	Centroid

16	ZINC000013637398 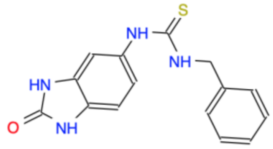 O=c1[nH]c2ccc(NC(=S)NCc3ccccc3)cc2[nH]1	−8.991 kcal/mol	0	55	Centroid
17	ZINC000078515864 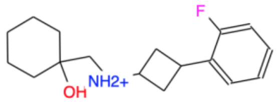 OC3(C[NH2+]C2CC(c1ccccc1F)C2)CCCCC3	−8.963 kcal/mol	0	9	Centroid
18	ZINC000014538415 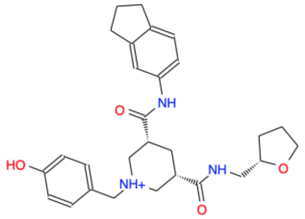 O=C(NC[C@@H]1CCCO1)[C@H]5C[C@@H](C(=O)Nc3ccc2CCCc2c3)C[NH+](Cc4ccc(O)cc4)C5	−8.948 kcal/mol	1	45	Centroid
19	ZINC000218873420 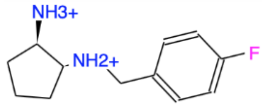 [NH3+][C@@H]1CCC[C@@H]1[NH2+]Cc2ccc(F)cc2	−8.934 kcal/mol	0	12	Centroid
20	ZINC000001743629 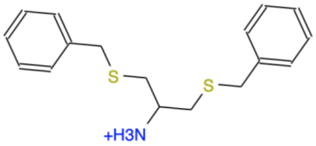 [NH3+]C(CSCc1ccccc1)CSCc2ccccc2	−8.897 kcal/mol	0	60	Centroid
21	ZINC000028295259 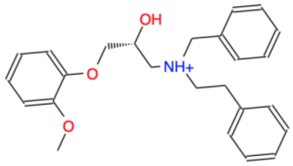 COc1ccccc1OC[C@H](O)C[NH+](CCc2ccccc2)Cc3ccccc3	−8.883 kcal/mol	1	48	Centroid
22	ZINC000014536536 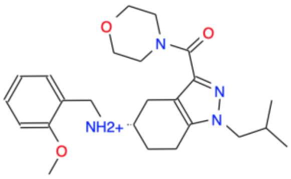 COc1ccccc1C[NH2+][C@H]2CCc4c(C2)c(C(=O)N3CCOCC3)nn4CC(C)C	−8.871 kcal/mol	0	64	Centroid
23	ZINC000147095213 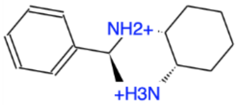 C[C@H]([NH2+][C@@H]1CCCC[C@@H]1[NH3+])c2ccccc2	−8.861 kcal/mol	0	14	Centroid

24	ZINC000095698957<br>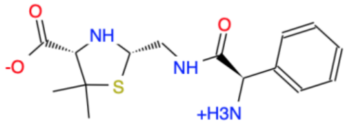<br>CC2(C)S[C@@H](CNC(=O)[C@H]([NH3+])c1ccccc1)N[C@H]2C(=O)[O-]	−8.860 kcal/mol	0	37	Centroid
25	ZINC000014888594<br>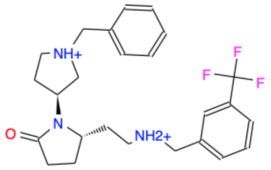<br>O=C2CC[C@@H](CC[NH2+]Cc1cccc(C(F)(F)F)c1)N2[C@H]4CC[NH+](Cc3ccccc3)C4	−8.849 kcal/mol	0	65	Centroid
26	ZINC000015080658<br>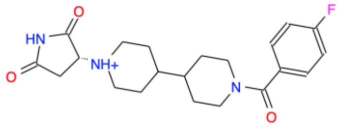<br>O=C4C[C@@H]([NH+]3CCC(C2CCN(C(=O)c1ccc(F)cc1)CC2)CC3)C(=O)N4	−8.849 kcal/mol	0	35	Centroid
27	ZINC000096027748<br>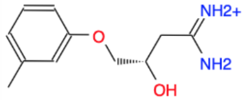<br>Cc1cccc(OC[C@@H](O)CC(N)=[NH2+])c1	−8.808 kcal/mol	0	73	Centroid
28	ZINC000067675691<br>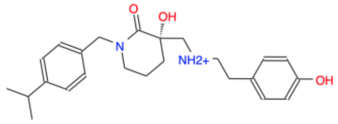<br>CC(C)c3ccc(CN2CCC[C@](O)(C[NH2+]CCc1ccc(O)cc1)C2=O)cc3	−8.780 kcal/mol	0	46	Centroid
29	ZINC000011691661<br>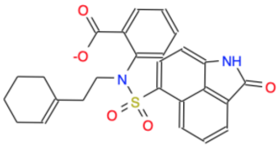<br>O=C([O-])c1ccccc1N(CCC2=CCCCC2)S(=O)(=O)c3ccc4[nH]c(=O)c5cccc3c45	−8.767 kcal/mol	1	41	Centroid
30	ZINC000096312018<br>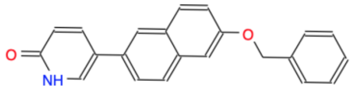<br>O=c1ccc(-c2ccc3cc(OCc4ccccc4)ccc3c2)c[nH]1	−8.727 kcal/mol	1	56	Centroid
31	ZINC000248261142<br>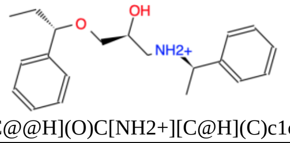<br>CC[C@H](OC[C@@H](O)C[NH2+][C@H](C)c1ccccc1)c2ccccc2	−8.709 kcal/mol	0	78	Centroid
32	ZINC000036160706<br>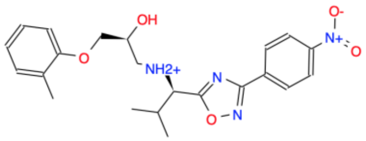<br>Cc1ccccc1OC[C@@H](O)C[NH2+][C@@H](c3nc(c2ccc(N(=O)=O)cc2)no3)C(C)C	−8.674 kcal/mol	0	49	Centroid

Figure S3. Top 32 ZINC compounds for the DOR crystal conformation including the crystal reference compound with Zinc ID, structure, SMILE code, docking score, number of STARs (indicator for "drug-likeness"), cluster IDs (ligand similarity clustering based on Canvas), and centroid (measures distances to the arithmetic means of clusters).

No.	Zinc ID/Structure/SMILE code	Docking score	# STAR	# Cluster ID	Centroid
Ref. Comp	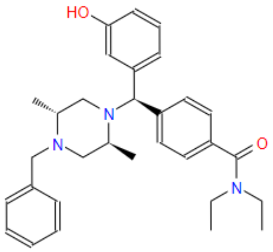	-8.635 kcal/mol			Yes Centroid
1	ZINC000025329384<br>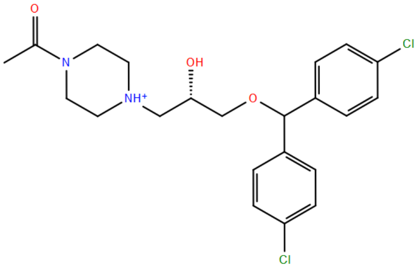<br>CC(=O)N1CCN(C[C@H](O)COC(c2ccc(Cl)cc2)c2ccc(Cl)cc2)CC1	-10.172 kcal/mol	0	58	Yes Centroid
2	ZINC000037556415<br>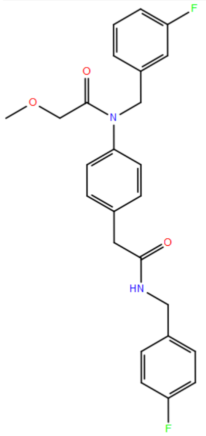<br>COCC(=O)N(Cc1cccc(F)c1)c1ccc(CC(=O)NCc2ccc(F)cc2)cc1	-9.450 kcal/mol	0	42	Yes Centroid
3	ZINC000095456365<br>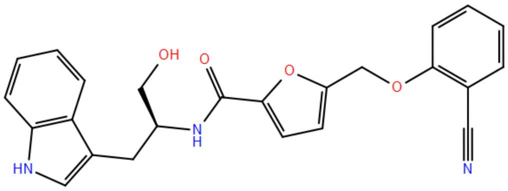<br>N#Cc1ccccc1OCc1ccc(C(=O)N[C@H](CO)Cc2c[nH]c3ccccc23)o1	-9.267 kcal/mol	0	49	Yes Centroid

4	ZINC000225318193<br>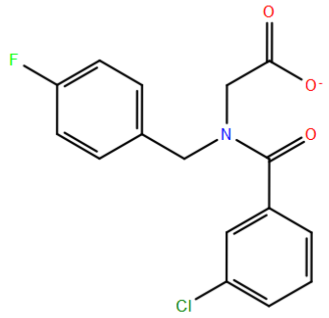<br>O=C(O)CN(Cc1ccc(F)cc1)C(=O)c1cccc(Cl)c1	-9.194 kcal/mol	0	16	Yes Centroid
5	ZINC000299770618<br>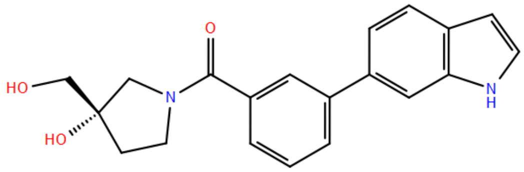<br>O=C(c1cccc(-c2ccc3cc[nH]c3c2)c1)N1CC[C@@](O)(CO)C1	-9.171 kcal/mol	0	51	Yes Centroid
6	ZINC000827360794<br>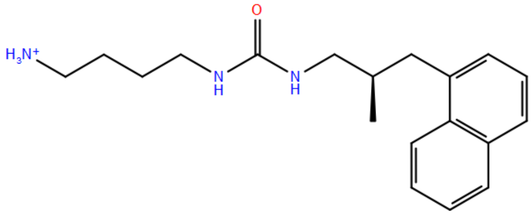<br>C[C@@H](CNC(=O)NCCCCN)Cc1cccc2ccccc12	-9.123 kcal/mol	0	31	Yes Centroid
7	ZINC000035373220<br>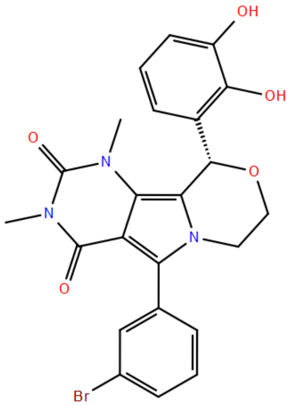<br>Cn1c(=O)c2c(-c3cccc(Br)c3)n3c(c2n(C)c1=O)[C@H](c1cccc(O)c1O)OCC3	-9.107 kcal/mol	0	39	Yes Centroid
8	ZINC000072430969<br>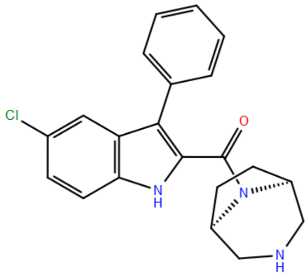<br>O=C(c1[nH]c2ccc(Cl)cc2c1-c1ccccc1)N1[C@H]2CC[C@@H]1CNC2	-9.068 kcal/mol	1	45	Yes Centroid

9	ZINC000078648574 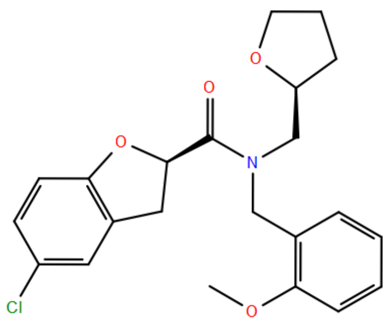 COc1ccccc1CN(C[C@@H]1CCCO1)C(=O)[C@H]1Cc2cc(Cl)ccc2O1	-9.051 kcal/mol	1	34	Yes Centroid
10	ZINC000005344596 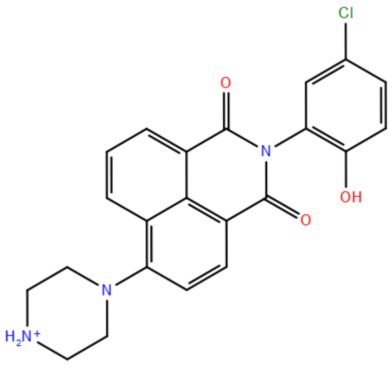 O=C1c2cccc3c(N4CCNCC4)ccc(c23)C(=O)N1c1cc(Cl)ccc1O	-9.029 kcal/mol	0	33	Yes Centroid
11	ZINC000069348668 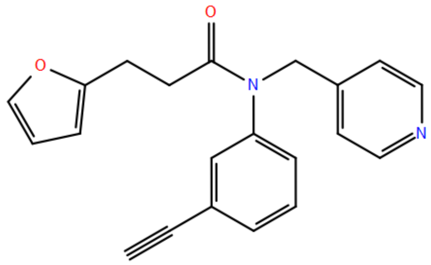 C#Cc1cccc(N(Cc2ccncc2)C(=O)CCc2ccco2)c1	-8.971 kcal/mol	0	44	Yes Centroid

Figure S4. Top 11 ZINC compounds for the first representative conformation of the DOR MD simulation structure conformation including the crystal reference compound with ZINC ID, structure, SMILE code, docking score, number of STARs (indicator for "drug-likeness"), cluster IDs (ligand similarity clustering based on Canvas), and centroid (measures distances to the arithmetic means of clusters).

No.	Zinc ID/Structure/SMILE code	Docking score	# STAR	# Cluster ID	Centroid
Ref. Comp	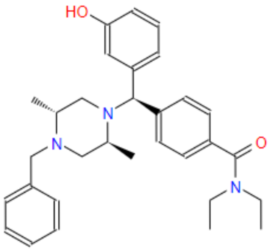	-8.635 kcal/mol			
1	ZINC000057999653 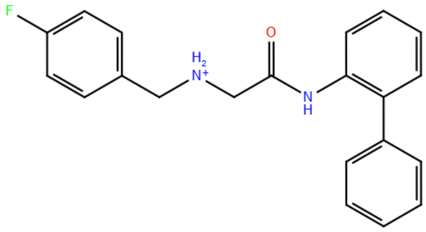 O=C(CNCc1ccc(F)cc1)Nc1ccccc1-c1ccccc1	-10.158 kcal/mol	1	35	Yes Centroid
2	ZINC000002877267 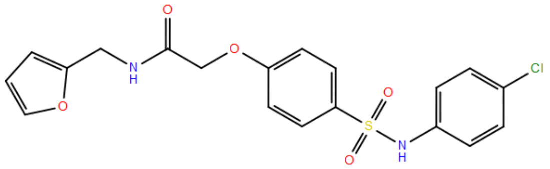 O=C(COc1ccc(S(=O)(=O)Nc2ccc(Cl)cc2)cc1)NCc1ccco1	-9.881 kcal/mol	0	24	Yes Centroid
3	ZINC000408693879 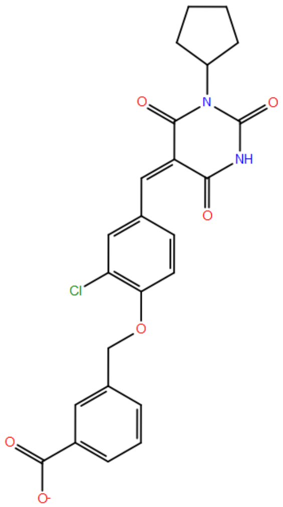 O=C1NC(=O)N(C2CCCC2)C(=O)/C1=C/c1ccc(OCc2cccc(C(=O)O)c2)c(Cl)c1	-9.791 kcal/mol	0	30	Yes Centroid
4	ZINC000000880008 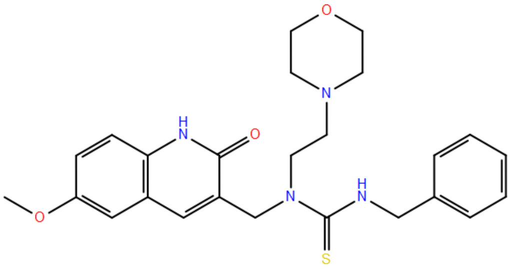 COc1ccc2[nH]c(=O)c(CN(CCN3CCOCC3)C(=S)NCc3ccccc3)cc2c1	-9.785 kcal/mol	0	23	Yes Centroid

5	ZINC000006664413 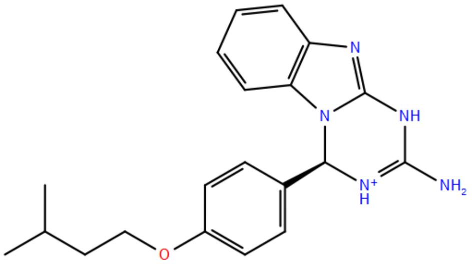 CC(C)CCOc1ccc([C@@H]2N=C(N)Nc3nc4ccccc4n32)cc1	-9.770 kcal/mol	0	8	Yes Centroid
6	ZINC000001408226 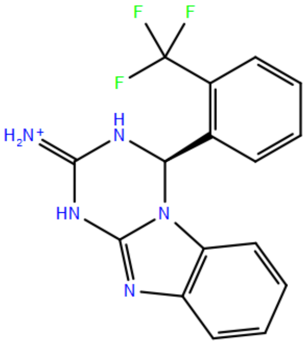 N=C1Nc2nc3ccccc3n2[C@H](c2ccccc2C(F)(F)F)N1	-9.746 kcal/mol	0	36	Yes Centroid
7	ZINC000001045477 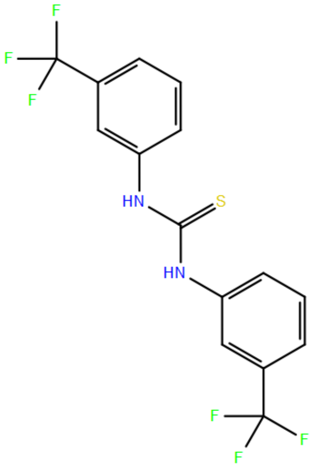 FC(F)(F)c1cccc(NC(=S)Nc2cccc(C(F)(F)F)c2)c1	-9.709 kcal/mol	1	47	Yes Centroid
8	ZINC000005493735 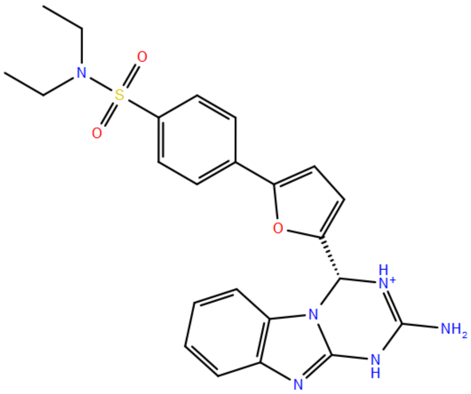 CCN(CC)S(=O)(=O)c1ccc(c2ccc([C@@H]3NC(N)=Nc4nc5ccccc5n43)o2)cc1	-9.683 kcal/mol	0	7	Yes Centroid

9	ZINC000001641702 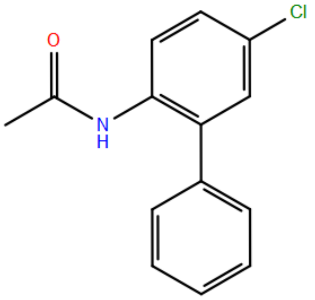 CC(=O)Nc1ccc(Cl)cc1-c1ccccc1	-9.605 kcal/mol	1	15	Yes Centroid
10	ZINC000000302628 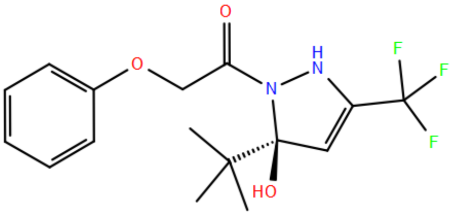 CC(C)(C)[C@@]1(O)CC(C(F)(F)F)=NN1C(=O)COc1ccccc1	-9.543 kcal/mol	0	29	Yes Centroid
11	ZINC000095418373 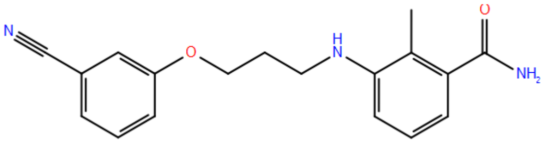 Cc1c(NCCCOc2cccc(C#N)c2)cccc1C(N)=O	-9.363 kcal/mol	0	40	Yes Centroid
12	ZINC000006750553 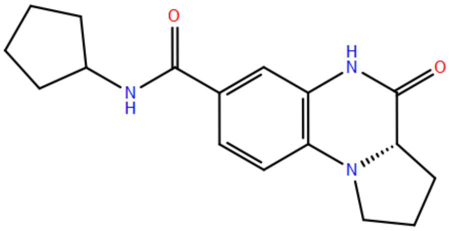 O=C(NC1CCCC1)c1ccc2c(c1)NC(=O)[C@@H]1CCCN21	-9.347 kcal/mol	0	16	Yes Centroid
13	ZINC000097002851 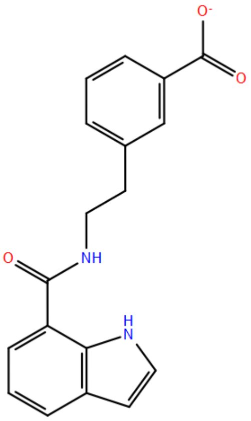 O=C(O)c1cccc(CCNC(=O)c2cccc3cc[nH]c23)c1	-9.314 kcal/mol	0	12	Yes Centroid

14	ZINC000005776998<br>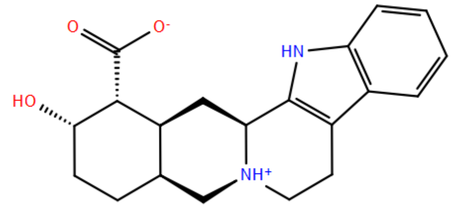<br>O=C(O)[C@H]1[C@@H](O)CC[C@@H]2CN3CCc4c([nH]c5ccccc45)[C@@H]3C[C@@H]21	-9.306 kcal/mol	0	19	Yes Centroid
15	ZINC000004663101<br>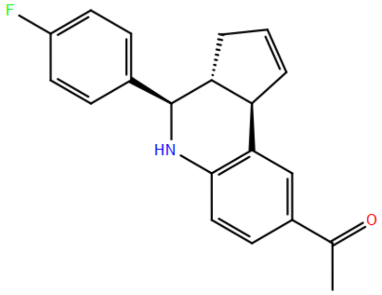<br>CC(=O)c1ccc2c(c1)[C@@H]1C=CC[C@H]1[C@H](c1ccc(F)cc1)N2	-9.292 kcal/mol	0	22	Yes Centroid
16	ZINC000005604766<br>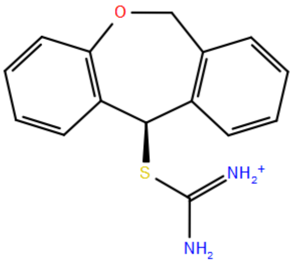<br>N=C(N)S[C@@H]1c2ccccc2COc2ccccc21	-9.271 kcal/mol	0	10	Yes Centroid
17	ZINC000225173433<br>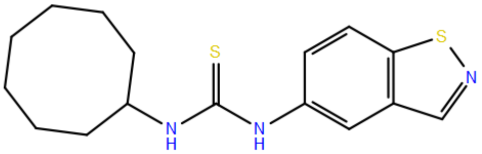<br>S=C(Nc1ccc2sncc2c1)NC1CCCCCCC1	-9.167 kcal/mol	1	48	Yes Centroid
18	ZINC000034720963<br>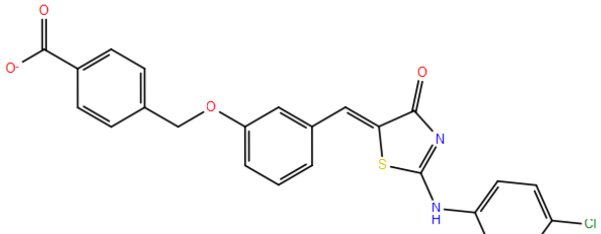<br>O=C1N=C(Nc2ccc(Cl)cc2)S/C1=C\c1cccc(OCc2ccc(C(=O)O)cc2)c1	-9.117 kcal/mol	1	31	Yes Centroid

19	ZINC000409066936<br>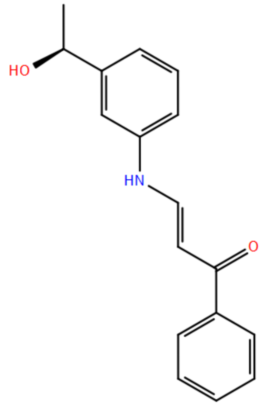<br>C[C@H](O)c1cccc(N/C=C/C(=O)c2ccccc2)c1	-9.115 kcal/mol	0	39	Yes Centroid
20	ZINC000014750115<br>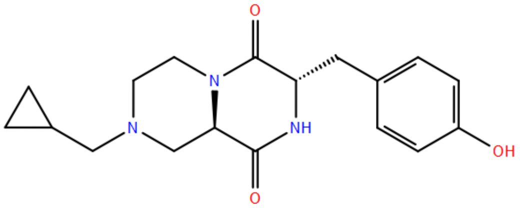<br>O=C1N[C@@H](Cc2ccc(O)cc2)C(=O)N2CCN(CC3CC3)C[C@H]12	-9.012 kcal/mol	0	34	Yes Centroid
21	ZINC000059677349<br>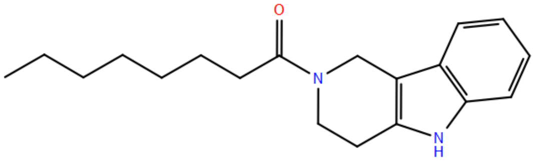<br>CCCCCCCC(=O)N1CCc2[nH]c3ccccc3c2C1	-8.999 kcal/mol	0	18	Yes Centroid
22	ZINC000002690402<br>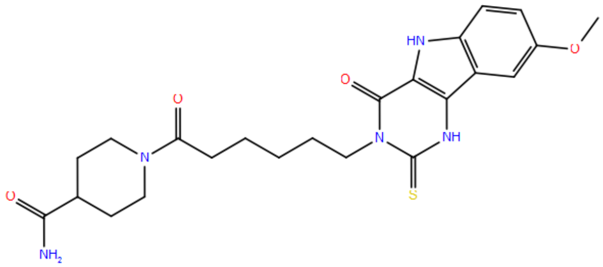<br>COc1ccc2[nH]c3c(=O)n(CCCCCC(=O)N4CCC(C(N)=O)CC4)c(=S)[nH]c3c2c1	-8.998 kcal/mol	0	33	Yes Centroid
23	ZINC000020572602<br>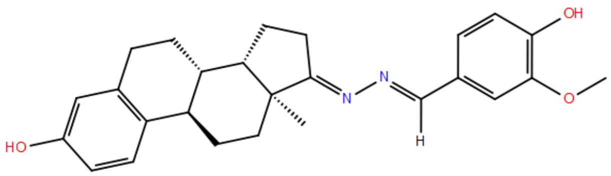<br>COc1cc(/C=N/N=C2\CC[C@H]3[C@H]4CCc5cc(O)ccc5[C@@H]4CC[C@]23C)ccc1O	-8.963 kcal/mol	1	32	Yes Centroid

24	ZINC000408729576 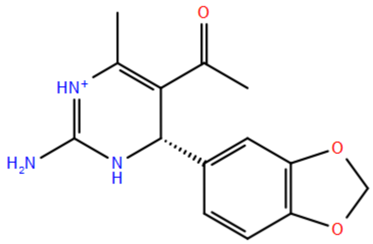 CC(=O)C1=C(C)NC(=N)N[C@H]1c1ccc2c(c1)OCO2	-8.940 kcal/mol	0	51	Yes Centroid
25	ZINC000009660785 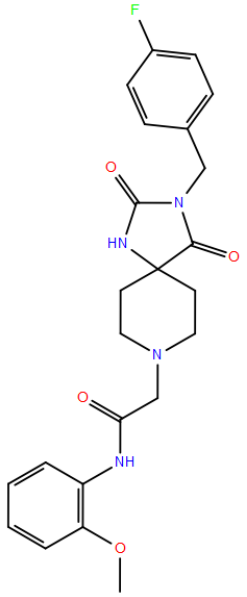 COc1ccccc1NC(=O)CN1CCC2(CC1)NC(=O)N(Cc1ccc(F)cc1)C2=O	-8.925 kcal/mol	0	21	Yes Centroid
26	ZINC000334160398 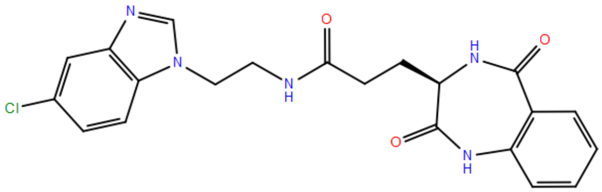 O=C(CC[C@H]1NC(=O)c2ccccc2NC1=O)NCCn1cnc2cc(Cl)ccc21	-8.824 kcal/mol	0	26	Yes Centroid

Figure S5. Top 26 ZINC compounds for the second representative conformation of the DOR MD simulation structure conformation including the crystal reference compound with ZINC ID, structure, SMILE code, docking score, number of STARs (indicator for "drug-likeness"), cluster IDs (ligand similarity clustering based on Canvas), and centroid (measures distances to the arithmetic means of clusters).

	Crystal and Docked Complexes	Crystal and Docked Ligand Structure	Ligand 2D Chemical Structure
Crystal ligand	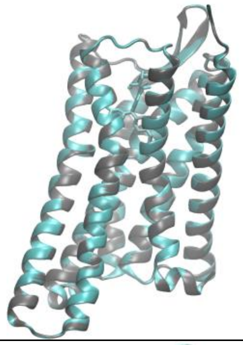	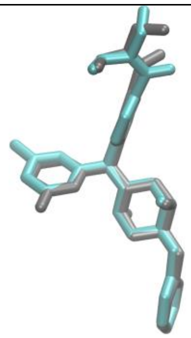	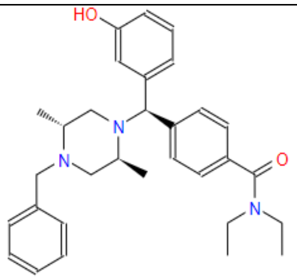
ZINC000020559278	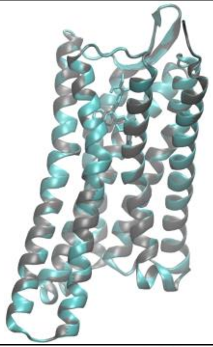	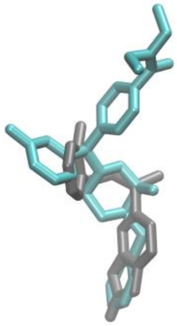	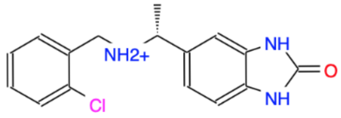
ZINC000014242201	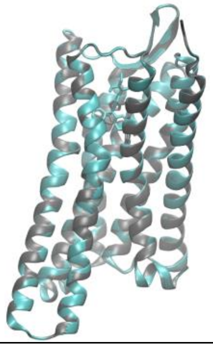	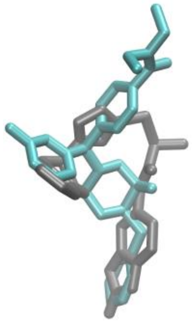	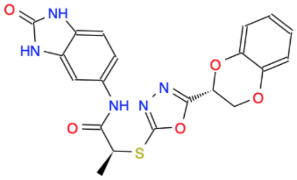
ZINC000562639987	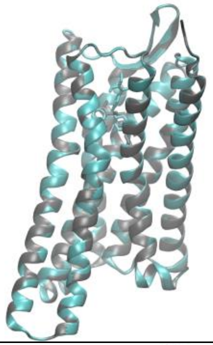	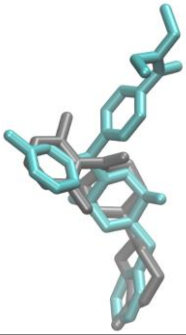	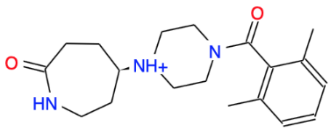
ZINC000071763967	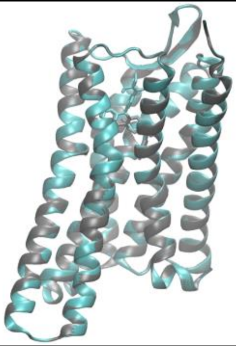	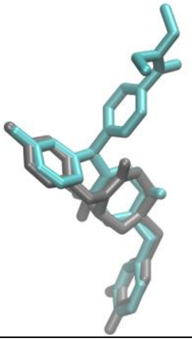	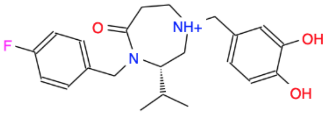

ZINC000067947687	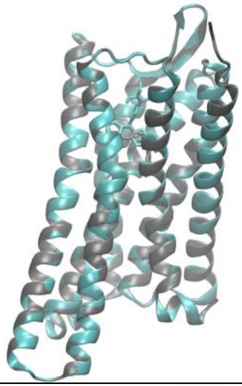	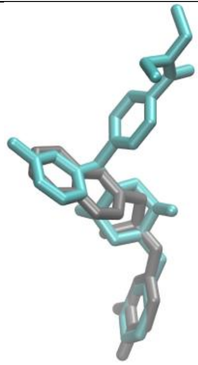	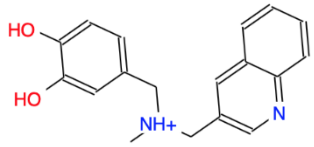
ZINC000634950507	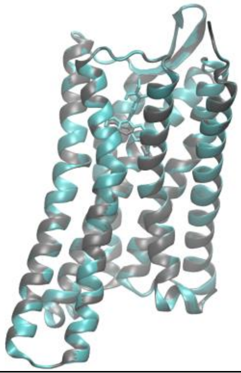	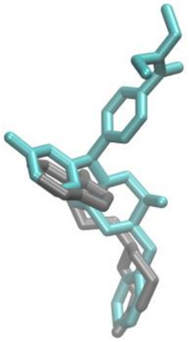	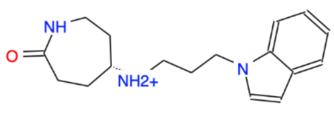
ZINC000072227493	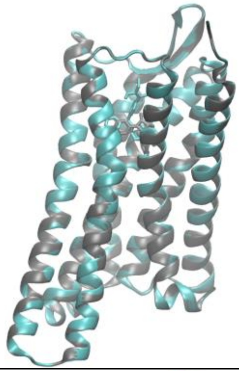	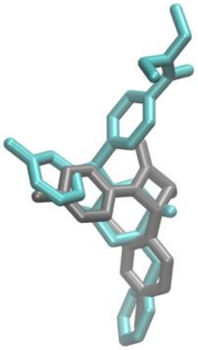	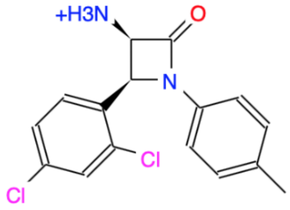
ZINC000257262766	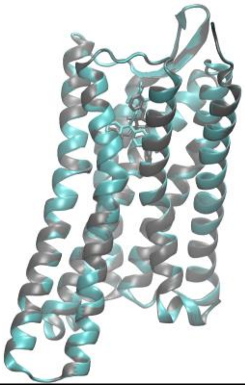	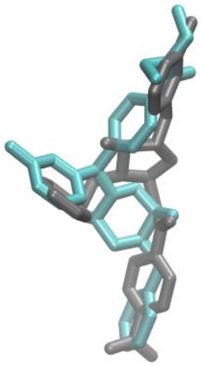	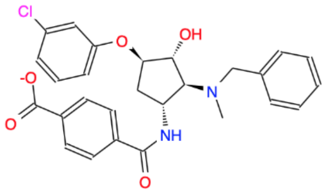

ZINC000006645700	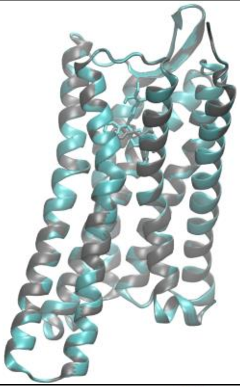	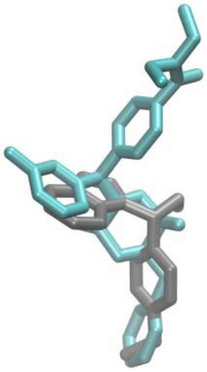	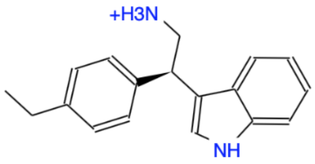
ZINC000082157638	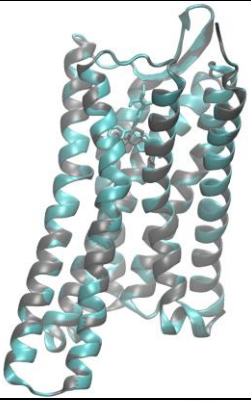	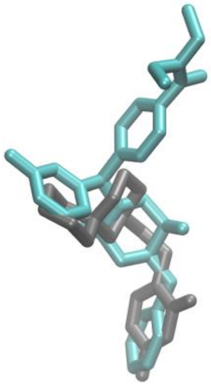	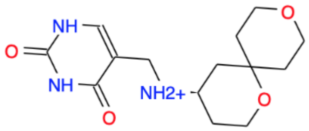
ZINC000091782363	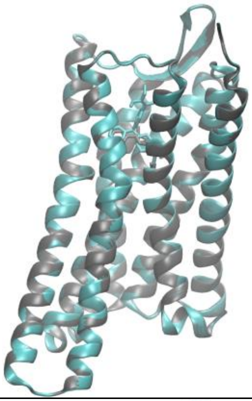	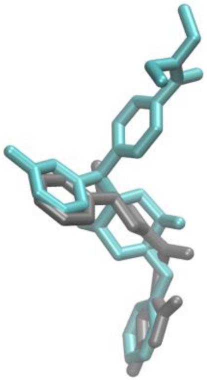	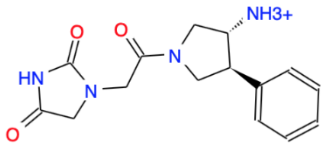
ZINC000091595762	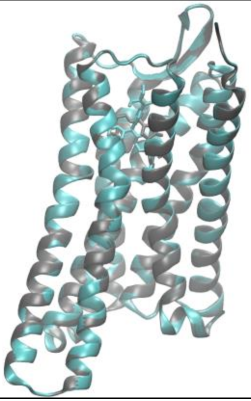	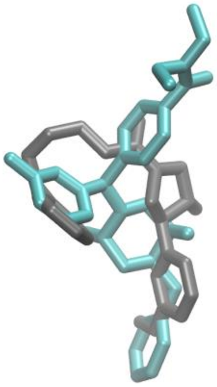	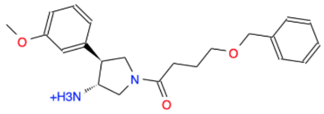

ZINC000067558896	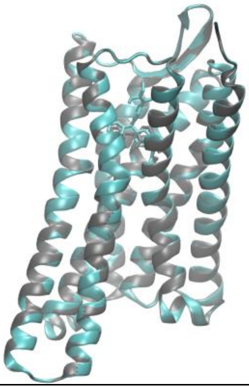	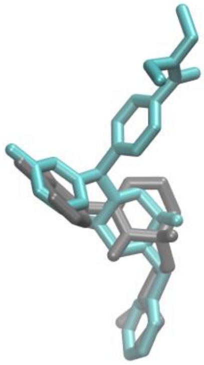	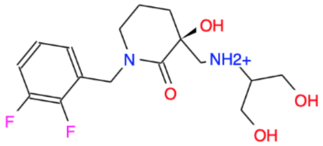
ZINC000757213857	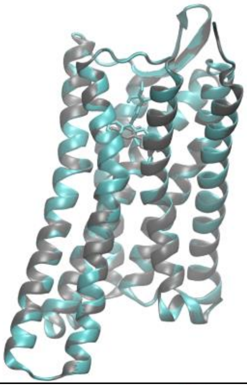	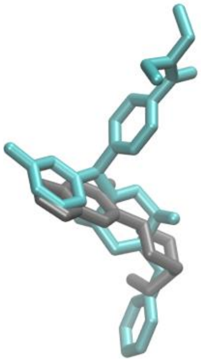	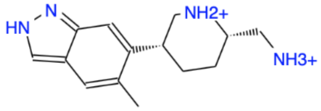
ZINC000218867772	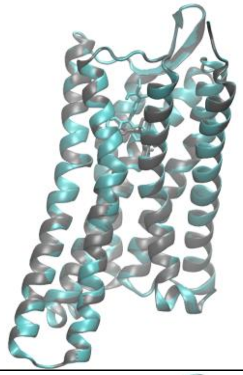	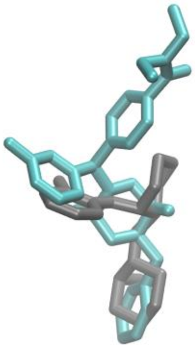	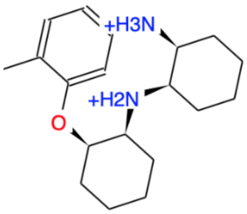
ZINC000013637398	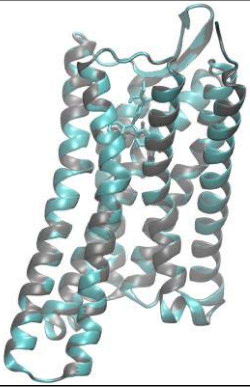	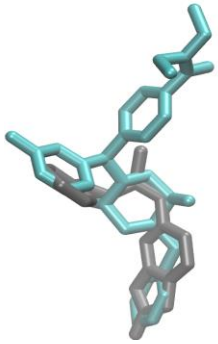	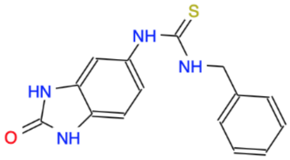

<table>
<tr><td>ZINC000078515864</td><td>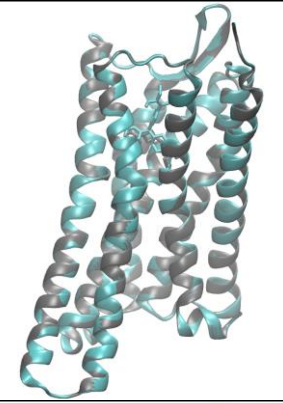</td><td>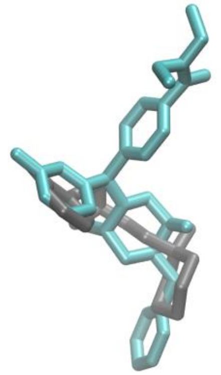</td><td>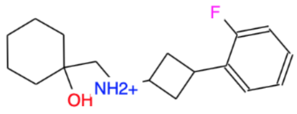</td></tr>
<tr><td>ZINC000014538415</td><td>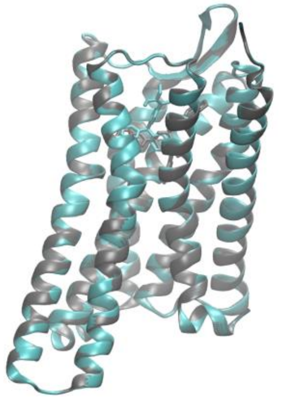</td><td>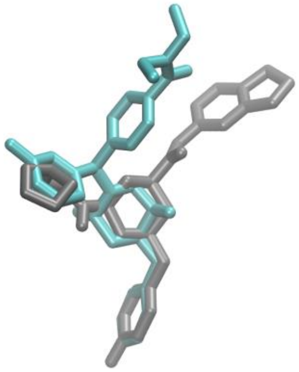</td><td>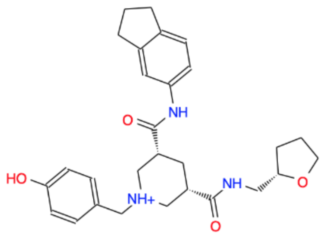</td></tr>
<tr><td>ZINC000218873420</td><td>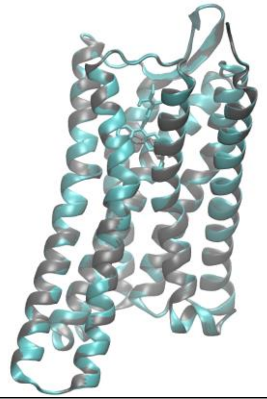</td><td>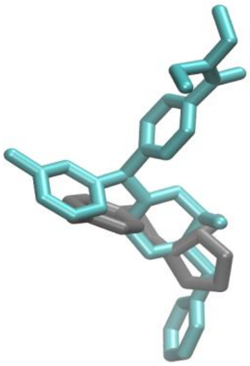</td><td>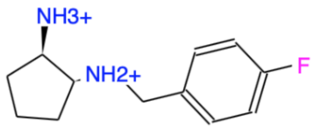</td></tr>
<tr><td>ZINC000001743629</td><td>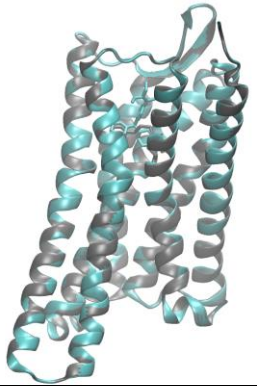</td><td>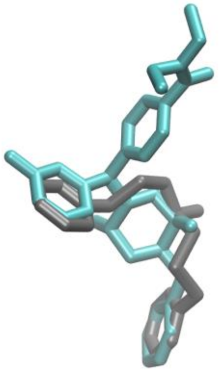</td><td>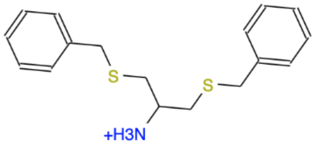</td></tr>
</table>

ZINC000028295259	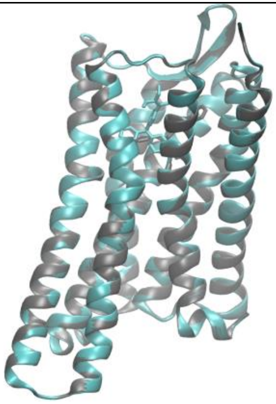	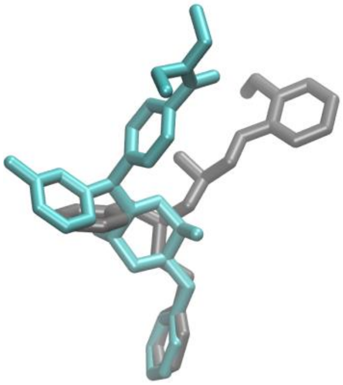	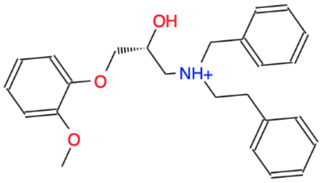
ZINC000014536536	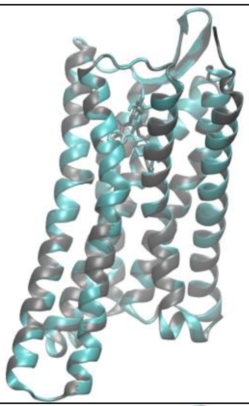	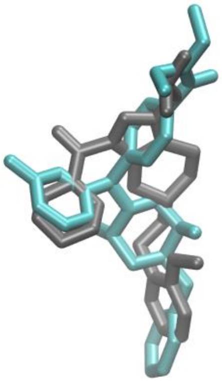	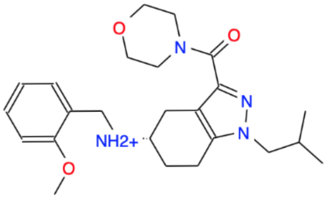
ZINC000147095213	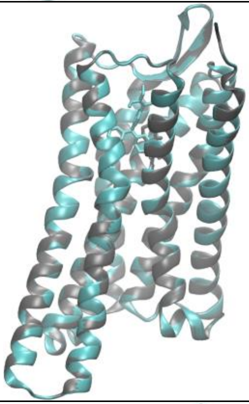	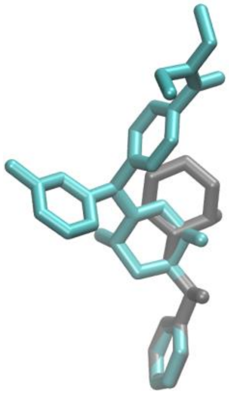	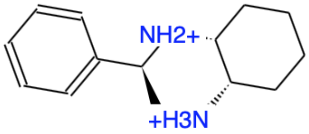
ZINC000095698957	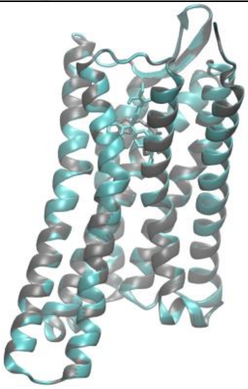	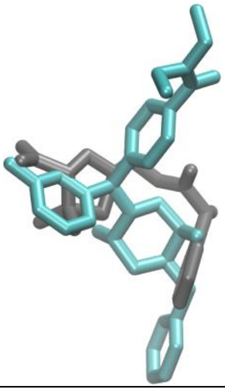	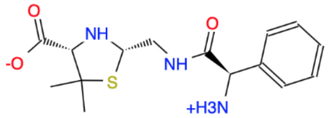

ZINC000014888594	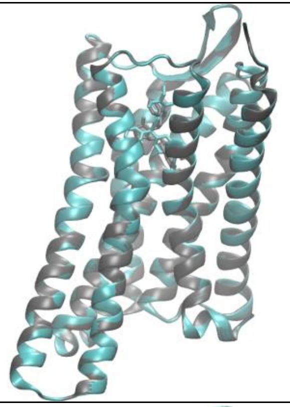	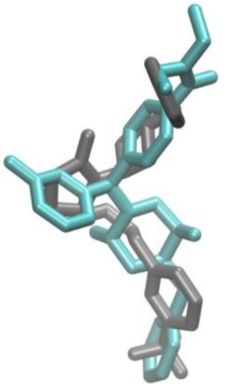	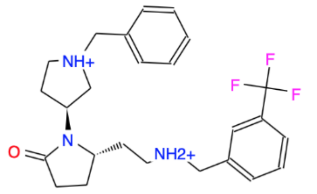
ZINC000015080658	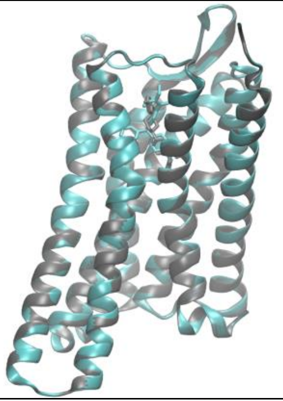	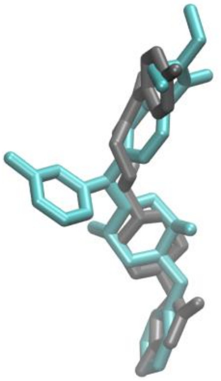	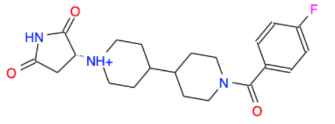
ZINC000096027748	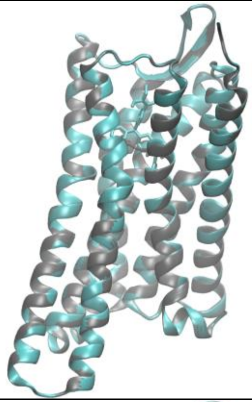	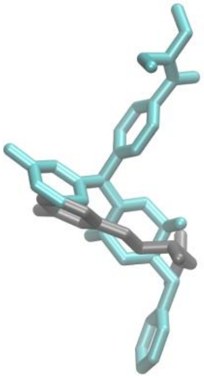	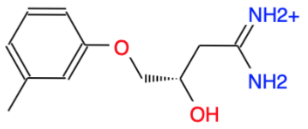
ZINC000067675691	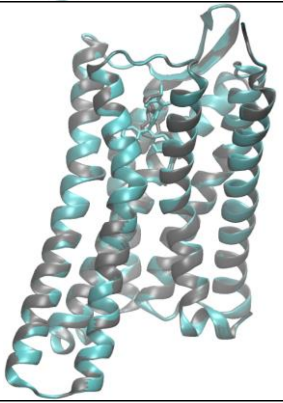	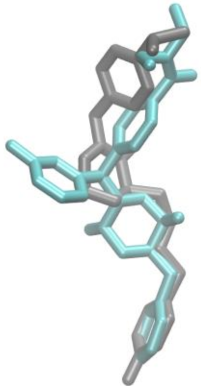	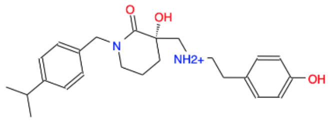

ZINC000011691661

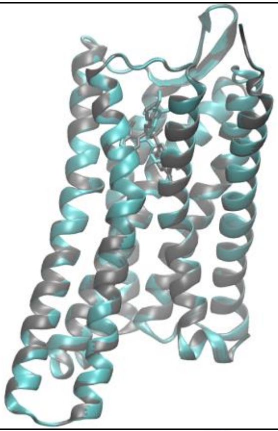

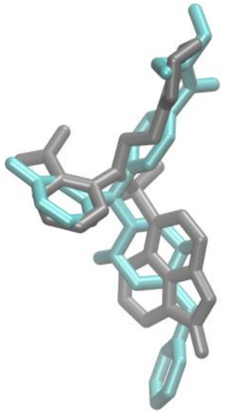

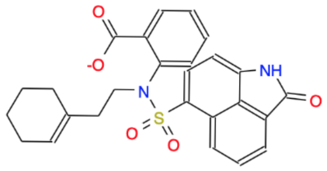

ZINC000096312018

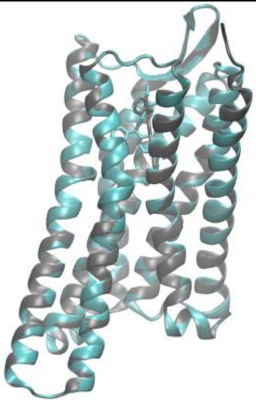

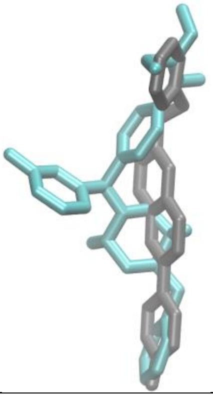

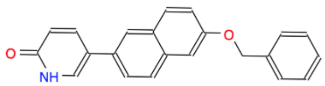

ZINC000248261142

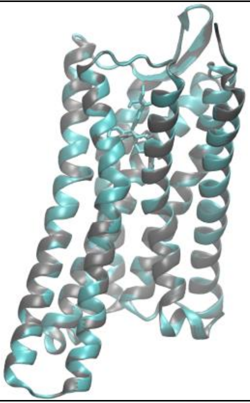

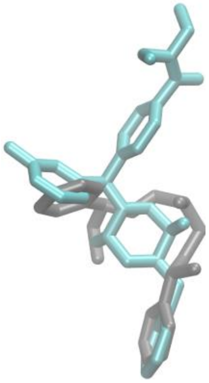

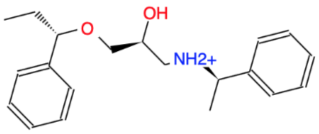

ZINC000036160706

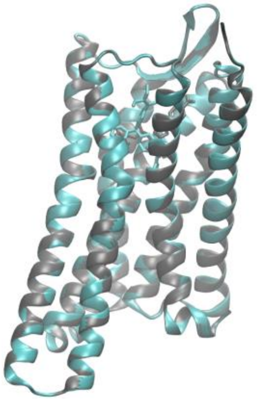

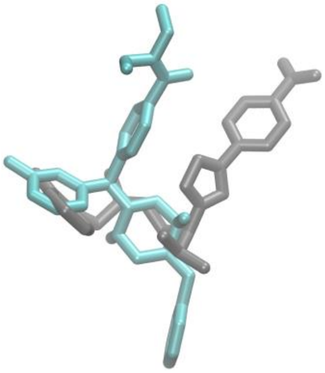

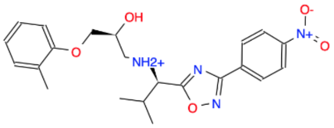

Figure S6. Comparison between the DOR crystal structure (PDB ID: 6PT3) (cyan) and the docked complex of the top 32 ZINC compounds (gray) in the side view and ligand view with the 2D chemical structure of the ZINC compounds.

	Crystal and Docked Complexes	Crystal and Docked Ligand Structure	Ligand 2D Chemical Structure
ZINC000002532984	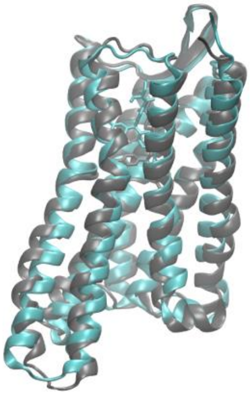	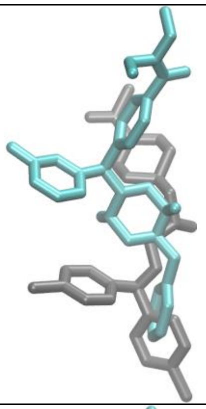	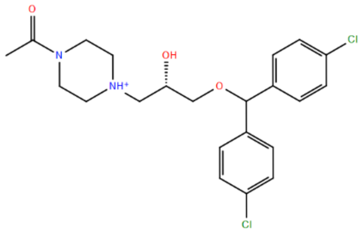
ZINC000037556415	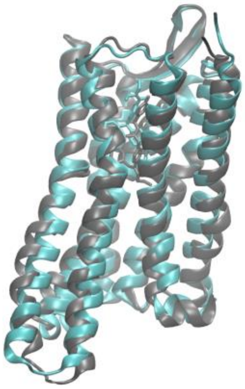	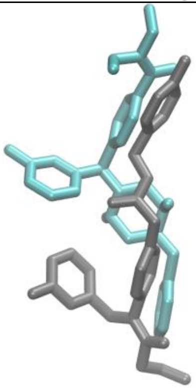	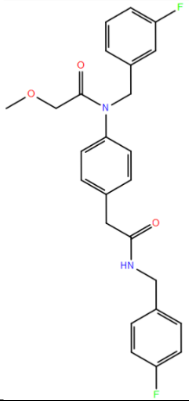
ZINC000095456365	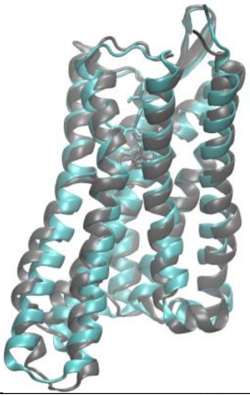	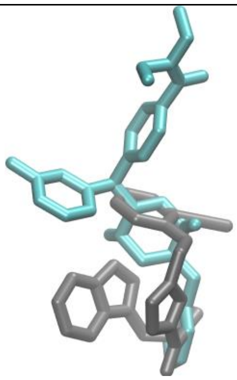	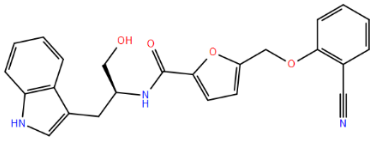
ZINC000225318193	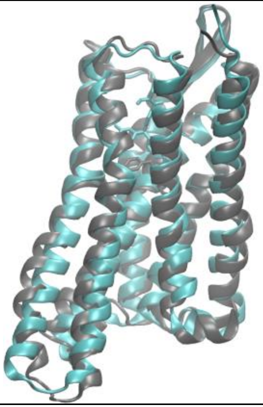	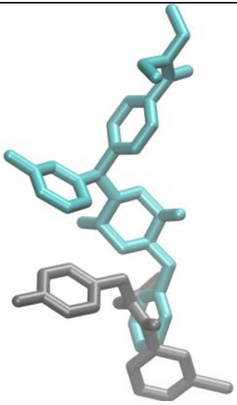	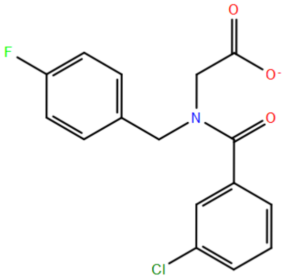

ZINC000299770618	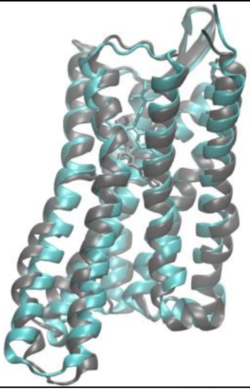	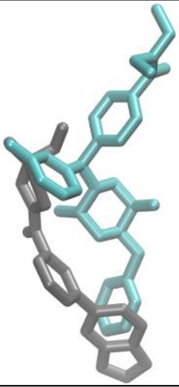	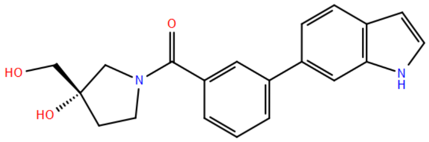
ZINC000827360794	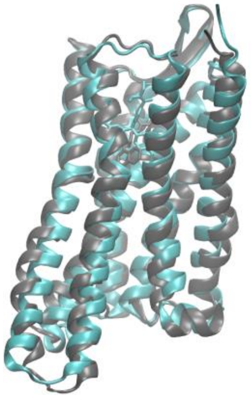	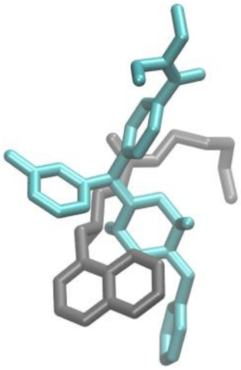	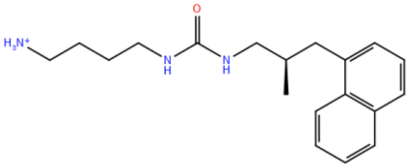
ZINC000035373220	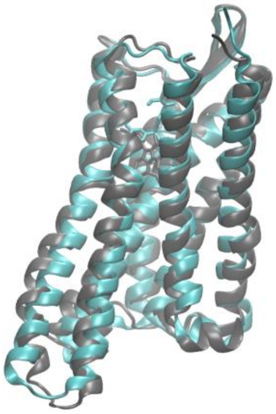	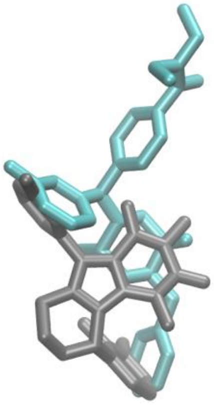	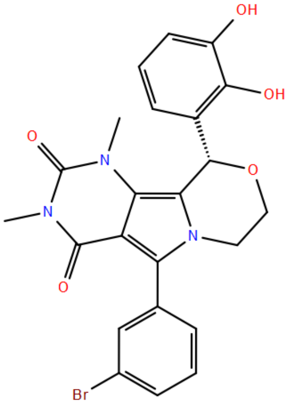
ZINC000072430969	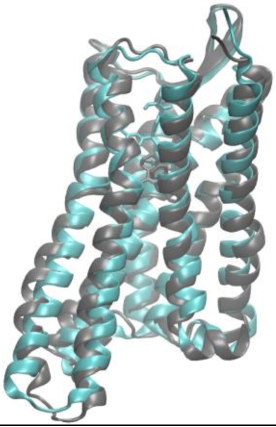	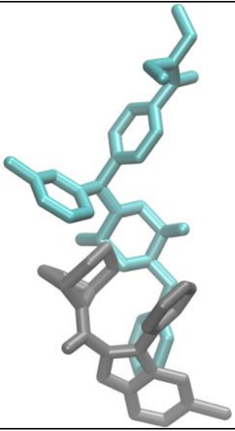	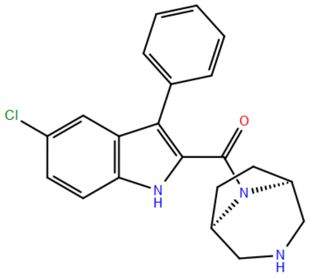

ZINC000078648574	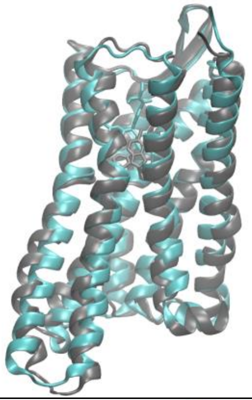	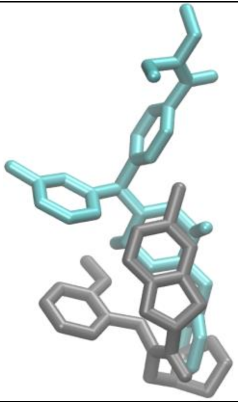	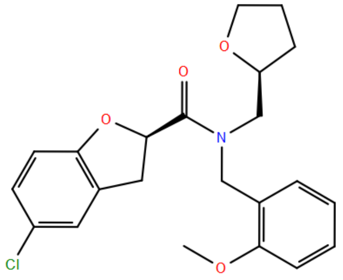
ZINC000005344596	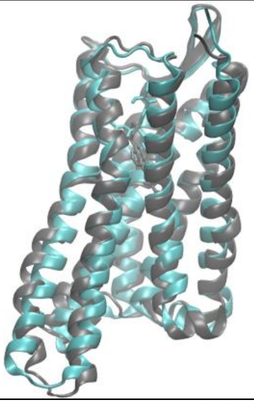	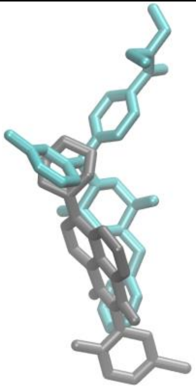	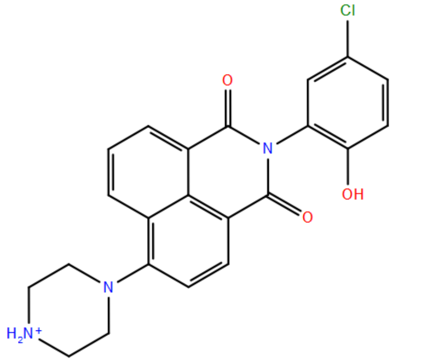
ZINC000069348668	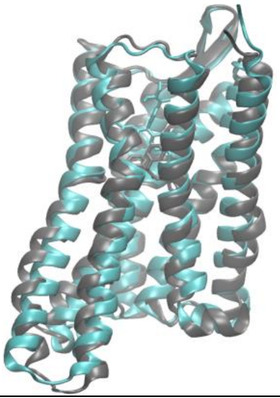	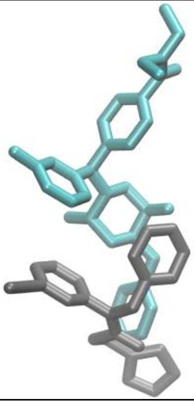	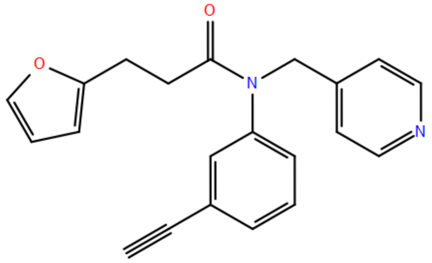

Figure S7. Comparison between the DOR crystal structure (PDB ID: 6PT3) (cyan) and the docked complex of the top 11 ZINC compounds (gray) of the first representative structure in the side view and ligand view with the 2D chemical structure of the ZINC compounds.

	Crystal and Docked Complexes	Crystal and Docked Ligand Structure	Ligand 2D Chemical Structure
ZINC000057999653	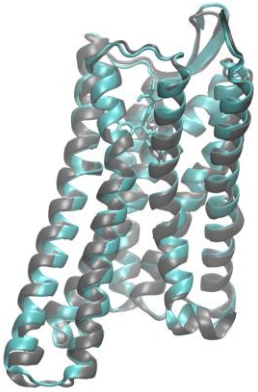	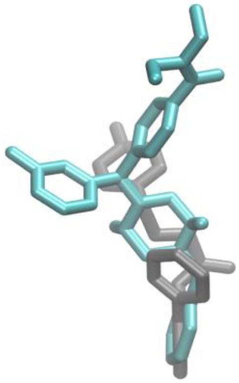	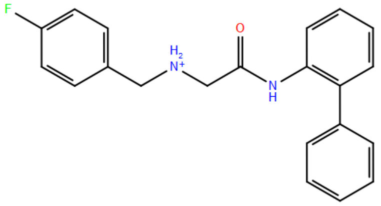
ZINC000002877267	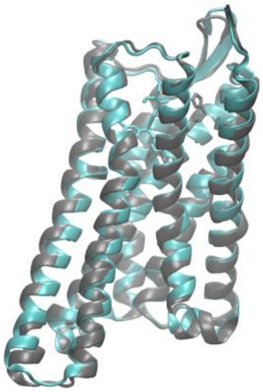	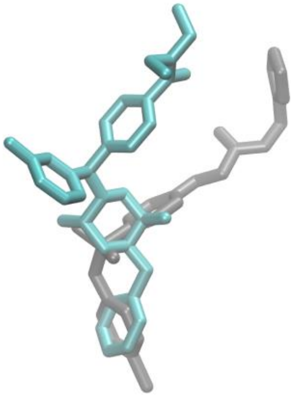	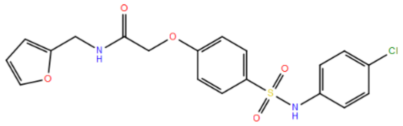
ZINC000408693879	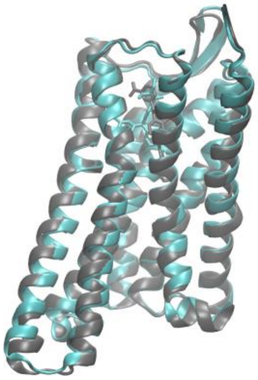	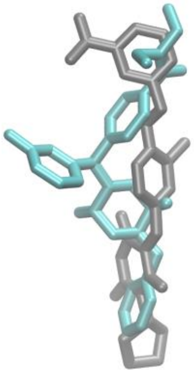	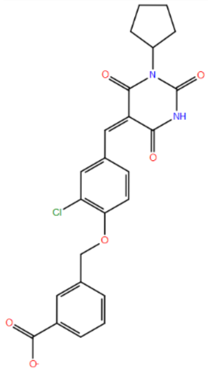
ZINC000000080008	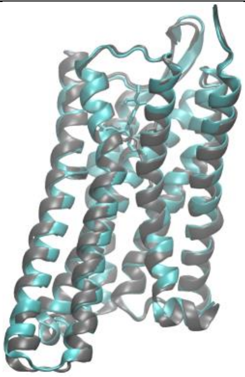	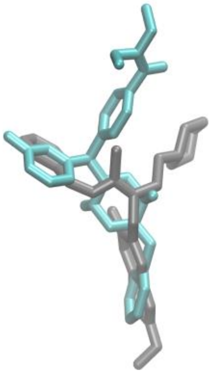	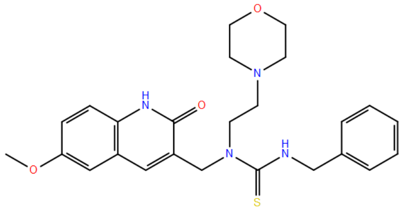

ZINC000006664413	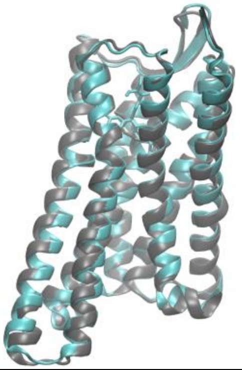	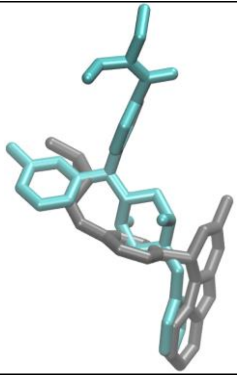	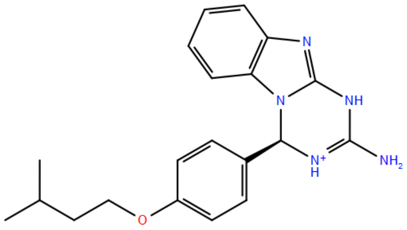
ZINC000001408226	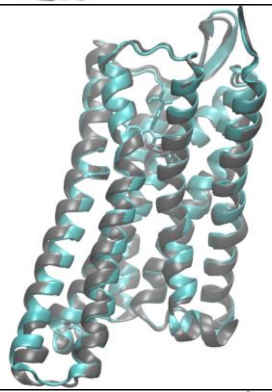	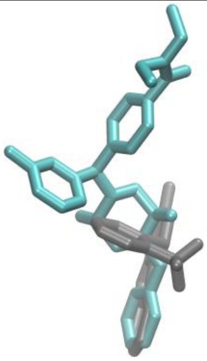	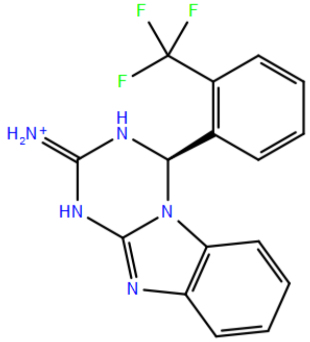
ZINC000001045477	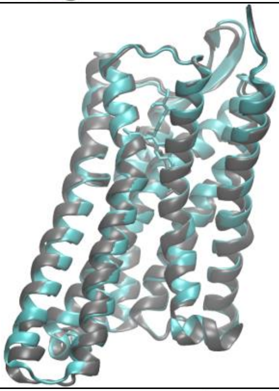	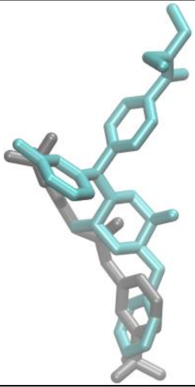	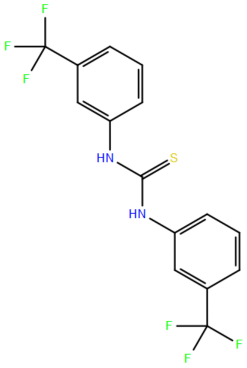
ZINC000005493735	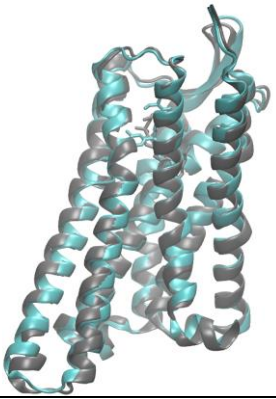	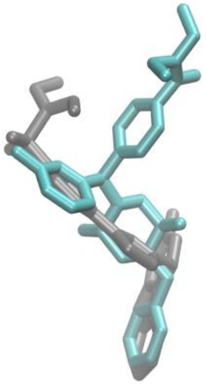	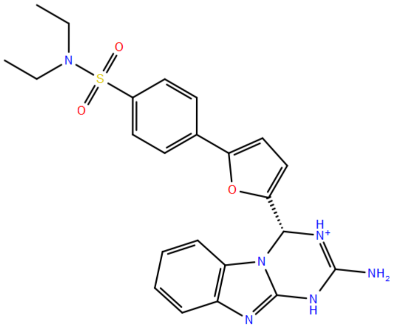

ZINC000001641702	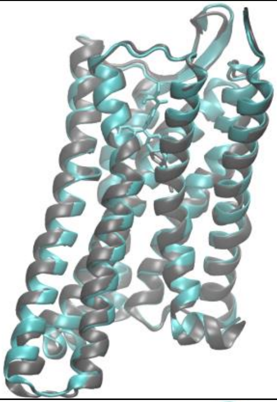	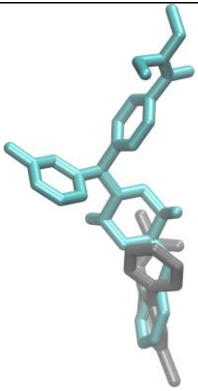	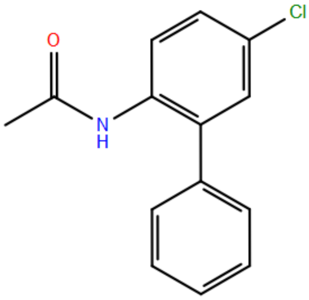
ZINC000000302628	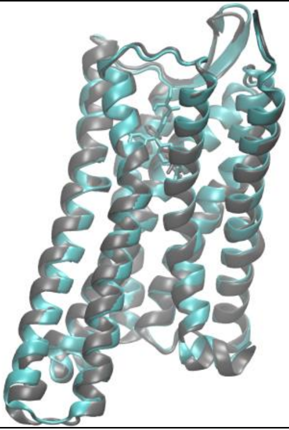	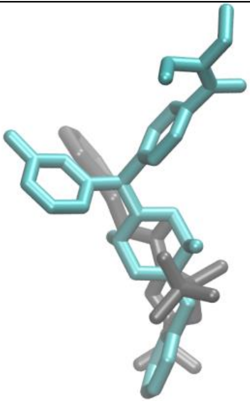	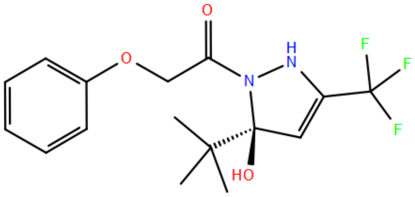
ZINC000095418373	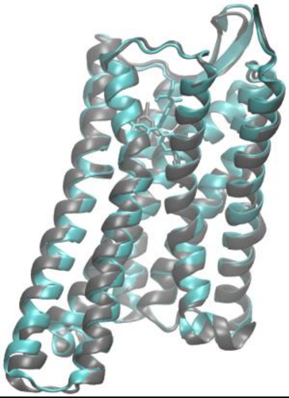	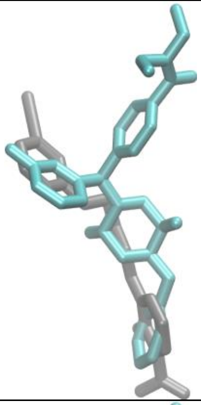	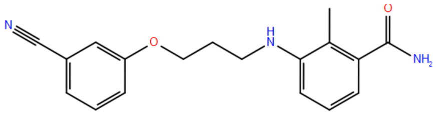
ZINC000006750553	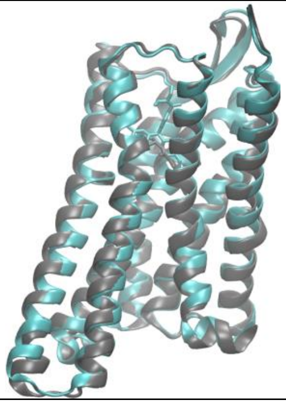	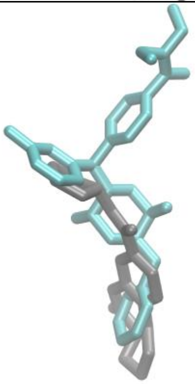	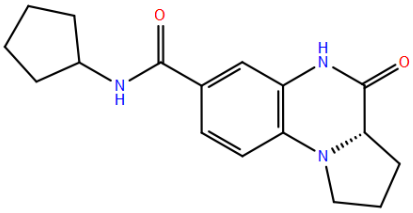

ZINC000097002851	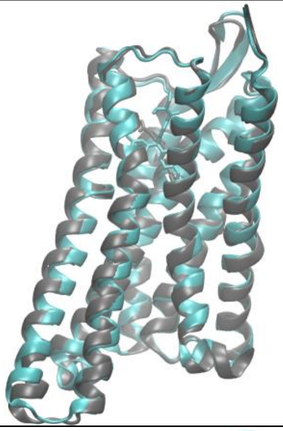	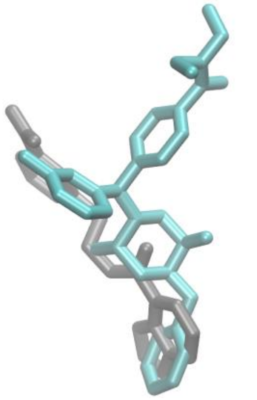	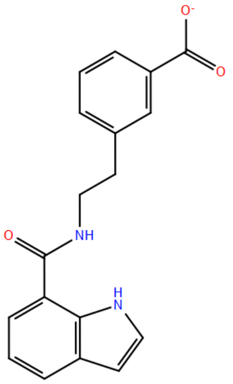
ZINC000005776998	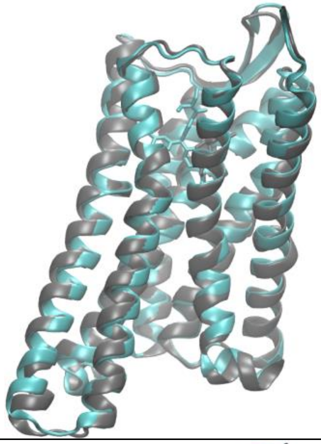	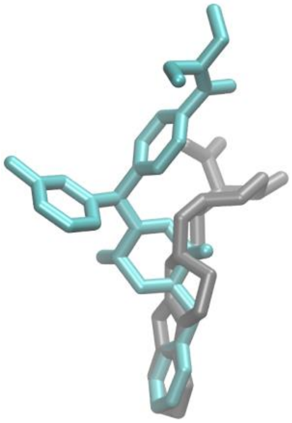	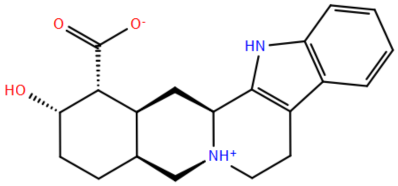
ZINC000004663101	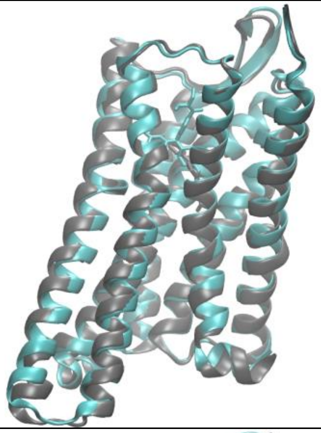	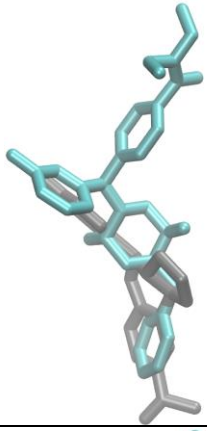	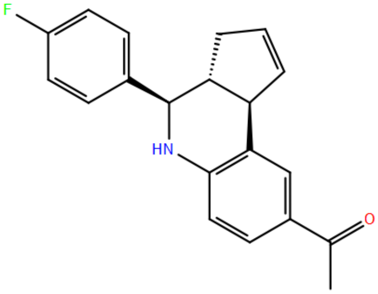
ZINC000005604766	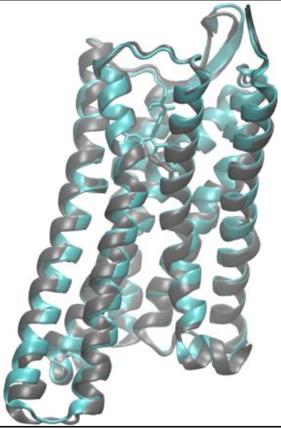	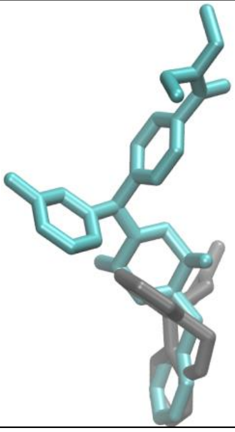	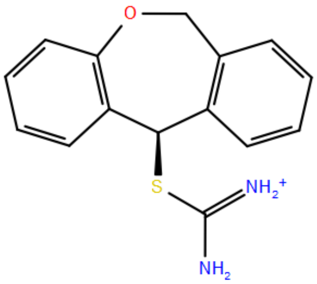

ZINC000225173433	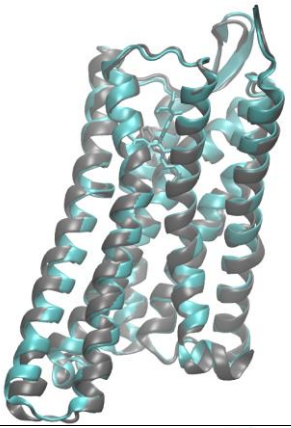	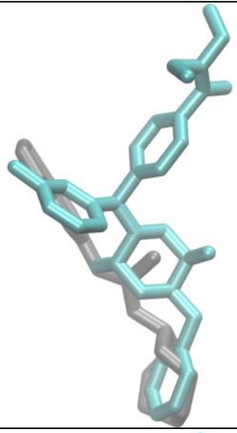	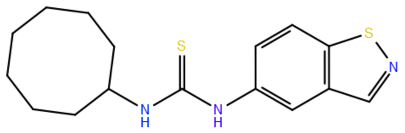
ZINC000034720963	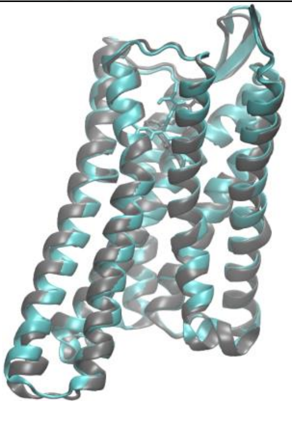	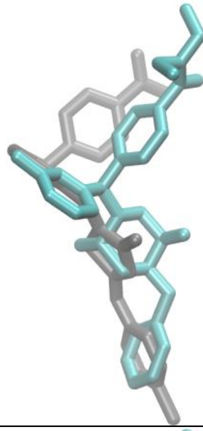	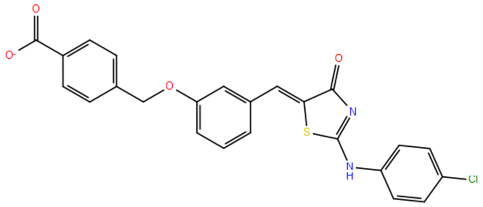
ZINC000409066936	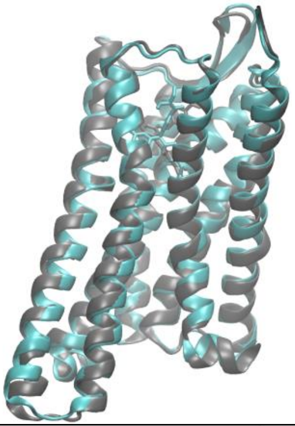	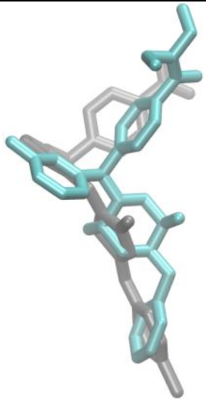	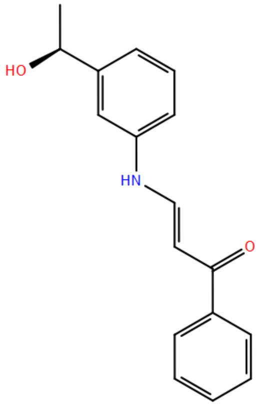
ZINC000014750115	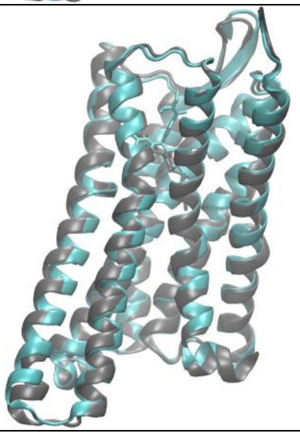	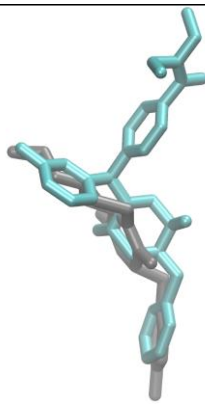	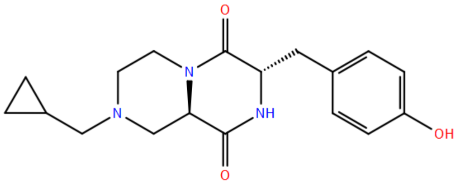

ZINC000059677349	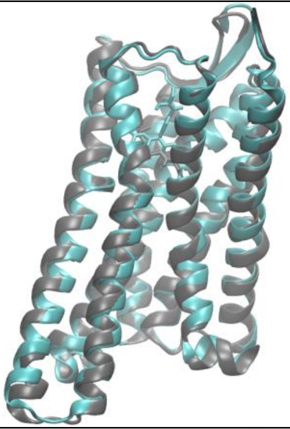	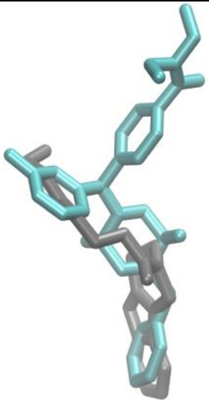	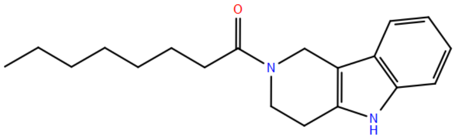
ZINC000002690402	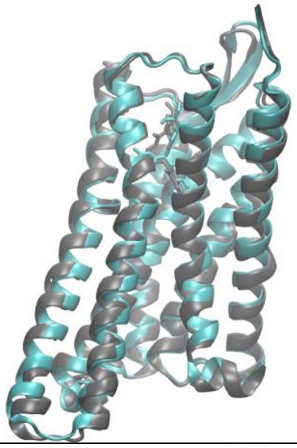	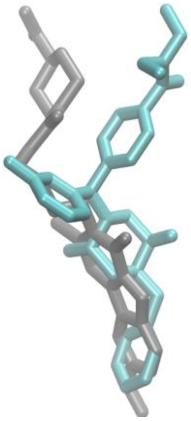	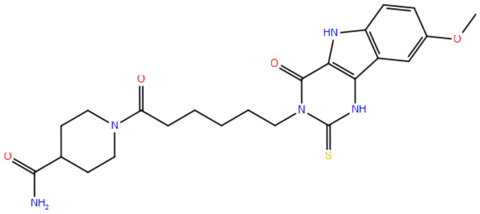
ZINC000020572602	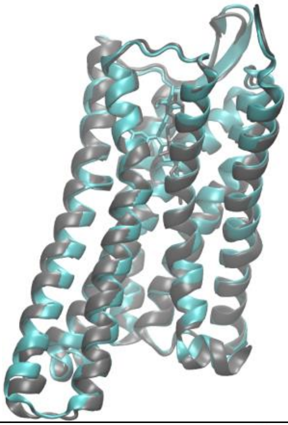	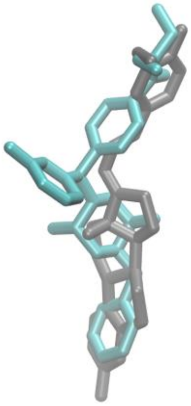	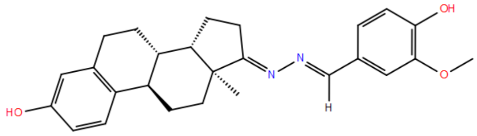
ZINC000408729576	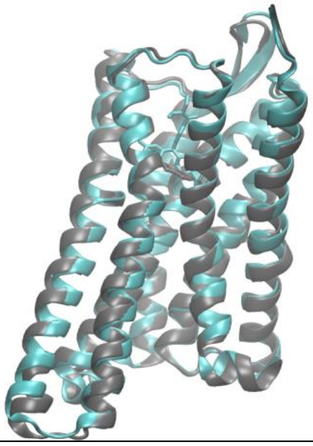	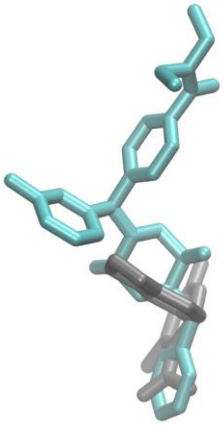	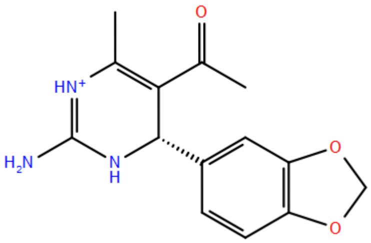

ZINC000009660785	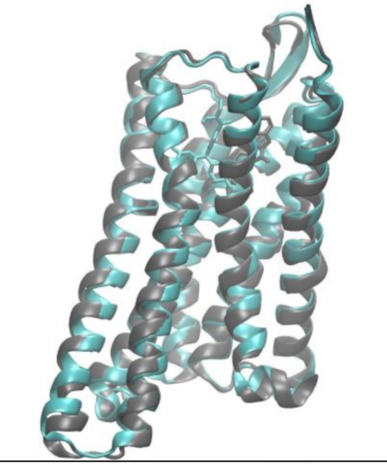	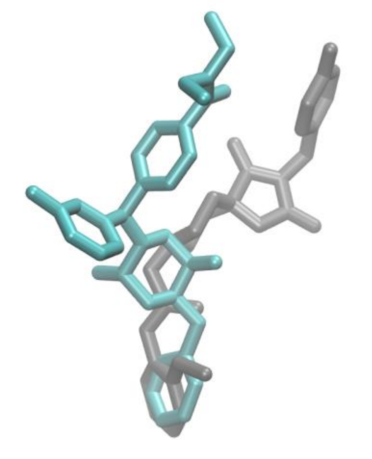	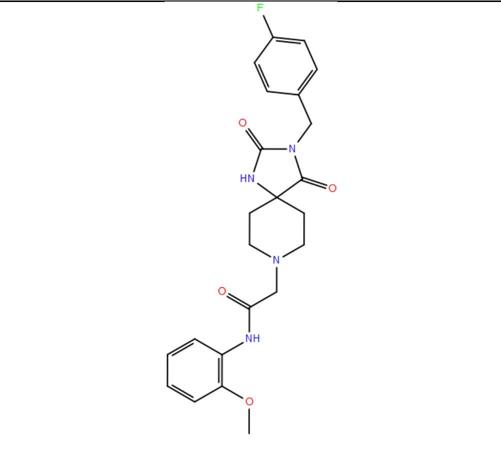
ZINC000334160398	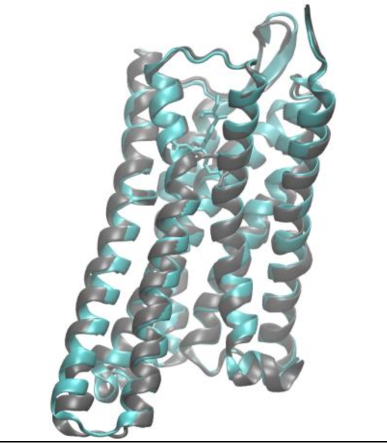	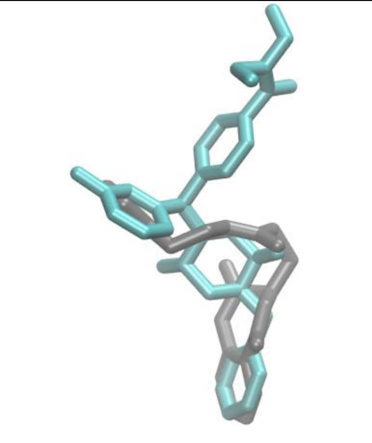	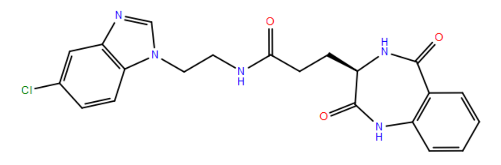

Figure S8. Comparison between the DOR crystal structure (PDB ID: 6PT3) (cyan) and the docked complex of the top 26 ZINC compounds (gray) of the second representative structure in the side view and ligand view with the 2D chemical structure of the ZINC compounds.

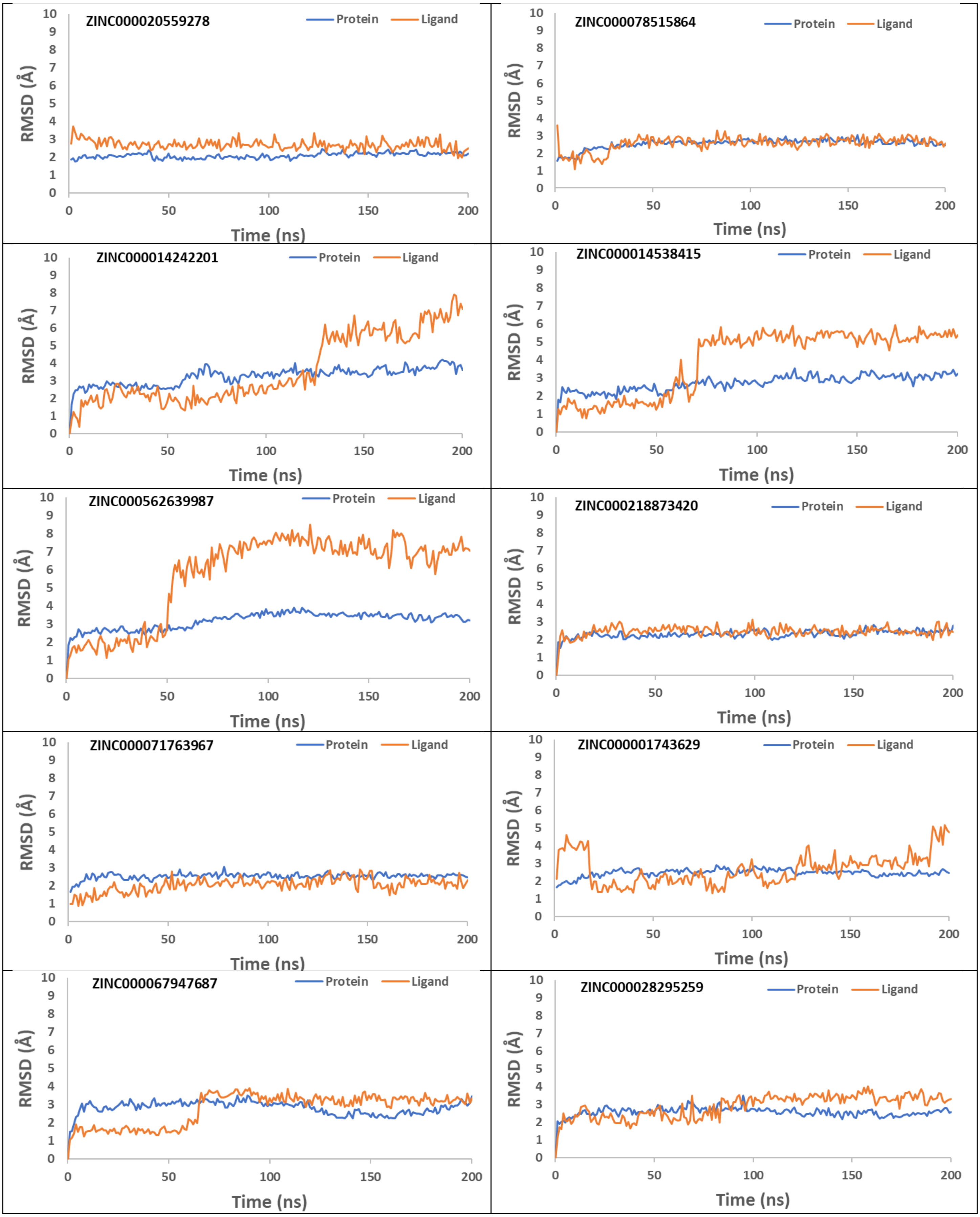

ZINC000020559278
ZINC000078515864
ZINC000014242201
ZINC000014538415
ZINC000562639987
ZINC000218873420
ZINC000071763967
ZINC000001743629
ZINC000067947687
ZINC000028295259
Protein
Ligand
RMSD (Å)
Time (ns)

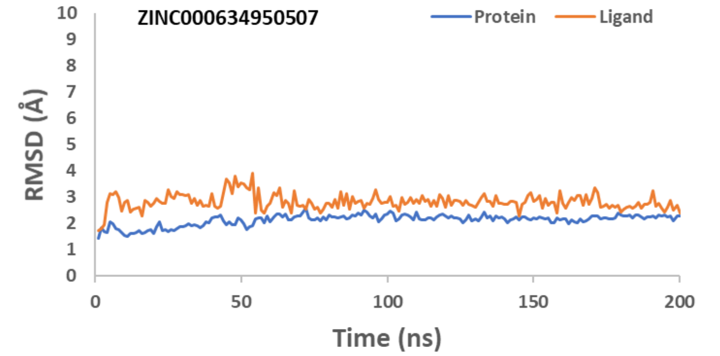
ZINC000634950507
Protein
Ligand
RMSD (Å)
Time (ns)

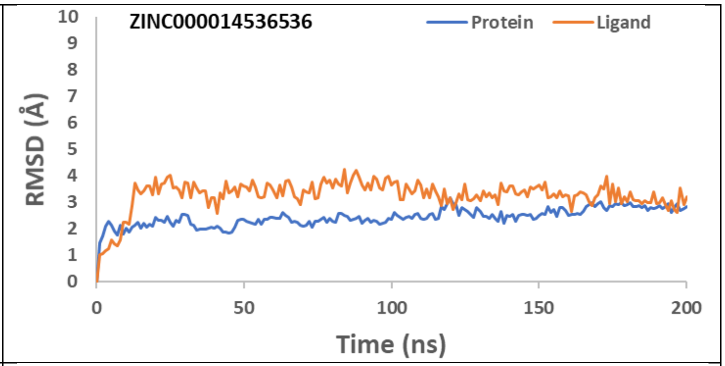
ZINC000014536536
Protein
Ligand
RMSD (Å)
Time (ns)

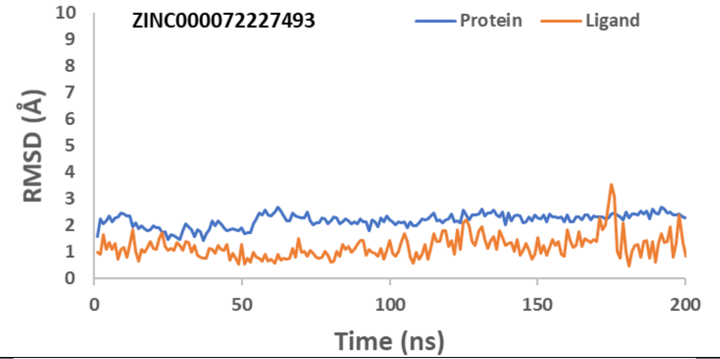
ZINC000072227493
Protein
Ligand
RMSD (Å)
Time (ns)

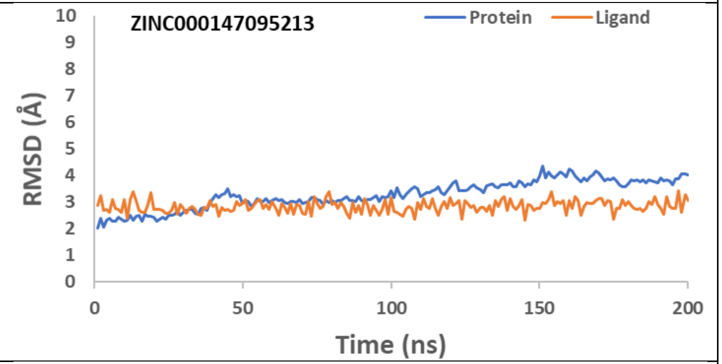
ZINC000147095213
Protein
Ligand
RMSD (Å)
Time (ns)

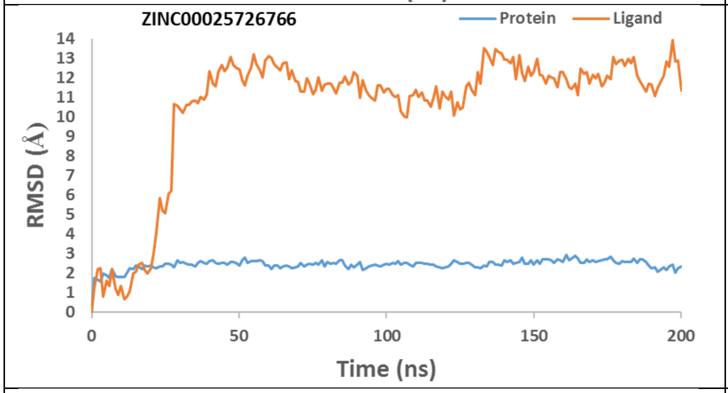
ZINC00025726766
Protein
Ligand
RMSD (Å)
Time (ns)

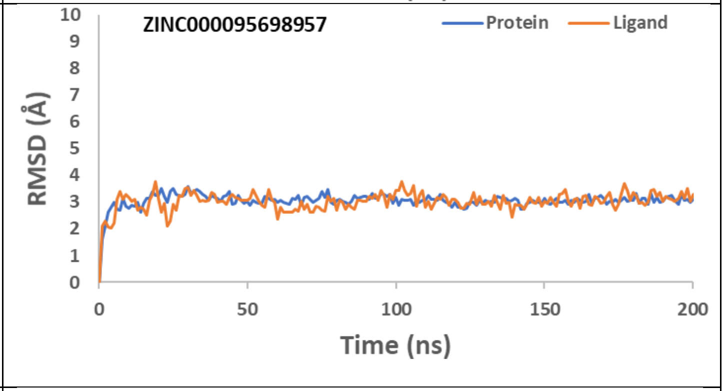
ZINC000095698957
Protein
Ligand
RMSD (Å)
Time (ns)

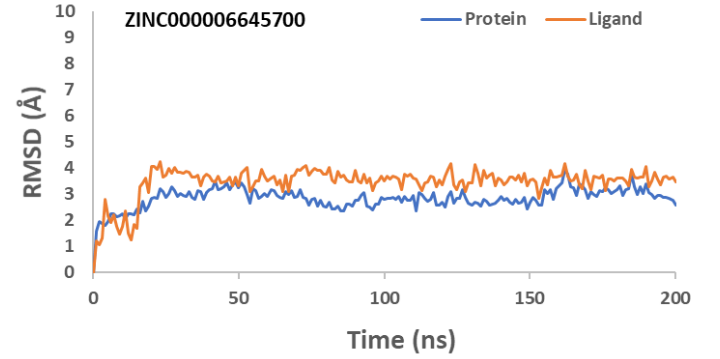
ZINC000006645700
Protein
Ligand
RMSD (Å)
Time (ns)

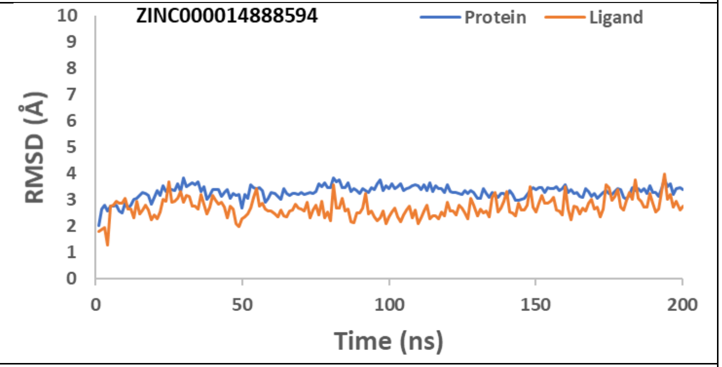
ZINC000014888594
Protein
Ligand
RMSD (Å)
Time (ns)

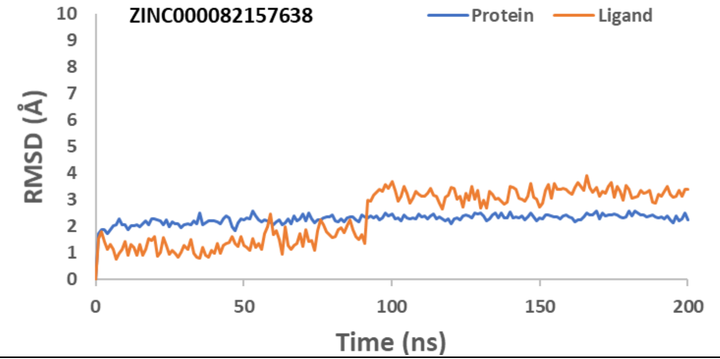
ZINC000082157638
Protein
Ligand
RMSD (Å)
Time (ns)

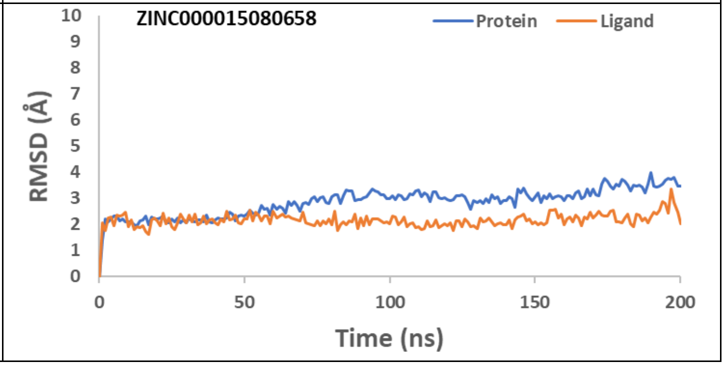
ZINC000015080658
Protein
Ligand
RMSD (Å)
Time (ns)

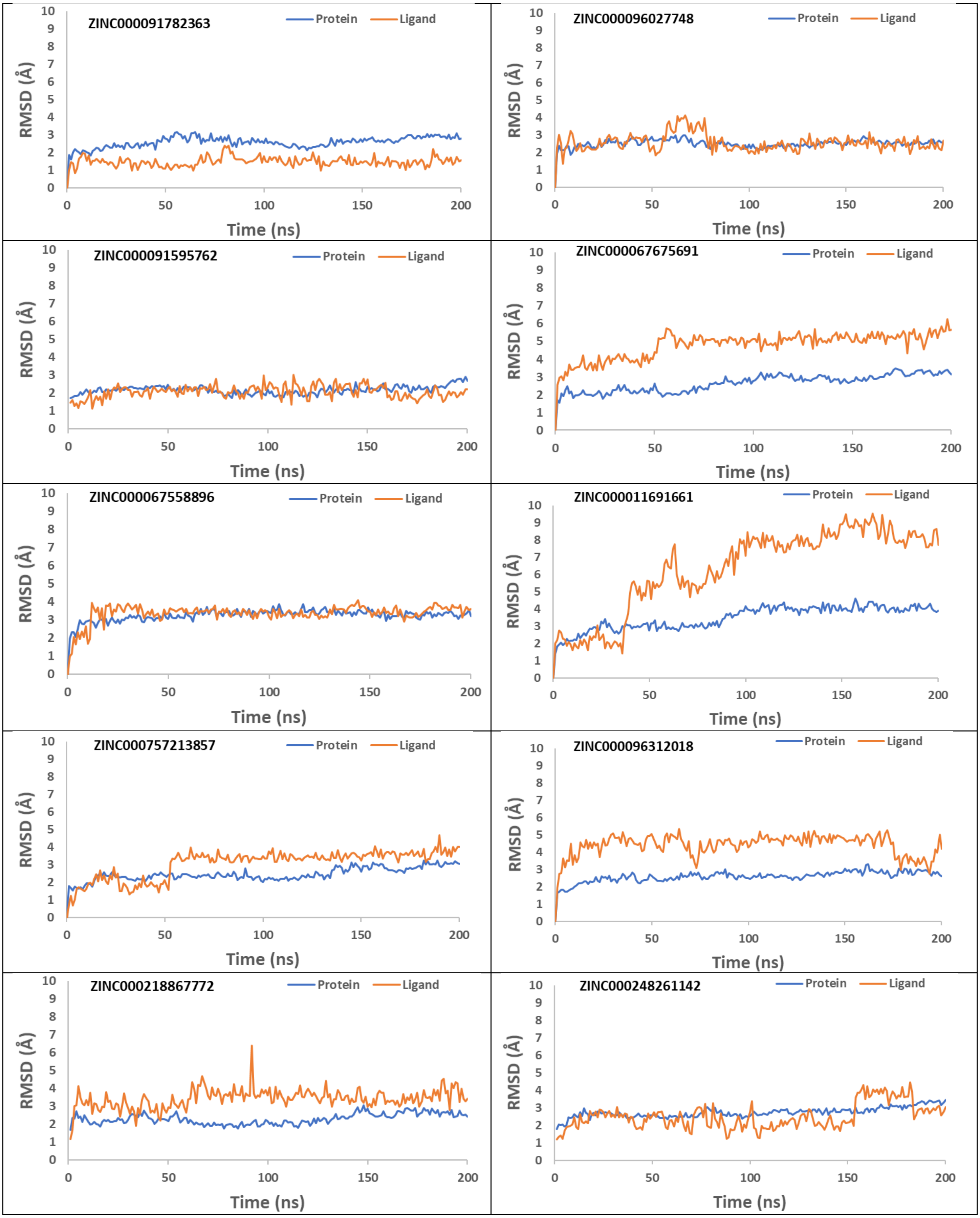
ZINC000091782363
ZINC000096027748
ZINC000091595762
ZINC000067675691
ZINC000067558896
ZINC000011691661
ZINC000757213857
ZINC000096312018
ZINC000218867772
ZINC000248261142
Protein
Ligand
RMSD (Å)
Time (ns)

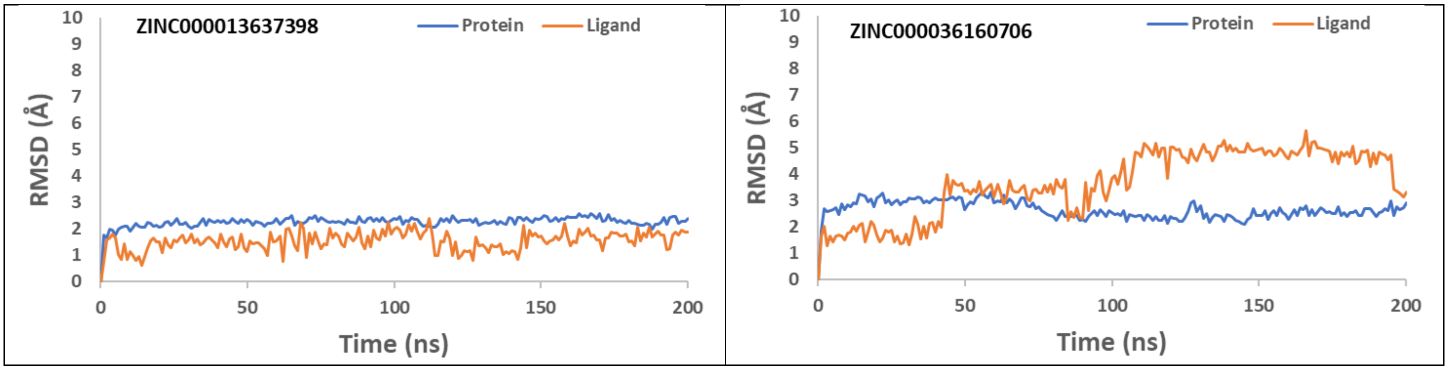

Figure S9. Cα RMSD of the top ZINC compounds during 200 ns MD simulation in reference to the crystal active DOR conformation (PDB ID: 6PT3).

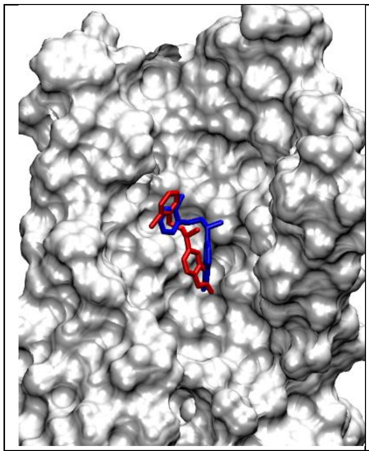	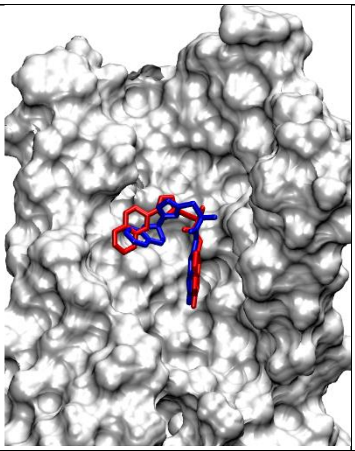	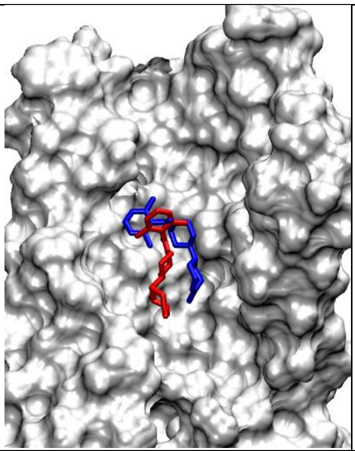	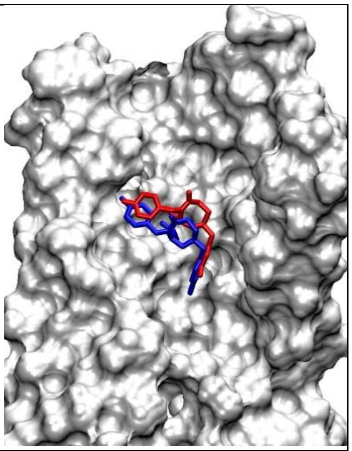
ZINC000020559278	ZINC000014242201	ZINC000562639987	ZINC000071763967
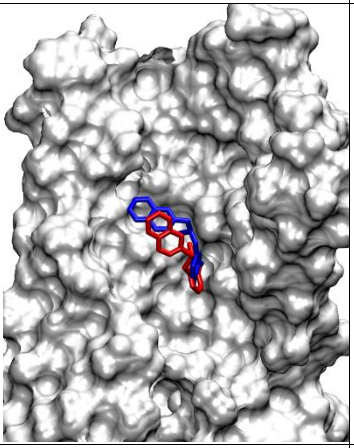	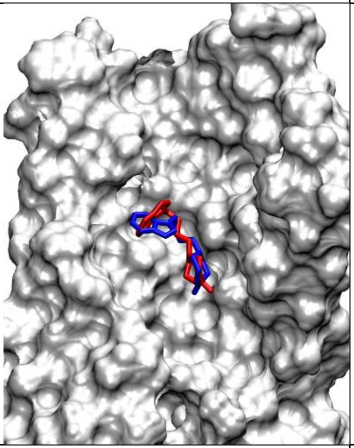	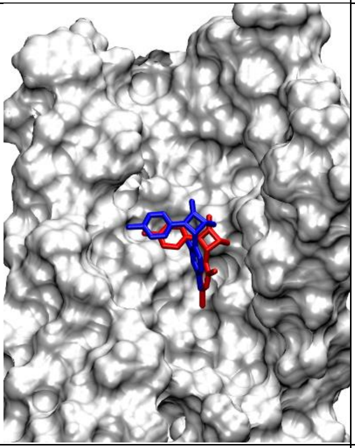	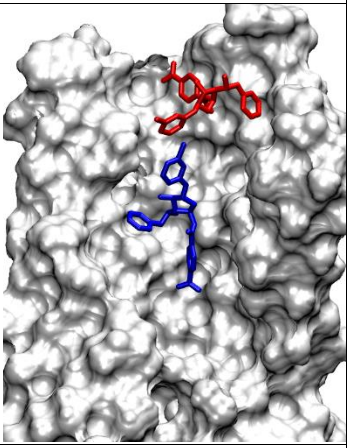
ZINC000067947687	ZINC000634950507	ZINC000072227493	ZINC000257262766
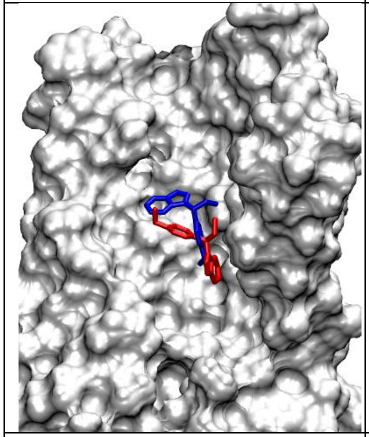	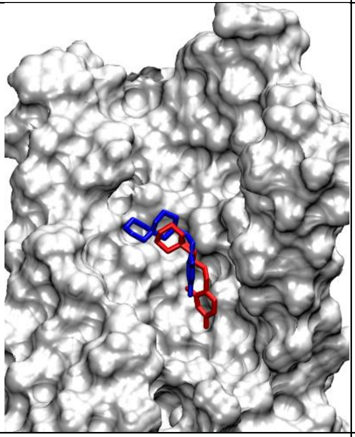	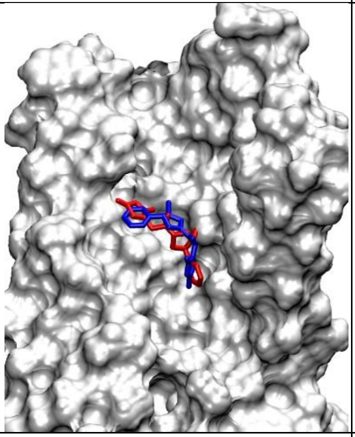	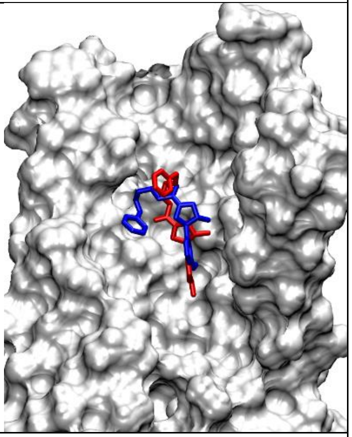
ZINC000006645700	ZINC000082157638	ZINC000091782363	ZINC000091595762

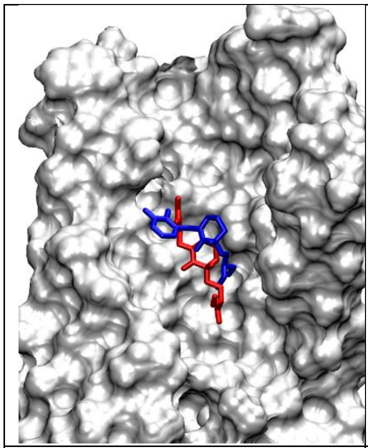
ZINC000067558896

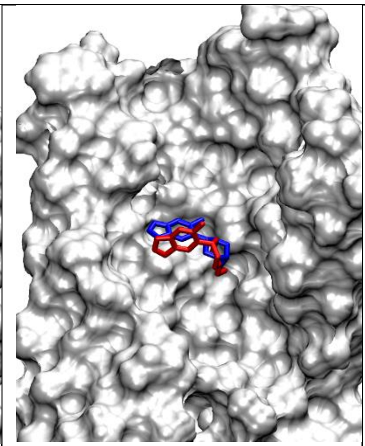
ZINC000757213857

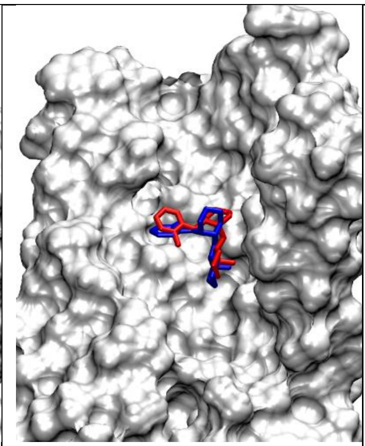
ZINC000218867772

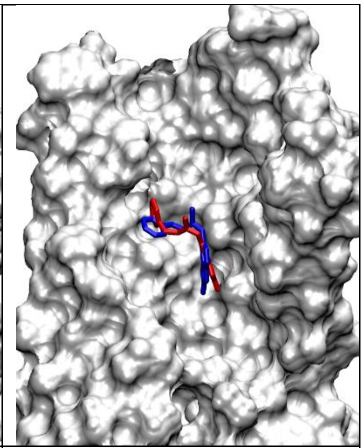
ZINC000013637398

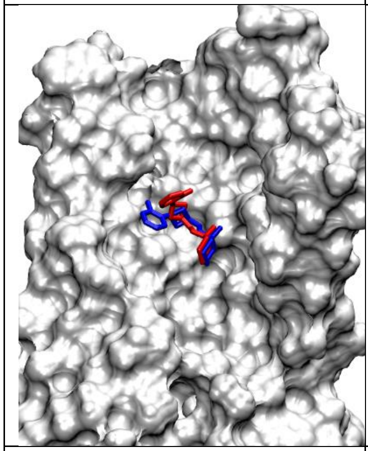
ZINC000078515864

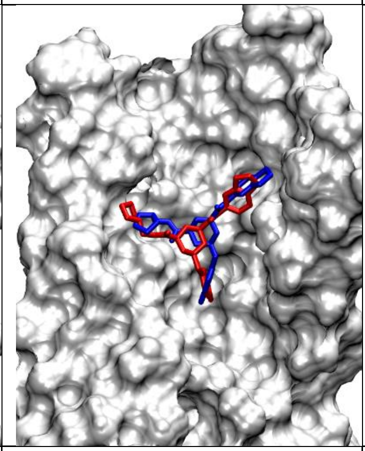
ZINC000014538415

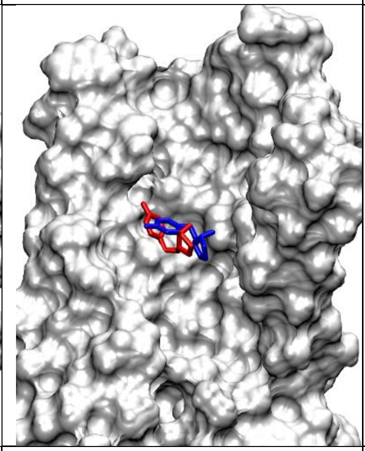
ZINC000218873420

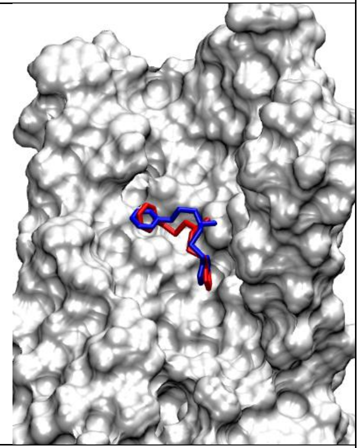
ZINC000001743629

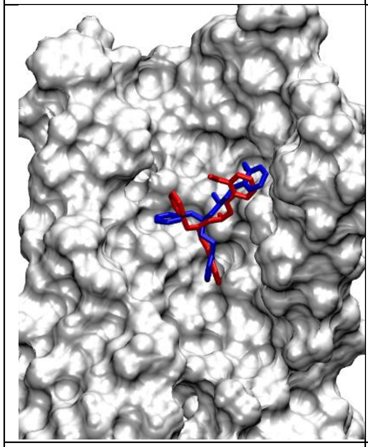
ZINC000028295259

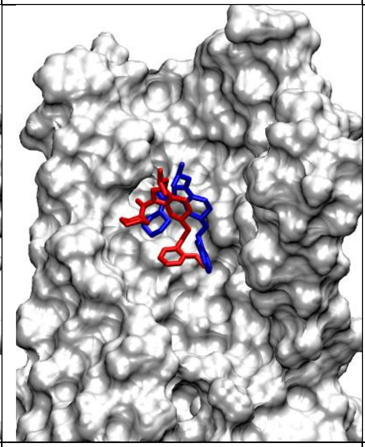
ZINC000014536536

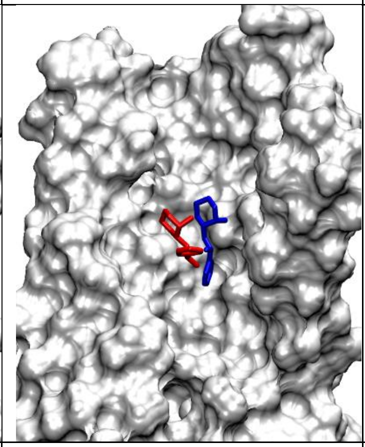
ZINC000147095213

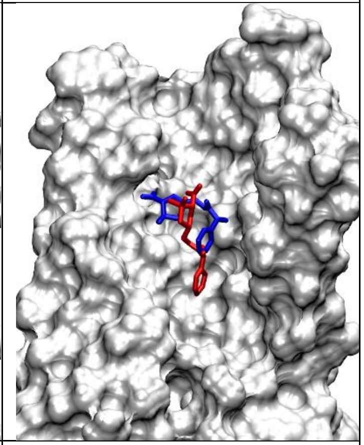
ZINC000095698957

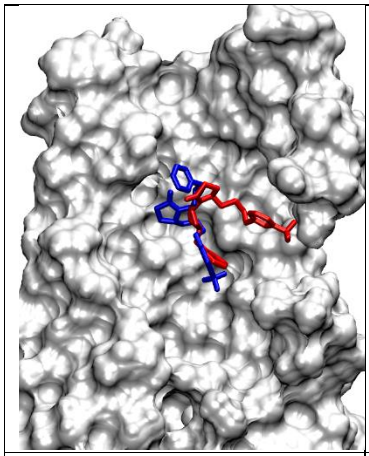

ZINC000014888594

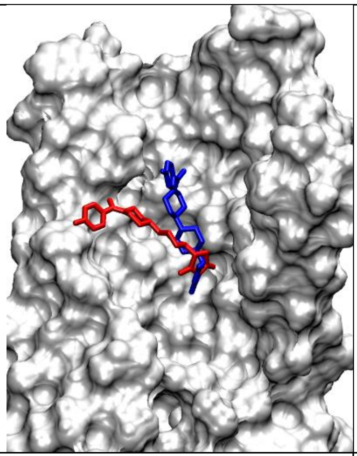

ZINC000015080658

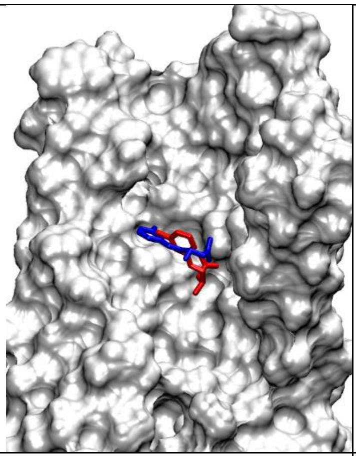

ZINC000096027748

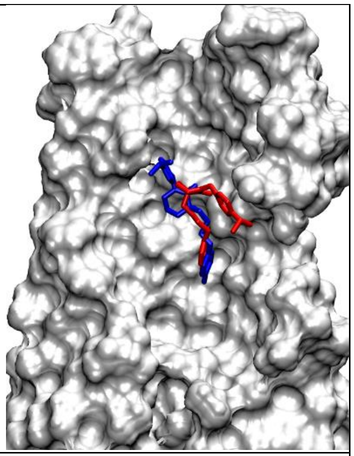

ZINC000067675691

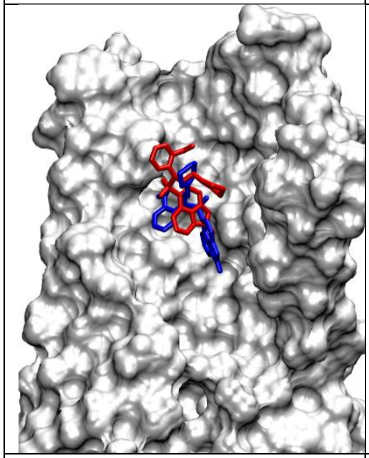

ZINC000011691661

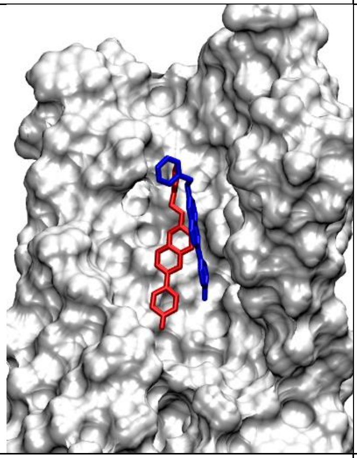

ZINC000096312018

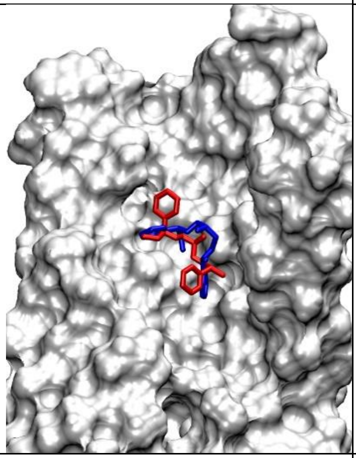

ZINC000248261142

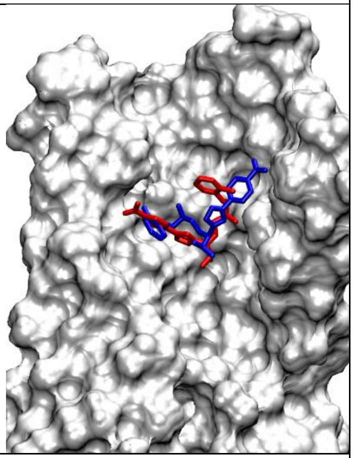

ZINC000036160706

Figure S10. Comparison of the top 32 ZINC compounds for the crystal conformation (PDB ID: 6PT3) in the docked pose (blue) and the MD simulation pose (red) with the DOR in surface representation (gray).

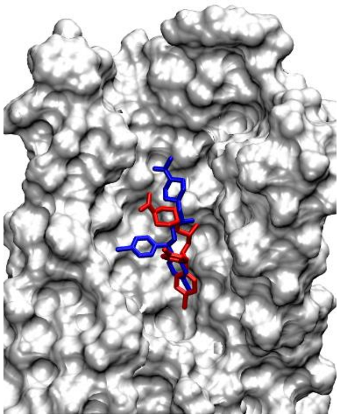	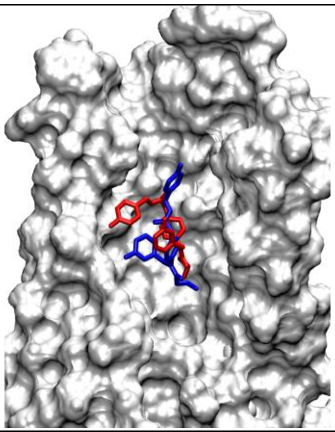	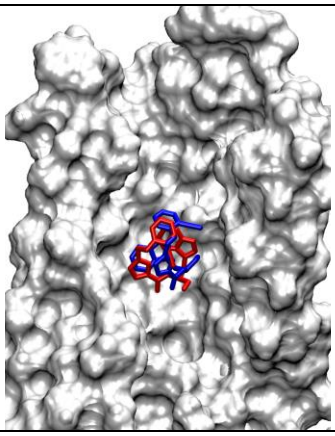	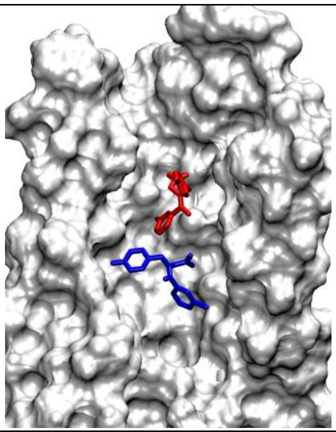
ZINC000025329384	ZINC000037556415	ZINC000095456365	ZINC000225318193
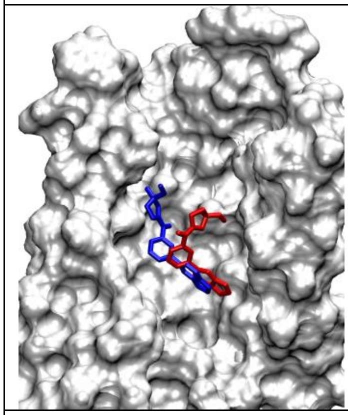	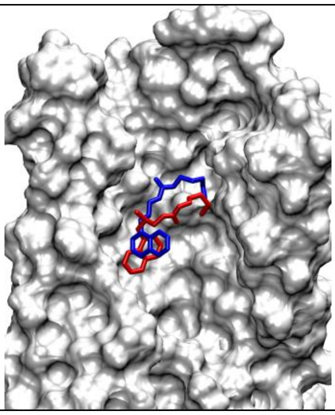	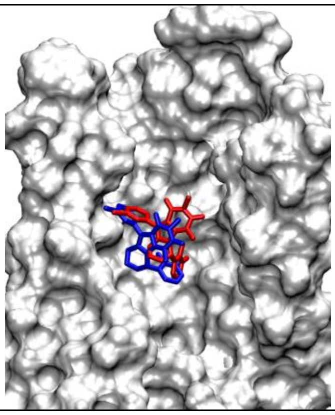	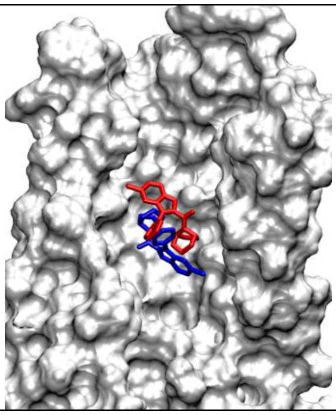
ZINC000299770618	ZINC000827360794	ZINC000035373220	ZINC000072430969
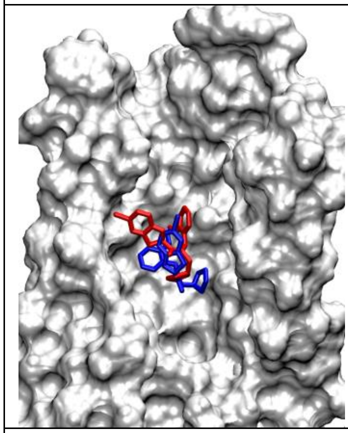	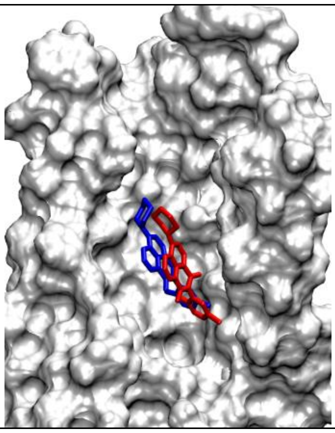	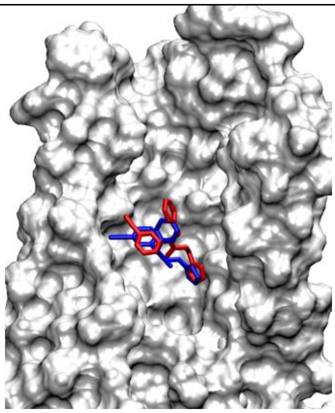	
ZINC000078648574	ZINC000005344596	ZINC000069348668	

Figure S11. Comparison of the top 11 ZINC compounds for the first representative structure from the MD crystal conformation (PDB ID: 6PT3) in the docked pose (blue) and the MD simulation pose (red) with the DOR in surface representation (gray).

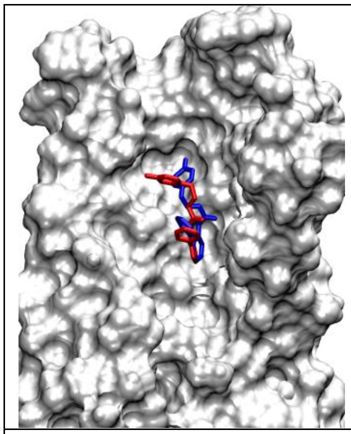	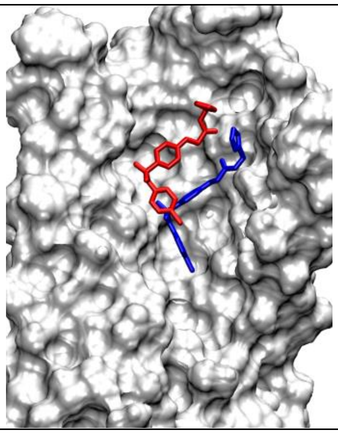	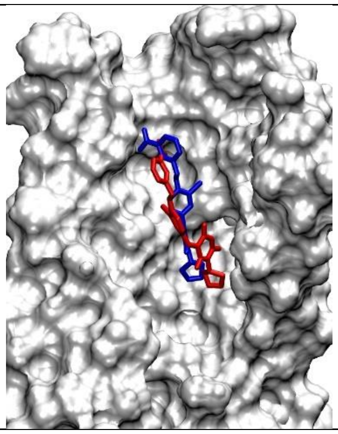	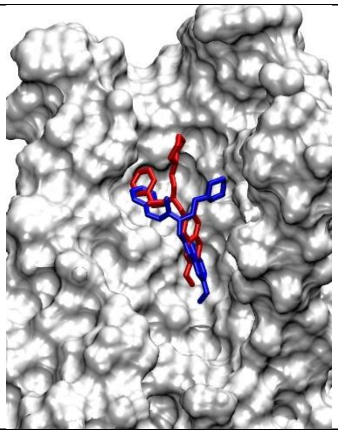
ZINC000057999653	ZINC000002877267	ZINC000408693879	ZINC000000880008
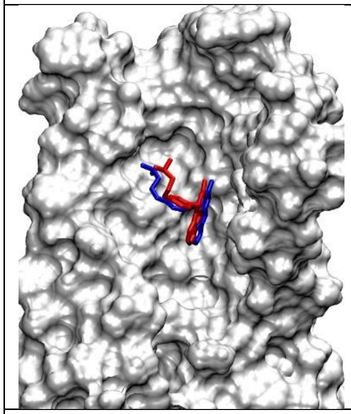	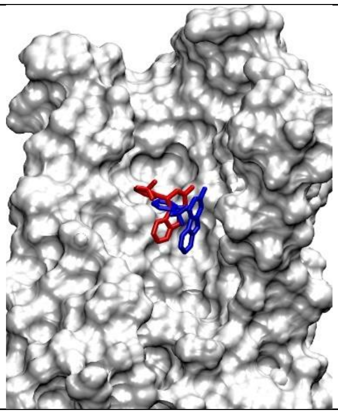	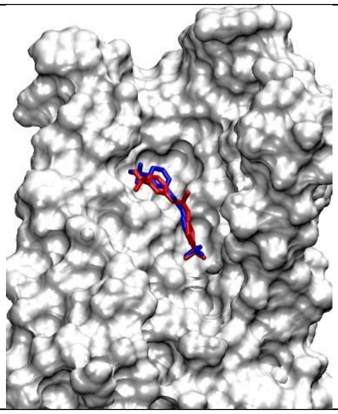	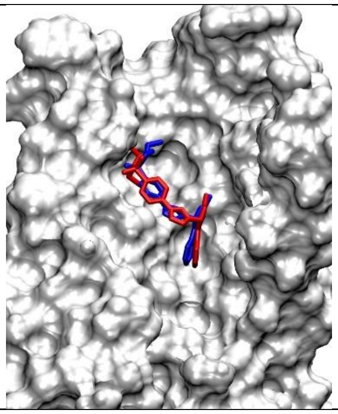
ZINC000006664413	ZINC000001408226	ZINC000001045477	ZINC000005493735
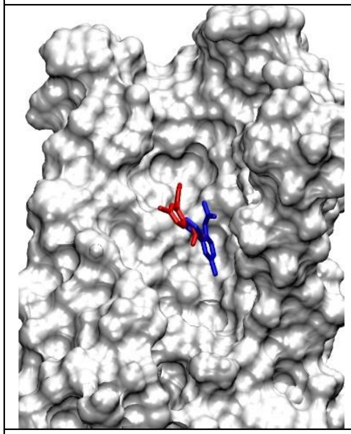	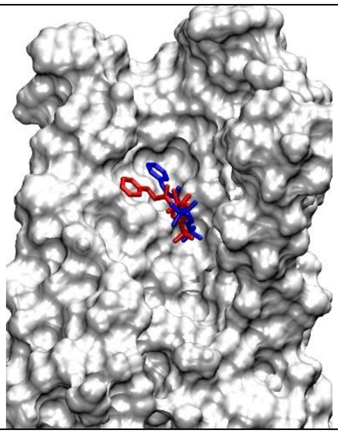	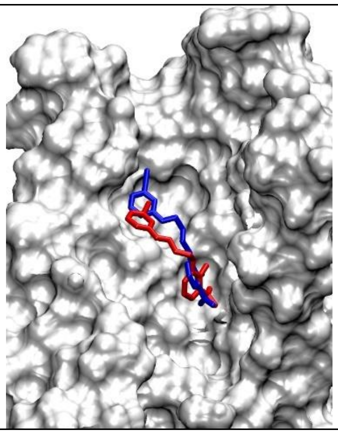	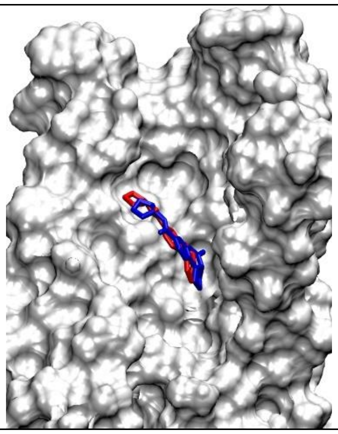
ZINC000001641702	ZINC000000302628	ZINC000095418373	ZINC000006750553
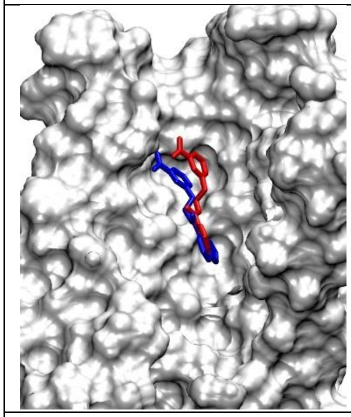	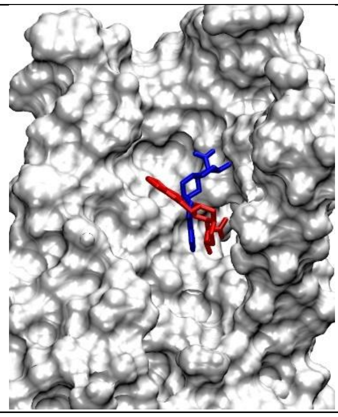	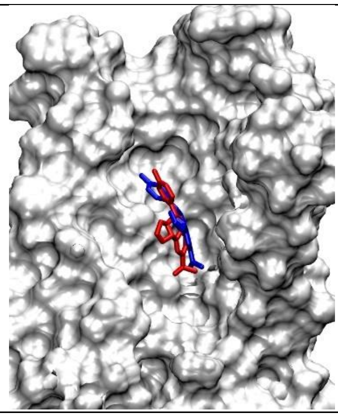	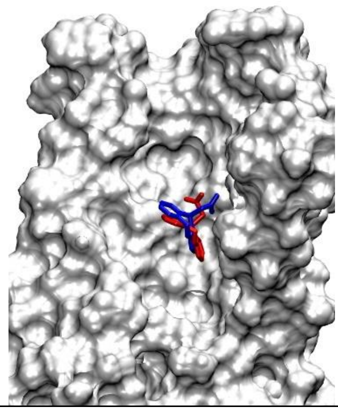
ZINC000097002851	ZINC000005776998	ZINC000004663101	ZINC000005604766

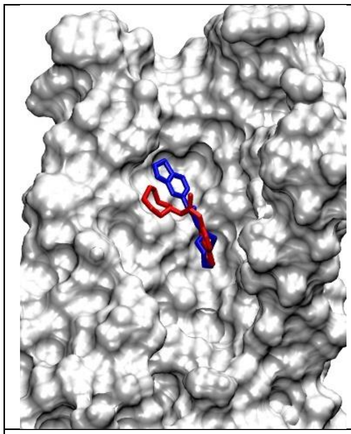	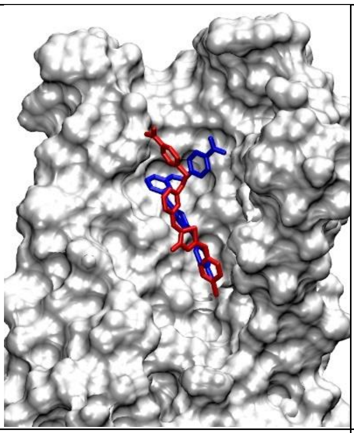	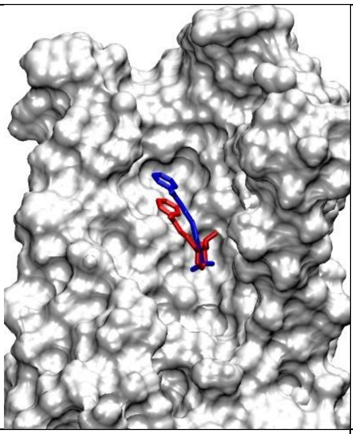	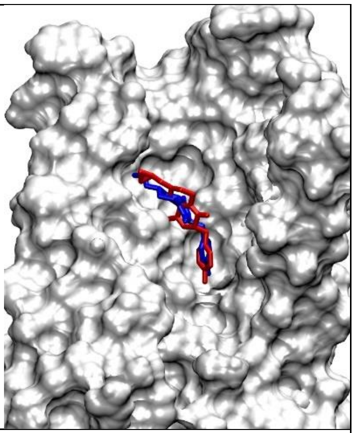
ZINC000225173433	ZINC000034720963	ZINC000409066936	ZINC000014750115
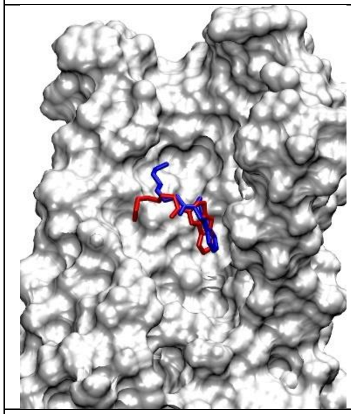	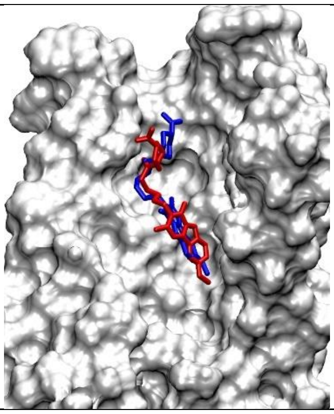	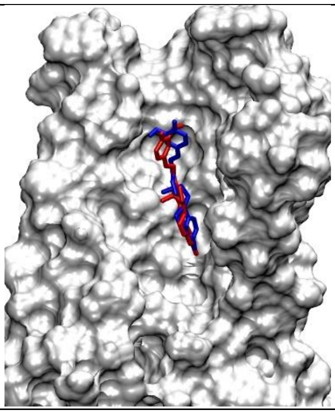	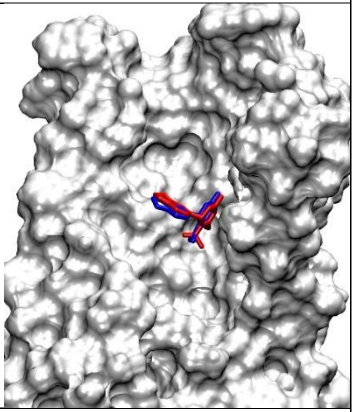
ZINC000059677349	ZINC000002690402	ZINC000020572602	ZINC000408729576
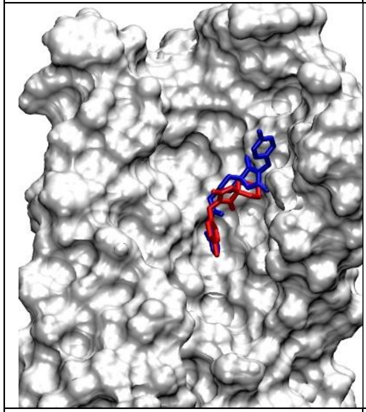	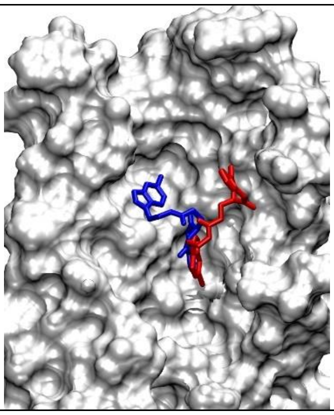		
ZINC000009660785	ZINC000334160398		

Figure S12. Comparison of the top 26 ZINC compounds for the second representative structure from the MD crystal conformation (PDB ID: 6PT3) in the docked pose (blue) and the MD simulation pose (red) with the DOR in surface representation (gray).

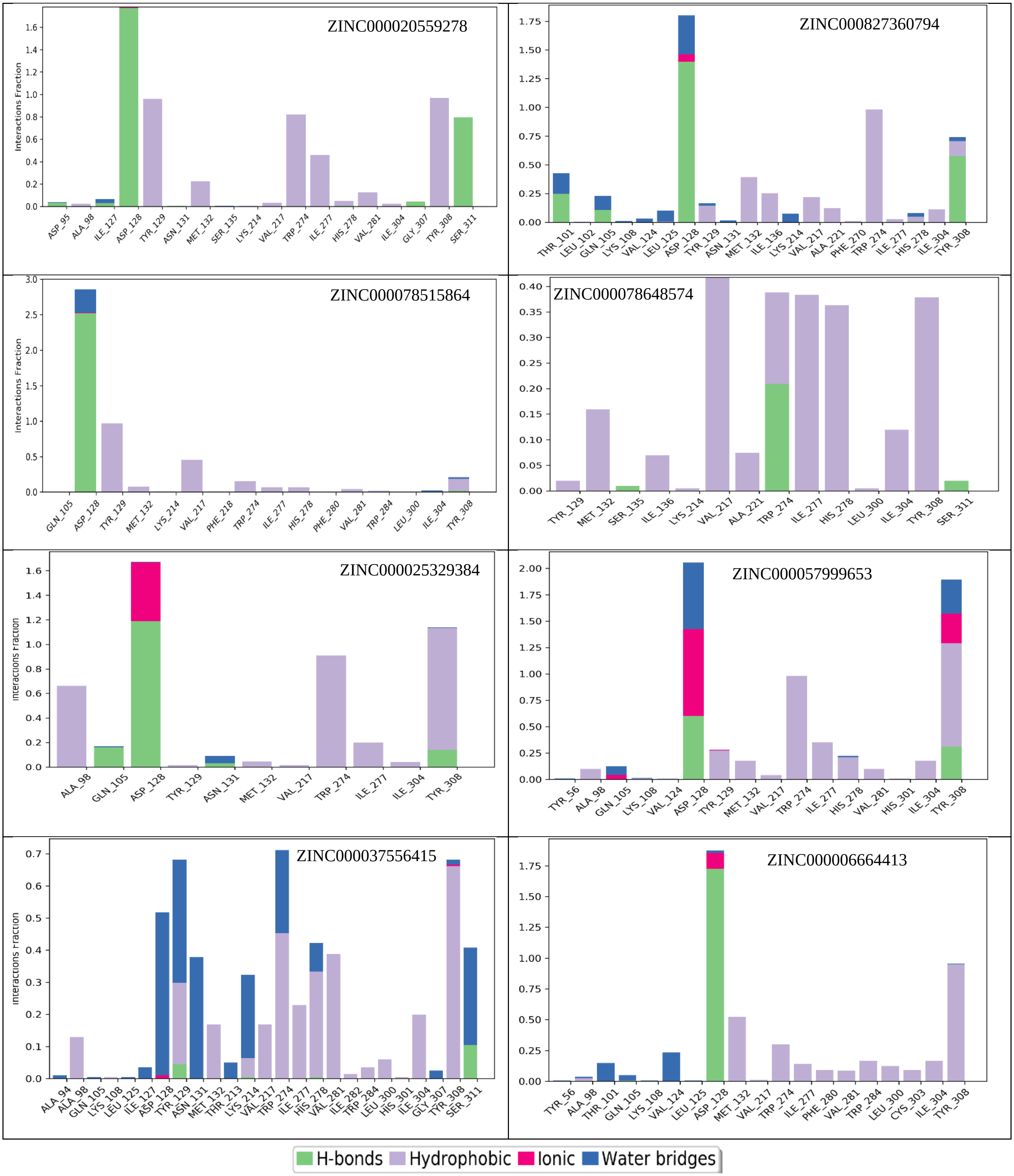

Figure S13. Protein-ligand contacts during MD simulations for the top 8 ZINC compounds. Interaction fraction greater than 1 is possible because of multiple contacts being made on the same residue.

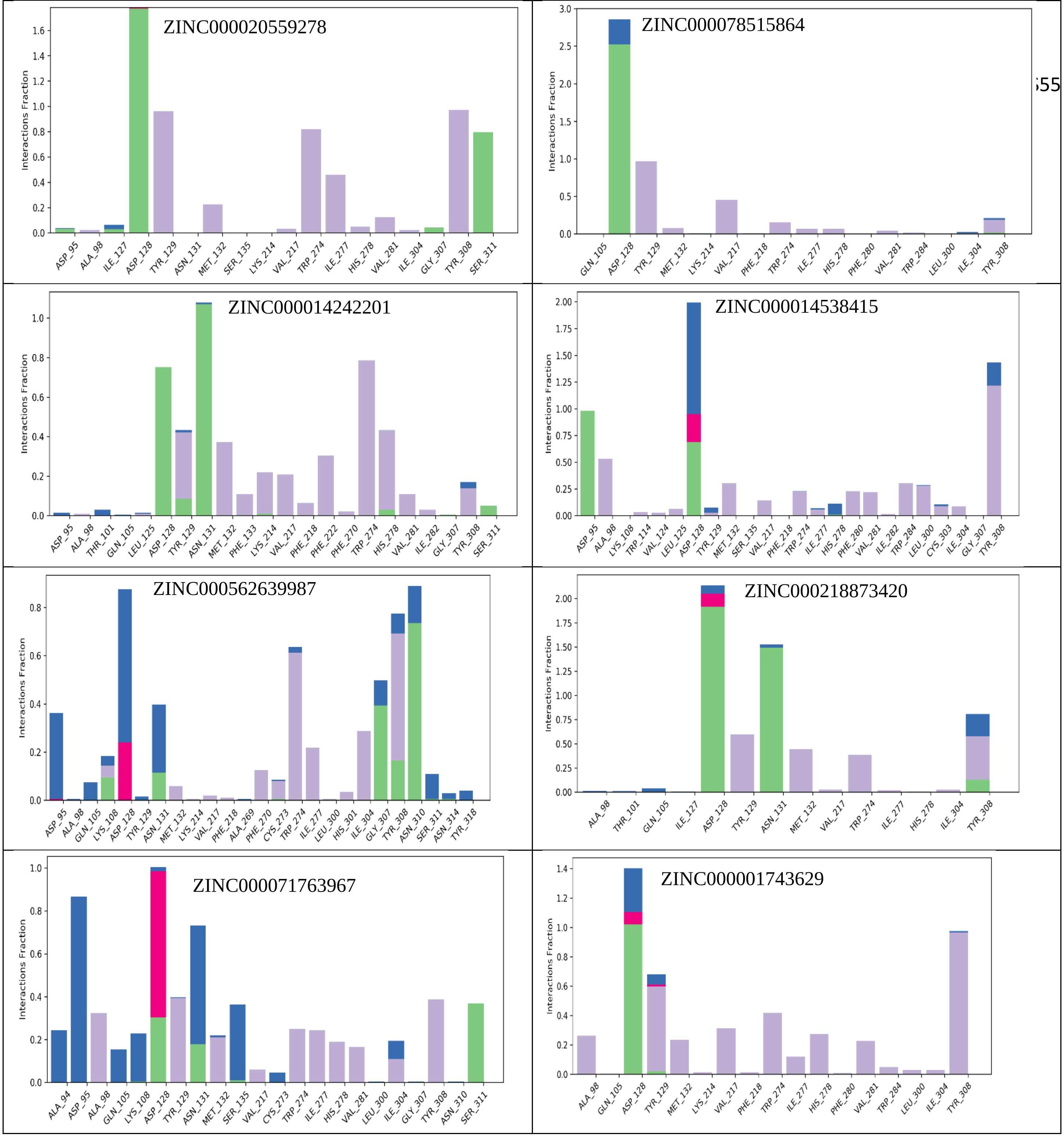
ZINC000020559278
ZINC000078515864
ZINC000014242201
ZINC000014538415
ZINC000562639987
ZINC000218873420
ZINC000071763967
ZINC000001743629
Interactions Fraction

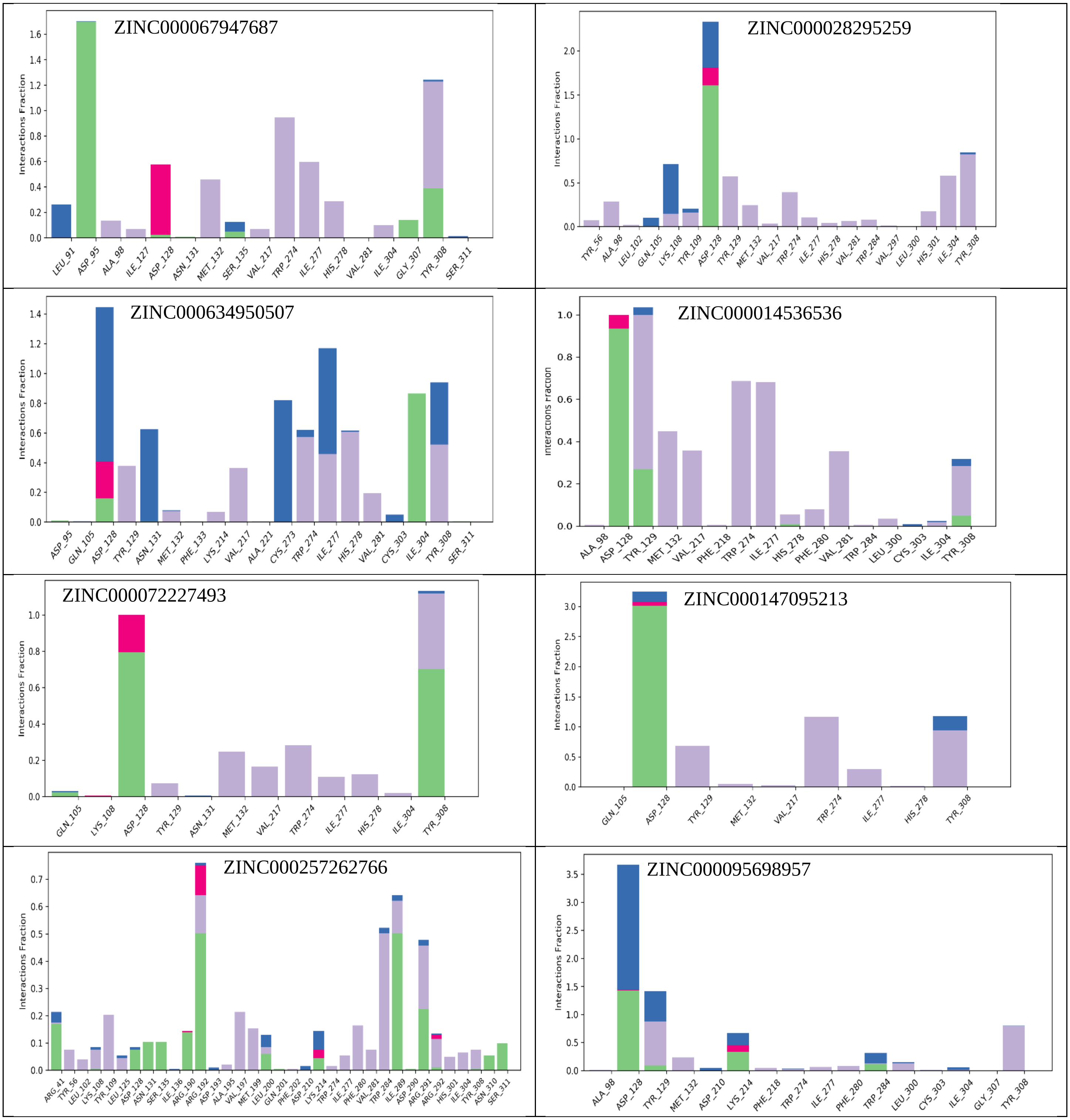
ZINC000067947687
ZINC000028295259
ZINC000634950507
ZINC000014536536
ZINC000072227493
ZINC000147095213
ZINC000257262766
ZINC000095698957
Interactions Fraction

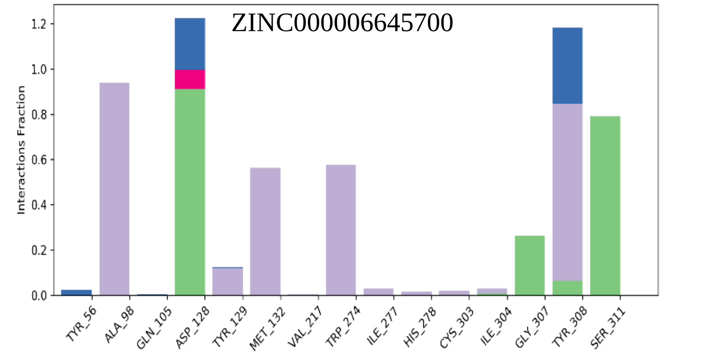
ZINC000006645700
Interactions Fraction
0.0
0.2
0.4
0.6
0.8
1.0
1.2
TYR_56
ALA_98
GLN_105
ASP_128
TYR_129
MET_132
VAL_217
TRP_274
ILE_277
HIS_278
CYS_303
ILE_304
GLY_307
TYR_308
SER_311

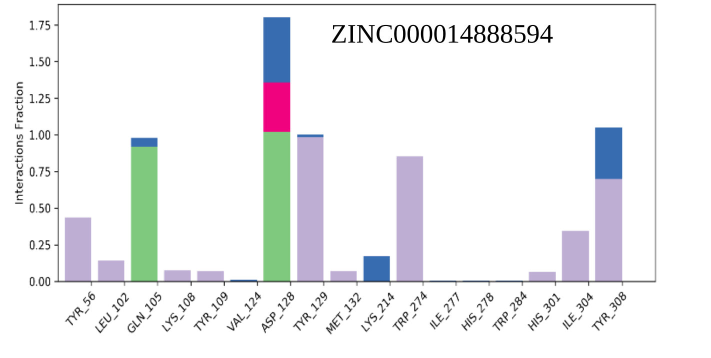
ZINC000014888594
Interactions Fraction
0.00
0.25
0.50
0.75
1.00
1.25
1.50
1.75
TYR_56
LEU_102
GLN_105
LYS_108
TYR_109
VAL_124
ASP_128
TYR_129
MET_132
LYS_214
TRP_274
ILE_277
HIS_278
TRP_284
HIS_301
ILE_304
TYR_308

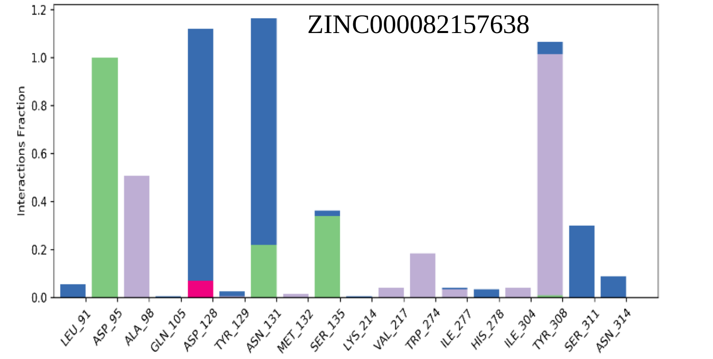
ZINC000082157638
Interactions Fraction
0.0
0.2
0.4
0.6
0.8
1.0
1.2
LEU_91
ASP_95
ALA_98
GLN_105
ASP_128
TYR_129
ASN_131
MET_132
SER_135
LYS_214
VAL_217
TRP_274
ILE_277
HIS_278
ILE_304
TYR_308
SER_311
ASN_314

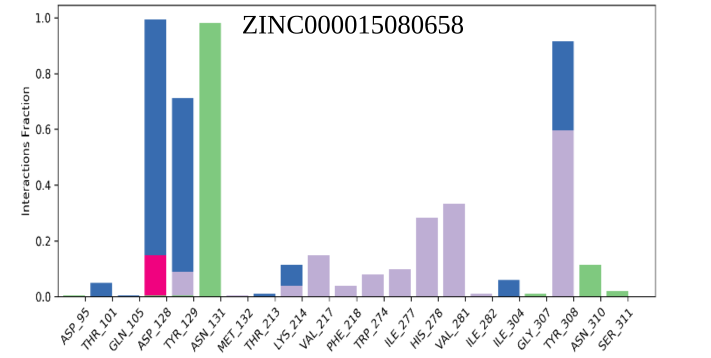
ZINC000015080658
Interactions Fraction
0.0
0.2
0.4
0.6
0.8
1.0
ASP_95
THR_101
GLN_105
ASP_128
TYR_129
ASN_131
MET_132
THR_213
LYS_214
VAL_217
PHE_218
TRP_274
ILE_277
HIS_278
VAL_281
ILE_282
ILE_304
GLY_307
TYR_308
ASN_310
SER_311

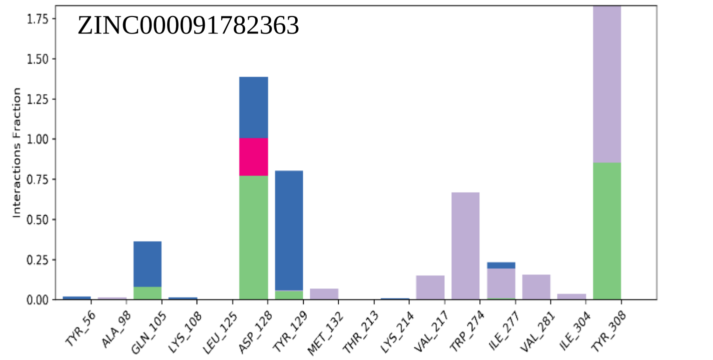
ZINC000091782363
Interactions Fraction
0.00
0.25
0.50
0.75
1.00
1.25
1.50
1.75
TYR_56
ALA_98
GLN_105
LYS_108
LEU_125
ASP_128
TYR_129
MET_132
THR_213
LYS_214
VAL_217
TRP_274
ILE_277
VAL_281
ILE_304
TYR_308

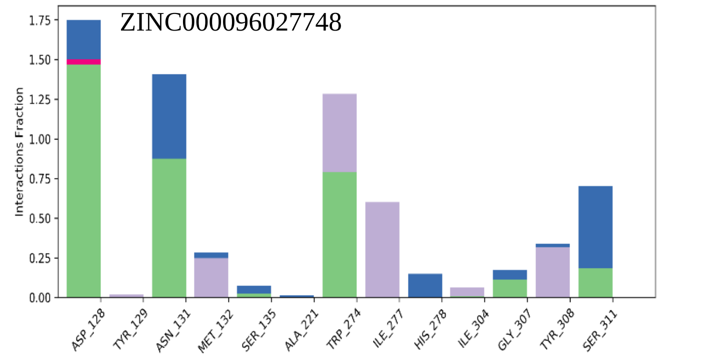
ZINC000096027748
Interactions Fraction
0.00
0.25
0.50
0.75
1.00
1.25
1.50
1.75
ASP_128
TYR_129
ASN_131
MET_132
SER_135
ALA_221
TRP_274
ILE_277
HIS_278
ILE_304
GLY_307
TYR_308
SER_311

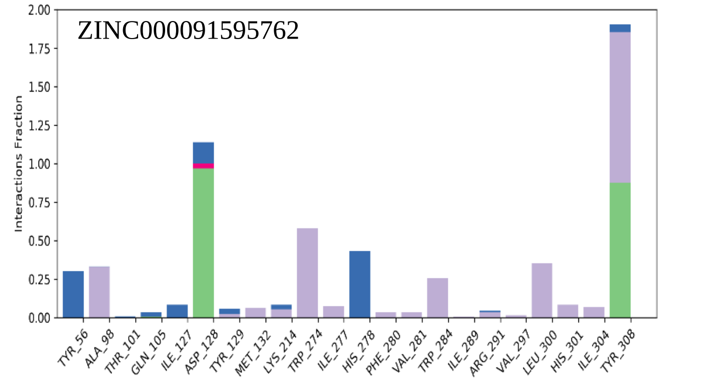
ZINC000091595762
Interactions Fraction
0.00
0.25
0.50
0.75
1.00
1.25
1.50
1.75
2.00
TYR_56
ALA_98
THR_101
GLN_105
ILE_127
ASP_128
TYR_129
MET_132
LYS_214
TRP_274
ILE_277
HIS_278
PHE_280
VAL_281
TRP_284
ILE_289
ARG_291
VAL_297
LEU_300
HIS_301
ILE_304
TYR_308

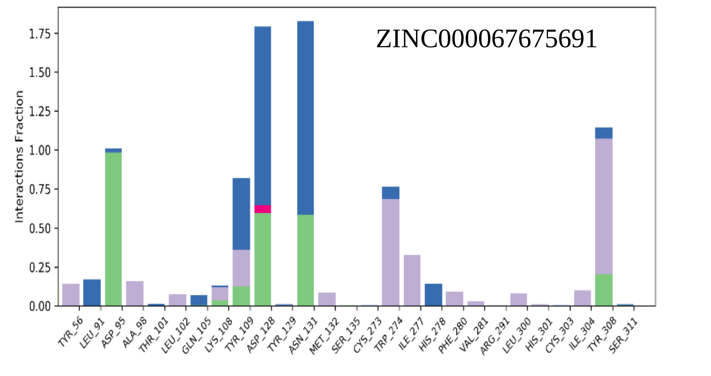
ZINC000067675691
Interactions Fraction
0.00
0.25
0.50
0.75
1.00
1.25
1.50
1.75
TYR_56
LEU_91
ASP_95
ALA_98
THR_101
LEU_102
GLN_105
LYS_108
TYR_109
ASP_128
TYR_129
ASN_131
MET_132
SER_135
CYS_273
TRP_274
ILE_277
HIS_278
PHE_280
VAL_281
ARG_291
LEU_300
HIS_301
CYS_303
ILE_304
TYR_308
SER_311

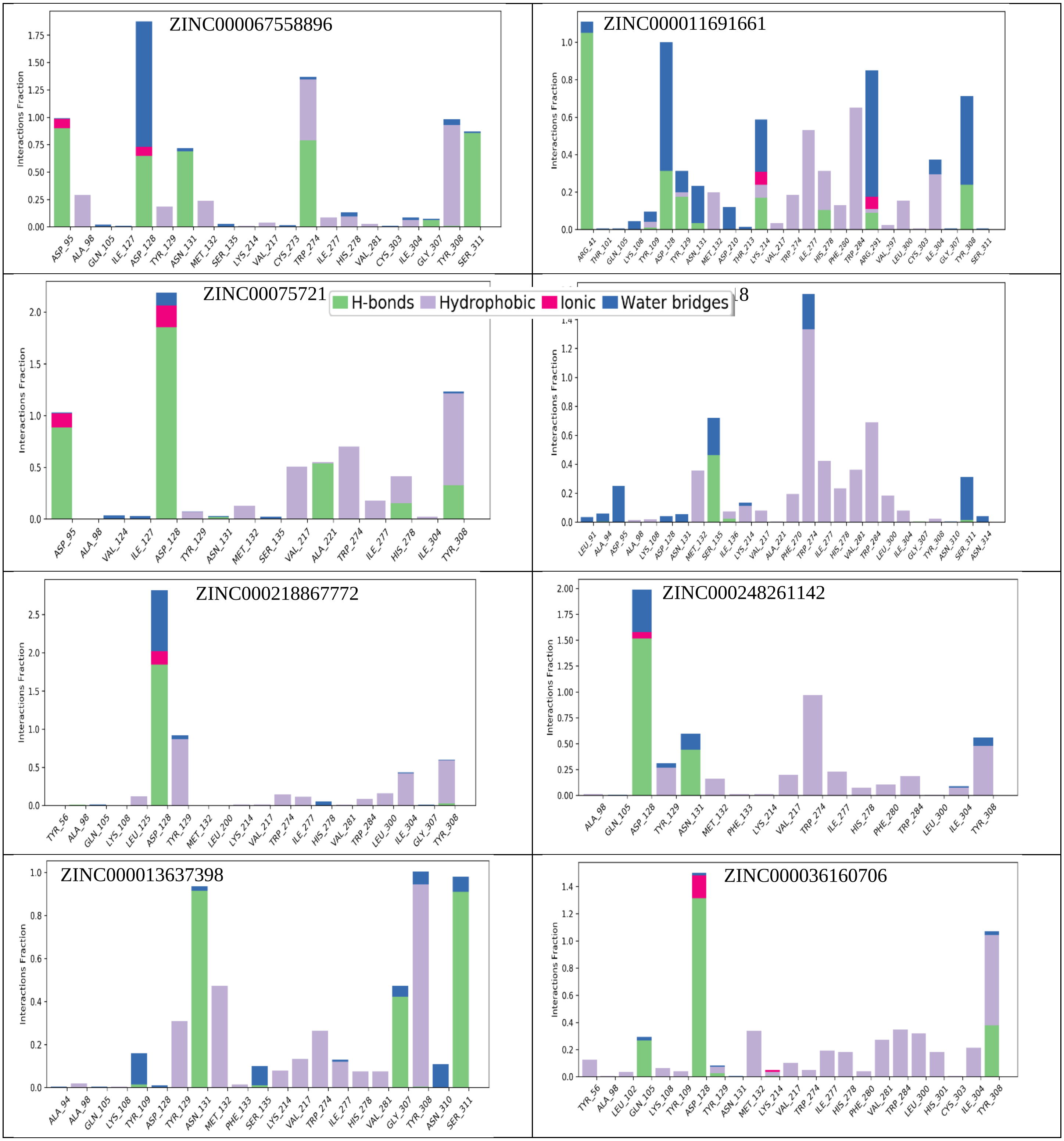

Figure S14. Protein-Ligand contacts during MD simulations for top 32 ZINC compounds for the crystal conformation (PDB ID: 6PT3). Interaction fraction greater than 1 is possible because of multiple contacts being made on the same residue.

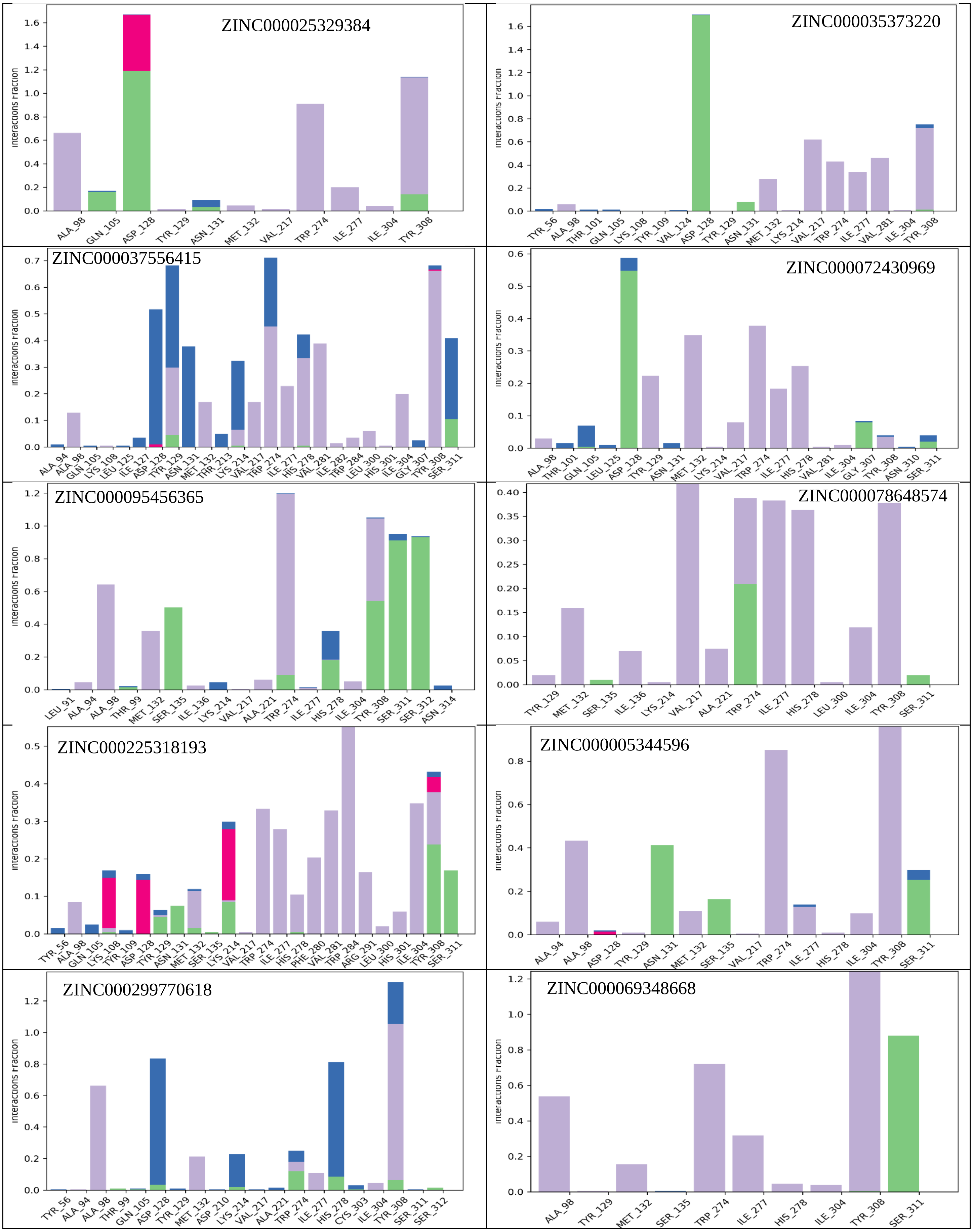
ZINC000025329384
interactions fraction
ALA_98 GLN_105 ASP_128 TYR_129 ASN_131 MET_132 VAL_217 TRP_274 ILE_277 ILE_304 TYR_308
ZINC000035373220
interactions fraction
TYR_56 ALA_98 THR_101 GLN_105 LYS_108 TYR_109 VAL_124 ASP_128 TYR_129 ASN_131 MET_132 LYS_214 VAL_217 TRP_274 ILE_277 VAL_281 ILE_304 TYR_308
ZINC000037556415
interactions fraction
ALA_94 ALA_98 GLN_105 LYS_108 LEU_125 ILE_127 ASP_128 TYR_129 ASN_131 MET_132 THR_213 LYS_214 VAL_217 TRP_274 ILE_277 HIS_278 VAL_281 ILE_282 TRP_284 LEU_300 HIS_301 ILE_304 GLY_307 TYR_308 SER_311
ZINC000072430969
interactions fraction
ALA_98 THR_101 GLN_105 LEU_125 ASP_128 TYR_129 ASN_131 MET_132 LYS_214 VAL_217 TRP_274 ILE_277 HIS_278 VAL_281 ILE_304 GLY_307 TYR_308 ASN_310 SER_311
ZINC000095456365
interactions fraction
LEU_91 ALA_94 ALA_98 THR_99 MET_132 SER_135 ILE_136 LYS_214 VAL_217 ALA_221 TRP_274 ILE_277 HIS_278 ILE_304 TYR_308 SER_311 SER_312 ASN_314
ZINC000078648574
TYR_129 MET_132 SER_135 ILE_136 LYS_214 VAL_217 ALA_221 TRP_274 ILE_277 HIS_278 LEU_300 ILE_304 TYR_308 SER_311
ZINC000225318193
interactions fraction
TYR_56 ALA_98 GLN_105 LYS_108 TYR_109 ASP_128 TYR_129 ASN_131 MET_132 SER_135 LYS_214 VAL_217 TRP_274 ILE_277 HIS_278 PHE_280 VAL_281 TRP_284 ARG_291 LEU_300 HIS_301 ILE_304 TYR_308 SER_311
ZINC000005344596
interactions fraction
ALA_94 ALA_98 ASP_128 TYR_129 ASN_131 MET_132 SER_135 VAL_217 TRP_274 ILE_277 HIS_278 ILE_304 TYR_308 SER_311
ZINC000299770618
interactions fraction
TYR_56 ALA_94 ALA_98 THR_99 GLN_105 ASP_128 TYR_129 MET_132 ASP_210 LYS_214 VAL_217 ALA_221 TRP_274 ILE_277 HIS_278 CYS_303 ILE_304 TYR_308 SER_311 SER_312
ZINC000069348668
interactions fraction
ALA_98 TYR_129 MET_132 SER_135 TRP_274 ILE_277 HIS_278 ILE_304 TYR_308 SER_311

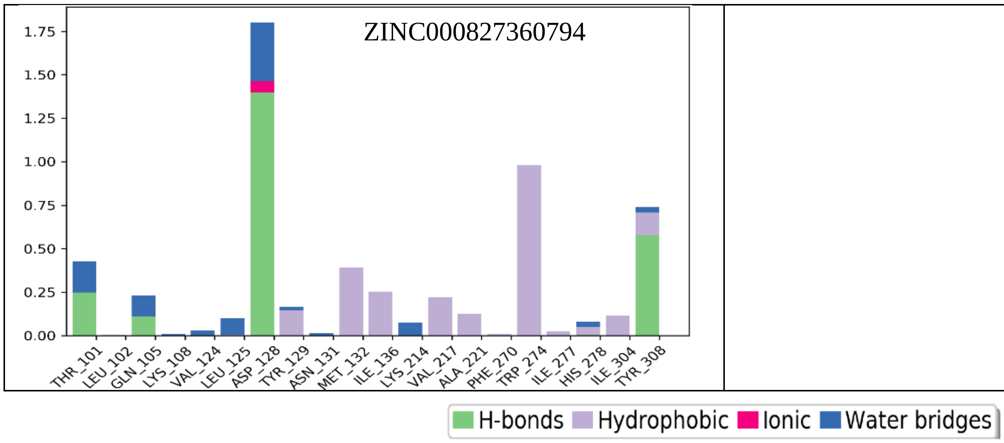

Figure S15. Protein-Ligand contacts during MD simulations for top 11 ZINC compounds for the first representative structure from the MD of the crystal conformation (PDB ID: 6PT3). Interaction fraction greater than 1 is possible because of multiple contacts being made on the same residue.

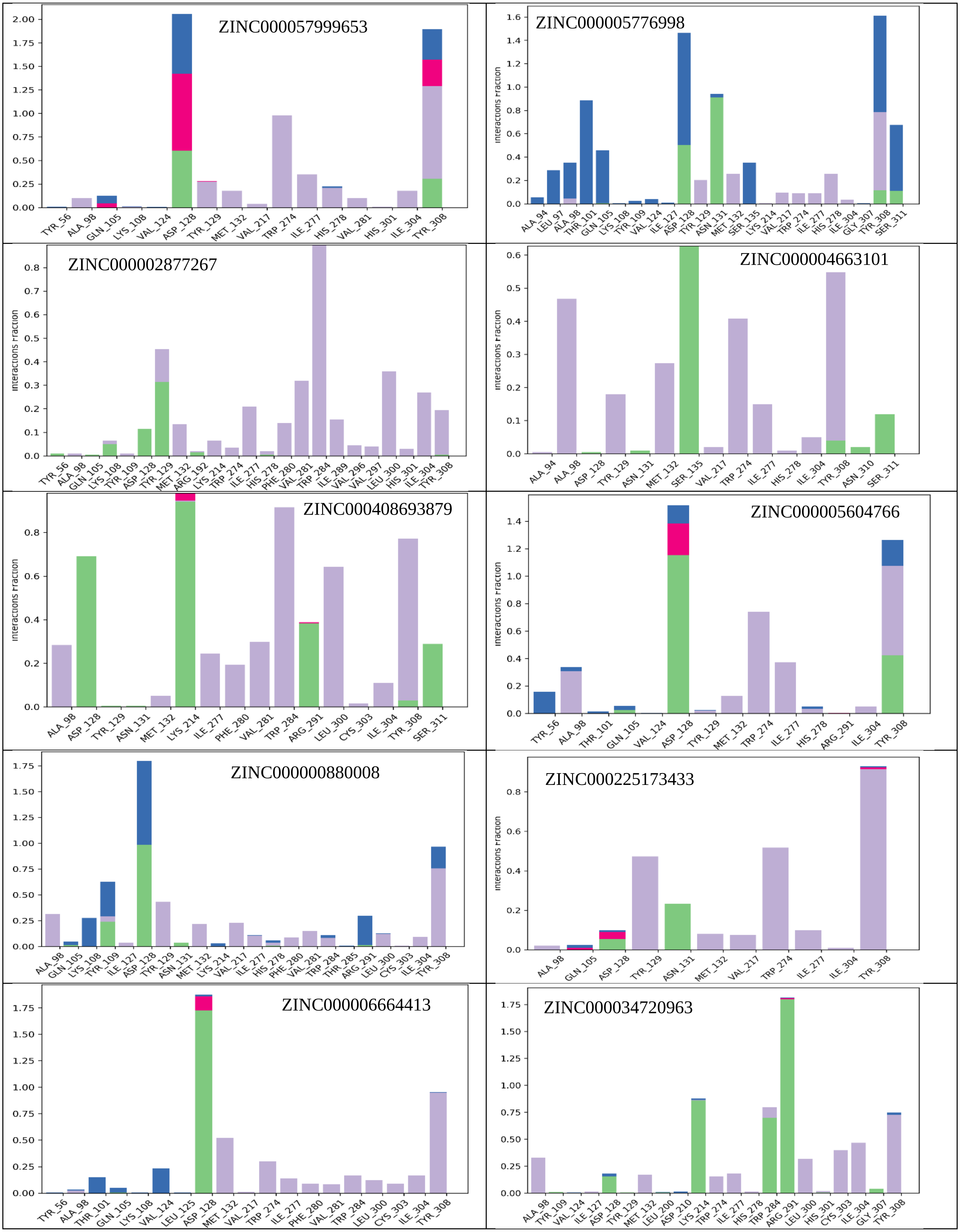
ZINC000057999653
ZINC000005776998
ZINC000002877267
ZINC000004663101
ZINC000408693879
ZINC000005604766
ZINC000000880008
ZINC000225173433
ZINC000006664413
ZINC000034720963
Interactions Fraction

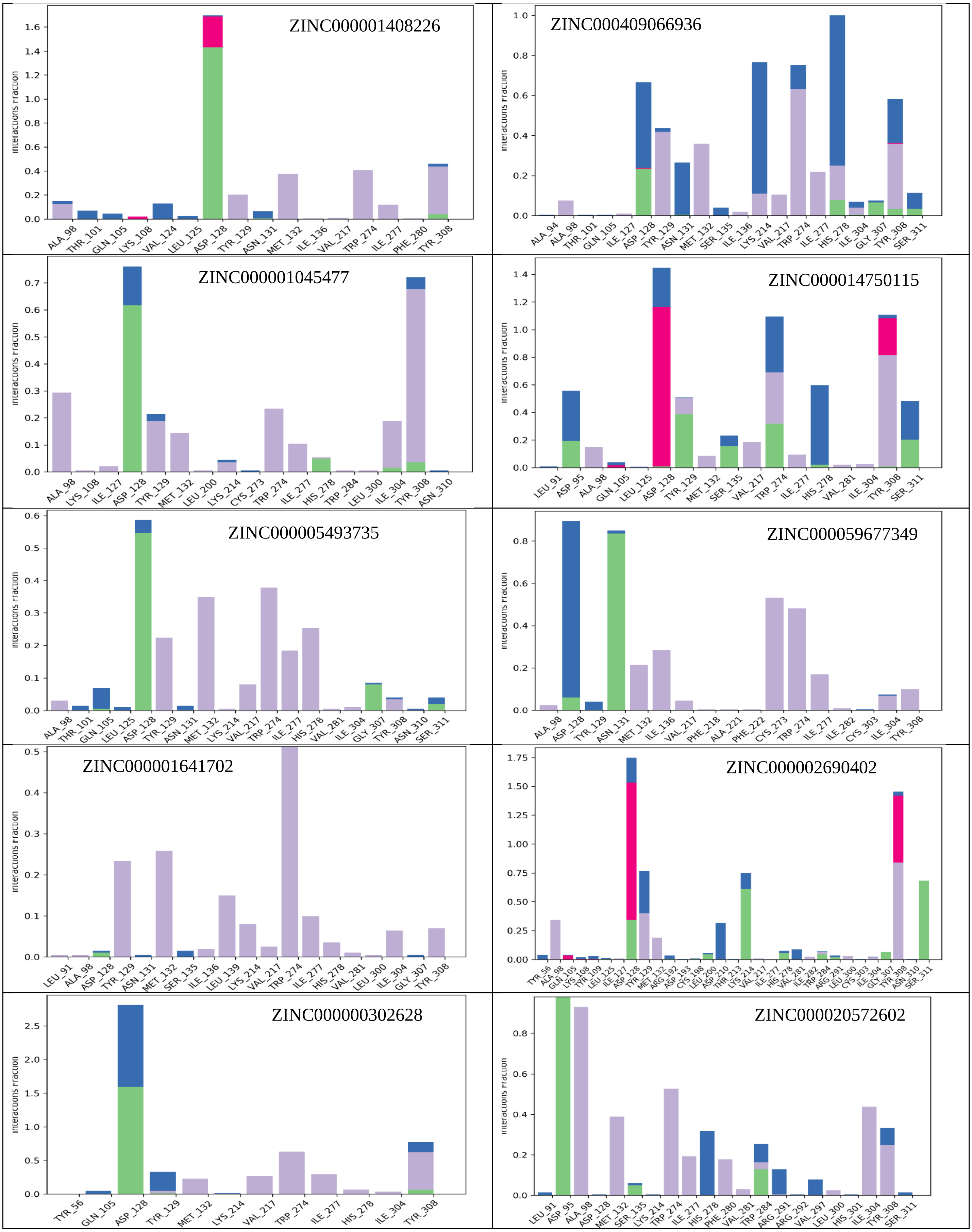
ZINC000001408226
ZINC000409066936
ZINC000001045477
ZINC000014750115
ZINC000005493735
ZINC000059677349
ZINC000001641702
ZINC000002690402
ZINC000000302628
ZINC000020572602
Interactions Fraction

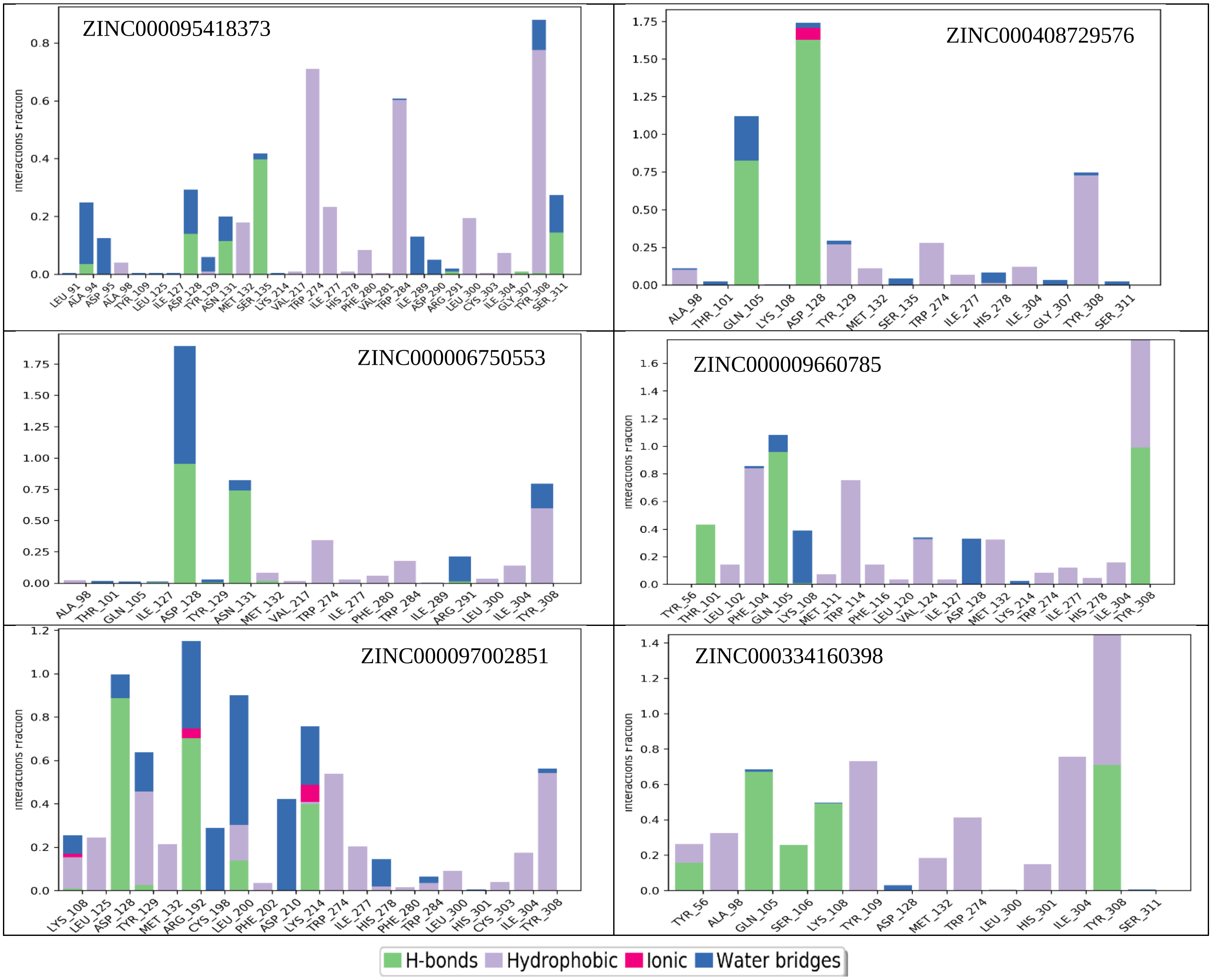

Figure S16. Protein-Ligand contacts during MD simulations for top 26 ZINC compounds for the second representative structure from the MD of the crystal conformation (PDB ID: 6PT3). Interaction fraction greater than 1 is possible because of multiple contacts being made on the same residue.

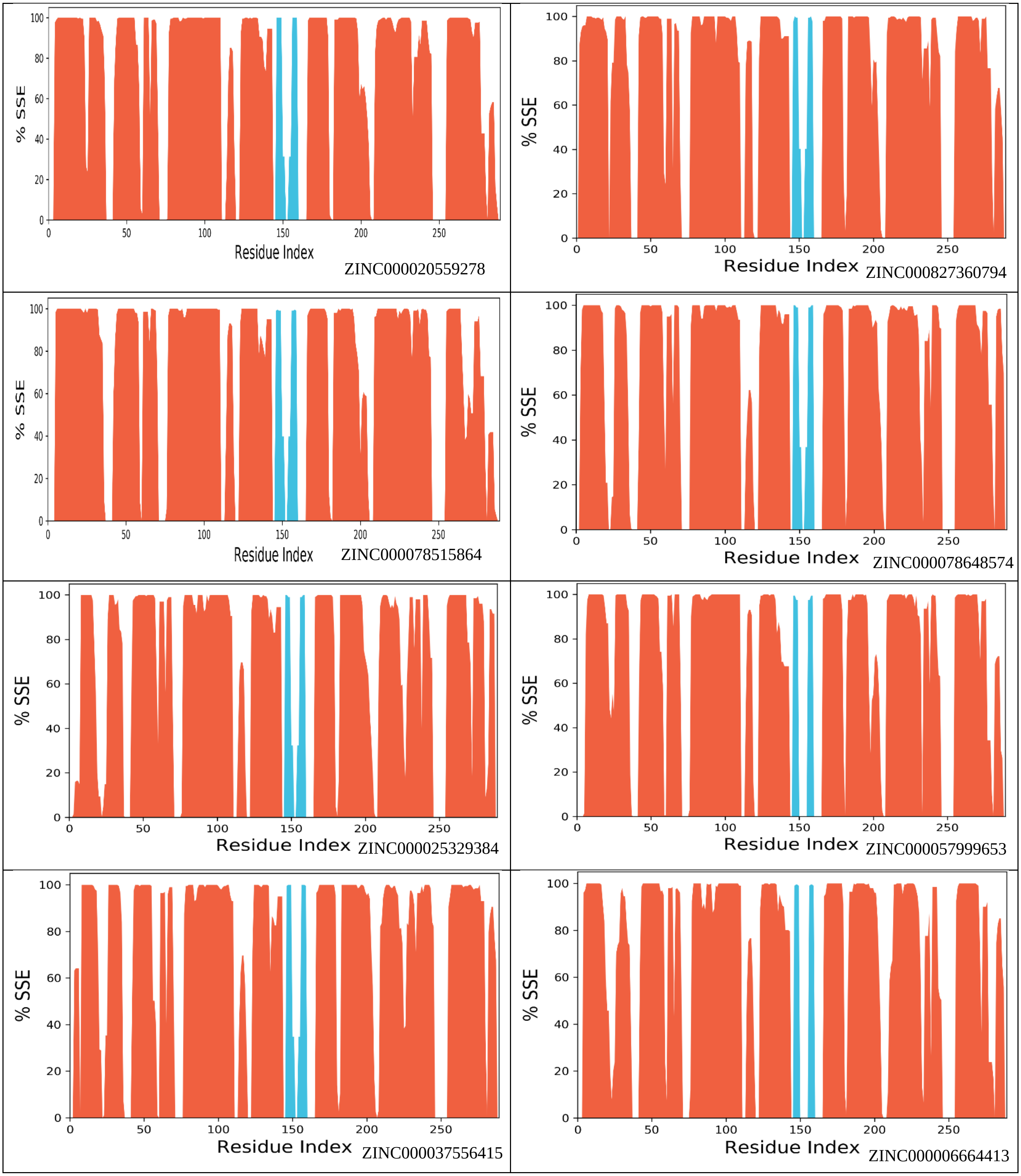

Figure S17. Protein secondary structure elements (SSE) of the receptor in complex with the top 8 ZINC compounds. Alpha helices are represented by orange and beta sheets are represented by blue.

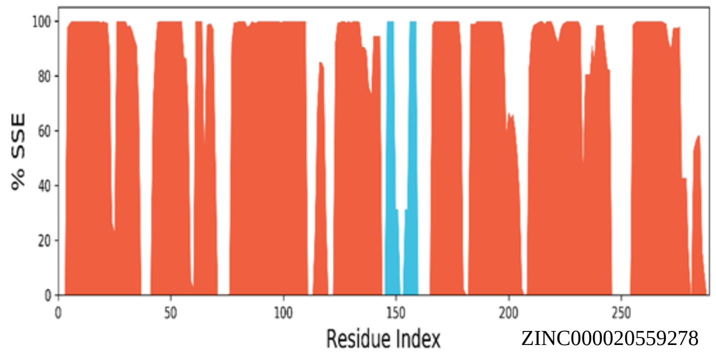

ZINC000020559278

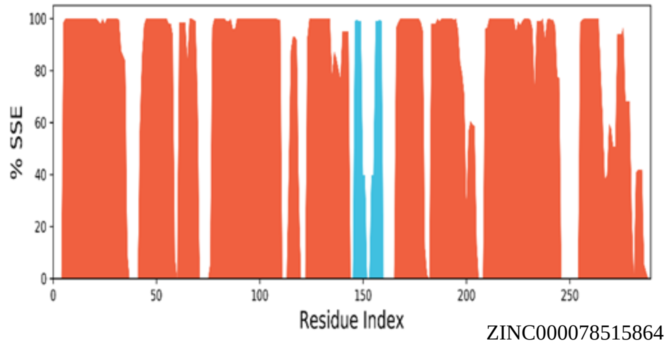

ZINC000078515864

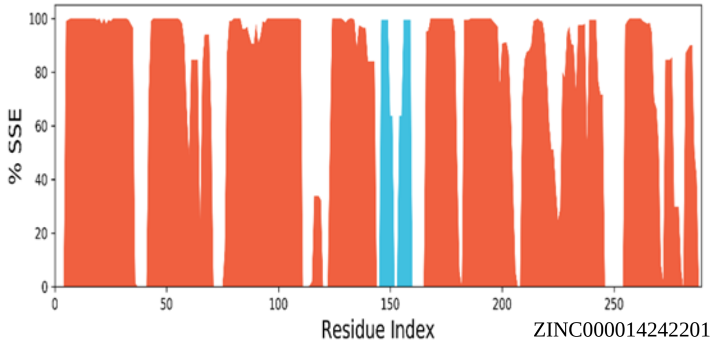

ZINC000014242201

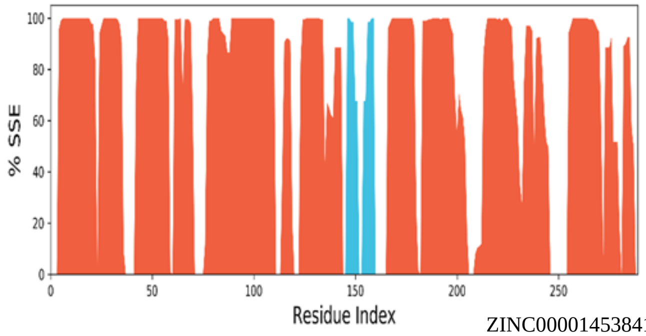

ZINC000014538415

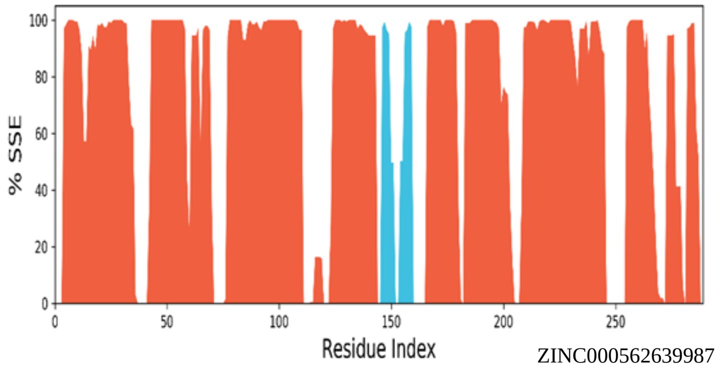

ZINC000562639987

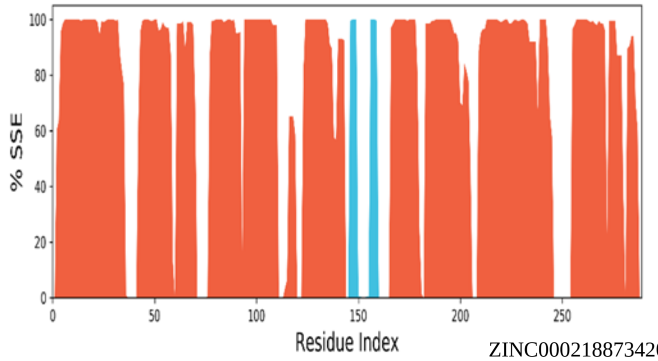

ZINC000218873420

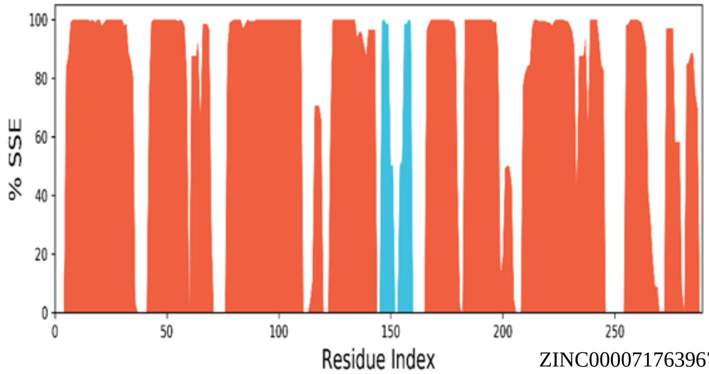

ZINC000071763967

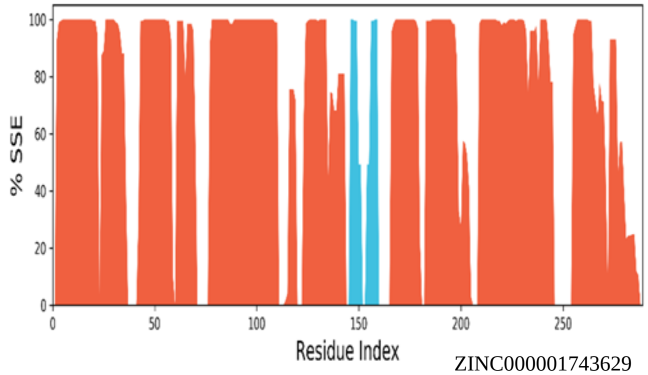

ZINC000001743629

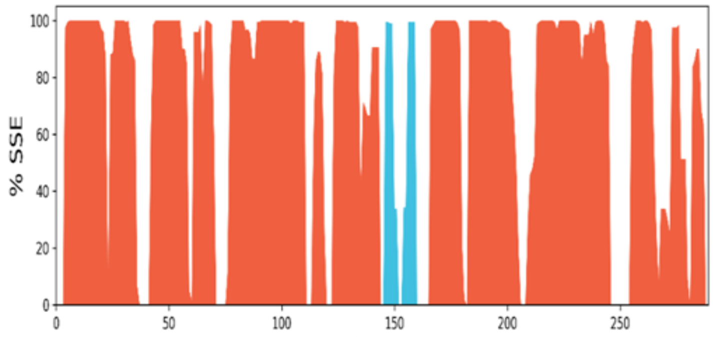

ZINC000067947687

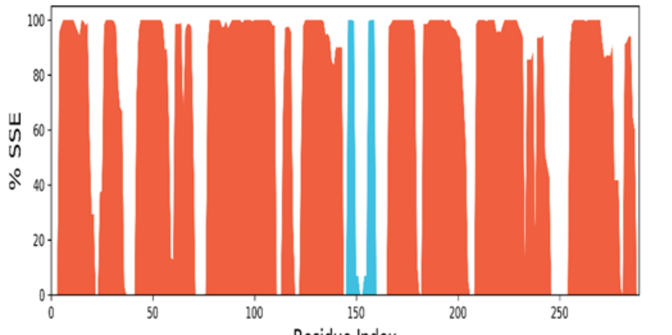

ZINC000028295259

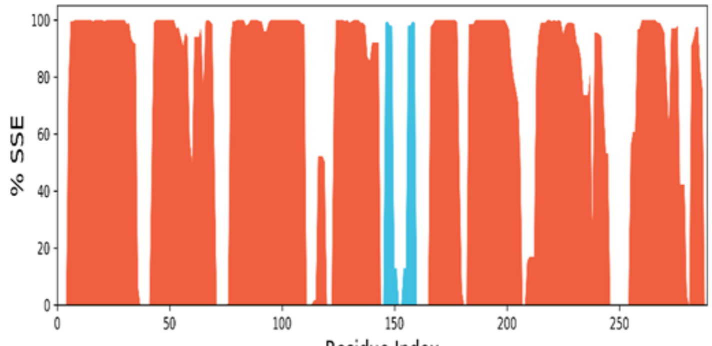

ZINC000634950507

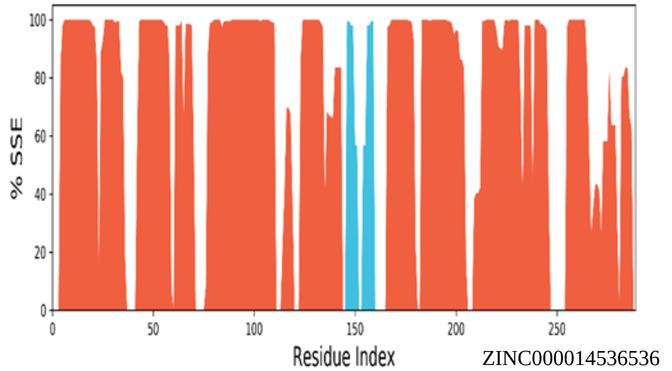

ZINC000014536536

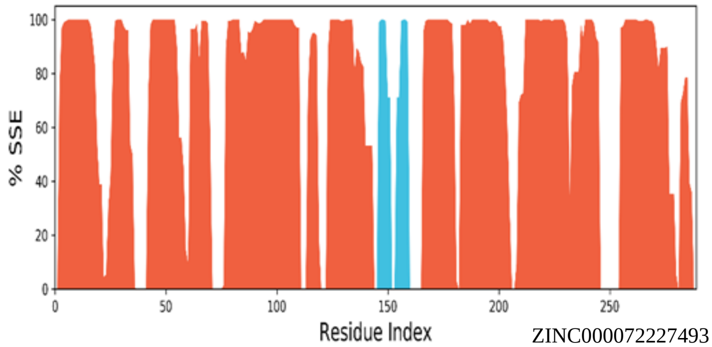

ZINC000072227493

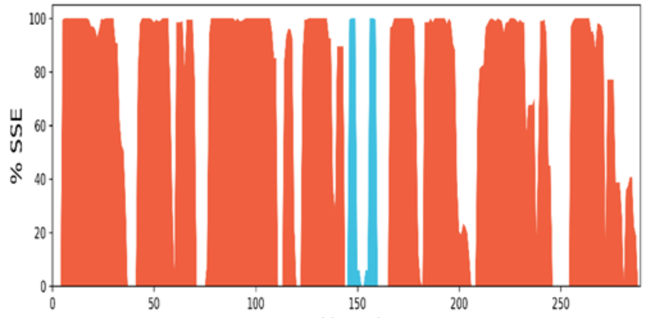

ZINC000147095213

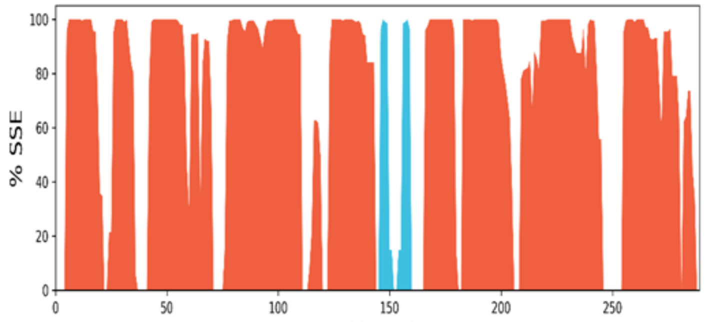

ZINC000257262766

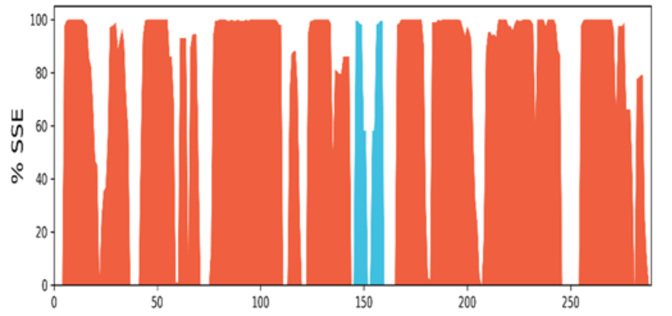

ZINC000095698957

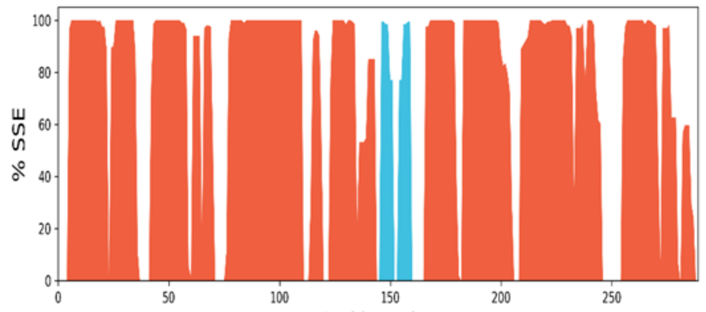

ZINC000006645700

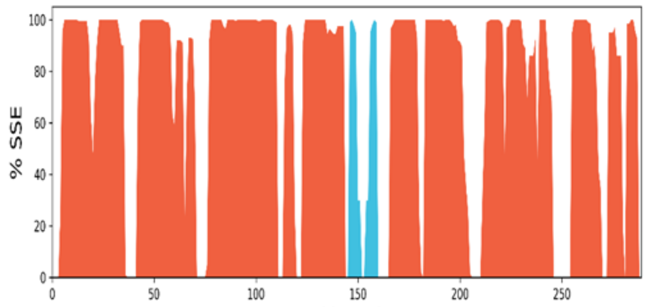

ZINC000014888594

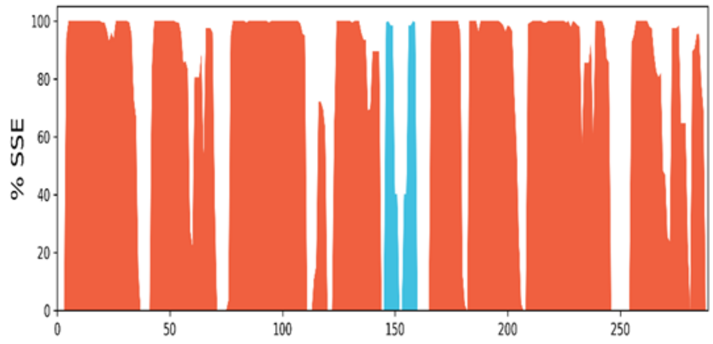

ZINC000082157638

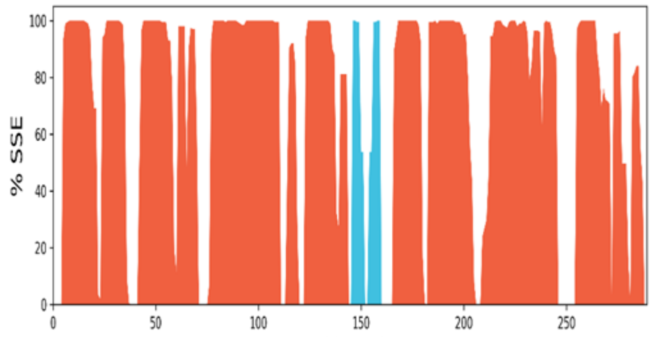

ZINC000015080658

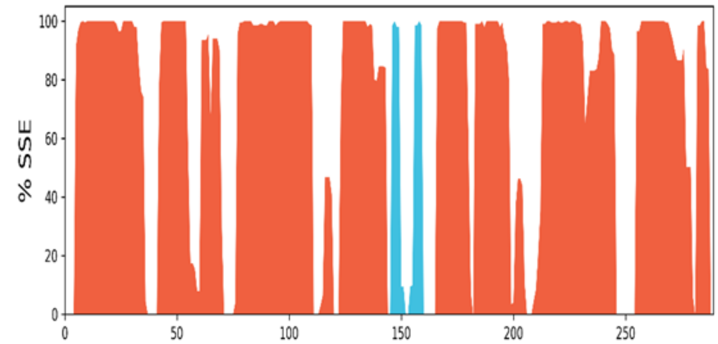

ZINC000091782363

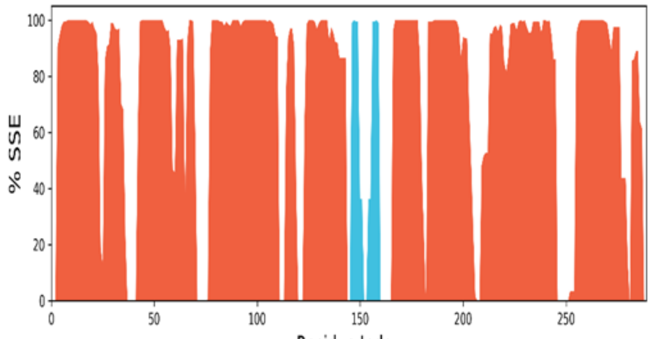

ZINC000096027748

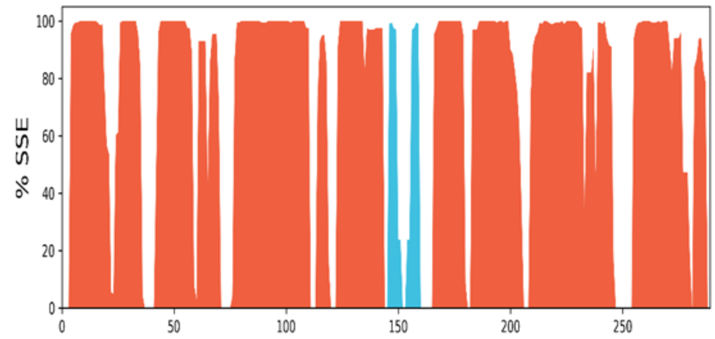

ZINC000091595762

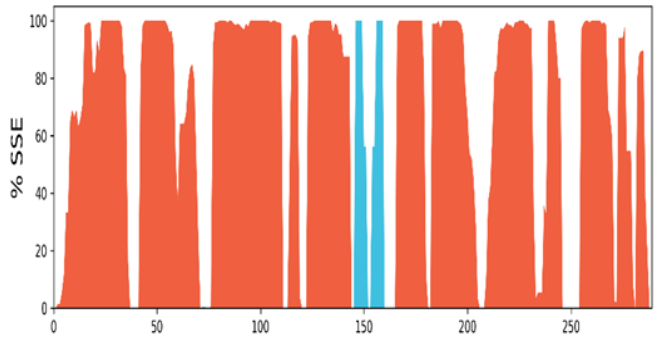

ZINC000067675691

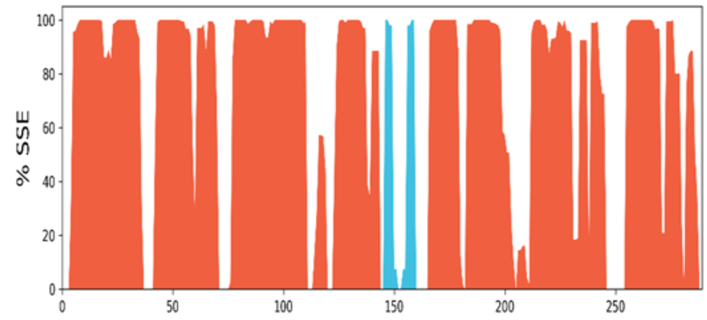

ZINC000067558896

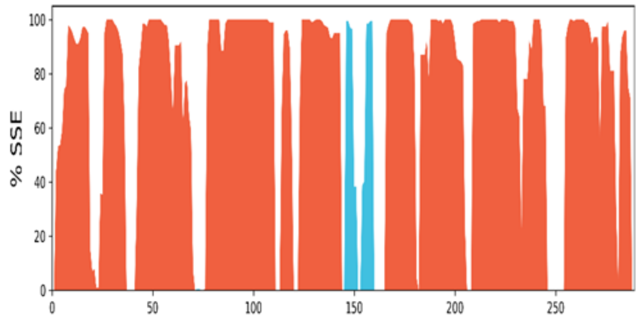

ZINC000011691661

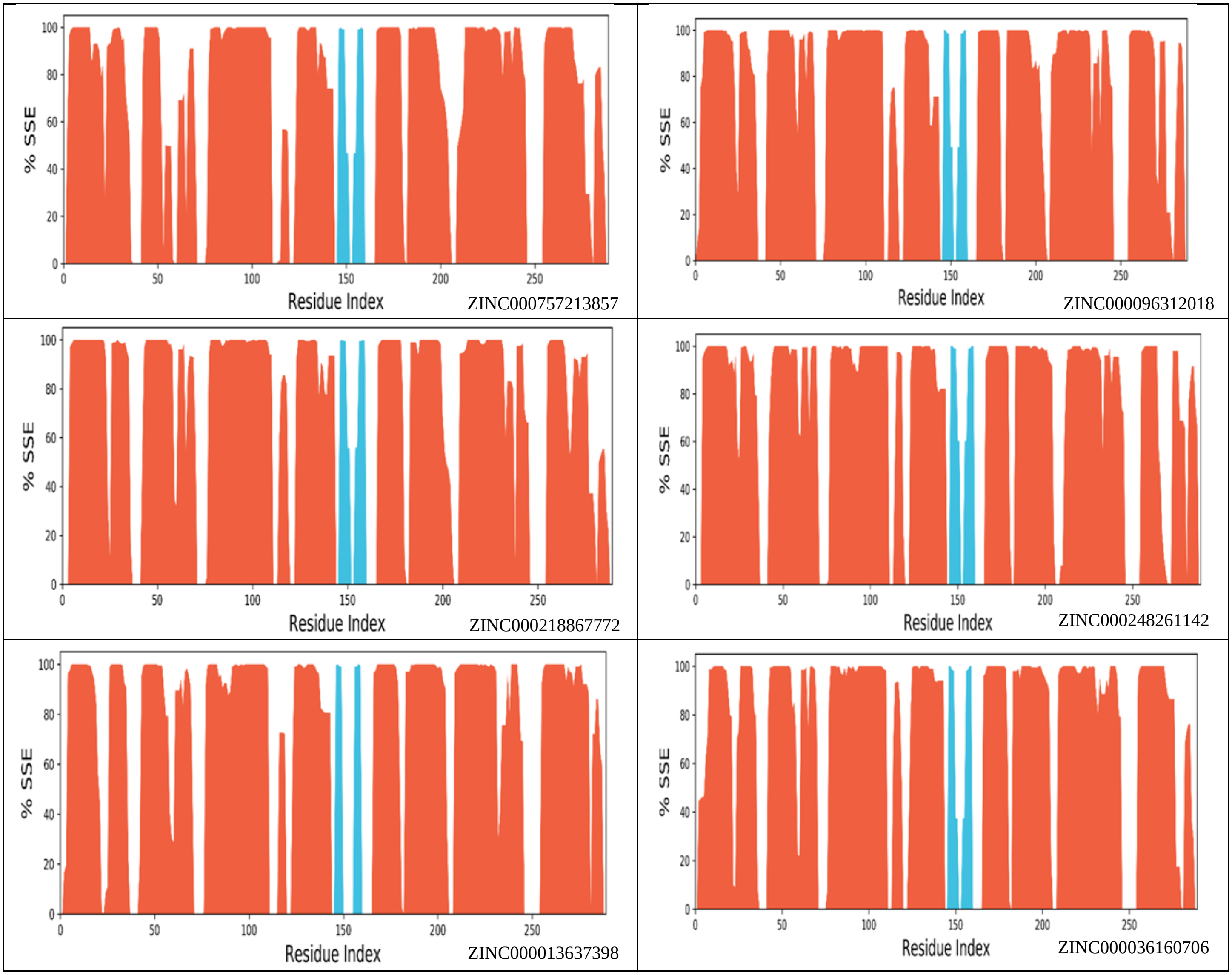

Figure S18. Protein Secondary Structure elements for the top 32 ZINC compounds for the crystal conformation (PDB ID: 6PT3). Alpha helices are in orange and beta sheets are in blue.

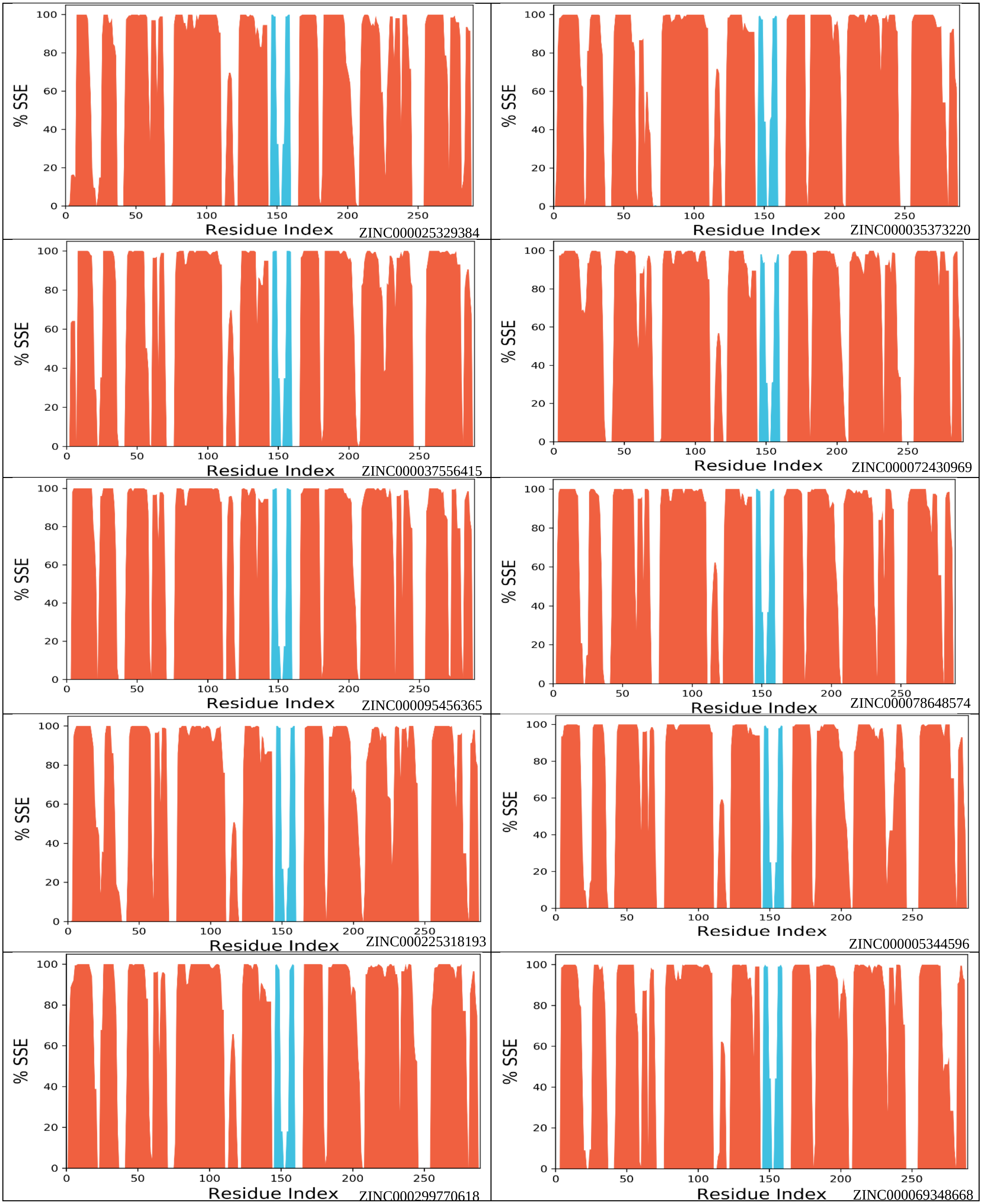
% SSE
Residue Index
ZINC000025329384
ZINC000035373220
ZINC000037556415
ZINC000072430969
ZINC000095456365
ZINC000078648574
ZINC000225318193
ZINC000005344596
ZINC000299770618
ZINC000069348668

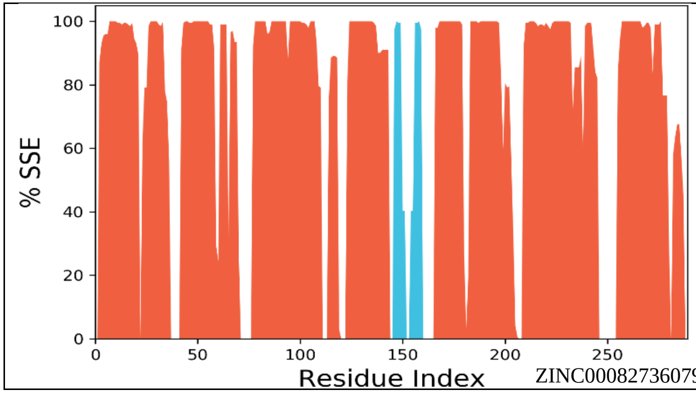

ZINC000827360794

Figure S19. Protein Secondary Structure elements for the top 11 ZINC compounds for the first representative structure from the crystal conformation (PDB ID: 6PT3). Alpha helices are in orange and beta sheets are in blue.

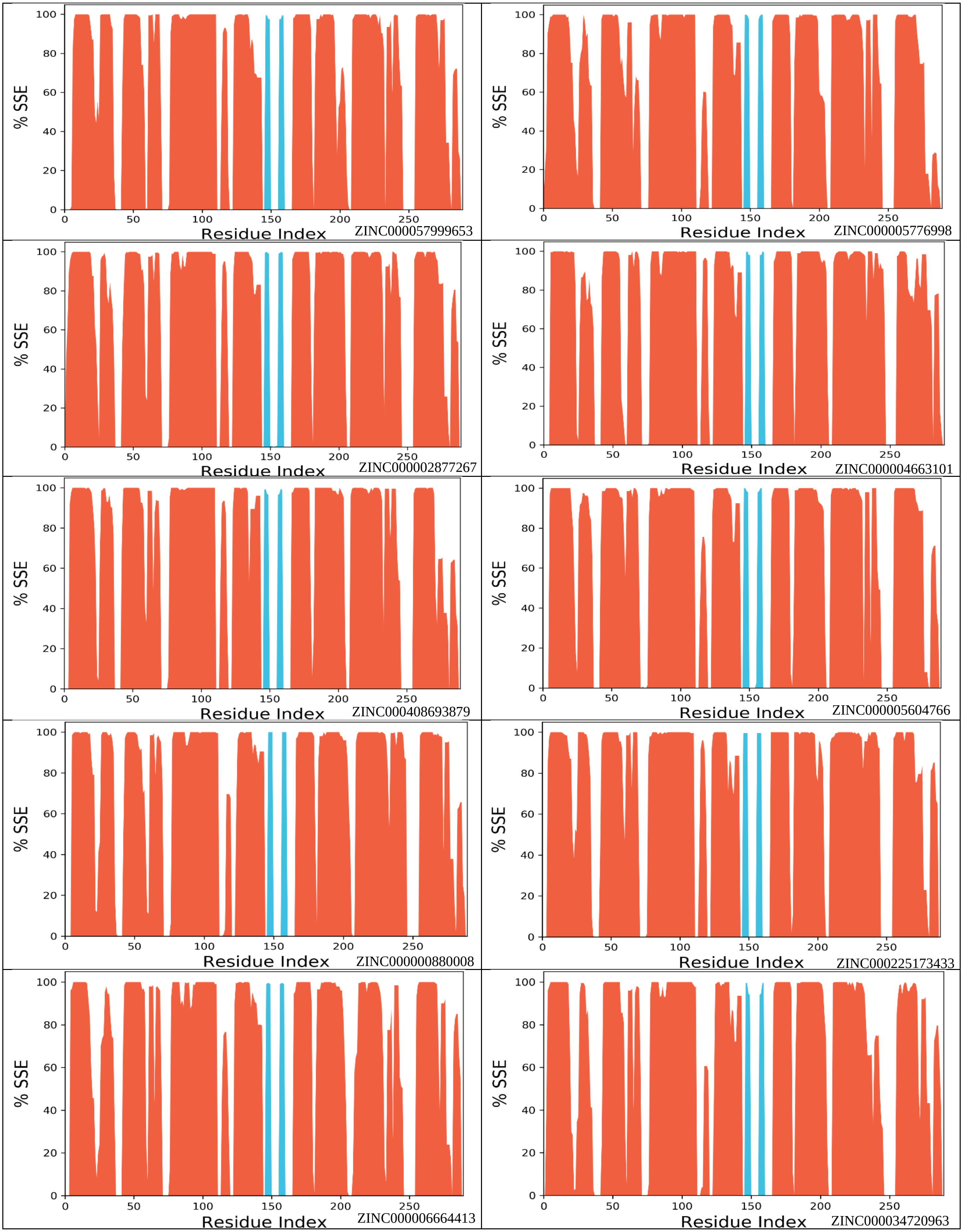
% SSE
Residue Index
ZINC000057999653
ZINC000005776998
ZINC000002877267
ZINC000004663101
ZINC000408693879
ZINC000005604766
ZINC000000880008
ZINC000225173433
ZINC000006664413
ZINC000034720963

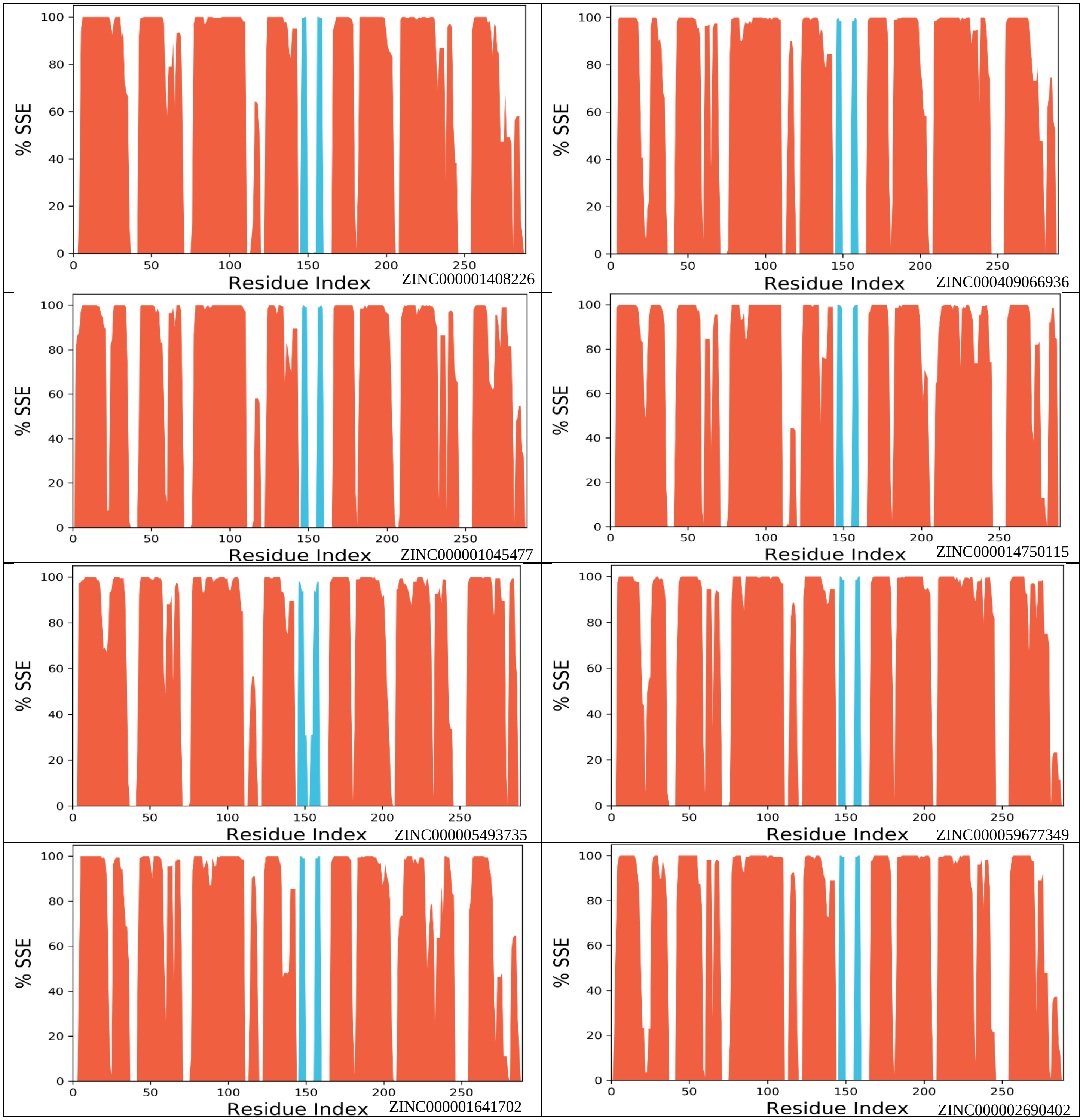
% SSE
Residue Index
ZINC000001408226
ZINC000409066936
ZINC000001045477
ZINC000014750115
ZINC000005493735
ZINC000059677349
ZINC000001641702
ZINC000002690402

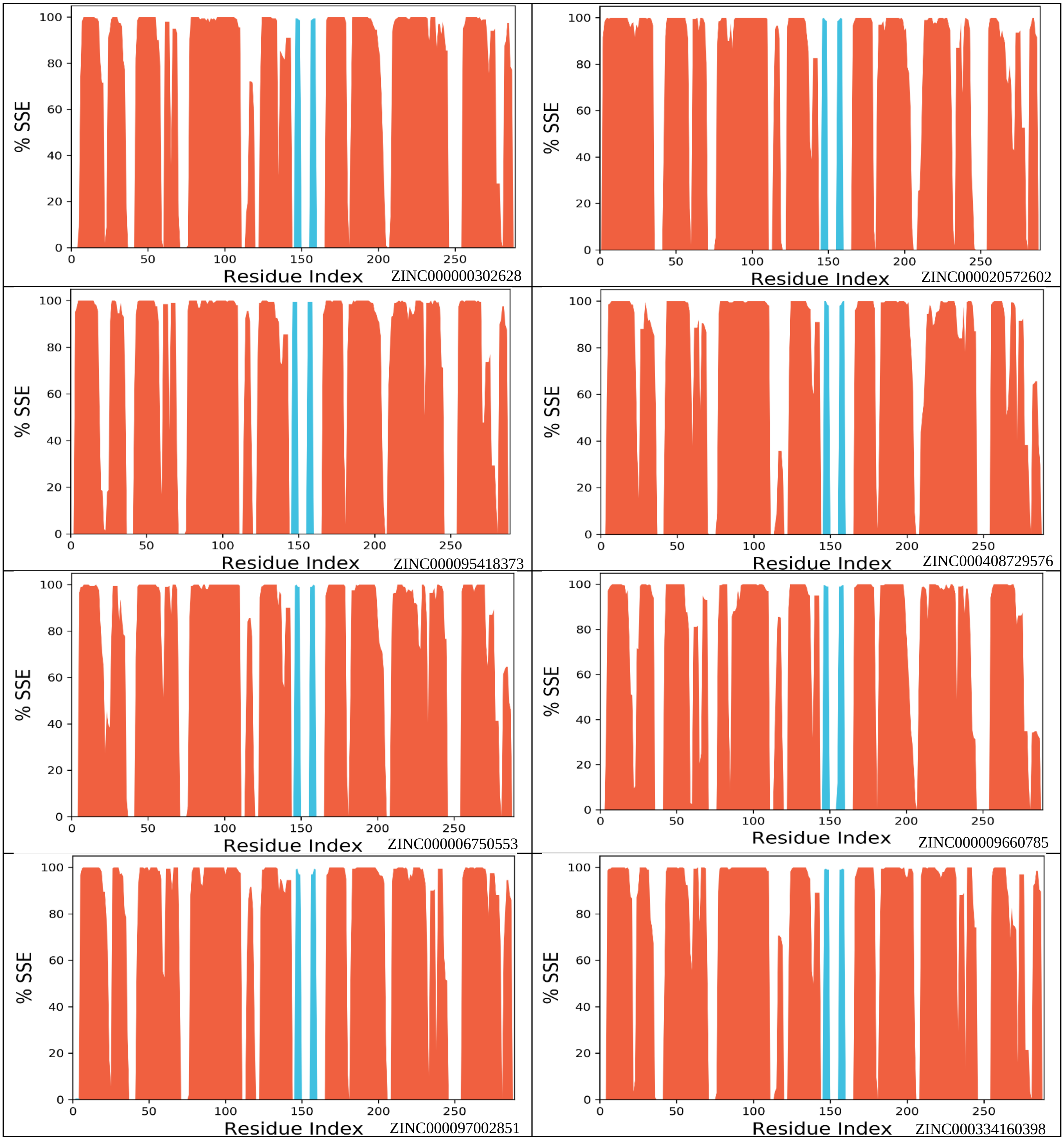

Figure S20. Protein Secondary Structure elements for the top 26 ZINC compounds for the second representative structure from the crystal conformation (PDB ID: 6PT3). Alpha helices are in orange and beta sheets are in blue.

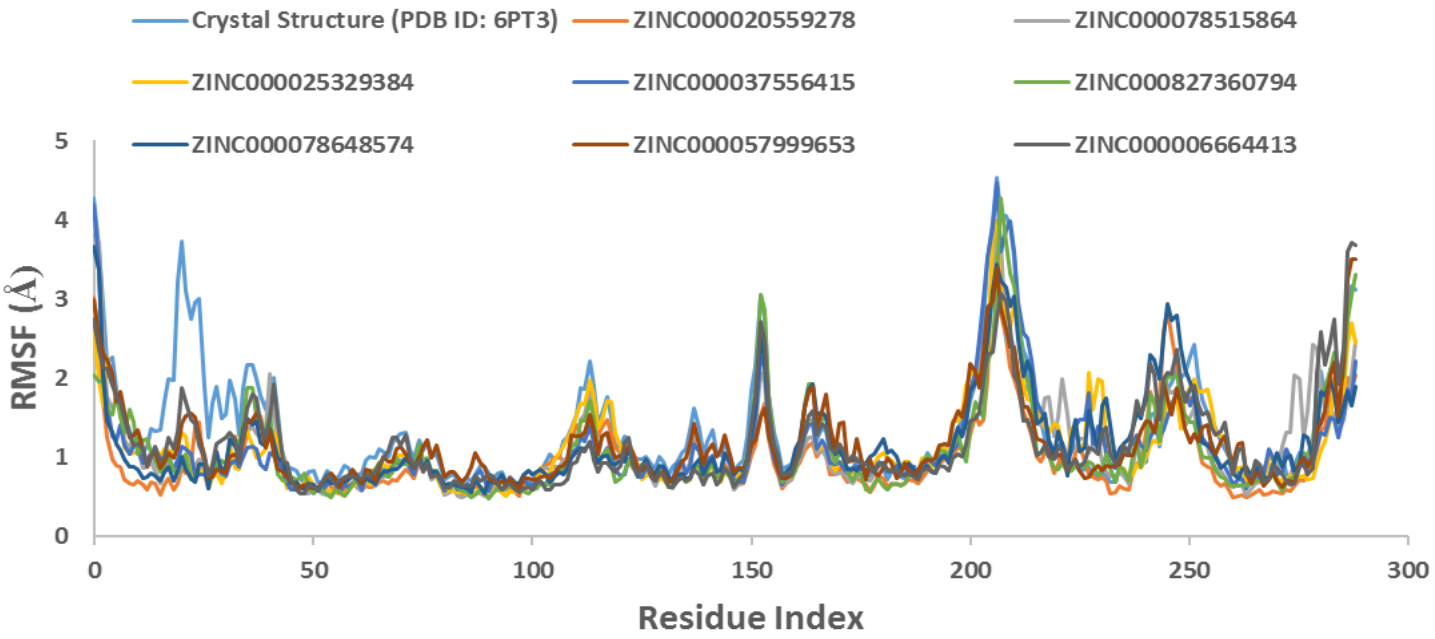

Figure S21. The Cα root mean square fluctuation (RMSF) of the receptor in complex with the top 8 ZINC compounds. The crystal ligand complex is included as the reference.

Ref. Compound	6PT3	CCN(CC)C(=O)c1ccc(cc1)[C@@H](N2C[C@@H](C)N(C[C@@H]2C)Cc3ccccc3)c4cccc(O)c4

Molecule 1

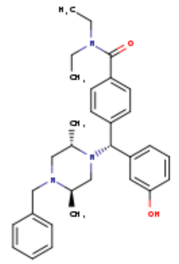

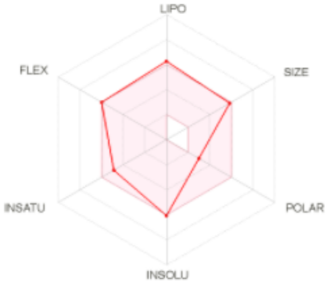

SMILES CCN(C(=O)c1ccc(cc1)[C@@H](N1C[C@@H](C)N(C[C@@H]1C)Cc1ccccc1)c1cccc(c1)O)CC

Physicochemical Properties	
Formula	C31H39N3O2
Molecular weight	485.66 g/mol
Num. heavy atoms	36
Num. arom. heavy atoms	18
Fraction Csp3	0.39
Num. rotatable bonds	9
Num. H-bond acceptors	4
Num. H-bond donors	1
Molar Refractivity	155.07
TPSA	47.02 Å²

Lipophilicity	
Log $P_{o/w}$ (iLOGP)	4.51
Log $P_{o/w}$ (XLOGP3)	5.46
Log $P_{o/w}$ (WLOGP)	4.32
Log $P_{o/w}$ (MLOGP)	3.80
Log $P_{o/w}$ (SILICOS-IT)	4.72
Consensus Log $P_{o/w}$	4.56

Water Solubility	
Log *S* (ESOL)	-6.07
Solubility	4.16e-04 mg/ml ; 8.57e-07 mol/l
Class	Poorly soluble
Log *S* (Ali)	-6.20
Solubility	3.03e-04 mg/ml ; 6.25e-07 mol/l
Class	Poorly soluble
Log *S* (SILICOS-IT)	-8.11
Solubility	3.76e-06 mg/ml ; 7.74e-09 mol/l
Class	Poorly soluble

Pharmacokinetics	
GI absorption	High
BBB permeant	Yes
P-gp substrate	Yes
CYP1A2 inhibitor	No
CYP2C19 inhibitor	No
CYP2C9 inhibitor	No
CYP2D6 inhibitor	Yes
CYP3A4 inhibitor	Yes
Log K_p (skin permeation)	-5.39 cm/s

Druglikeness	
Lipinski	Yes; 0 violation
Ghose	No; 3 violations: MW>480, MR>130, #atoms>70
Veber	Yes
Egan	Yes
Muegge	No; 1 violation: XLOGP3>5
Bioavailability Score	0.55

Medicinal Chemistry	
PAINS	0 alert
Brenk	0 alert
Leadlikeness	No; 3 violations: MW>350, Rotors>7, XLOGP3>3.5
Synthetic accessibility	4.20

1	6PT3	C[C@H](c1ccc2c(c1)[nH]c(=O)[nH]2)[NH2+]Cc3ccccc3Cl

Molecule 1

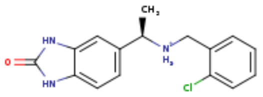

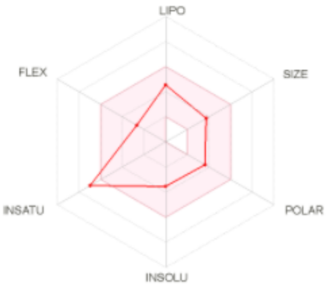

SMILES Clc1ccccc1C[NH2+][C@@H](c1ccc2c(c1)[nH]c(=O)[nH]2)C

Physicochemical Properties	
Formula	C16H17ClN3O
Molecular weight	302.78 g/mol
Num. heavy atoms	21
Num. arom. heavy atoms	15
Fraction Csp3	0.19
Num. rotatable bonds	4
Num. H-bond acceptors	1
Num. H-bond donors	3
Molar Refractivity	87.06
TPSA	65.26 Å²

Lipophilicity	
Log $P_{o/w}$ (iLOGP)	2.60
Log $P_{o/w}$ (XLOGP3)	2.46
Log $P_{o/w}$ (WLOGP)	1.86
Log $P_{o/w}$ (MLOGP)	-0.86
Log $P_{o/w}$ (SILICOS-IT)	4.41
Consensus Log $P_{o/w}$	2.09

Water Solubility	
Log *S* (ESOL)	-3.53
Solubility	8.90e-02 mg/ml ; 2.94e-04 mol/l
Class	Soluble
Log *S* (Ali)	-3.47
Solubility	1.02e-01 mg/ml ; 3.35e-04 mol/l
Class	Soluble
Log *S* (SILICOS-IT)	-6.83
Solubility	4.46e-05 mg/ml ; 1.47e-07 mol/l
Class	Poorly soluble

Pharmacokinetics	
GI absorption	High
BBB permeant	Yes
P-gp substrate	No
CYP1A2 inhibitor	Yes
CYP2C19 inhibitor	No
CYP2C9 inhibitor	No
CYP2D6 inhibitor	Yes
CYP3A4 inhibitor	Yes
Log K_p (skin permeation)	-6.40 cm/s

Druglikeness	
Lipinski	Yes; 0 violation
Ghose	Yes
Veber	Yes
Egan	Yes
Muegge	Yes
Bioavailability Score	0.55

Medicinal Chemistry	
PAINS	0 alert
Brenk	0 alert
Leadlikeness	Yes
Synthetic accessibility	2.55

2	6PT3	C[C@H](Sc1nnc([C@H]2COc3ccccc3O2)o1)C(=O)Nc1ccc2[nH]c(=O)[nH]c2c1

Molecule 2

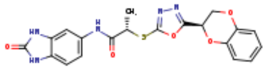

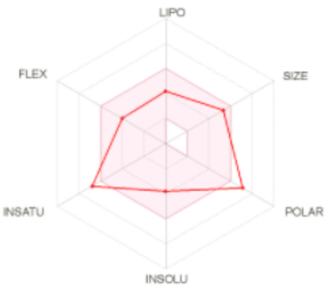

SMILES O=C([C@@H](Sc1nnc(o1)[C@H]1COc2c(O1)cccc2)C)Nc1ccc2c(c1)[nH]c(=O)[nH]2

Physicochemical Properties	
Formula	C20H17N5O5S
Molecular weight	439.44 g/mol
Num. heavy atoms	31
Num. arom. heavy atoms	20
Fraction Csp3	0.20
Num. rotatable bonds	6
Num. H-bond acceptors	7
Num. H-bond donors	3
Molar Refractivity	112.49
TPSA	160.43 Å²

Lipophilicity	
Log $P_{o/w}$ (iLOGP)	2.56
Log $P_{o/w}$ (XLOGP3)	1.82
Log $P_{o/w}$ (WLOGP)	2.36
Log $P_{o/w}$ (MLOGP)	1.33
Log $P_{o/w}$ (SILICOS-IT)	2.92
Consensus Log $P_{o/w}$	2.20

Water Solubility	
Log *S* (ESOL)	-3.79
Solubility	7.08e-02 mg/ml ; 1.61e-04 mol/l
Class	Soluble
Log *S* (Ali)	-4.81
Solubility	6.82e-03 mg/ml ; 1.55e-05 mol/l
Class	Moderately soluble
Log *S* (SILICOS-IT)	-6.55
Solubility	1.22e-04 mg/ml ; 2.79e-07 mol/l
Class	Poorly soluble

Pharmacokinetics	
GI absorption	Low
BBB permeant	No
P-gp substrate	Yes
CYP1A2 inhibitor	No
CYP2C19 inhibitor	No
CYP2C9 inhibitor	No
CYP2D6 inhibitor	No
CYP3A4 inhibitor	Yes
Log K_p (skin permeation)	-7.69 cm/s

Druglikeness	
Lipinski	Yes; 0 violation
Ghose	Yes
Veber	No; 1 violation: TPSA>140
Egan	No; 1 violation: TPSA>131.6
Muegge	No; 1 violation: TPSA>150
Bioavailability Score	0.55

Medicinal Chemistry	
PAINS	0 alert
Brenk	0 alert
Leadlikeness	No; 1 violation: MW>350
Synthetic accessibility	4.40

3	6PT3	Cc1cccc(C)c1C(=O)N3CC[NH+]([C@H]2CCNC(=O)CC2)CC3

Molecule 3

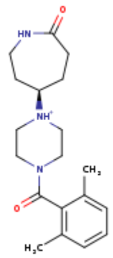

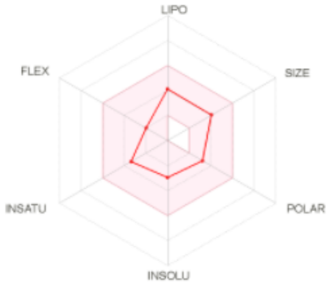

SMILES O=C1NCC[C@@H](CC1)[NH+]1CCN(CC1)C(=O)c1c(C)cccc1C

Physicochemical Properties	
Formula	C19H28N3O2
Molecular weight	330.44 g/mol
Num. heavy atoms	24
Num. arom. heavy atoms	6
Fraction Csp3	0.58
Num. rotatable bonds	3
Num. H-bond acceptors	2
Num. H-bond donors	2
Molar Refractivity	107.10
TPSA	53.85 Å²

Lipophilicity	
Log $P_{o/w}$ (iLOGP)	2.81
Log $P_{o/w}$ (XLOGP3)	1.73
Log $P_{o/w}$ (WLOGP)	-0.83
Log $P_{o/w}$ (MLOGP)	-1.84
Log $P_{o/w}$ (SILICOS-IT)	2.53
Consensus Log $P_{o/w}$	0.88

Water Solubility	
Log *S* (ESOL)	-2.97
Solubility	3.58e-01 mg/ml ; 1.08e-03 mol/l
Class	Soluble
Log *S* (Ali)	-2.48
Solubility	1.10e+00 mg/ml ; 3.33e-03 mol/l
Class	Soluble
Log *S* (SILICOS-IT)	-4.25
Solubility	1.85e-02 mg/ml ; 5.58e-05 mol/l
Class	Moderately soluble

Pharmacokinetics	
GI absorption	High
BBB permeant	No
P-gp substrate	Yes
CYP1A2 inhibitor	No
CYP2C19 inhibitor	No
CYP2C9 inhibitor	No
CYP2D6 inhibitor	No
CYP3A4 inhibitor	No
Log K_p (skin permeation)	-7.09 cm/s

Druglikeness	
Lipinski	Yes; 0 violation
Ghose	No; 1 violation: WLOGP<-0.4
Veber	Yes
Egan	Yes
Muegge	Yes
Bioavailability Score	0.55

Medicinal Chemistry	
PAINS	0 alert
Brenk	0 alert
Leadlikeness	Yes
Synthetic accessibility	3.17

4	6PT3	CC(C)[C@H]2C[NH+](Cc1ccc(O)c(O)c1)CCC(=O)N2Cc3ccc(F)cc3

Molecule 4

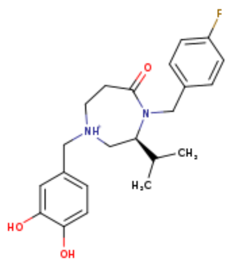

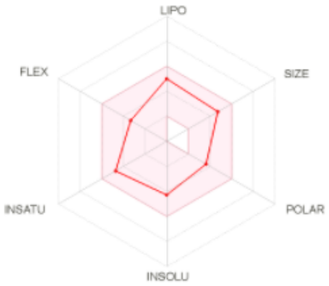

SMILES Fc1ccc(cc1)CN1C(=O)CC[NH+](C[C@@H]1C(C)C)Cc1ccc(c(c1)O)O

Physicochemical Properties	
Formula	C22H28FN2O3
Molecular weight	387.47 g/mol
Num. heavy atoms	28
Num. arom. heavy atoms	12
Fraction Csp3	0.41
Num. rotatable bonds	5
Num. H-bond acceptors	4
Num. H-bond donors	3
Molar Refractivity	115.83
TPSA	65.21 Å²

Lipophilicity	
Log $P_{o/w}$ (iLOGP)	3.16
Log $P_{o/w}$ (XLOGP3)	3.24
Log $P_{o/w}$ (WLOGP)	1.43
Log $P_{o/w}$ (MLOGP)	-1.10
Log $P_{o/w}$ (SILICOS-IT)	3.36
Consensus Log $P_{o/w}$	2.02

Water Solubility	
Log *S* (ESOL)	-4.27
Solubility	2.08e-02 mg/ml ; 5.36e-05 mol/l
Class	Moderately soluble
Log *S* (Ali)	-4.28
Solubility	2.02e-02 mg/ml ; 5.21e-05 mol/l
Class	Moderately soluble
Log *S* (SILICOS-IT)	-5.33
Solubility	1.82e-03 mg/ml ; 4.69e-06 mol/l
Class	Moderately soluble

Pharmacokinetics	
GI absorption	High
BBB permeant	Yes
P-gp substrate	Yes
CYP1A2 inhibitor	No
CYP2C19 inhibitor	No
CYP2C9 inhibitor	No
CYP2D6 inhibitor	No
CYP3A4 inhibitor	No
Log K_p (skin permeation)	-6.36 cm/s

Druglikeness	
Lipinski	Yes; 0 violation
Ghose	Yes
Veber	Yes
Egan	Yes
Muegge	Yes
Bioavailability Score	0.55

Medicinal Chemistry	
PAINS	1 alert: catechol_A
Brenk	1 alert: catechol
Leadlikeness	No; 1 violation: MW>350
Synthetic accessibility	3.09

5	6PT3	C[NH+](Cc1ccc(O)c(O)c1)Cc3cnc2ccccc2c3

Molecule 5

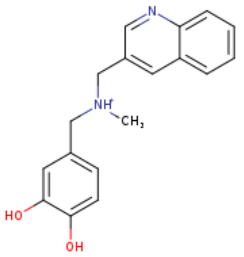

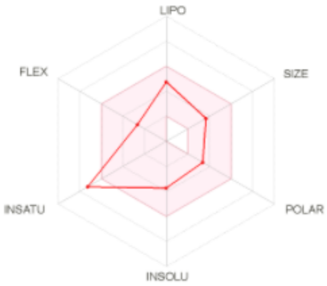

SMILES C[NH+](Cc1ccc(c(c1)O)O)Cc1cnc2c(c1)cccc2

Physicochemical Properties	
Formula	C18H19N2O2
Molecular weight	295.36 g/mol
Num. heavy atoms	22
Num. arom. heavy atoms	16
Fraction Csp3	0.17
Num. rotatable bonds	4
Num. H-bond acceptors	3
Num. H-bond donors	3
Molar Refractivity	89.01
TPSA	57.79 Å²

Lipophilicity	
Log $P_{o/w}$ (iLOGP)	2.48
Log $P_{o/w}$ (XLOGP3)	2.81
Log $P_{o/w}$ (WLOGP)	1.56
Log $P_{o/w}$ (MLOGP)	-1.80
Log $P_{o/w}$ (SILICOS-IT)	2.88
Consensus Log $P_{o/w}$	1.59

Water Solubility	
Log *S* (ESOL)	-3.72
Solubility	5.68e-02 mg/ml ; 1.92e-04 mol/l
Class	Soluble
Log *S* (Ali)	-3.68
Solubility	6.16e-02 mg/ml ; 2.09e-04 mol/l
Class	Soluble
Log *S* (SILICOS-IT)	-5.59
Solubility	7.56e-04 mg/ml ; 2.56e-06 mol/l
Class	Moderately soluble

Pharmacokinetics	
GI absorption	High
BBB permeant	Yes
P-gp substrate	No
CYP1A2 inhibitor	Yes
CYP2C19 inhibitor	No
CYP2C9 inhibitor	No
CYP2D6 inhibitor	Yes
CYP3A4 inhibitor	No
Log K_p (skin permeation)	-6.11 cm/s

Druglikeness	
Lipinski	Yes; 0 violation
Ghose	Yes
Veber	Yes
Egan	Yes
Muegge	Yes
Bioavailability Score	0.55

Medicinal Chemistry	
PAINS	1 alert: catechol_A
Brenk	1 alert: catechol
Leadlikeness	Yes
Synthetic accessibility	1.98

6	6PT3	O=C3CC[C@@H]([NH2+]CCCn2ccc1ccccc12)CCN3

Molecule 1

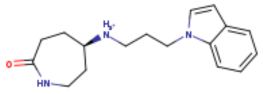

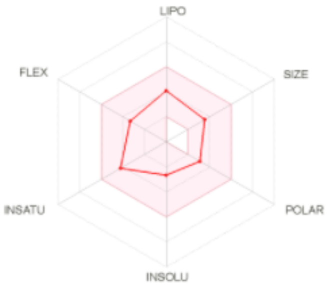

SMILES O=C1NCC[C@@H](CC1)[NH2+]CCCn1ccc2c1cccc2

Physicochemical Properties	
Formula	C17H24N3O
Molecular weight	286.39 g/mol
Num. heavy atoms	21
Num. arom. heavy atoms	9
Fraction Csp3	0.47
Num. rotatable bonds	5
Num. H-bond acceptors	1
Num. H-bond donors	2
Molar Refractivity	90.52
TPSA	50.64 Å²

Lipophilicity	
Log $P_{o/w}$ (iLOGP)	2.69
Log $P_{o/w}$ (XLOGP3)	1.66
Log $P_{o/w}$ (WLOGP)	0.88
Log $P_{o/w}$ (MLOGP)	-1.97
Log $P_{o/w}$ (SILICOS-IT)	2.40
Consensus Log $P_{o/w}$	1.13

Water Solubility	
Log *S* (ESOL)	-2.65
Solubility	6.43e-01 mg/ml ; 2.25e-03 mol/l
Class	Soluble
Log *S* (Ali)	-2.34
Solubility	1.32e+00 mg/ml ; 4.60e-03 mol/l
Class	Soluble
Log *S* (SILICOS-IT)	-5.10
Solubility	2.30e-03 mg/ml ; 8.02e-06 mol/l
Class	Moderately soluble

Pharmacokinetics	
GI absorption	High
BBB permeant	Yes
P-gp substrate	Yes
CYP1A2 inhibitor	No
CYP2C19 inhibitor	No
CYP2C9 inhibitor	No
CYP2D6 inhibitor	No
CYP3A4 inhibitor	No
Log K_p (skin permeation)	-6.87 cm/s

Druglikeness	
Lipinski	Yes; 0 violation
Ghose	Yes
Veber	Yes
Egan	Yes
Muegge	Yes
Bioavailability Score	0.55

Medicinal Chemistry	
PAINS	0 alert
Brenk	0 alert
Leadlikeness	Yes
Synthetic accessibility	2.63

7	6PT3	Cc3ccc(N1C(=O)[C@H]([NH3+])[C@@H]1c2ccc(Cl)cc2Cl)cc3

Molecule 2

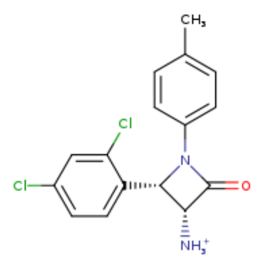

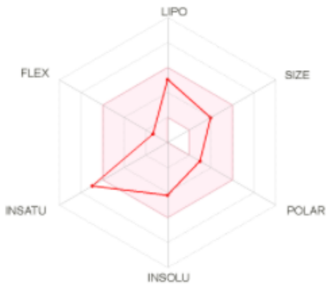

SMILES Cc1ccc(cc1)N1C(=O)[C@@H]([C@@H]1c1ccc(cc1Cl)Cl)[NH3+]

Physicochemical Properties	
Formula	C16H15Cl2N2O
Molecular weight	322.21 g/mol
Num. heavy atoms	21
Num. arom. heavy atoms	12
Fraction Csp3	0.19
Num. rotatable bonds	2
Num. H-bond acceptors	1
Num. H-bond donors	1
Molar Refractivity	89.92
TPSA	47.95 Å²

Lipophilicity	
Log $P_{o/w}$ (iLOGP)	2.93
Log $P_{o/w}$ (XLOGP3)	3.33
Log $P_{o/w}$ (WLOGP)	2.29
Log $P_{o/w}$ (MLOGP)	0.35
Log $P_{o/w}$ (SILICOS-IT)	3.49
Consensus Log $P_{o/w}$	2.48

Water Solubility	
Log *S* (ESOL)	-4.23
Solubility	1.91e-02 mg/ml ; 5.94e-05 mol/l
Class	Moderately soluble
Log *S* (Ali)	-4.01
Solubility	3.12e-02 mg/ml ; 9.69e-05 mol/l
Class	Moderately soluble
Log *S* (SILICOS-IT)	-5.75
Solubility	5.77e-04 mg/ml ; 1.79e-06 mol/l
Class	Moderately soluble

Pharmacokinetics	
GI absorption	High
BBB permeant	Yes
P-gp substrate	No
CYP1A2 inhibitor	Yes
CYP2C19 inhibitor	Yes
CYP2C9 inhibitor	Yes
CYP2D6 inhibitor	No
CYP3A4 inhibitor	No
Log K_p (skin permeation)	-5.90 cm/s

Druglikeness	
Lipinski	Yes; 0 violation
Ghose	Yes
Veber	Yes
Egan	Yes
Muegge	Yes
Bioavailability Score	0.55

Medicinal Chemistry	
PAINS	0 alert
Brenk	0 alert
Leadlikeness	Yes
Synthetic accessibility	2.89

8	6PT3	CN(Cc1ccccc1)[C@@H]3[C@@H](O)[C@H](Oc2cccc(Cl)c2)C[C@H]3 NC(=O)c4ccc(C(=O)[O-])cc4

Molecule 3

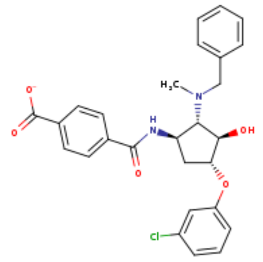

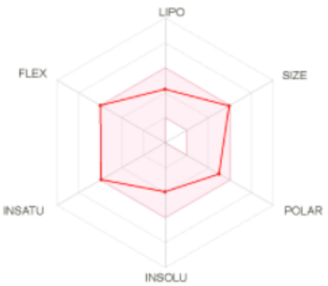

SMILES Clc1cccc(c1)O[C@@H]1C[C@H]([C@@H]([C@H]1O)N(Cc1ccccc1)C)NC(=O)c1ccc(cc1)C(=O)[O-]

Physicochemical Properties	
Formula	C27H26ClN2O5
Molecular weight	493.96 g/mol
Num. heavy atoms	35
Num. arom. heavy atoms	18
Fraction Csp3	0.26
Num. rotatable bonds	9
Num. H-bond acceptors	6
Num. H-bond donors	2
Molar Refractivity	130.75
TPSA	101.93 Å²

Lipophilicity	
Log $P_{o/w}$ (iLOGP)	3.30
Log $P_{o/w}$ (XLOGP3)	1.98
Log $P_{o/w}$ (WLOGP)	2.36
Log $P_{o/w}$ (MLOGP)	2.88
Log $P_{o/w}$ (SILICOS-IT)	3.10
Consensus Log $P_{o/w}$	2.72

Water Solubility	
Log *S* (ESOL)	-3.94
Solubility	5.72e-02 mg/ml ; 1.16e-04 mol/l
Class	Soluble
Log *S* (Ali)	-3.75
Solubility	8.86e-02 mg/ml ; 1.79e-04 mol/l
Class	Soluble
Log *S* (SILICOS-IT)	-7.01
Solubility	4.84e-05 mg/ml ; 9.79e-08 mol/l
Class	Poorly soluble

Pharmacokinetics	
GI absorption	High
BBB permeant	No
P-gp substrate	Yes
CYP1A2 inhibitor	No
CYP2C19 inhibitor	No
CYP2C9 inhibitor	No
CYP2D6 inhibitor	Yes
CYP3A4 inhibitor	Yes
Log K_p (skin permeation)	-7.91 cm/s

Druglikeness	
Lipinski	Yes; 0 violation
Ghose	No; 2 violations: MW>480, MR>130
Veber	Yes
Egan	Yes
Muegge	Yes
Bioavailability Score	0.55

Medicinal Chemistry	
PAINS	0 alert
Brenk	0 alert
Leadlikeness	No; 2 violations: MW>350, Rotors>7
Synthetic accessibility	4.18

9	6PT3	CCc3ccc([C@@H](C[NH3+])c1c[nH]c2ccccc12)cc3

Molecule 4

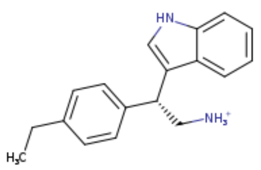

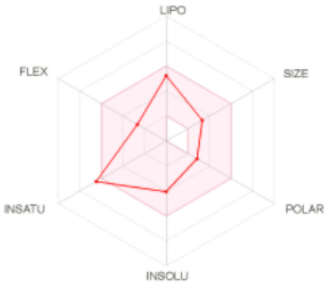

SMILES CCc1ccc(cc1)[C@H](c1c[nH]c2c1cccc2)C[NH3+]

Physicochemical Properties	
Formula	C18H21N2
Molecular weight	265.37 g/mol
Num. heavy atoms	20
Num. arom. heavy atoms	15
Fraction Csp3	0.22
Num. rotatable bonds	4
Num. H-bond acceptors	0
Num. H-bond donors	2
Molar Refractivity	86.30
TPSA	43.43 Å²

Lipophilicity	
Log $P_{o/w}$ (iLOGP)	2.59
Log $P_{o/w}$ (XLOGP3)	3.62
Log $P_{o/w}$ (WLOGP)	3.10
Log $P_{o/w}$ (MLOGP)	-0.57
Log $P_{o/w}$ (SILICOS-IT)	4.65
Consensus Log $P_{o/w}$	2.68

Water Solubility	
Log *S* (ESOL)	-4.06
Solubility	2.33e-02 mg/ml ; 8.77e-05 mol/l
Class	Moderately soluble
Log *S* (Ali)	-4.22
Solubility	1.60e-02 mg/ml ; 6.03e-05 mol/l
Class	Moderately soluble
Log *S* (SILICOS-IT)	-6.69
Solubility	5.44e-05 mg/ml ; 2.05e-07 mol/l
Class	Poorly soluble

Pharmacokinetics	
GI absorption	High
BBB permeant	Yes
P-gp substrate	No
CYP1A2 inhibitor	Yes
CYP2C19 inhibitor	Yes
CYP2C9 inhibitor	No
CYP2D6 inhibitor	Yes
CYP3A4 inhibitor	No
Log K_p (skin permeation)	-5.35 cm/s

Druglikeness	
Lipinski	Yes; 0 violation
Ghose	Yes
Veber	Yes
Egan	Yes
Muegge	Yes
Bioavailability Score	0.55

Medicinal Chemistry	
PAINS	0 alert
Brenk	0 alert
Leadlikeness	No; 1 violation: XLOGP3>3.5
Synthetic accessibility	2.66

10	6PT3	O=c3[nH]cc(C[NH2+][C@@H]2CCOC1(CCOCC1)C2)c(=O)[nH]3

Molecule 5

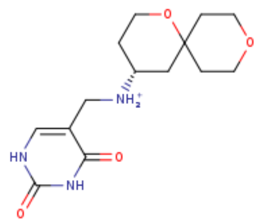

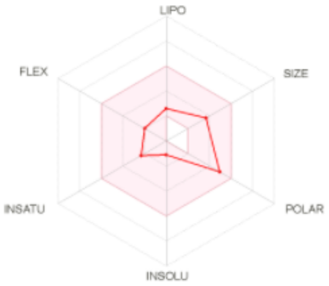

SMILES O=c1[nH]c(=O)[nH]cc1C[NH2+][C@@H]1CCOC2(C1)CCOCC2

Physicochemical Properties	
Formula	C14H22N3O4
Molecular weight	296.34 g/mol
Num. heavy atoms	21
Num. arom. heavy atoms	6
Fraction Csp3	0.71
Num. rotatable bonds	3
Num. H-bond acceptors	4
Num. H-bond donors	3
Molar Refractivity	77.95
TPSA	100.79 Å²

Lipophilicity	
Log $P_{o/w}$ (iLOGP)	1.72
Log $P_{o/w}$ (XLOGP3)	-0.96
Log $P_{o/w}$ (WLOGP)	-1.30
Log $P_{o/w}$ (MLOGP)	-3.72
Log $P_{o/w}$ (SILICOS-IT)	2.28
Consensus Log $P_{o/w}$	-0.40

Water Solubility	
Log *S* (ESOL)	-1.09
Solubility	2.43e+01 mg/ml ; 8.20e-02 mol/l
Class	Very soluble
Log *S* (Ali)	-0.67
Solubility	6.31e+01 mg/ml ; 2.13e-01 mol/l
Class	Very soluble
Log *S* (SILICOS-IT)	-3.65
Solubility	6.62e-02 mg/ml ; 2.23e-04 mol/l
Class	Soluble

Pharmacokinetics	
GI absorption	High
BBB permeant	No
P-gp substrate	No
CYP1A2 inhibitor	No
CYP2C19 inhibitor	No
CYP2C9 inhibitor	No
CYP2D6 inhibitor	No
CYP3A4 inhibitor	No
Log K_p (skin permeation)	-8.79 cm/s

Druglikeness	
Lipinski	Yes; 0 violation
Ghose	No; 1 violation: WLOGP<-0.4
Veber	Yes
Egan	Yes
Muegge	Yes
Bioavailability Score	0.55

Medicinal Chemistry	
PAINS	0 alert
Brenk	0 alert
Leadlikeness	Yes
Synthetic accessibility	3.62

11	6PT3	[NH3+][C@H]2CN(C(=O)CN1CC(=O)NC1=O)C[C@@H]2c3ccccc3

Molecule 6

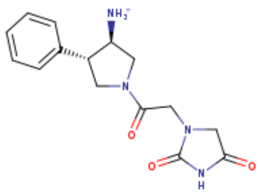

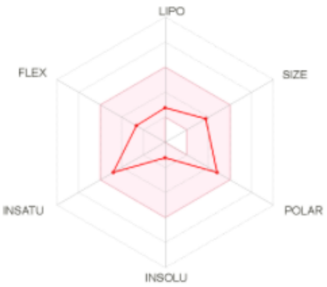

SMILES O=C1CN(C(=O)N1)CC(=O)N1C[C@@H]([C@H](C1)c1ccccc1)[NH3+]

Physicochemical Properties	
Formula	C15H19N4O3
Molecular weight	303.34 g/mol
Num. heavy atoms	22
Num. arom. heavy atoms	6
Fraction Csp3	0.40
Num. rotatable bonds	4
Num. H-bond acceptors	3
Num. H-bond donors	2
Molar Refractivity	90.93
TPSA	97.36 Å²

Lipophilicity	
Log $P_{o/w}$ (iLOGP)	1.20
Log $P_{o/w}$ (XLOGP3)	-0.69
Log $P_{o/w}$ (WLOGP)	-2.37
Log $P_{o/w}$ (MLOGP)	-3.55
Log $P_{o/w}$ (SILICOS-IT)	-0.26
Consensus Log $P_{o/w}$	-1.13

Water Solubility	
Log *S* (ESOL)	-1.22
Solubility	1.81e+01 mg/ml ; 5.97e-02 mol/l
Class	Very soluble
Log *S* (Ali)	-0.88
Solubility	4.00e+01 mg/ml ; 1.32e-01 mol/l
Class	Very soluble
Log *S* (SILICOS-IT)	-2.36
Solubility	1.32e+00 mg/ml ; 4.37e-03 mol/l
Class	Soluble

Pharmacokinetics	
GI absorption	Low
BBB permeant	No
P-gp substrate	No
CYP1A2 inhibitor	No
CYP2C19 inhibitor	No
CYP2C9 inhibitor	No
CYP2D6 inhibitor	No
CYP3A4 inhibitor	No
Log K_p (skin permeation)	-8.64 cm/s

Druglikeness	
Lipinski	Yes; 0 violation
Ghose	No; 1 violation: WLOGP<-0.4
Veber	Yes
Egan	Yes
Muegge	Yes
Bioavailability Score	0.55

Medicinal Chemistry	
PAINS	0 alert
Brenk	1 alert: hydantoin
Leadlikeness	Yes
Synthetic accessibility	2.96

12	6PT3	COc3cccc([C@H]2CN(C(=O)CCCOCc1ccccc1)C[C@@H]2[NH3+])c3

Molecule 7

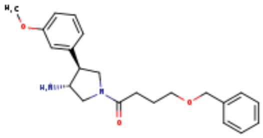

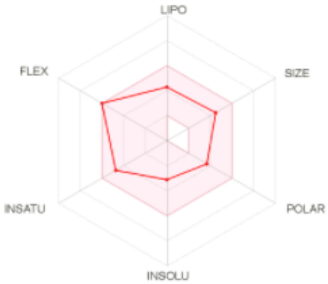

SMILES	COc1cccc(c1)[C@H]1CN(C[C@@H]1[NH3+])C(=O)CCCOCc1ccccc1

Physicochemical Properties	
Formula	C22H29N2O3
Molecular weight	369.48 g/mol
Num. heavy atoms	27
Num. arom. heavy atoms	12
Fraction Csp3	0.41
Num. rotatable bonds	9
Num. H-bond acceptors	3
Num. H-bond donors	1
Molar Refractivity	110.79
TPSA	66.41 Å²

Lipophilicity	
Log $P_{o/w}$ (iLOGP)	3.36
Log $P_{o/w}$ (XLOGP3)	1.98
Log $P_{o/w}$ (WLOGP)	1.70
Log $P_{o/w}$ (MLOGP)	-1.74
Log $P_{o/w}$ (SILICOS-IT)	3.30
Consensus Log $P_{o/w}$	1.72

Water Solubility	
Log *S* (ESOL)	-3.11
Solubility	2.85e-01 mg/ml ; 7.71e-04 mol/l
Class	Soluble
Log *S* (Ali)	-3.00
Solubility	3.69e-01 mg/ml ; 9.99e-04 mol/l
Class	Soluble
Log *S* (SILICOS-IT)	-5.92
Solubility	4.41e-04 mg/ml ; 1.19e-06 mol/l
Class	Moderately soluble

Pharmacokinetics	
GI absorption	High
BBB permeant	Yes
P-gp substrate	Yes
CYP1A2 inhibitor	No
CYP2C19 inhibitor	No
CYP2C9 inhibitor	No
CYP2D6 inhibitor	No
CYP3A4 inhibitor	No
Log K_p (skin permeation)	-7.15 cm/s

Druglikeness	
Lipinski	Yes; 0 violation
Ghose	Yes
Veber	Yes
Egan	Yes
Muegge	Yes
Bioavailability Score	0.55

Medicinal Chemistry	
PAINS	0 alert
Brenk	0 alert
Leadlikeness	No; 2 violations: MW>350, Rotors>7
Synthetic accessibility	3.59

13	6PT3	O=C2N(Cc1cccc(F)c1F)CCC[C@]2(O)C[NH2+]C(CO)CO

Molecule 1

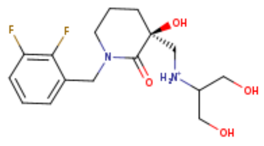

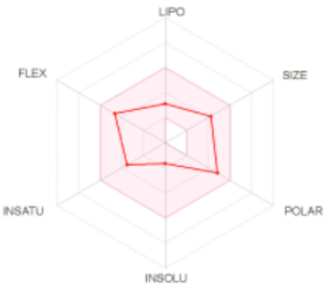

SMILES OCC([NH2+]C[C@@]1(O)CCCN(C1=O)Cc1cccc(c1F)F)CO

Physicochemical Properties	
Formula	C16H23F2N2O4
Molecular weight	345.36 g/mol
Num. heavy atoms	24
Num. arom. heavy atoms	6
Fraction Csp3	0.56
Num. rotatable bonds	7
Num. H-bond acceptors	6
Num. H-bond donors	4
Molar Refractivity	87.07
TPSA	97.61 Å²

Lipophilicity	
Log $P_{o/w}$ (iLOGP)	2.65
Log $P_{o/w}$ (XLOGP3)	-0.07
Log $P_{o/w}$ (WLOGP)	-0.96
Log $P_{o/w}$ (MLOGP)	-3.18
Log $P_{o/w}$ (SILICOS-IT)	1.92
Consensus Log $P_{o/w}$	0.07

Water Solubility	
Log *S* (ESOL)	-1.66
Solubility	7.55e+00 mg/ml ; 2.19e-02 mol/l
Class	Very soluble
Log *S* (Ali)	-1.53
Solubility	1.02e+01 mg/ml ; 2.96e-02 mol/l
Class	Very soluble
Log *S* (SILICOS-IT)	-3.52
Solubility	1.05e-01 mg/ml ; 3.04e-04 mol/l
Class	Soluble

Pharmacokinetics	
GI absorption	High
BBB permeant	No
P-gp substrate	Yes
CYP1A2 inhibitor	No
CYP2C19 inhibitor	No
CYP2C9 inhibitor	No
CYP2D6 inhibitor	No
CYP3A4 inhibitor	No
Log K_p (skin permeation)	-8.46 cm/s

Druglikeness	
Lipinski	Yes; 0 violation
Ghose	No; 1 violation: WLOGP<-0.4
Veber	Yes
Egan	Yes
Muegge	Yes
Bioavailability Score	0.55

Medicinal Chemistry	
PAINS	0 alert
Brenk	0 alert
Leadlikeness	Yes
Synthetic accessibility	2.92

14	6PT3	Cc2cc1c[nH]nc1cc2[C@H]3CC[C@@H](C[NH3+])[NH2+]C3

Molecule 2

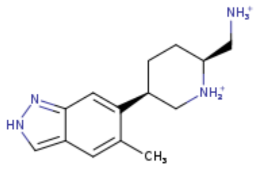

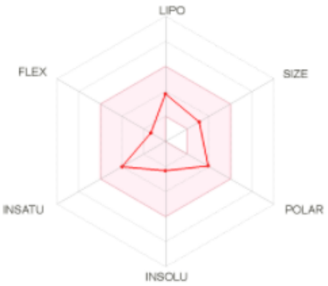

SMILES [NH3+]C[C@@H]1CC[C@@H](C[NH2+]1)c1cc2n[nH]cc2cc1C

Physicochemical Properties	
Formula	C14H22N4
Molecular weight	246.35 g/mol
Num. heavy atoms	18
Num. arom. heavy atoms	9
Fraction Csp3	0.50
Num. rotatable bonds	2
Num. H-bond acceptors	1
Num. H-bond donors	3
Molar Refractivity	79.59
TPSA	72.93 Å²

Lipophilicity	
Log $P_{o/w}$ (iLOGP)	1.68
Log $P_{o/w}$ (XLOGP3)	1.15
Log $P_{o/w}$ (WLOGP)	-0.46
Log $P_{o/w}$ (MLOGP)	-5.92
Log $P_{o/w}$ (SILICOS-IT)	2.40
Consensus Log $P_{o/w}$	-0.23

Water Solubility	
Log *S* (ESOL)	-2.33
Solubility	1.15e+00 mg/ml ; 4.68e-03 mol/l
Class	Soluble
Log *S* (Ali)	-2.28
Solubility	1.30e+00 mg/ml ; 5.30e-03 mol/l
Class	Soluble
Log *S* (SILICOS-IT)	-4.33
Solubility	1.15e-02 mg/ml ; 4.69e-05 mol/l
Class	Moderately soluble

Pharmacokinetics	
GI absorption	High
BBB permeant	No
P-gp substrate	Yes
CYP1A2 inhibitor	No
CYP2C19 inhibitor	No
CYP2C9 inhibitor	No
CYP2D6 inhibitor	No
CYP3A4 inhibitor	No
Log K_p (skin permeation)	-6.99 cm/s

Druglikeness	
Lipinski	Yes; 0 violation
Ghose	No; 1 violation: WLOGP<-0.4
Veber	Yes
Egan	Yes
Muegge	Yes
Bioavailability Score	0.55

Medicinal Chemistry	
PAINS	0 alert
Brenk	0 alert
Leadlikeness	No; 1 violation: MW<250
Synthetic accessibility	2.91

15	6PT3	Cc1ccccc1O[C@@H]2CCCC[C@@H]2[NH2+][C@@H]3CCCC[C@@H]3[NH3+]

Molecule 3

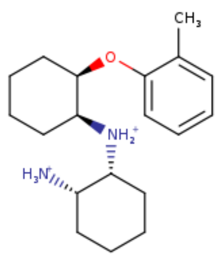

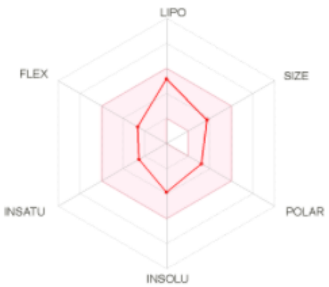

SMILES Cc1ccccc1O[C@@H]1CCCC[C@@H]1[NH2+][C@@H]1CCCC[C@@H]1[NH3+]

Physicochemical Properties	
Formula	C19H32N2O
Molecular weight	304.47 g/mol
Num. heavy atoms	22
Num. arom. heavy atoms	6
Fraction Csp3	0.68
Num. rotatable bonds	4
Num. H-bond acceptors	1
Num. H-bond donors	2
Molar Refractivity	94.57
TPSA	53.48 Å²

Lipophilicity	
Log $P_{o/w}$ (iLOGP)	3.56
Log $P_{o/w}$ (XLOGP3)	3.56
Log $P_{o/w}$ (WLOGP)	1.80
Log $P_{o/w}$ (MLOGP)	-4.48
Log $P_{o/w}$ (SILICOS-IT)	2.97
Consensus Log $P_{o/w}$	1.48

Water Solubility	
Log *S* (ESOL)	-3.91
Solubility	3.76e-02 mg/ml ; 1.23e-04 mol/l
Class	Soluble
Log *S* (Ali)	-4.37
Solubility	1.30e-02 mg/ml ; 4.28e-05 mol/l
Class	Moderately soluble
Log *S* (SILICOS-IT)	-4.62
Solubility	7.34e-03 mg/ml ; 2.41e-05 mol/l
Class	Moderately soluble

Pharmacokinetics	
GI absorption	High
BBB permeant	Yes
P-gp substrate	No
CYP1A2 inhibitor	No
CYP2C19 inhibitor	No
CYP2C9 inhibitor	No
CYP2D6 inhibitor	No
CYP3A4 inhibitor	No
Log K_p (skin permeation)	-5.63 cm/s

Druglikeness	
Lipinski	Yes; 0 violation
Ghose	Yes
Veber	Yes
Egan	Yes
Muegge	Yes
Bioavailability Score	0.55

Medicinal Chemistry	
PAINS	0 alert
Brenk	0 alert
Leadlikeness	No; 1 violation: XLOGP3>3.5
Synthetic accessibility	3.73

16	6PT3	O=c1[nH]c2ccc(NC(=S)NCc3ccccc3)cc2[nH]1

Molecule 4

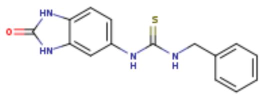

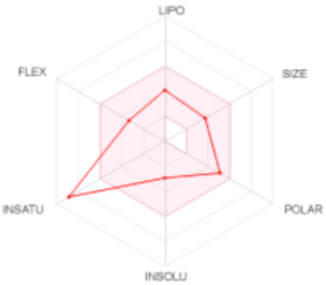

SMILES S=C(Nc1ccc2c(c1)[nH]c(=O)[nH]2)NCc1ccccc1

Physicochemical Properties	
Formula	C15H14N4OS
Molecular weight	298.36 g/mol
Num. heavy atoms	21
Num. arom. heavy atoms	15
Fraction Csp3	0.07
Num. rotatable bonds	5
Num. H-bond acceptors	1
Num. H-bond donors	4
Molar Refractivity	88.11
TPSA	104.80 Å²

Lipophilicity	
Log $P_{o/w}$ (iLOGP)	2.06
Log $P_{o/w}$ (XLOGP3)	1.66
Log $P_{o/w}$ (WLOGP)	2.00
Log $P_{o/w}$ (MLOGP)	1.84
Log $P_{o/w}$ (SILICOS-IT)	3.78
Consensus Log $P_{o/w}$	2.27

Water Solubility	
Log *S* (ESOL)	-2.93
Solubility	3.47e-01 mg/ml ; 1.16e-03 mol/l
Class	Soluble
Log *S* (Ali)	-3.47
Solubility	1.00e-01 mg/ml ; 3.35e-04 mol/l
Class	Soluble
Log *S* (SILICOS-IT)	-5.99
Solubility	3.09e-04 mg/ml ; 1.04e-06 mol/l
Class	Moderately soluble

Pharmacokinetics	
GI absorption	High
BBB permeant	No
P-gp substrate	No
CYP1A2 inhibitor	Yes
CYP2C19 inhibitor	Yes
CYP2C9 inhibitor	No
CYP2D6 inhibitor	No
CYP3A4 inhibitor	Yes
Log K_p (skin permeation)	-6.94 cm/s

Druglikeness	
Lipinski	Yes; 0 violation
Ghose	Yes
Veber	Yes
Egan	Yes
Muegge	Yes
Bioavailability Score	0.55

Medicinal Chemistry	
PAINS	0 alert
Brenk	1 alert: thiocarbonyl_group
Leadlikeness	Yes
Synthetic accessibility	2.30

17	6PT3	OC3(C[NH2+]C2CC(c1ccccc1F)C2)CCCCC3

Molecule 5

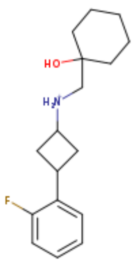

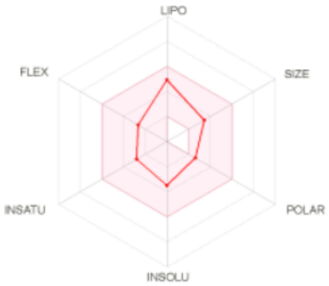

SMILES Fc1ccccc1C1CC(C1)[NH2+]CC1(O)CCCCC1

Physicochemical Properties	
Formula	C17H25FNO
Molecular weight	278.38 g/mol
Num. heavy atoms	20
Num. arom. heavy atoms	6
Fraction Csp3	0.65
Num. rotatable bonds	4
Num. H-bond acceptors	2
Num. H-bond donors	2
Molar Refractivity	80.47
TPSA	36.84 Å²
**Lipophilicity**	
Log $P_{o/w}$ (iLOGP)	3.30
Log $P_{o/w}$ (XLOGP3)	3.14
Log $P_{o/w}$ (WLOGP)	2.75
Log $P_{o/w}$ (MLOGP)	-0.27
Log $P_{o/w}$ (SILICOS-IT)	3.88
Consensus Log $P_{o/w}$	2.56

Water Solubility	
Log *S* (ESOL)	-3.50
Solubility	8.76e-02 mg/ml ; 3.15e-04 mol/l
Class	Soluble
Log *S* (Ali)	-3.58
Solubility	7.27e-02 mg/ml ; 2.61e-04 mol/l
Class	Soluble
Log *S* (SILICOS-IT)	-4.86
Solubility	3.84e-03 mg/ml ; 1.38e-05 mol/l
Class	Moderately soluble
**Pharmacokinetics**	
GI absorption	High
BBB permeant	Yes
P-gp substrate	No
CYP1A2 inhibitor	No
CYP2C19 inhibitor	No
CYP2C9 inhibitor	No
CYP2D6 inhibitor	Yes
CYP3A4 inhibitor	No
Log K_p (skin permeation)	-5.77 cm/s
**Druglikeness**	
Lipinski	Yes; 0 violation
Ghose	Yes
Veber	Yes
Egan	Yes
Muegge	Yes
Bioavailability Score	0.55
**Medicinal Chemistry**	
PAINS	0 alert
Brenk	0 alert
Leadlikeness	Yes
Synthetic accessibility	3.07

18	6PT3	O=C(NC[C@@H]1CCCO1)[C@H]5C[C@@H](C(=O)Nc3ccc2CCCc2c3)C[NH+](Cc4ccc(O)cc4)C5

Molecule 6

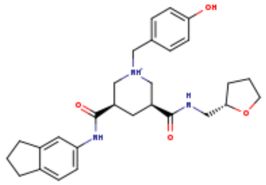

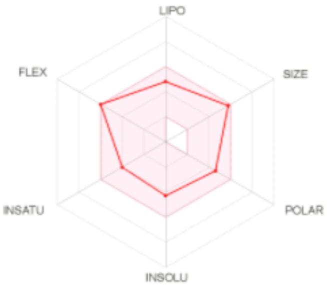

SMILES Oc1ccc(cc1)C[NH+]1C[C@@H](C[C@@H](C1)C(=O)NC[C@@H]1CCCO1)C(=O)Nc1ccc2c(c1)CCC2

Physicochemical Properties	
Formula	C28H36N3O4
Molecular weight	478.60 g/mol
Num. heavy atoms	35
Num. arom. heavy atoms	12
Fraction Csp3	0.50
Num. rotatable bonds	9
Num. H-bond acceptors	4
Num. H-bond donors	4
Molar Refractivity	140.40
TPSA	92.10 Å²

Lipophilicity	
Log $P_{o/w}$ (iLOGP)	3.86
Log $P_{o/w}$ (XLOGP3)	2.90
Log $P_{o/w}$ (WLOGP)	1.11
Log $P_{o/w}$ (MLOGP)	-1.52
Log $P_{o/w}$ (SILICOS-IT)	3.71
Consensus Log $P_{o/w}$	2.01

Water Solubility	
Log *S* (ESOL)	-4.29
Solubility	2.43e-02 mg/ml ; 5.08e-05 mol/l
Class	Moderately soluble
Log *S* (Ali)	-4.49
Solubility	1.53e-02 mg/ml ; 3.20e-05 mol/l
Class	Moderately soluble
Log *S* (SILICOS-IT)	-6.71
Solubility	9.38e-05 mg/ml ; 1.96e-07 mol/l
Class	Poorly soluble

Pharmacokinetics	
GI absorption	High
BBB permeant	No
P-gp substrate	Yes
CYP1A2 inhibitor	No
CYP2C19 inhibitor	No
CYP2C9 inhibitor	No
CYP2D6 inhibitor	No
CYP3A4 inhibitor	No
Log K_p (skin permeation)	-7.16 cm/s

Druglikeness	
Lipinski	Yes; 0 violation
Ghose	No; 2 violations: MR>130, #atoms>70
Veber	Yes
Egan	Yes
Muegge	Yes
Bioavailability Score	0.55

Medicinal Chemistry	
PAINS	0 alert
Brenk	0 alert
Leadlikeness	No; 2 violations: MW>350, Rotors>7
Synthetic accessibility	4.33

19	6PT3	[NH3+][C@@H]1CCC[C@@H]1[NH2+]Cc2ccc(F)cc2

Molecule 7

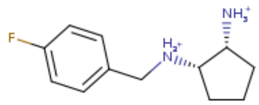

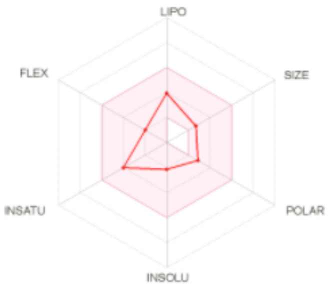

SMILES Fc1ccc(cc1)C[NH2+][C@H]1CCC[C@H]1[NH3+]

Physicochemical Properties	
Formula	C12H19FN2
Molecular weight	210.29 g/mol
Num. heavy atoms	15
Num. arom. heavy atoms	6
Fraction Csp3	0.50
Num. rotatable bonds	3
Num. H-bond acceptors	1
Num. H-bond donors	2
Molar Refractivity	61.31
TPSA	44.25 Å²

Lipophilicity	
Log $P_{o/w}$ (iLOGP)	2.46
Log $P_{o/w}$ (XLOGP3)	1.43
Log $P_{o/w}$ (WLOGP)	0.32
Log $P_{o/w}$ (MLOGP)	-5.22
Log $P_{o/w}$ (SILICOS-IT)	2.19
Consensus Log $P_{o/w}$	0.24

Water Solubility	
Log *S* (ESOL)	-2.14
Solubility	1.51e+00 mg/ml ; 7.20e-03 mol/l
Class	Soluble
Log *S* (Ali)	-1.96
Solubility	2.28e+00 mg/ml ; 1.09e-02 mol/l
Class	Very soluble
Log *S* (SILICOS-IT)	-3.71
Solubility	4.13e-02 mg/ml ; 1.96e-04 mol/l
Class	Soluble

Pharmacokinetics	
GI absorption	High
BBB permeant	No
P-gp substrate	No
CYP1A2 inhibitor	Yes
CYP2C19 inhibitor	No
CYP2C9 inhibitor	No
CYP2D6 inhibitor	No
CYP3A4 inhibitor	No
Log K_p (skin permeation)	-6.57 cm/s

Druglikeness	
Lipinski	Yes; 0 violation
Ghose	Yes
Veber	Yes
Egan	Yes
Muegge	Yes
Bioavailability Score	0.55

Medicinal Chemistry	
PAINS	0 alert
Brenk	0 alert
Leadlikeness	No; 1 violation: MW<250
Synthetic accessibility	2.22

20	6PT3	[NH3+]C(CSCc1ccccc1)CSCc2ccccc2

Molecule 8

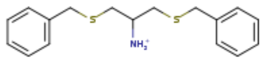

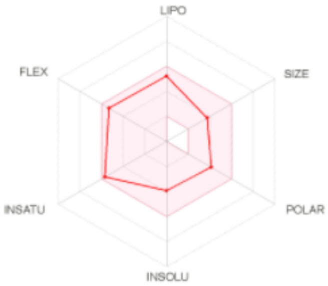

SMILES [NH3+]C(CSCc1ccccc1)CSCc1ccccc1

Physicochemical Properties	
Formula	C17H22NS2
Molecular weight	304.49 g/mol
Num. heavy atoms	20
Num. arom. heavy atoms	12
Fraction Csp3	0.29
Num. rotatable bonds	8
Num. H-bond acceptors	0
Num. H-bond donors	1
Molar Refractivity	94.27
TPSA	78.24 Å²

Lipophilicity	
Log $P_{o/w}$ (iLOGP)	2.96
Log $P_{o/w}$ (XLOGP3)	3.64
Log $P_{o/w}$ (WLOGP)	3.16
Log $P_{o/w}$ (MLOGP)	0.41
Log $P_{o/w}$ (SILICOS-IT)	4.49
Consensus Log $P_{o/w}$	2.93

Water Solubility	
Log *S* (ESOL)	-3.94
Solubility	3.52e-02 mg/ml ; 1.16e-04 mol/l
Class	Soluble
Log *S* (Ali)	-4.97
Solubility	3.25e-03 mg/ml ; 1.07e-05 mol/l
Class	Moderately soluble
Log *S* (SILICOS-IT)	-6.43
Solubility	1.13e-04 mg/ml ; 3.71e-07 mol/l
Class	Poorly soluble

Pharmacokinetics	
GI absorption	High
BBB permeant	Yes
P-gp substrate	No
CYP1A2 inhibitor	Yes
CYP2C19 inhibitor	Yes
CYP2C9 inhibitor	Yes
CYP2D6 inhibitor	Yes
CYP3A4 inhibitor	No
Log K_p (skin permeation)	-5.57 cm/s

Druglikeness	
Lipinski	Yes; 0 violation
Ghose	Yes
Veber	Yes
Egan	Yes
Muegge	Yes
Bioavailability Score	0.55

Medicinal Chemistry	
PAINS	0 alert
Brenk	0 alert
Leadlikeness	No; 2 violations: Rotors>7, XLOGP3>3.5
Synthetic accessibility	3.20

21	6PT3	COc1ccccc1OC[C@H](O)C[NH+](CCc2ccccc2)Cc3ccccc3

Molecule 1

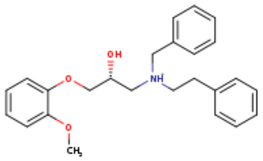

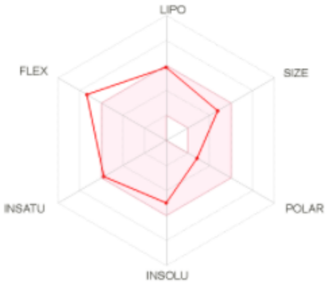

SMILES COc1ccccc1OC[C@@H](C[NH+](Cc1ccccc1)CCc1ccccc1)O

Physicochemical Properties	
Formula	C25H30NO3
Molecular weight	392.51 g/mol
Num. heavy atoms	29
Num. arom. heavy atoms	18
Fraction Csp3	0.28
Num. rotatable bonds	11
Num. H-bond acceptors	3
Num. H-bond donors	2
Molar Refractivity	117.75
TPSA	43.13 Å²

Lipophilicity	
Log $P_{o/w}$ (iLOGP)	4.09
Log $P_{o/w}$ (XLOGP3)	4.73
Log $P_{o/w}$ (WLOGP)	2.61
Log $P_{o/w}$ (MLOGP)	-0.18
Log $P_{o/w}$ (SILICOS-IT)	4.84
Consensus Log $P_{o/w}$	3.22

Water Solubility	
Log *S* (ESOL)	-4.99
Solubility	4.05e-03 mg/ml ; 1.03e-05 mol/l
Class	Moderately soluble
Log *S* (Ali)	-5.37
Solubility	1.69e-03 mg/ml ; 4.31e-06 mol/l
Class	Moderately soluble
Log *S* (SILICOS-IT)	-8.06
Solubility	3.40e-06 mg/ml ; 8.67e-09 mol/l
Class	Poorly soluble

Pharmacokinetics	
GI absorption	High
BBB permeant	Yes
P-gp substrate	Yes
CYP1A2 inhibitor	No
CYP2C19 inhibitor	No
CYP2C9 inhibitor	No
CYP2D6 inhibitor	No
CYP3A4 inhibitor	No
Log K_p (skin permeation)	-5.34 cm/s

Druglikeness	
Lipinski	Yes; 0 violation
Ghose	Yes
Veber	No; 1 violation: Rotors>10
Egan	Yes
Muegge	Yes
Bioavailability Score	0.55

Medicinal Chemistry	
PAINS	0 alert
Brenk	0 alert
Leadlikeness	No; 3 violations: MW>350, Rotors>7, XLOGP3>3.5
Synthetic accessibility	3.27

22	6PT3	COc1ccccc1C[NH2+][C@H]2CCc4c(C2)c(C(=O)N3CCOCC3)nn4CC(C)C

Molecule 2

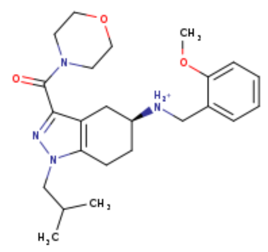

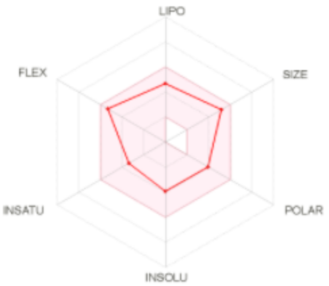

SMILES COc1ccccc1C[NH2+][C@H]1CCc2c(C1)c(nn2CC(C)C)C(=O)N1CCOCC1

Physicochemical Properties	
Formula	C24H35N4O3
Molecular weight	427.56 g/mol
Num. heavy atoms	31
Num. arom. heavy atoms	11
Fraction Csp3	0.58
Num. rotatable bonds	8
Num. H-bond acceptors	4
Num. H-bond donors	1
Molar Refractivity	125.58
TPSA	73.20 Å²

Lipophilicity	
Log $P_{o/w}$ (iLOGP)	3.76
Log $P_{o/w}$ (XLOGP3)	2.71
Log $P_{o/w}$ (WLOGP)	1.11
Log $P_{o/w}$ (MLOGP)	-1.81
Log $P_{o/w}$ (SILICOS-IT)	3.35
Consensus Log $P_{o/w}$	1.82

Water Solubility	
Log *S* (ESOL)	-3.93
Solubility	4.99e-02 mg/ml ; 1.17e-04 mol/l
Class	Soluble
Log *S* (Ali)	-3.90
Solubility	5.38e-02 mg/ml ; 1.26e-04 mol/l
Class	Soluble
Log *S* (SILICOS-IT)	-5.65
Solubility	9.57e-04 mg/ml ; 2.24e-06 mol/l
Class	Moderately soluble

Pharmacokinetics	
GI absorption	High
BBB permeant	No
P-gp substrate	Yes
CYP1A2 inhibitor	No
CYP2C19 inhibitor	No
CYP2C9 inhibitor	No
CYP2D6 inhibitor	No
CYP3A4 inhibitor	No
Log K_p (skin permeation)	-6.98 cm/s

Druglikeness	
Lipinski	Yes; 0 violation
Ghose	Yes
Veber	Yes
Egan	Yes
Muegge	Yes
Bioavailability Score	0.55

Medicinal Chemistry	
PAINS	0 alert
Brenk	0 alert
Leadlikeness	No; 2 violations: MW>350, Rotors>7
Synthetic accessibility	4.32

23	6PT3	C[C@H]([NH2+][C@@H]1CCCC[C@@H]1[NH3+])c2ccccc2

Molecule 3

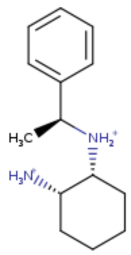

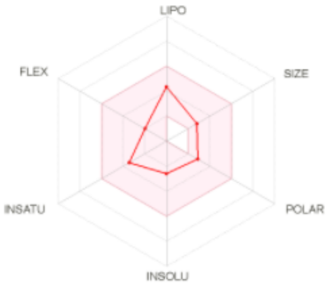

SMILES [NH3+][C@H]1CCCC[C@H]1[NH2+][C@H](c1ccccc1)C

Physicochemical Properties	
Formula	C14H24N2
Molecular weight	220.35 g/mol
Num. heavy atoms	16
Num. arom. heavy atoms	6
Fraction Csp3	0.57
Num. rotatable bonds	3
Num. H-bond acceptors	0
Num. H-bond donors	2
Molar Refractivity	70.97
TPSA	44.25 Å²

Lipophilicity	
Log $P_{o/w}$ (iLOGP)	2.77
Log $P_{o/w}$ (XLOGP3)	2.09
Log $P_{o/w}$ (WLOGP)	0.54
Log $P_{o/w}$ (MLOGP)	-5.09
Log $P_{o/w}$ (SILICOS-IT)	2.22
Consensus Log $P_{o/w}$	0.51

Water Solubility	
Log *S* (ESOL)	-2.60
Solubility	5.50e-01 mg/ml ; 2.50e-03 mol/l
Class	Soluble
Log *S* (Ali)	-2.65
Solubility	4.94e-01 mg/ml ; 2.24e-03 mol/l
Class	Soluble
Log *S* (SILICOS-IT)	-3.74
Solubility	4.02e-02 mg/ml ; 1.82e-04 mol/l
Class	Soluble

Pharmacokinetics	
GI absorption	High
BBB permeant	Yes
P-gp substrate	No
CYP1A2 inhibitor	Yes
CYP2C19 inhibitor	No
CYP2C9 inhibitor	No
CYP2D6 inhibitor	No
CYP3A4 inhibitor	No
Log K_p (skin permeation)	-6.16 cm/s

Druglikeness	
Lipinski	Yes; 0 violation
Ghose	Yes
Veber	Yes
Egan	Yes
Muegge	Yes
Bioavailability Score	0.55

Medicinal Chemistry	
PAINS	0 alert
Brenk	0 alert
Leadlikeness	No; 1 violation: MW<250
Synthetic accessibility	2.42

24	6PT3	CC2(C)S[C@@H](CNC(=O)[C@H]([NH3+])c1ccccc1)N[C@H]2C(=O)[O-]

Molecule 4

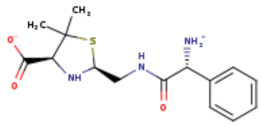

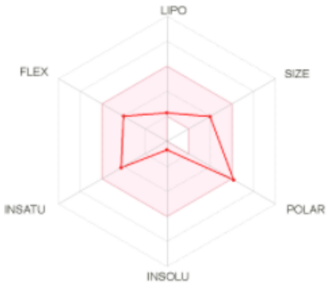

SMILES [NH3+][C@H](c1ccccc1)C(=O)NC[C@H]1N[C@H](C(S1)(C)C)C(=O)[O-]

Physicochemical Properties	
Formula	C15H21N3O3S
Molecular weight	323.41 g/mol
Num. heavy atoms	22
Num. arom. heavy atoms	6
Fraction Csp3	0.47
Num. rotatable bonds	6
Num. H-bond acceptors	4
Num. H-bond donors	3
Molar Refractivity	88.89
TPSA	134.20 Å²

Lipophilicity	
Log $P_{o/w}$ (iLOGP)	1.58
Log $P_{o/w}$ (XLOGP3)	-1.55
Log $P_{o/w}$ (WLOGP)	-2.06
Log $P_{o/w}$ (MLOGP)	-5.69
Log $P_{o/w}$ (SILICOS-IT)	0.76
Consensus Log $P_{o/w}$	-1.39

Water Solubility	
Log *S* (ESOL)	-0.67
Solubility	6.84e+01 mg/ml ; 2.12e-01 mol/l
Class	Very soluble
Log *S* (Ali)	-0.76
Solubility	5.61e+01 mg/ml ; 1.73e-01 mol/l
Class	Very soluble
Log *S* (SILICOS-IT)	-3.14
Solubility	2.35e-01 mg/ml ; 7.28e-04 mol/l
Class	Soluble

Pharmacokinetics	
GI absorption	Low
BBB permeant	No
P-gp substrate	No
CYP1A2 inhibitor	No
CYP2C19 inhibitor	Yes
CYP2C9 inhibitor	No
CYP2D6 inhibitor	No
CYP3A4 inhibitor	No
Log K_p (skin permeation)	-9.37 cm/s

Druglikeness	
Lipinski	Yes; 0 violation
Ghose	No; 1 violation: WLOGP<-0.4
Veber	Yes
Egan	No; 1 violation: TPSA>131.6
Muegge	Yes
Bioavailability Score	0.55

Medicinal Chemistry	
PAINS	0 alert
Brenk	0 alert
Leadlikeness	Yes
Synthetic accessibility	3.85

25	6PT3	O=C2CC[C@@H](CC[NH2+]Cc1cccc(C(F)(F)F)c1)N2[C@H]4CC[NH+](Cc3ccccc3)C4

Molecule 5

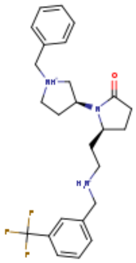

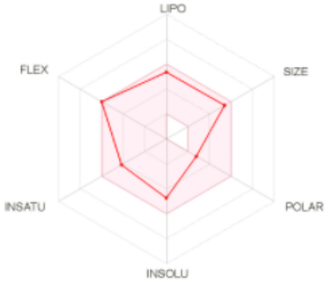

SMILES O=C1CC[C@H](N1[C@H]1CC[NH+](C1)Cc1ccccc1)CC[NH2+]Cc1cccc(c1)C(F)(F)F

Physicochemical Properties	
Formula	C25H32F3N3O
Molecular weight	447.54 g/mol
Num. heavy atoms	32
Num. arom. heavy atoms	12
Fraction Csp3	0.48
Num. rotatable bonds	9
Num. H-bond acceptors	4
Num. H-bond donors	2
Molar Refractivity	128.39
TPSA	41.36 Å²

Lipophilicity	
Log $P_{o/w}$ (iLOGP)	3.94
Log $P_{o/w}$ (XLOGP3)	3.89
Log $P_{o/w}$ (WLOGP)	2.09
Log $P_{o/w}$ (MLOGP)	-3.66
Log $P_{o/w}$ (SILICOS-IT)	4.80
Consensus Log $P_{o/w}$	2.22

Water Solubility	
Log *S* (ESOL)	-4.75
Solubility	7.98e-03 mg/ml ; 1.78e-05 mol/l
Class	Moderately soluble
Log *S* (Ali)	-4.46
Solubility	1.56e-02 mg/ml ; 3.50e-05 mol/l
Class	Moderately soluble
Log *S* (SILICOS-IT)	-7.67
Solubility	9.59e-06 mg/ml ; 2.14e-08 mol/l
Class	Poorly soluble

Pharmacokinetics	
GI absorption	High
BBB permeant	Yes
P-gp substrate	Yes
CYP1A2 inhibitor	No
CYP2C19 inhibitor	No
CYP2C9 inhibitor	No
CYP2D6 inhibitor	No
CYP3A4 inhibitor	No
Log K_p (skin permeation)	-6.27 cm/s

Druglikeness	
Lipinski	Yes; 0 violation
Ghose	Yes
Veber	Yes
Egan	Yes
Muegge	Yes
Bioavailability Score	0.55

Medicinal Chemistry	
PAINS	0 alert
Brenk	0 alert
Leadlikeness	No; 3 violations: MW>350, Rotors>7, XLOGP3>3.5
Synthetic accessibility	3.73

26	6PT3	O=C4C[C@@H]([NH+]3CCC(C2CCN(C(=O)c1ccc(F)cc1)CC2)CC3)C(=O)N4

Molecule 6

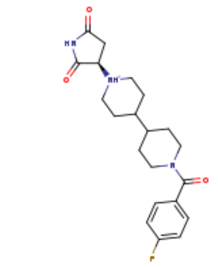

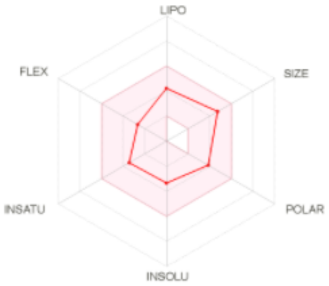

SMILES	O=C(c1ccc(cc1)F)N1CCC(CC1)C1CC[NH+](CC1)[C@@H]1CC(=O)NC1=O

Physicochemical Properties	
Formula	C21H27FN3O3
Molecular weight	388.46 g/mol
Num. heavy atoms	28
Num. arom. heavy atoms	6
Fraction Csp3	0.57
Num. rotatable bonds	4
Num. H-bond acceptors	4
Num. H-bond donors	2
Molar Refractivity	114.44
TPSA	70.92 Å²

Lipophilicity	
Log $P_{o/w}$ (iLOGP)	2.52
Log $P_{o/w}$ (XLOGP3)	1.88
Log $P_{o/w}$ (WLOGP)	-0.33
Log $P_{o/w}$ (MLOGP)	-1.10
Log $P_{o/w}$ (SILICOS-IT)	2.38
Consensus Log $P_{o/w}$	1.07

Water Solubility	
Log *S* (ESOL)	-3.33
Solubility	1.83e-01 mg/ml ; 4.71e-04 mol/l
Class	Soluble
Log *S* (Ali)	-2.99
Solubility	3.96e-01 mg/ml ; 1.02e-03 mol/l
Class	Soluble
Log *S* (SILICOS-IT)	-4.25
Solubility	2.17e-02 mg/ml ; 5.58e-05 mol/l
Class	Moderately soluble

Pharmacokinetics	
GI absorption	High
BBB permeant	No
P-gp substrate	Yes
CYP1A2 inhibitor	No
CYP2C19 inhibitor	No
CYP2C9 inhibitor	No
CYP2D6 inhibitor	No
CYP3A4 inhibitor	No
Log K_p (skin permeation)	-7.33 cm/s

Druglikeness	
Lipinski	Yes; 0 violation
Ghose	Yes
Veber	Yes
Egan	Yes
Muegge	Yes
Bioavailability Score	0.55

Medicinal Chemistry	
PAINS	0 alert
Brenk	1 alert: phthalimide
Leadlikeness	No; 1 violation: MW>350
Synthetic accessibility	3.66

27	6PT3	Cc1cccc(OC[C@@H](O)CC(N)=[NH2+])c1

Molecule 7

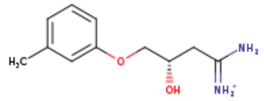

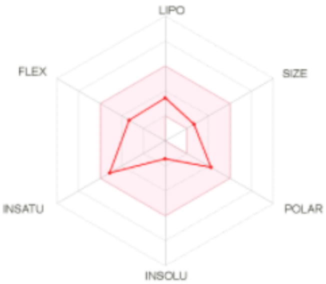

SMILES O[C@@H](CC(=[NH2+])N)COc1cccc(c1)C

Physicochemical Properties	
Formula	C11H17N2O2
Molecular weight	209.26 g/mol
Num. heavy atoms	15
Num. arom. heavy atoms	6
Fraction Csp3	0.36
Num. rotatable bonds	5
Num. H-bond acceptors	2
Num. H-bond donors	3
Molar Refractivity	60.45
TPSA	81.07 Å²

Lipophilicity	
Log $P_{o/w}$ (iLOGP)	1.70
Log $P_{o/w}$ (XLOGP3)	0.50
Log $P_{o/w}$ (WLOGP)	-0.76
Log $P_{o/w}$ (MLOGP)	1.11
Log $P_{o/w}$ (SILICOS-IT)	1.41
Consensus Log $P_{o/w}$	0.79

Water Solubility	
Log *S* (ESOL)	-1.42
Solubility	7.98e+00 mg/ml ; 3.82e-02 mol/l
Class	Very soluble
Log *S* (Ali)	-1.77
Solubility	3.53e+00 mg/ml ; 1.69e-02 mol/l
Class	Very soluble
Log *S* (SILICOS-IT)	-2.55
Solubility	5.91e-01 mg/ml ; 2.82e-03 mol/l
Class	Soluble

Pharmacokinetics	
GI absorption	High
BBB permeant	No
P-gp substrate	No
CYP1A2 inhibitor	No
CYP2C19 inhibitor	No
CYP2C9 inhibitor	No
CYP2D6 inhibitor	No
CYP3A4 inhibitor	No
Log K_p (skin permeation)	-7.22 cm/s

Druglikeness	
Lipinski	Yes; 0 violation
Ghose	No; 1 violation: WLOGP<-0.4
Veber	Yes
Egan	Yes
Muegge	Yes
Bioavailability Score	0.55

Medicinal Chemistry	
PAINS	0 alert
Brenk	2 alerts: imine_1, imine_2
Leadlikeness	No; 1 violation: MW<250
Synthetic accessibility	2.45

28	6PT3	CC(C)c3ccc(CN2CCC[C@](O)(C[NH2+]CCc1ccc(O)cc1)C2=O)cc3

Molecule 8

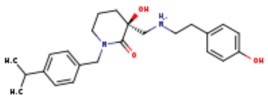

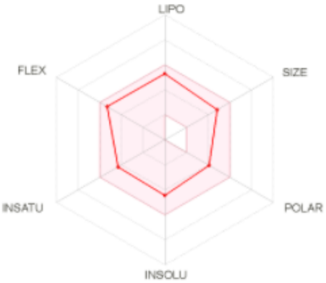

SMILES Oc1ccc(cc1)CC[NH2+]C[C@@]1(O)CCCN(C1=O)Cc1ccc(cc1)C(C)C

Physicochemical Properties	
Formula	C24H33N2O3
Molecular weight	397.53 g/mol
Num. heavy atoms	29
Num. arom. heavy atoms	12
Fraction Csp3	0.46
Num. rotatable bonds	8
Num. H-bond acceptors	3
Num. H-bond donors	3
Molar Refractivity	121.11
TPSA	77.38 Å²

Lipophilicity	
Log $P_{o/w}$ (iLOGP)	3.11
Log $P_{o/w}$ (XLOGP3)	3.76
Log $P_{o/w}$ (WLOGP)	1.64
Log $P_{o/w}$ (MLOGP)	-1.32
Log $P_{o/w}$ (SILICOS-IT)	4.22
Consensus Log $P_{o/w}$	2.28

Water Solubility	
Log *S* (ESOL)	-4.45
Solubility	1.40e-02 mg/ml ; 3.53e-05 mol/l
Class	Moderately soluble
Log *S* (Ali)	-5.08
Solubility	3.32e-03 mg/ml ; 8.36e-06 mol/l
Class	Moderately soluble
Log *S* (SILICOS-IT)	-6.79
Solubility	6.52e-05 mg/ml ; 1.64e-07 mol/l
Class	Poorly soluble

Pharmacokinetics	
GI absorption	High
BBB permeant	No
P-gp substrate	No
CYP1A2 inhibitor	No
CYP2C19 inhibitor	No
CYP2C9 inhibitor	No
CYP2D6 inhibitor	No
CYP3A4 inhibitor	No
Log K_p (skin permeation)	-6.06 cm/s

Druglikeness	
Lipinski	Yes; 0 violation
Ghose	Yes
Veber	Yes
Egan	Yes
Muegge	Yes
Bioavailability Score	0.55

Medicinal Chemistry	
PAINS	0 alert
Brenk	0 alert
Leadlikeness	No; 3 violations: MW>350, Rotors>7, XLOGP3>3.5
Synthetic accessibility	3.20

29	6PT3	O=C([O-])c1ccccc1N(CCC2=CCCCC2)S(=O)(=O)c3ccc4[nH]c(=O)c5cccc3c45

Molecule 1

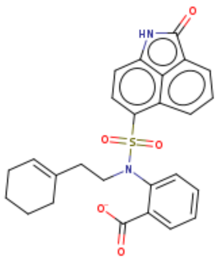

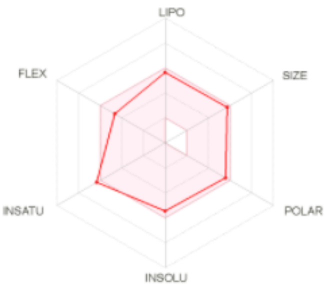

SMILES [O-]C(=O)c1ccccc1N(S(=O)(=O)c1ccc2c3c1cccc3c(=O)[nH]2)CCC1=CCCCC1

Physicochemical Properties	
Formula	C26H23N2O5S
Molecular weight	475.54 g/mol
Num. heavy atoms	34
Num. arom. heavy atoms	18
Fraction Csp3	0.23
Num. rotatable bonds	7
Num. H-bond acceptors	5
Num. H-bond donors	1
Molar Refractivity	131.35
TPSA	118.75 Å²

Lipophilicity	
Log $P_{o/w}$ (iLOGP)	2.77
Log $P_{o/w}$ (XLOGP3)	4.37
Log $P_{o/w}$ (WLOGP)	4.65
Log $P_{o/w}$ (MLOGP)	3.42
Log $P_{o/w}$ (SILICOS-IT)	4.16
Consensus Log $P_{o/w}$	3.87

Water Solubility	
Log *S* (ESOL)	-5.47
Solubility	1.61e-03 mg/ml ; 3.38e-06 mol/l
Class	Moderately soluble
Log *S* (Ali)	-6.58
Solubility	1.25e-04 mg/ml ; 2.63e-07 mol/l
Class	Poorly soluble
Log *S* (SILICOS-IT)	-7.67
Solubility	1.02e-05 mg/ml ; 2.14e-08 mol/l
Class	Poorly soluble

Pharmacokinetics	
GI absorption	High
BBB permeant	No
P-gp substrate	No
CYP1A2 inhibitor	Yes
CYP2C19 inhibitor	Yes
CYP2C9 inhibitor	Yes
CYP2D6 inhibitor	No
CYP3A4 inhibitor	Yes
Log K_p (skin permeation)	-6.10 cm/s

Druglikeness	
Lipinski	Yes; 0 violation
Ghose	No; 1 violation: MR>130
Veber	Yes
Egan	Yes
Muegge	Yes
Bioavailability Score	0.56

Medicinal Chemistry	
PAINS	0 alert
Brenk	1 alert: isolated_alkene
Leadlikeness	No; 2 violations: MW>350, XLOGP3>3.5
Synthetic accessibility	3.57

30	6PT3	O=c1ccc(-c2ccc3cc(OCc4ccccc4)ccc3c2)c[nH]1

Molecule 2

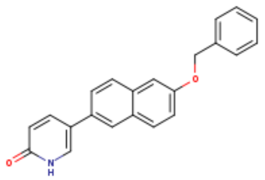

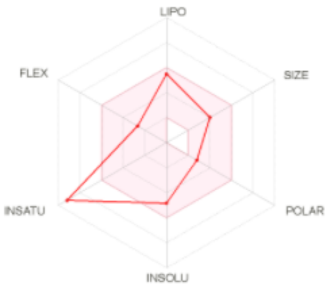

SMILES O=c1ccc(c[nH]1)c1ccc2c(c1)ccc(c2)OCc1ccccc1

Physicochemical Properties	
Formula	C22H17NO2
Molecular weight	327.38 g/mol
Num. heavy atoms	25
Num. arom. heavy atoms	22
Fraction Csp3	0.05
Num. rotatable bonds	4
Num. H-bond acceptors	2
Num. H-bond donors	1
Molar Refractivity	100.98
TPSA	42.09 Å²

Lipophilicity	
Log $P_{o/w}$ (iLOGP)	3.18
Log $P_{o/w}$ (XLOGP3)	4.10
Log $P_{o/w}$ (WLOGP)	4.62
Log $P_{o/w}$ (MLOGP)	3.62
Log $P_{o/w}$ (SILICOS-IT)	5.58
Consensus Log $P_{o/w}$	4.22

Water Solubility	
Log *S* (ESOL)	-4.84
Solubility	4.73e-03 mg/ml ; 1.45e-05 mol/l
Class	Moderately soluble
Log *S* (Ali)	-4.69
Solubility	6.69e-03 mg/ml ; 2.04e-05 mol/l
Class	Moderately soluble
Log *S* (SILICOS-IT)	-8.76
Solubility	5.68e-07 mg/ml ; 1.73e-09 mol/l
Class	Poorly soluble

Pharmacokinetics	
GI absorption	High
BBB permeant	Yes
P-gp substrate	Yes
CYP1A2 inhibitor	Yes
CYP2C19 inhibitor	Yes
CYP2C9 inhibitor	Yes
CYP2D6 inhibitor	Yes
CYP3A4 inhibitor	Yes
Log K_p (skin permeation)	-5.39 cm/s

Druglikeness	
Lipinski	Yes; 0 violation
Ghose	Yes
Veber	Yes
Egan	Yes
Muegge	Yes
Bioavailability Score	0.55

Medicinal Chemistry	
PAINS	0 alert
Brenk	0 alert
Leadlikeness	No; 1 violation: XLOGP3>3.5
Synthetic accessibility	2.58

31	6PT3	CC[C@H](OC[C@@H](O)C[NH2+][C@H](C)c1ccccc1)c2ccccc2

Molecule 3

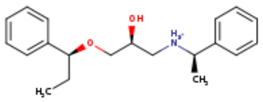

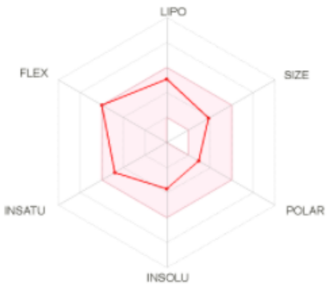

SMILES CC[C@@H](c1ccccc1)OC[C@H](C[NH2+][C@@H](c1ccccc1)C)O

Physicochemical Properties	
Formula	C20H28NO2
Molecular weight	314.44 g/mol
Num. heavy atoms	23
Num. arom. heavy atoms	12
Fraction Csp3	0.40
Num. rotatable bonds	9
Num. H-bond acceptors	2
Num. H-bond donors	2
Molar Refractivity	95.85
TPSA	46.07 Å²

Lipophilicity	
Log $P_{o/w}$ (iLOGP)	3.28
Log $P_{o/w}$ (XLOGP3)	3.35
Log $P_{o/w}$ (WLOGP)	2.19
Log $P_{o/w}$ (MLOGP)	-0.71
Log $P_{o/w}$ (SILICOS-IT)	4.13
Consensus Log $P_{o/w}$	2.45

Water Solubility	
Log *S* (ESOL)	-3.69
Solubility	6.39e-02 mg/ml ; 2.03e-04 mol/l
Class	Soluble
Log *S* (Ali)	-3.99
Solubility	3.18e-02 mg/ml ; 1.01e-04 mol/l
Class	Soluble
Log *S* (SILICOS-IT)	-6.25
Solubility	1.78e-04 mg/ml ; 5.65e-07 mol/l
Class	Poorly soluble

Pharmacokinetics	
GI absorption	High
BBB permeant	Yes
P-gp substrate	Yes
CYP1A2 inhibitor	Yes
CYP2C19 inhibitor	No
CYP2C9 inhibitor	No
CYP2D6 inhibitor	Yes
CYP3A4 inhibitor	No
Log K_p (skin permeation)	-5.84 cm/s

Druglikeness	
Lipinski	Yes; 0 violation
Ghose	Yes
Veber	Yes
Egan	Yes
Muegge	Yes
Bioavailability Score	0.55

Medicinal Chemistry	
PAINS	0 alert
Brenk	0 alert
Leadlikeness	No; 1 violation: Rotors>7
Synthetic accessibility	3.44

32	6PT3	Cc1ccccc1OC[C@@H](O)C[NH2+][C@@H](c3nc(c2ccc(N(=O)=O)cc2)no3)C(C)C

Molecule 4

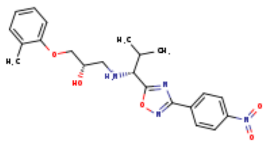

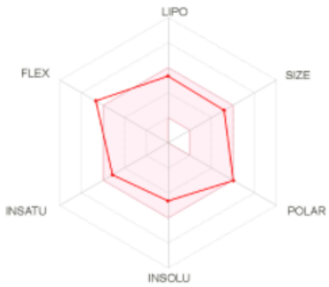

SMILES O[C@H](COc1ccccc1C)C[NH2+][C@@H](c1onc(n1)c1ccc(cc1)N(=O)=O)C(C)C

Physicochemical Properties	
Formula	C22H27N4O5
Molecular weight	427.47 g/mol
Num. heavy atoms	31
Num. arom. heavy atoms	17
Fraction Csp3	0.36
Num. rotatable bonds	10
Num. H-bond acceptors	7
Num. H-bond donors	2
Molar Refractivity	118.56
TPSA	130.81 Å²

Lipophilicity	
Log $P_{o/w}$ (iLOGP)	3.63
Log $P_{o/w}$ (XLOGP3)	3.81
Log $P_{o/w}$ (WLOGP)	2.85
Log $P_{o/w}$ (MLOGP)	-0.71
Log $P_{o/w}$ (SILICOS-IT)	2.41
Consensus Log $P_{o/w}$	2.40

Water Solubility	
Log *S* (ESOL)	-4.64
Solubility	9.87e-03 mg/ml ; 2.31e-05 mol/l
Class	Moderately soluble
Log *S* (Ali)	-6.25
Solubility	2.39e-04 mg/ml ; 5.60e-07 mol/l
Class	Poorly soluble
Log *S* (SILICOS-IT)	-6.99
Solubility	4.35e-05 mg/ml ; 1.02e-07 mol/l
Class	Poorly soluble

Pharmacokinetics	
GI absorption	High
BBB permeant	No
P-gp substrate	No
CYP1A2 inhibitor	No
CYP2C19 inhibitor	No
CYP2C9 inhibitor	Yes
CYP2D6 inhibitor	No
CYP3A4 inhibitor	Yes
Log K_p (skin permeation)	-6.20 cm/s

Druglikeness	
Lipinski	Yes; 0 violation
Ghose	Yes
Veber	Yes
Egan	Yes
Muegge	Yes
Bioavailability Score	0.55

Medicinal Chemistry	
PAINS	0 alert
Brenk	1 alert: nitro_group
Leadlikeness	No; 3 violations: MW>350, Rotors>7, XLOGP3>3.5
Synthetic accessibility	4.50

Figure S22. The predicted ADME properties for the top 32 ZINC compounds based on the crystal conformation (PDB ID: 6PT3) including the reference compound from the SwissADME server.
